# Supplementary figures and images for: Correlation analysis of m6A-modified regulators with immune microenvironment infiltrating cells in lung adenocarcinoma
Source: PLoS One. 2022 Feb 23;17(2):e0264384. doi: 10.1371/journal.pone.0264384 (PMC8865675; doi:10.1371/journal.pone.0264384)

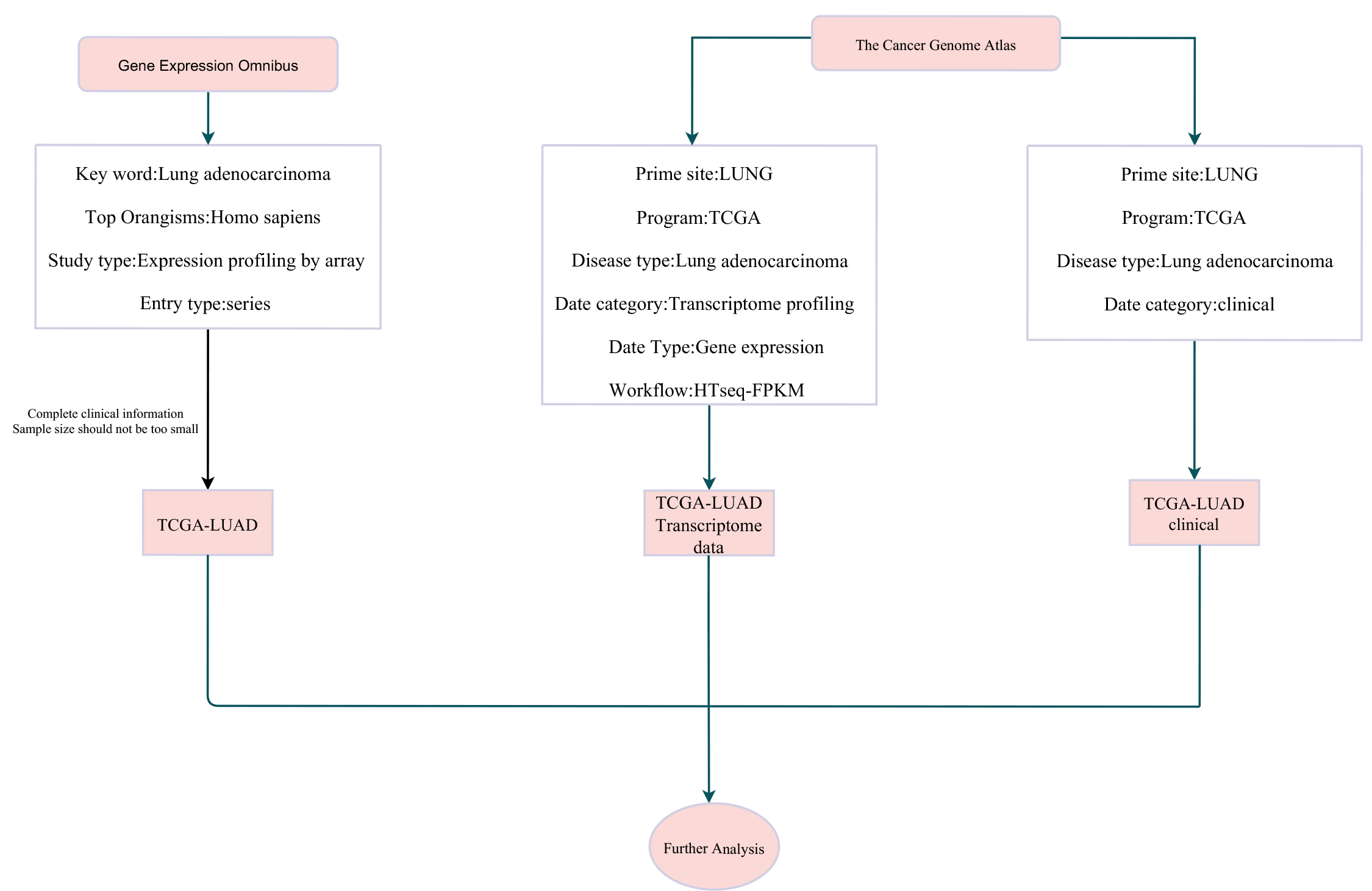

Supplement: S1 Fig — (TIF) [file pone.0264384.s001.tif]

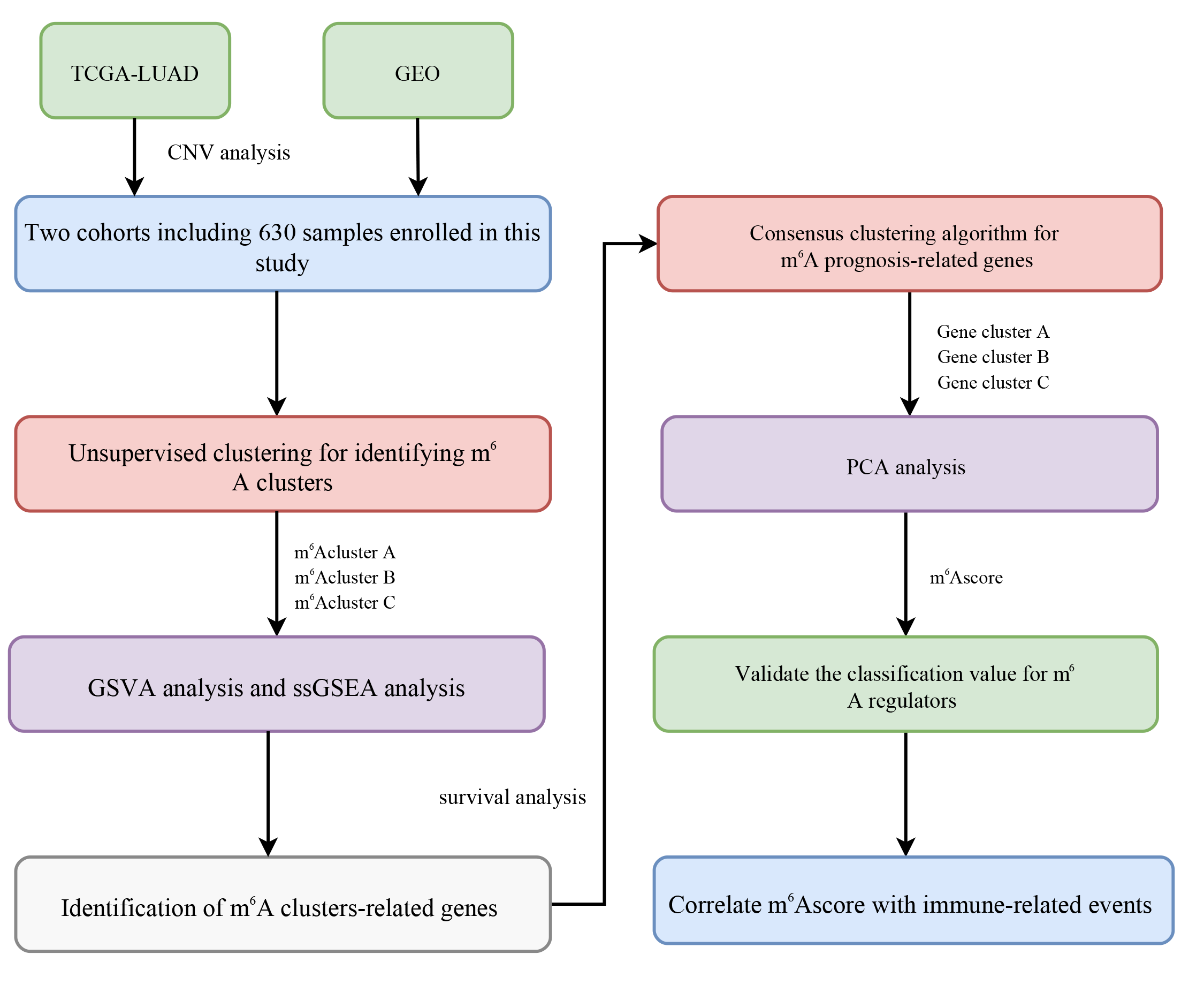

Supplement: S2 Fig — (TIF) [file pone.0264384.s002.tif]
