# Supplementary material for: Correlation analysis of m6A-modified regulators with immune microenvironment infiltrating cells in lung adenocarcinoma
Source: PLoS One. 2022 Feb 23;17(2):e0264384. doi: 10.1371/journal.pone.0264384 (PMC8865675; doi:10.1371/journal.pone.0264384)
Supplement: S1 Table — (DOCX) [file pone.0264384.s003.docx]

**S1 Table Prognostic analysis of m6A regulators using a univariate Cox regression model**

| **ID** | **HR** | **HR.95L** | **HR.95H** | **pvalue** | **km** |
| --- | --- | --- | --- | --- | --- |
| METTL3 | 0.819308401 | 0.67041707 | 1.001266652 | 0.051463617 | 0.00568808 |
| METTL16 | 0.935878855 | 0.717083453 | 1.221432772 | 0.625725559 | 0.115294495 |
| WTAP | 1.008463125 | 0.761840777 | 1.334921817 | 0.953033461 | 0.123753693 |
| VIRMA | 1.188825665 | 0.927588669 | 1.523634893 | 0.171866121 | 0.003149851 |
| ZC3H13 | 0.929391403 | 0.743365834 | 1.161969439 | 0.520484794 | 0.080176283 |
| RBM15 | 1.217526011 | 0.932501414 | 1.589670068 | 0.148066046 | 0.005375679 |
| RBM15B | 1.216757259 | 0.894539775 | 1.655039014 | 0.211323328 | 0.092295491 |
| YTHDC1 | 0.792289687 | 0.565751279 | 1.109538718 | 0.175409932 | 0.039680788 |
| YTHDC2 | 0.790673724 | 0.625865534 | 0.998880596 | 0.048912304 | 0.00297875 |
| YTHDF1 | 0.78791376 | 0.59486804 | 1.043606399 | 0.09645107 | 0.031566184 |
| YTHDF2 | 0.78891745 | 0.562419692 | 1.106630425 | 0.169703309 | 0.002244612 |
| YTHDF3 | 1.119632771 | 0.870030819 | 1.440842686 | 0.379896931 | 0.074858278 |
| HNRNPC | 1.478633055 | 1.061606197 | 2.059479038 | 0.020689572 | 0.000157831 |
| FMR1 | 0.858061375 | 0.676703126 | 1.088024121 | 0.206377164 | 0.00514653 |
| LRPPRC | 1.079969363 | 0.85532902 | 1.363608387 | 0.517899116 | 0.027817588 |
| HNRNPA2B1 | 1.235065719 | 0.931249199 | 1.638001227 | 0.142776704 | 0.000509657 |
| IGFBP2 | 0.95323244 | 0.891881726 | 1.018803345 | 0.158207865 | 0.030887034 |
| IGFBP3 | 1.119024685 | 1.018247839 | 1.2297755 | 0.019516312 | 0.004700864 |
| FTO | 0.930472985 | 0.730170667 | 1.185722758 | 0.560138842 | 0.220548152 |
| ALKBH5 | 1.021436727 | 0.75017926 | 1.390778235 | 0.892860306 | 0.036797409 |
