# Supplementary material for: Correlation analysis of m6A-modified regulators with immune microenvironment infiltrating cells in lung adenocarcinoma
Source: PLoS One. 2022 Feb 23;17(2):e0264384. doi: 10.1371/journal.pone.0264384 (PMC8865675; doi:10.1371/journal.pone.0264384)
Supplement: S2 Table — (DOCX) [file pone.0264384.s004.docx]

**Table S2 The changes of m6Acluster**

| **ID** | **m6Acluster** | **ID** | **m6Acluster** | **ID** | **m6Acluster** | **ID** | **m6Acluster** |
| --- | --- | --- | --- | --- | --- | --- | --- |
| TCGA_TCGA-91-6840 | A | TCGA_TCGA-38-4627 | C | TCGA_TCGA-91-A4BD | B | TCGA_TCGA-50-6673 | B |
| TCGA_TCGA-55-6986 | B | TCGA_TCGA-93-A4JO | B | TCGA_TCGA-05-4250 | B | TCGA_TCGA-78-7542 | B |
| TCGA_TCGA-05-4395 | A | TCGA_TCGA-05-4426 | C | TCGA_TCGA-55-7994 | B | TCGA_TCGA-38-4625 | B |
| TCGA_TCGA-44-7672 | A | TCGA_TCGA-97-7937 | B | TCGA_TCGA-99-8028 | A | TCGA_TCGA-55-7570 | C |
| TCGA_TCGA-44-2662 | C | TCGA_TCGA-75-6206 | C | TCGA_TCGA-97-8171 | B | TCGA_TCGA-69-7979 | A |
| TCGA_TCGA-97-8175 | B | TCGA_TCGA-62-A472 | B | TCGA_TCGA-67-6216 | C | TCGA_TCGA-55-A4DF | C |
| TCGA_TCGA-55-8087 | A | TCGA_TCGA-99-AA5R | B | TCGA_TCGA-91-6848 | B | TCGA_TCGA-67-3773 | B |
| TCGA_TCGA-78-7160 | A | TCGA_TCGA-55-8085 | A | TCGA_TCGA-44-2665 | A | TCGA_TCGA-55-7573 | A |
| TCGA_TCGA-L4-A4E5 | A | TCGA_TCGA-44-8117 | B | TCGA_TCGA-86-7701 | C | TCGA_TCGA-50-5068 | A |
| TCGA_TCGA-97-A4LX | B | TCGA_TCGA-55-8506 | B | TCGA_TCGA-78-7156 | C | TCGA_TCGA-49-AARN | A |
| TCGA_TCGA-86-8359 | C | TCGA_TCGA-NJ-A7XG | B | TCGA_TCGA-05-5420 | B | TCGA_TCGA-78-7150 | C |
| TCGA_TCGA-44-6145 | B | TCGA_TCGA-05-4384 | C | TCGA_TCGA-55-6642 | A | TCGA_TCGA-MP-A4TA | A |
| TCGA_TCGA-78-7155 | C | TCGA_TCGA-64-1679 | C | TCGA_TCGA-78-7149 | C | TCGA_TCGA-55-7907 | A |
| TCGA_TCGA-55-7816 | B | TCGA_TCGA-73-4666 | B | TCGA_TCGA-05-4418 | A | TCGA_TCGA-55-5899 | C |
| TCGA_TCGA-99-8025 | A | TCGA_TCGA-55-8616 | A | TCGA_TCGA-05-4422 | C | TCGA_TCGA-55-7574 | B |
| TCGA_TCGA-44-A47G | A | TCGA_TCGA-38-6178 | A | TCGA_TCGA-78-7146 | B | TCGA_TCGA-44-6148 | C |
| TCGA_TCGA-55-8615 | C | TCGA_TCGA-93-7348 | C | TCGA_TCGA-86-A4P8 | B | TCGA_TCGA-MN-A4N1 | A |
| TCGA_TCGA-73-7499 | A | TCGA_TCGA-55-1594 | C | TCGA_TCGA-67-6217 | B | TCGA_TCGA-05-5429 | A |
| TCGA_TCGA-86-7711 | A | TCGA_TCGA-73-4658 | A | TCGA_TCGA-78-8640 | A | TCGA_TCGA-91-6847 | C |
| TCGA_TCGA-86-8075 | A | TCGA_TCGA-80-5607 | A | TCGA_TCGA-44-7670 | A | TCGA_TCGA-55-8511 | B |
| TCGA_TCGA-86-7955 | B | TCGA_TCGA-55-8505 | A | TCGA_TCGA-49-AARO | A | TCGA_TCGA-50-5944 | C |
| TCGA_TCGA-55-8508 | A | TCGA_TCGA-MP-A4T4 | A | TCGA_TCGA-75-7030 | A | TCGA_TCGA-55-1592 | C |
| TCGA_TCGA-67-3771 | C | TCGA_TCGA-49-4506 | A | TCGA_TCGA-50-5941 | B | TCGA_TCGA-50-8457 | B |
| TCGA_TCGA-55-A4DG | A | TCGA_TCGA-97-8172 | B | TCGA_TCGA-49-AARR | A | TCGA_TCGA-78-7159 | C |
| TCGA_TCGA-91-7771 | C | TCGA_TCGA-O1-A52J | A | TCGA_TCGA-J2-8192 | C | TCGA_TCGA-67-6215 | B |
| TCGA_TCGA-91-6849 | C | TCGA_TCGA-78-7537 | C | TCGA_TCGA-64-5779 | C | TCGA_TCGA-MP-A4T9 | A |
| TCGA_TCGA-64-5781 | B | TCGA_TCGA-73-4662 | B | TCGA_TCGA-62-A46O | B | TCGA_TCGA-97-7938 | B |
| TCGA_TCGA-44-6146 | B | TCGA_TCGA-62-8398 | A | TCGA_TCGA-50-8459 | A | TCGA_TCGA-05-5425 | B |
| TCGA_TCGA-97-7552 | B | TCGA_TCGA-49-AAQV | C | TCGA_TCGA-86-A4JF | B | TCGA_TCGA-55-7913 | A |
| TCGA_TCGA-80-5608 | C | TCGA_TCGA-55-8621 | C | TCGA_TCGA-05-4249 | B | TCGA_TCGA-05-4417 | A |
| TCGA_TCGA-91-6829 | A | TCGA_TCGA-53-7626 | B | TCGA_TCGA-J2-A4AG | A | TCGA_TCGA-75-6203 | C |
| TCGA_TCGA-49-AARE | A | TCGA_TCGA-44-7669 | C | TCGA_TCGA-44-7671 | C | TCGA_TCGA-05-4434 | A |
| TCGA_TCGA-50-5946 | A | TCGA_TCGA-93-8067 | A | TCGA_TCGA-44-6774 | C | TCGA_TCGA-55-8096 | C |
| TCGA_TCGA-99-7458 | A | TCGA_TCGA-97-A4M2 | B | TCGA_TCGA-50-5066 | A | TCGA_TCGA-49-6767 | B |
| TCGA_TCGA-05-4424 | A | TCGA_TCGA-05-4425 | B | TCGA_TCGA-50-6594 | B | TCGA_TCGA-MP-A4T6 | B |
| TCGA_TCGA-44-2666 | C | TCGA_TCGA-69-7763 | B | TCGA_TCGA-44-8119 | A | TCGA_TCGA-MP-A4TE | C |
| TCGA_TCGA-44-6775 | A | TCGA_TCGA-86-8056 | B | TCGA_TCGA-49-AAR2 | C | GSE26939_GSM663284 | C |
| TCGA_TCGA-38-4631 | C | TCGA_TCGA-50-5931 | C | TCGA_TCGA-44-6779 | B | GSE26939_GSM663285 | B |
| TCGA_TCGA-55-7283 | B | TCGA_TCGA-55-8512 | A | TCGA_TCGA-86-8669 | A | GSE26939_GSM663286 | C |
| TCGA_TCGA-95-7567 | B | TCGA_TCGA-75-7025 | C | TCGA_TCGA-05-5715 | B | GSE26939_GSM663287 | B |
| TCGA_TCGA-38-4629 | A | TCGA_TCGA-50-5930 | A | TCGA_TCGA-69-A59K | B | GSE26939_GSM663288 | A |
| TCGA_TCGA-91-8497 | C | TCGA_TCGA-75-6214 | A | TCGA_TCGA-55-A491 | A | GSE26939_GSM663289 | C |
| TCGA_TCGA-78-7540 | B | TCGA_TCGA-L9-A743 | A | TCGA_TCGA-71-8520 | A | GSE26939_GSM663290 | C |
| TCGA_TCGA-55-A48Y | B | TCGA_TCGA-50-5939 | B | TCGA_TCGA-91-8496 | C | GSE26939_GSM663291 | A |
| TCGA_TCGA-55-7995 | A | TCGA_TCGA-78-7161 | C | TCGA_TCGA-69-8254 | B | GSE26939_GSM663292 | A |
| TCGA_TCGA-44-3919 | C | TCGA_TCGA-44-3398 | A | TCGA_TCGA-95-A4VP | A | GSE26939_GSM663293 | A |
| TCGA_TCGA-69-7764 | C | TCGA_TCGA-97-8547 | A | TCGA_TCGA-95-8039 | B | GSE26939_GSM663294 | C |
| TCGA_TCGA-95-7947 | B | TCGA_TCGA-86-8280 | A | TCGA_TCGA-86-8076 | A | GSE26939_GSM663295 | A |
| TCGA_TCGA-MP-A4TH | B | TCGA_TCGA-78-7166 | A | TCGA_TCGA-44-2659 | A | GSE26939_GSM663296 | C |
| TCGA_TCGA-55-7725 | B | TCGA_TCGA-91-8499 | C | TCGA_TCGA-55-8203 | A | GSE26939_GSM663297 | C |
| TCGA_TCGA-L9-A5IP | A | TCGA_TCGA-71-6725 | B | TCGA_TCGA-MP-A5C7 | C | GSE26939_GSM663298 | A |
| TCGA_TCGA-97-7554 | B | TCGA_TCGA-69-7980 | B | TCGA_TCGA-78-7220 | C | GSE26939_GSM663299 | A |
| TCGA_TCGA-55-8619 | B | TCGA_TCGA-L9-A8F4 | A | TCGA_TCGA-44-3917 | C | GSE26939_GSM663300 | A |
| TCGA_TCGA-55-7227 | A | TCGA_TCGA-05-4433 | B | TCGA_TCGA-78-7153 | C | GSE26939_GSM663301 | B |
| TCGA_TCGA-67-3770 | C | TCGA_TCGA-55-A492 | A | TCGA_TCGA-55-7910 | A | GSE26939_GSM663302 | C |
| TCGA_TCGA-78-7145 | C | TCGA_TCGA-73-4675 | C | TCGA_TCGA-64-5778 | B | GSE26939_GSM663303 | A |
| TCGA_TCGA-49-AAR3 | B | TCGA_TCGA-50-5936 | A | TCGA_TCGA-55-7815 | B | GSE26939_GSM663304 | A |
| TCGA_TCGA-44-A479 | C | TCGA_TCGA-44-7662 | A | TCGA_TCGA-55-6985 | C | GSE26939_GSM663305 | B |
| TCGA_TCGA-4B-A93V | C | TCGA_TCGA-78-7154 | B | TCGA_TCGA-69-7761 | B | GSE26939_GSM663306 | B |
| TCGA_TCGA-78-7633 | C | TCGA_TCGA-05-4244 | B | TCGA_TCGA-MN-A4N4 | A | GSE26939_GSM663307 | B |
| TCGA_TCGA-NJ-A4YP | A | TCGA_TCGA-73-7498 | C | TCGA_TCGA-55-6972 | B | GSE26939_GSM663308 | B |
| TCGA_TCGA-38-4626 | B | TCGA_TCGA-50-5933 | B | TCGA_TCGA-97-A4M1 | B | GSE26939_GSM663309 | C |
| TCGA_TCGA-78-7535 | A | TCGA_TCGA-55-8507 | C | TCGA_TCGA-83-5908 | B | GSE26939_GSM663310 | C |
| TCGA_TCGA-55-6970 | B | TCGA_TCGA-S2-AA1A | C | TCGA_TCGA-97-8179 | A | GSE26939_GSM663311 | C |
| TCGA_TCGA-55-6543 | C | TCGA_TCGA-95-7948 | C | TCGA_TCGA-64-1680 | C | GSE26939_GSM663312 | A |
| TCGA_TCGA-05-4402 | C | TCGA_TCGA-67-3774 | C | TCGA_TCGA-73-4670 | A | GSE26939_GSM663313 | C |
| TCGA_TCGA-55-1596 | A | TCGA_TCGA-38-4628 | B | TCGA_TCGA-97-8174 | B | GSE26939_GSM663314 | C |
| TCGA_TCGA-49-4490 | C | TCGA_TCGA-50-6595 | A | TCGA_TCGA-44-6777 | C | GSE26939_GSM663315 | C |
| TCGA_TCGA-62-A471 | A | TCGA_TCGA-55-7903 | B | TCGA_TCGA-62-A46P | A | GSE26939_GSM663316 | B |
| TCGA_TCGA-86-A456 | A | TCGA_TCGA-44-3396 | A | TCGA_TCGA-55-8089 | A | GSE26939_GSM663317 | C |
| TCGA_TCGA-49-4512 | A | TCGA_TCGA-80-5611 | B | TCGA_TCGA-MN-A4N5 | A | GSE26939_GSM663318 | C |
| TCGA_TCGA-75-5122 | B | TCGA_TCGA-53-7624 | A | TCGA_TCGA-49-AAR9 | B | GSE26939_GSM663319 | A |
| TCGA_TCGA-55-A48X | B | TCGA_TCGA-91-6835 | B | TCGA_TCGA-86-8671 | A | GSE26939_GSM663320 | B |
| TCGA_TCGA-86-8278 | A | TCGA_TCGA-L9-A50W | A | TCGA_TCGA-49-4507 | B | GSE26939_GSM663321 | B |
| TCGA_TCGA-55-6982 | C | TCGA_TCGA-86-7713 | C | TCGA_TCGA-62-A46V | B | GSE26939_GSM663322 | A |
| TCGA_TCGA-50-5045 | A | TCGA_TCGA-50-5044 | B | TCGA_TCGA-05-4389 | B | GSE26939_GSM663323 | B |
| TCGA_TCGA-05-4398 | A | TCGA_TCGA-97-7941 | B | TCGA_TCGA-50-6592 | A | GSE26939_GSM663324 | A |
| TCGA_TCGA-55-8513 | B | TCGA_TCGA-86-7714 | B | TCGA_TCGA-91-6831 | C | GSE26939_GSM663325 | B |
| TCGA_TCGA-MP-A4TC | A | TCGA_TCGA-62-8402 | C | TCGA_TCGA-55-6987 | A | GSE26939_GSM663326 | B |
| TCGA_TCGA-05-4420 | A | TCGA_TCGA-78-7162 | C | TCGA_TCGA-49-4510 | C | GSE26939_GSM663327 | B |
| TCGA_TCGA-75-5146 | B | TCGA_TCGA-49-AAR0 | C | TCGA_TCGA-86-8672 | A | GSE26939_GSM663328 | A |
| TCGA_TCGA-78-7167 | C | TCGA_TCGA-35-4122 | A | TCGA_TCGA-44-2668 | B | GSE26939_GSM663329 | C |
| TCGA_TCGA-J2-8194 | C | TCGA_TCGA-55-7726 | A | TCGA_TCGA-93-A4JQ | B | GSE26939_GSM663330 | A |
| TCGA_TCGA-97-A4M0 | B | TCGA_TCGA-62-A46Y | A | TCGA_TCGA-97-8177 | C | GSE26939_GSM663331 | C |
| TCGA_TCGA-38-4632 | B | TCGA_TCGA-55-6980 | A | TCGA_TCGA-05-4396 | C | GSE26939_GSM663332 | B |
| TCGA_TCGA-44-7661 | A | TCGA_TCGA-05-4430 | A | TCGA_TCGA-55-6978 | B | GSE26939_GSM663333 | A |
| TCGA_TCGA-55-8205 | B | TCGA_TCGA-67-3772 | C | TCGA_TCGA-69-8453 | B | GSE26939_GSM663334 | A |
| TCGA_TCGA-55-A48Z | A | TCGA_TCGA-MP-A4SY | A | TCGA_TCGA-50-7109 | C | GSE26939_GSM663335 | C |
| TCGA_TCGA-44-5645 | C | TCGA_TCGA-78-7148 | C | TCGA_TCGA-49-4501 | A | GSE26939_GSM663336 | A |
| TCGA_TCGA-05-5428 | A | TCGA_TCGA-35-3615 | C | TCGA_TCGA-50-5049 | A | GSE26939_GSM663337 | B |
| TCGA_TCGA-69-8255 | A | TCGA_TCGA-55-A493 | C | TCGA_TCGA-78-8660 | B | GSE26939_GSM663338 | A |
| TCGA_TCGA-75-5125 | B | TCGA_TCGA-49-6744 | A | TCGA_TCGA-91-6828 | B | GSE26939_GSM663339 | C |
| TCGA_TCGA-55-8097 | B | TCGA_TCGA-49-4494 | C | TCGA_TCGA-50-6590 | B | GSE26939_GSM663340 | A |
| TCGA_TCGA-78-7152 | C | TCGA_TCGA-78-7143 | C | TCGA_TCGA-97-8176 | B | GSE26939_GSM663341 | B |
| TCGA_TCGA-MP-A4TF | B | TCGA_TCGA-50-5051 | C | TCGA_TCGA-50-5935 | A | GSE26939_GSM663342 | B |
| TCGA_TCGA-67-4679 | B | TCGA_TCGA-86-6851 | B | TCGA_TCGA-55-7281 | B | GSE26939_GSM663343 | C |
| TCGA_TCGA-91-6836 | B | TCGA_TCGA-75-6205 | B | TCGA_TCGA-49-4488 | C | GSE26939_GSM663344 | B |
| TCGA_TCGA-78-8648 | B | TCGA_TCGA-49-6743 | A | TCGA_TCGA-NJ-A55A | C | GSE26939_GSM663345 | B |
| TCGA_TCGA-44-A47A | B | TCGA_TCGA-50-6597 | B | TCGA_TCGA-95-8494 | A | GSE26939_GSM663346 | B |
| TCGA_TCGA-55-A57B | C | TCGA_TCGA-55-A490 | A | TCGA_TCGA-44-2657 | C | GSE26939_GSM663347 | C |
| TCGA_TCGA-55-8207 | B | TCGA_TCGA-99-8032 | C | TCGA_TCGA-78-7158 | B | GSE26939_GSM663348 | B |
| TCGA_TCGA-55-7576 | B | TCGA_TCGA-55-8299 | A | TCGA_TCGA-93-7347 | C | GSE26939_GSM663349 | B |
| TCGA_TCGA-NJ-A55O | C | TCGA_TCGA-64-1676 | A | TCGA_TCGA-38-4630 | C | GSE26939_GSM663350 | B |
| TCGA_TCGA-55-A494 | A | TCGA_TCGA-86-8279 | A | TCGA_TCGA-86-7953 | C | GSE26939_GSM663351 | A |
| TCGA_TCGA-95-7043 | B | TCGA_TCGA-97-A4M5 | C | TCGA_TCGA-50-5072 | C | GSE26939_GSM663352 | B |
| TCGA_TCGA-L9-A443 | A | TCGA_TCGA-78-7536 | A | TCGA_TCGA-62-A46S | C | GSE26939_GSM663353 | A |
| TCGA_TCGA-69-7974 | B | TCGA_TCGA-55-8208 | A | TCGA_TCGA-53-A4EZ | C | GSE26939_GSM663354 | C |
| TCGA_TCGA-NJ-A4YG | B | TCGA_TCGA-95-7562 | C | TCGA_TCGA-69-7973 | A | GSE26939_GSM663355 | B |
| TCGA_TCGA-69-7760 | A | TCGA_TCGA-MP-A4TK | A | TCGA_TCGA-55-8204 | C | GSE26939_GSM663356 | B |
| TCGA_TCGA-49-4486 | C | TCGA_TCGA-49-6761 | C | TCGA_TCGA-35-5375 | C | GSE26939_GSM663357 | A |
| TCGA_TCGA-93-A4JN | A | TCGA_TCGA-50-6593 | A | TCGA_TCGA-78-7539 | B | GSE26939_GSM663358 | B |
| TCGA_TCGA-86-8073 | B | TCGA_TCGA-55-7728 | B | TCGA_TCGA-95-A4VK | A | GSE26939_GSM663359 | B |
| TCGA_TCGA-L9-A7SV | B | TCGA_TCGA-86-8055 | C | TCGA_TCGA-50-5055 | A | GSE26939_GSM663360 | B |
| TCGA_TCGA-93-A4JP | B | TCGA_TCGA-73-4668 | A | TCGA_TCGA-44-7667 | C | GSE26939_GSM663361 | B |
| TCGA_TCGA-78-7163 | C | TCGA_TCGA-55-8090 | C | TCGA_TCGA-MP-A4TI | A | GSE26939_GSM663362 | C |
| TCGA_TCGA-64-1678 | C | TCGA_TCGA-38-7271 | B | TCGA_TCGA-55-6979 | A | GSE26939_GSM663363 | B |
| TCGA_TCGA-NJ-A55R | C | TCGA_TCGA-55-8514 | C | TCGA_TCGA-55-7284 | B | GSE26939_GSM663364 | C |
| TCGA_TCGA-97-A4M7 | C | TCGA_TCGA-50-5932 | B | TCGA_TCGA-44-2656 | B | GSE26939_GSM663365 | B |
| TCGA_TCGA-38-A44F | A | TCGA_TCGA-95-A4VN | A | TCGA_TCGA-44-6778 | C | GSE26939_GSM663366 | B |
| TCGA_TCGA-62-8399 | A | TCGA_TCGA-86-7954 | A | TCGA_TCGA-35-4123 | A | GSE26939_GSM663367 | A |
| TCGA_TCGA-49-4514 | A | TCGA_TCGA-91-A4BC | C | TCGA_TCGA-44-6776 | C | GSE26939_GSM663368 | C |
| TCGA_TCGA-44-5643 | C | TCGA_TCGA-MP-A4TJ | A | TCGA_TCGA-73-4676 | B | GSE26939_GSM663369 | A |
| TCGA_TCGA-44-6147 | A | TCGA_TCGA-75-5126 | A | TCGA_TCGA-86-8673 | C | GSE26939_GSM663370 | A |
| TCGA_TCGA-J2-A4AE | A | TCGA_TCGA-75-6211 | C | TCGA_TCGA-62-8395 | B | GSE26939_GSM663371 | B |
| TCGA_TCGA-05-4415 | A | TCGA_TCGA-62-A470 | C | TCGA_TCGA-44-A4SS | A | GSE26939_GSM663372 | A |
| TCGA_TCGA-91-6830 | A | TCGA_TCGA-73-4677 | C | TCGA_TCGA-86-8358 | C | GSE26939_GSM663373 | B |
| TCGA_TCGA-49-6745 | B | TCGA_TCGA-55-7911 | B | TCGA_TCGA-55-6968 | C | GSE26939_GSM663374 | A |
| TCGA_TCGA-86-A4P7 | B | TCGA_TCGA-50-8460 | C | TCGA_TCGA-64-5775 | B | GSE26939_GSM663375 | A |
| TCGA_TCGA-44-4112 | A | TCGA_TCGA-55-6981 | C | TCGA_TCGA-97-7547 | C | GSE26939_GSM663376 | B |
| TCGA_TCGA-55-6975 | C | TCGA_TCGA-44-8120 | A | TCGA_TCGA-75-7031 | C | GSE26939_GSM663377 | A |
| TCGA_TCGA-95-7039 | A | TCGA_TCGA-55-8614 | C | TCGA_TCGA-44-2655 | C | GSE26939_GSM663378 | B |
| TCGA_TCGA-05-4432 | C | TCGA_TCGA-64-1677 | C | TCGA_TCGA-75-6212 | C | GSE26939_GSM663379 | C |
| TCGA_TCGA-49-4487 | A | TCGA_TCGA-97-8552 | A | TCGA_TCGA-05-4390 | C | GSE26939_GSM663380 | A |
| TCGA_TCGA-55-7727 | A | TCGA_TCGA-69-8253 | C | TCGA_TCGA-86-8674 | C | GSE26939_GSM663381 | C |
| TCGA_TCGA-73-A9RS | B | TCGA_TCGA-86-8668 | C | TCGA_TCGA-49-6742 | C | GSE26939_GSM663382 | B |
| TCGA_TCGA-44-5644 | C | TCGA_TCGA-86-8074 | C | TCGA_TCGA-44-A47B | B | GSE26939_GSM663383 | B |
| TCGA_TCGA-55-7914 | B | TCGA_TCGA-69-7765 | A | TCGA_TCGA-MP-A4SW | C | GSE26939_GSM663384 | A |
| TCGA_TCGA-62-A46R | B | TCGA_TCGA-55-8092 | A | TCGA_TCGA-64-5815 | A | GSE26939_GSM663385 | A |
| TCGA_TCGA-49-AARQ | C | TCGA_TCGA-NJ-A4YQ | A | TCGA_TCGA-MP-A4T8 | C | GSE26939_GSM663386 | B |
| TCGA_TCGA-75-6207 | B | TCGA_TCGA-55-6983 | C | TCGA_TCGA-NJ-A4YI | B | GSE26939_GSM663387 | C |
| TCGA_TCGA-53-7813 | C | TCGA_TCGA-62-8394 | A | TCGA_TCGA-L4-A4E6 | B | GSE26939_GSM663388 | B |
| TCGA_TCGA-44-3918 | A | TCGA_TCGA-44-2661 | C | TCGA_TCGA-55-8302 | A | GSE26939_GSM663389 | B |
| TCGA_TCGA-64-1681 | C | TCGA_TCGA-44-7659 | B | TCGA_TCGA-05-4405 | B | GSE26939_GSM663390 | C |
| TCGA_TCGA-05-4410 | A | TCGA_TCGA-55-6712 | A | TCGA_TCGA-78-7147 | C | GSE26939_GSM663391 | B |
| TCGA_TCGA-55-8620 | C | TCGA_TCGA-05-4403 | B | TCGA_TCGA-MP-A4TD | C | GSE26939_GSM663392 | C |
| TCGA_TCGA-64-5774 | C | TCGA_TCGA-97-7553 | B | TCGA_TCGA-55-6971 | C | GSE26939_GSM663393 | B |
| TCGA_TCGA-86-A4D0 | C | TCGA_TCGA-78-8655 | B | TCGA_TCGA-50-6591 | C | GSE26939_GSM663394 | B |
| TCGA_TCGA-L9-A444 | A | TCGA_TCGA-55-7724 | A | TCGA_TCGA-95-7944 | B | GSE26939_GSM663395 | B |
| TCGA_TCGA-MP-A4T7 | B | TCGA_TCGA-73-4659 | B | TCGA_TCGA-NJ-A4YF | C | GSE26939_GSM663396 | C |
| TCGA_TCGA-05-4382 | A | TCGA_TCGA-75-5147 | A | TCGA_TCGA-55-8091 | C | GSE26939_GSM663397 | B |
| TCGA_TCGA-05-5423 | B | TCGA_TCGA-97-7546 | B | TCGA_TCGA-49-4505 | A | GSE26939_GSM663398 | C |
| TCGA_TCGA-49-AAR4 | C | TCGA_TCGA-86-8585 | A | TCGA_TCGA-62-8397 | B | GSE26939_GSM663399 | A |
| TCGA_TCGA-97-A4M3 | C | TCGA_TCGA-55-8301 | A | TCGA_TCGA-50-5942 | A | TCGA_TCGA-05-4427 | B |
| TCGA_TCGA-05-4397 | A | TCGA_TCGA-86-6562 | A | TCGA_TCGA-55-8206 | B | TCGA_TCGA-86-8281 | C |
| TCGA_TCGA-55-6984 | C | TCGA_TCGA-J2-A4AD | A | TCGA_TCGA-55-8094 | B | TCGA_TCGA-44-A4SU | C |
| TCGA_TCGA-86-8054 | A | TCGA_TCGA-55-8510 | A | TCGA_TCGA-78-8662 | A | TCGA_TCGA-69-7978 | A |
| TCGA_TCGA-44-7660 | C | TCGA_TCGA-99-8033 | A | TCGA_TCGA-75-7027 | C | TCGA_TCGA-MP-A4SV | A |
| TCGA_TCGA-97-A4M6 | A |  |  |  |  |  |  |
