# Supplementary material for: Correlation analysis of m6A-modified regulators with immune microenvironment infiltrating cells in lung adenocarcinoma
Source: PLoS One. 2022 Feb 23;17(2):e0264384. doi: 10.1371/journal.pone.0264384 (PMC8865675; doi:10.1371/journal.pone.0264384)
Supplement: S3 Table — (DOCX) [file pone.0264384.s005.docx]

**S3 Table Prognostic analysis of Intersecting genes using a univariate Cox regression model**

| **ID** | **HR** | **HR.95L** | **HR.95H** | **Pvalue** |
| --- | --- | --- | --- | --- |
| IGFBP3 | 1.119026609 | 1.018245787 | 1.229782206 | 0.019519271 |
| TMEM158 | 1.136160958 | 1.011795741 | 1.275812567 | 0.030910425 |
| TWIST1 | 1.147440766 | 1.031188858 | 1.276798427 | 0.011620149 |
| COL7A1 | 1.107835888 | 1.032122768 | 1.189103074 | 0.004577533 |
| TPM2 | 1.143300465 | 1.005876563 | 1.299499363 | 0.040399389 |
| KRT17 | 1.077443546 | 1.01927876 | 1.138927485 | 0.008429665 |
| TMEM45A | 1.104408618 | 1.012589317 | 1.204553885 | 0.024931559 |
| FKBP9 | 1.286291529 | 1.064003098 | 1.555019812 | 0.009299204 |
| P2RY12 | 0.754776855 | 0.63114647 | 0.902624238 | 0.002052995 |
| PLEKHM1 | 0.781183855 | 0.633551439 | 0.963218103 | 0.020854178 |
| C1orf116 | 0.917659405 | 0.854694715 | 0.985262655 | 0.017819611 |
| GFPT2 | 1.161228206 | 1.030124459 | 1.309017502 | 0.014463651 |
| ARHGEF3 | 0.76349978 | 0.615487309 | 0.94710631 | 0.014118927 |
| CNGA3 | 0.882242847 | 0.809652051 | 0.961341901 | 0.004237607 |
| PRRX2 | 1.132042224 | 1.030737121 | 1.24330401 | 0.009517289 |
| PTGES | 1.151868573 | 1.041651809 | 1.273747328 | 0.005865869 |
| TLR2 | 0.84723234 | 0.762858942 | 0.940937569 | 0.001952229 |
| NLRC4 | 0.812654299 | 0.696252827 | 0.94851609 | 0.008536206 |
| SPN | 0.859634445 | 0.767133227 | 0.963289495 | 0.009218106 |
| TSPAN11 | 0.870530961 | 0.799384586 | 0.948009465 | 0.001436081 |
| SAMD9 | 1.15305646 | 1.023553589 | 1.298944396 | 0.019131261 |
| PRKCA | 1.154554095 | 1.023947543 | 1.301819774 | 0.01895918 |
| MAP3K3 | 0.734411352 | 0.604502652 | 0.892237666 | 0.001883579 |
| TMEM59L | 0.938556087 | 0.881808068 | 0.998956078 | 0.046284765 |
| DIAPH1 | 1.392645343 | 1.082143366 | 1.792240392 | 0.01007272 |
| ACADSB | 0.759956233 | 0.653754478 | 0.883410356 | 0.000351492 |
| HOXD8 | 1.174754698 | 1.04843162 | 1.316298148 | 0.005523838 |
| MARVELD1 | 1.348285233 | 1.14564321 | 1.586770692 | 0.000322903 |
| RASGRP4 | 0.800343686 | 0.662512449 | 0.96684978 | 0.020912446 |
| TNFRSF19 | 0.874671998 | 0.788576211 | 0.970167617 | 0.011314589 |
| ANKRD44 | 0.774433501 | 0.67597734 | 0.88722981 | 0.000228999 |
| COL6A2 | 1.134608449 | 1.015141693 | 1.268134628 | 0.026101166 |
| ZNF700 | 0.764376311 | 0.622464828 | 0.938641218 | 0.010339226 |
| CAPN13 | 0.894646281 | 0.836026261 | 0.957376586 | 0.001283069 |
| CD300LF | 0.840799194 | 0.745216509 | 0.94864147 | 0.004858423 |
| RGS18 | 0.849539117 | 0.734132445 | 0.983087883 | 0.02860165 |
| MNDA | 0.887561346 | 0.805507517 | 0.97797367 | 0.015953617 |
| KRT80 | 1.181569291 | 1.066395161 | 1.3091826 | 0.001430314 |
