# Supplementary material for: Correlation analysis of m6A-modified regulators with immune microenvironment infiltrating cells in lung adenocarcinoma
Source: PLoS One. 2022 Feb 23;17(2):e0264384. doi: 10.1371/journal.pone.0264384 (PMC8865675; doi:10.1371/journal.pone.0264384)
Supplement: S4 Table — (DOCX) [file pone.0264384.s006.docx]

**S4 Table The changes of Genotyping**

| **ID** | **genetype** | **ID** | **genetype** | **ID** | **genetype** | **ID** | **genetype** |
| --- | --- | --- | --- | --- | --- | --- | --- |
| TCGA_TCGA-91-6840 | A | TCGA_TCGA-55-8091 | B | TCGA_TCGA-MP-A4T7 | C | TCGA_TCGA-95-A4VN | B |
| TCGA_TCGA-55-6986 | B | TCGA_TCGA-49-4505 | B | TCGA_TCGA-91-A4BD | B | TCGA_TCGA-86-7954 | B |
| TCGA_TCGA-05-4395 | C | TCGA_TCGA-62-8397 | B | TCGA_TCGA-05-4250 | C | TCGA_TCGA-91-A4BC | A |
| TCGA_TCGA-44-7672 | B | TCGA_TCGA-50-5942 | B | TCGA_TCGA-55-7994 | B | TCGA_TCGA-MP-A4TJ | A |
| TCGA_TCGA-44-2662 | B | TCGA_TCGA-55-8206 | B | TCGA_TCGA-99-8028 | B | TCGA_TCGA-75-5126 | B |
| TCGA_TCGA-97-8175 | B | TCGA_TCGA-55-8094 | C | TCGA_TCGA-97-8171 | B | TCGA_TCGA-75-6211 | A |
| TCGA_TCGA-55-8087 | A | TCGA_TCGA-05-4382 | C | TCGA_TCGA-67-6216 | B | TCGA_TCGA-62-A470 | A |
| TCGA_TCGA-78-7160 | B | TCGA_TCGA-05-5423 | B | TCGA_TCGA-91-6848 | B | TCGA_TCGA-73-4677 | A |
| TCGA_TCGA-L4-A4E5 | C | TCGA_TCGA-49-AAR4 | A | TCGA_TCGA-44-2665 | C | TCGA_TCGA-55-7911 | B |
| TCGA_TCGA-97-A4LX | B | TCGA_TCGA-97-A4M3 | A | TCGA_TCGA-86-7701 | B | TCGA_TCGA-50-8460 | B |
| TCGA_TCGA-86-8359 | A | TCGA_TCGA-05-4397 | C | TCGA_TCGA-78-7156 | A | TCGA_TCGA-55-6981 | A |
| TCGA_TCGA-44-6145 | C | TCGA_TCGA-55-6984 | C | TCGA_TCGA-05-5420 | B | TCGA_TCGA-44-8120 | A |
| TCGA_TCGA-78-7155 | A | TCGA_TCGA-05-4427 | B | TCGA_TCGA-55-6642 | B | TCGA_TCGA-55-8614 | C |
| TCGA_TCGA-55-7816 | B | TCGA_TCGA-86-8281 | A | TCGA_TCGA-78-7149 | A | TCGA_TCGA-64-1677 | A |
| TCGA_TCGA-99-8025 | A | TCGA_TCGA-44-A4SU | A | TCGA_TCGA-05-4418 | C | TCGA_TCGA-97-8552 | B |
| TCGA_TCGA-44-A47G | B | TCGA_TCGA-75-5147 | B | TCGA_TCGA-05-4422 | B | TCGA_TCGA-69-8253 | A |
| TCGA_TCGA-55-8615 | A | TCGA_TCGA-97-7546 | B | TCGA_TCGA-78-7146 | C | TCGA_TCGA-86-8668 | B |
| TCGA_TCGA-73-7499 | B | TCGA_TCGA-86-8585 | C | TCGA_TCGA-86-A4P8 | B | TCGA_TCGA-86-8074 | C |
| TCGA_TCGA-86-7711 | C | TCGA_TCGA-55-8301 | B | TCGA_TCGA-67-6217 | B | TCGA_TCGA-69-7765 | B |
| TCGA_TCGA-86-8075 | C | TCGA_TCGA-86-6562 | B | TCGA_TCGA-78-8640 | C | TCGA_TCGA-55-8092 | C |
| TCGA_TCGA-86-7955 | C | TCGA_TCGA-J2-A4AD | C | TCGA_TCGA-44-7670 | A | TCGA_TCGA-NJ-A4YQ | B |
| TCGA_TCGA-55-8508 | A | TCGA_TCGA-55-8510 | B | TCGA_TCGA-49-AARO | B | TCGA_TCGA-55-6983 | A |
| TCGA_TCGA-67-3771 | B | TCGA_TCGA-99-8033 | C | TCGA_TCGA-75-7030 | C | TCGA_TCGA-62-8394 | C |
| TCGA_TCGA-55-A4DG | B | TCGA_TCGA-69-7978 | B | TCGA_TCGA-50-5941 | B | TCGA_TCGA-44-2661 | B |
| TCGA_TCGA-91-7771 | B | TCGA_TCGA-86-8054 | A | TCGA_TCGA-49-AARR | B | TCGA_TCGA-44-7659 | B |
| TCGA_TCGA-91-6849 | A | TCGA_TCGA-44-7660 | C | TCGA_TCGA-J2-8192 | B | TCGA_TCGA-55-6712 | B |
| TCGA_TCGA-64-5781 | C | TCGA_TCGA-78-8662 | C | TCGA_TCGA-64-5779 | A | TCGA_TCGA-05-4403 | B |
| TCGA_TCGA-44-6146 | C | TCGA_TCGA-75-7027 | C | TCGA_TCGA-62-A46O | C | TCGA_TCGA-97-7553 | B |
| TCGA_TCGA-97-7552 | B | TCGA_TCGA-97-A4M6 | B | TCGA_TCGA-50-8459 | C | TCGA_TCGA-78-8655 | B |
| TCGA_TCGA-80-5608 | A | TCGA_TCGA-MP-A4SV | B | TCGA_TCGA-86-A4JF | B | TCGA_TCGA-55-7724 | B |
| TCGA_TCGA-91-6829 | A | TCGA_TCGA-38-4627 | C | TCGA_TCGA-05-4249 | B | TCGA_TCGA-73-4659 | A |
| TCGA_TCGA-49-AARE | C | TCGA_TCGA-93-A4JO | B | TCGA_TCGA-J2-A4AG | B | TCGA_TCGA-50-6673 | B |
| TCGA_TCGA-50-5946 | A | TCGA_TCGA-05-4426 | B | TCGA_TCGA-44-7671 | A | TCGA_TCGA-78-7542 | C |
| TCGA_TCGA-99-7458 | B | TCGA_TCGA-97-7937 | A | TCGA_TCGA-44-6774 | A | TCGA_TCGA-38-4625 | B |
| TCGA_TCGA-05-4424 | B | TCGA_TCGA-75-6206 | A | TCGA_TCGA-50-5066 | B | TCGA_TCGA-55-7570 | C |
| TCGA_TCGA-44-2666 | B | TCGA_TCGA-62-A472 | B | TCGA_TCGA-50-6594 | B | TCGA_TCGA-69-7979 | C |
| TCGA_TCGA-44-6775 | B | TCGA_TCGA-99-AA5R | B | TCGA_TCGA-44-8119 | A | TCGA_TCGA-55-A4DF | B |
| TCGA_TCGA-38-4631 | C | TCGA_TCGA-55-8085 | A | TCGA_TCGA-49-AAR2 | C | TCGA_TCGA-67-3773 | B |
| TCGA_TCGA-55-7283 | B | TCGA_TCGA-44-8117 | B | TCGA_TCGA-44-6779 | C | TCGA_TCGA-55-7573 | B |
| TCGA_TCGA-95-7567 | B | TCGA_TCGA-55-8506 | B | TCGA_TCGA-86-8669 | A | TCGA_TCGA-50-5068 | B |
| TCGA_TCGA-38-4629 | C | TCGA_TCGA-NJ-A7XG | C | TCGA_TCGA-05-5715 | B | TCGA_TCGA-49-AARN | B |
| TCGA_TCGA-91-8497 | A | TCGA_TCGA-05-4384 | A | TCGA_TCGA-69-A59K | B | TCGA_TCGA-78-7150 | C |
| TCGA_TCGA-78-7540 | C | TCGA_TCGA-64-1679 | C | TCGA_TCGA-55-A491 | A | TCGA_TCGA-MP-A4TA | C |
| TCGA_TCGA-55-A48Y | A | TCGA_TCGA-73-4666 | B | TCGA_TCGA-71-8520 | B | TCGA_TCGA-55-7907 | B |
| TCGA_TCGA-55-7995 | B | TCGA_TCGA-55-8616 | C | TCGA_TCGA-91-8496 | B | TCGA_TCGA-55-5899 | C |
| TCGA_TCGA-44-3919 | B | TCGA_TCGA-38-6178 | C | TCGA_TCGA-69-8254 | B | TCGA_TCGA-55-7574 | A |
| TCGA_TCGA-69-7764 | A | TCGA_TCGA-93-7348 | A | TCGA_TCGA-95-A4VP | A | TCGA_TCGA-44-6148 | A |
| TCGA_TCGA-95-7947 | B | TCGA_TCGA-55-1594 | A | TCGA_TCGA-95-8039 | B | TCGA_TCGA-MN-A4N1 | A |
| TCGA_TCGA-MP-A4TH | A | TCGA_TCGA-73-4658 | B | TCGA_TCGA-86-8076 | A | TCGA_TCGA-05-5429 | C |
| TCGA_TCGA-55-7725 | B | TCGA_TCGA-80-5607 | B | TCGA_TCGA-44-2659 | B | TCGA_TCGA-91-6847 | A |
| TCGA_TCGA-L9-A5IP | C | TCGA_TCGA-55-8505 | C | TCGA_TCGA-55-8203 | A | TCGA_TCGA-55-8511 | C |
| TCGA_TCGA-97-7554 | B | TCGA_TCGA-MP-A4T4 | B | TCGA_TCGA-MP-A5C7 | A | TCGA_TCGA-50-5944 | B |
| TCGA_TCGA-55-8619 | B | TCGA_TCGA-49-4506 | C | TCGA_TCGA-78-7220 | C | TCGA_TCGA-55-1592 | B |
| TCGA_TCGA-55-7227 | B | TCGA_TCGA-97-8172 | B | TCGA_TCGA-44-3917 | A | TCGA_TCGA-50-8457 | B |
| TCGA_TCGA-67-3770 | B | TCGA_TCGA-O1-A52J | B | TCGA_TCGA-78-7153 | A | TCGA_TCGA-78-7159 | A |
| TCGA_TCGA-78-7145 | B | TCGA_TCGA-78-7537 | A | TCGA_TCGA-55-7910 | A | TCGA_TCGA-67-6215 | B |
| TCGA_TCGA-49-AAR3 | B | TCGA_TCGA-73-4662 | B | TCGA_TCGA-64-5778 | B | TCGA_TCGA-MP-A4T9 | B |
| TCGA_TCGA-44-A479 | A | TCGA_TCGA-62-8398 | C | TCGA_TCGA-55-7815 | B | TCGA_TCGA-97-7938 | B |
| TCGA_TCGA-4B-A93V | A | TCGA_TCGA-49-AAQV | C | TCGA_TCGA-55-6985 | A | TCGA_TCGA-05-5425 | B |
| TCGA_TCGA-78-7633 | A | TCGA_TCGA-55-8621 | B | TCGA_TCGA-69-7761 | B | TCGA_TCGA-55-7913 | A |
| TCGA_TCGA-NJ-A4YP | C | TCGA_TCGA-53-7626 | B | TCGA_TCGA-MN-A4N4 | C | TCGA_TCGA-05-4417 | B |
| TCGA_TCGA-38-4626 | B | TCGA_TCGA-44-7669 | B | TCGA_TCGA-55-6972 | A | TCGA_TCGA-75-6203 | B |
| TCGA_TCGA-78-7535 | C | TCGA_TCGA-93-8067 | A | TCGA_TCGA-97-A4M1 | B | TCGA_TCGA-05-4434 | C |
| TCGA_TCGA-55-6970 | B | TCGA_TCGA-97-A4M2 | B | TCGA_TCGA-83-5908 | B | TCGA_TCGA-55-8096 | B |
| TCGA_TCGA-55-6543 | A | TCGA_TCGA-05-4425 | B | TCGA_TCGA-97-8179 | A | TCGA_TCGA-49-6767 | C |
| TCGA_TCGA-05-4402 | A | TCGA_TCGA-69-7763 | A | TCGA_TCGA-64-1680 | B | TCGA_TCGA-MP-A4T6 | B |
| TCGA_TCGA-55-1596 | C | TCGA_TCGA-86-8056 | B | TCGA_TCGA-73-4670 | C | TCGA_TCGA-MP-A4TE | A |
| TCGA_TCGA-49-4490 | C | TCGA_TCGA-50-5931 | C | TCGA_TCGA-97-8174 | A | GSE26939_GSM663284 | A |
| TCGA_TCGA-62-A471 | C | TCGA_TCGA-55-8512 | A | TCGA_TCGA-44-6777 | B | GSE26939_GSM663285 | B |
| TCGA_TCGA-86-A456 | B | TCGA_TCGA-75-7025 | B | TCGA_TCGA-62-A46P | A | GSE26939_GSM663286 | B |
| TCGA_TCGA-49-4512 | B | TCGA_TCGA-50-5930 | C | TCGA_TCGA-55-8089 | B | GSE26939_GSM663287 | B |
| TCGA_TCGA-75-5122 | B | TCGA_TCGA-75-6214 | C | TCGA_TCGA-MN-A4N5 | B | GSE26939_GSM663288 | C |
| TCGA_TCGA-55-A48X | B | TCGA_TCGA-L9-A743 | B | TCGA_TCGA-49-AAR9 | C | GSE26939_GSM663289 | B |
| TCGA_TCGA-86-8278 | C | TCGA_TCGA-50-5939 | C | TCGA_TCGA-86-8671 | B | GSE26939_GSM663290 | C |
| TCGA_TCGA-55-6982 | B | TCGA_TCGA-78-7161 | A | TCGA_TCGA-49-4507 | C | GSE26939_GSM663291 | B |
| TCGA_TCGA-50-5045 | B | TCGA_TCGA-44-3398 | B | TCGA_TCGA-62-A46V | B | GSE26939_GSM663292 | B |
| TCGA_TCGA-05-4398 | B | TCGA_TCGA-97-8547 | B | TCGA_TCGA-05-4389 | B | GSE26939_GSM663293 | B |
| TCGA_TCGA-55-8513 | B | TCGA_TCGA-86-8280 | B | TCGA_TCGA-50-6592 | C | GSE26939_GSM663294 | A |
| TCGA_TCGA-MP-A4TC | C | TCGA_TCGA-78-7166 | C | TCGA_TCGA-91-6831 | A | GSE26939_GSM663295 | A |
| TCGA_TCGA-05-4420 | A | TCGA_TCGA-91-8499 | C | TCGA_TCGA-55-6987 | B | GSE26939_GSM663296 | A |
| TCGA_TCGA-75-5146 | A | TCGA_TCGA-71-6725 | B | TCGA_TCGA-49-4510 | A | GSE26939_GSM663297 | C |
| TCGA_TCGA-78-7167 | A | TCGA_TCGA-69-7980 | B | TCGA_TCGA-86-8672 | C | GSE26939_GSM663298 | B |
| TCGA_TCGA-J2-8194 | A | TCGA_TCGA-L9-A8F4 | C | TCGA_TCGA-44-2668 | B | GSE26939_GSM663299 | B |
| TCGA_TCGA-97-A4M0 | B | TCGA_TCGA-05-4433 | B | TCGA_TCGA-93-A4JQ | B | GSE26939_GSM663300 | A |
| TCGA_TCGA-38-4632 | B | TCGA_TCGA-55-A492 | A | TCGA_TCGA-97-8177 | B | GSE26939_GSM663301 | B |
| TCGA_TCGA-44-7661 | B | TCGA_TCGA-73-4675 | A | TCGA_TCGA-05-4396 | C | GSE26939_GSM663302 | B |
| TCGA_TCGA-55-8205 | B | TCGA_TCGA-50-5936 | C | TCGA_TCGA-55-6978 | B | GSE26939_GSM663303 | B |
| TCGA_TCGA-55-A48Z | A | TCGA_TCGA-44-7662 | C | TCGA_TCGA-69-8453 | B | GSE26939_GSM663304 | C |
| TCGA_TCGA-44-5645 | B | TCGA_TCGA-78-7154 | C | TCGA_TCGA-50-7109 | A | GSE26939_GSM663305 | A |
| TCGA_TCGA-05-5428 | C | TCGA_TCGA-05-4244 | B | TCGA_TCGA-49-4501 | B | GSE26939_GSM663306 | C |
| TCGA_TCGA-69-8255 | C | TCGA_TCGA-73-7498 | A | TCGA_TCGA-50-5049 | B | GSE26939_GSM663307 | B |
| TCGA_TCGA-75-5125 | B | TCGA_TCGA-50-5933 | B | TCGA_TCGA-78-8660 | B | GSE26939_GSM663308 | B |
| TCGA_TCGA-55-8097 | B | TCGA_TCGA-55-8507 | A | TCGA_TCGA-91-6828 | B | GSE26939_GSM663309 | C |
| TCGA_TCGA-78-7152 | A | TCGA_TCGA-S2-AA1A | B | TCGA_TCGA-50-6590 | C | GSE26939_GSM663310 | C |
| TCGA_TCGA-MP-A4TF | C | TCGA_TCGA-95-7948 | C | TCGA_TCGA-97-8176 | C | GSE26939_GSM663311 | C |
| TCGA_TCGA-67-4679 | B | TCGA_TCGA-67-3774 | A | TCGA_TCGA-50-5935 | B | GSE26939_GSM663312 | B |
| TCGA_TCGA-91-6836 | C | TCGA_TCGA-38-4628 | B | TCGA_TCGA-55-7281 | B | GSE26939_GSM663313 | B |
| TCGA_TCGA-78-8648 | B | TCGA_TCGA-50-6595 | C | TCGA_TCGA-49-4488 | B | GSE26939_GSM663314 | A |
| TCGA_TCGA-44-A47A | B | TCGA_TCGA-55-7903 | B | TCGA_TCGA-NJ-A55A | B | GSE26939_GSM663315 | A |
| TCGA_TCGA-55-A57B | B | TCGA_TCGA-44-3396 | B | TCGA_TCGA-95-8494 | B | GSE26939_GSM663316 | B |
| TCGA_TCGA-55-8207 | B | TCGA_TCGA-80-5611 | C | TCGA_TCGA-44-2657 | B | GSE26939_GSM663317 | B |
| TCGA_TCGA-55-7576 | B | TCGA_TCGA-53-7624 | C | TCGA_TCGA-78-7158 | B | GSE26939_GSM663318 | C |
| TCGA_TCGA-NJ-A55O | B | TCGA_TCGA-91-6835 | B | TCGA_TCGA-93-7347 | B | GSE26939_GSM663319 | A |
| TCGA_TCGA-55-A494 | A | TCGA_TCGA-L9-A50W | B | TCGA_TCGA-38-4630 | C | GSE26939_GSM663320 | B |
| TCGA_TCGA-95-7043 | C | TCGA_TCGA-86-7713 | A | TCGA_TCGA-86-7953 | A | GSE26939_GSM663321 | C |
| TCGA_TCGA-L9-A443 | A | TCGA_TCGA-50-5044 | C | TCGA_TCGA-50-5072 | C | GSE26939_GSM663322 | B |
| TCGA_TCGA-69-7974 | B | TCGA_TCGA-97-7941 | B | TCGA_TCGA-62-A46S | A | GSE26939_GSM663323 | B |
| TCGA_TCGA-NJ-A4YG | B | TCGA_TCGA-86-7714 | B | TCGA_TCGA-53-A4EZ | C | GSE26939_GSM663324 | A |
| TCGA_TCGA-69-7760 | C | TCGA_TCGA-62-8402 | A | TCGA_TCGA-69-7973 | A | GSE26939_GSM663325 | B |
| TCGA_TCGA-49-4486 | A | TCGA_TCGA-78-7162 | A | TCGA_TCGA-55-8204 | C | GSE26939_GSM663326 | B |
| TCGA_TCGA-93-A4JN | A | TCGA_TCGA-49-AAR0 | A | TCGA_TCGA-35-5375 | C | GSE26939_GSM663327 | C |
| TCGA_TCGA-86-8073 | B | TCGA_TCGA-35-4122 | B | TCGA_TCGA-78-7539 | B | GSE26939_GSM663328 | C |
| TCGA_TCGA-L9-A7SV | B | TCGA_TCGA-55-7726 | C | TCGA_TCGA-95-A4VK | C | GSE26939_GSM663329 | A |
| TCGA_TCGA-93-A4JP | B | TCGA_TCGA-62-A46Y | B | TCGA_TCGA-50-5055 | B | GSE26939_GSM663330 | A |
| TCGA_TCGA-78-7163 | A | TCGA_TCGA-55-6980 | B | TCGA_TCGA-44-7667 | A | GSE26939_GSM663331 | C |
| TCGA_TCGA-64-1678 | A | TCGA_TCGA-05-4430 | A | TCGA_TCGA-MP-A4TI | B | GSE26939_GSM663332 | A |
| TCGA_TCGA-NJ-A55R | A | TCGA_TCGA-67-3772 | B | TCGA_TCGA-55-6979 | B | GSE26939_GSM663333 | B |
| TCGA_TCGA-97-A4M7 | B | TCGA_TCGA-MP-A4SY | C | TCGA_TCGA-55-7284 | B | GSE26939_GSM663334 | B |
| TCGA_TCGA-38-A44F | B | TCGA_TCGA-78-7148 | C | TCGA_TCGA-44-2656 | B | GSE26939_GSM663335 | C |
| TCGA_TCGA-62-8399 | A | TCGA_TCGA-35-3615 | A | TCGA_TCGA-44-6778 | B | GSE26939_GSM663336 | B |
| TCGA_TCGA-49-4514 | C | TCGA_TCGA-55-A493 | B | TCGA_TCGA-35-4123 | B | GSE26939_GSM663337 | B |
| TCGA_TCGA-44-5643 | C | TCGA_TCGA-49-6744 | B | TCGA_TCGA-44-6776 | A | GSE26939_GSM663338 | A |
| TCGA_TCGA-44-6147 | B | TCGA_TCGA-49-4494 | C | TCGA_TCGA-73-4676 | B | GSE26939_GSM663339 | B |
| TCGA_TCGA-J2-A4AE | B | TCGA_TCGA-78-7143 | B | TCGA_TCGA-86-8673 | C | GSE26939_GSM663340 | B |
| TCGA_TCGA-05-4415 | C | TCGA_TCGA-50-5051 | A | TCGA_TCGA-62-8395 | A | GSE26939_GSM663341 | C |
| TCGA_TCGA-91-6830 | C | TCGA_TCGA-86-6851 | B | TCGA_TCGA-44-A4SS | C | GSE26939_GSM663342 | B |
| TCGA_TCGA-49-6745 | B | TCGA_TCGA-75-6205 | B | TCGA_TCGA-86-8358 | C | GSE26939_GSM663343 | C |
| TCGA_TCGA-86-A4P7 | B | TCGA_TCGA-49-6743 | C | TCGA_TCGA-55-6968 | C | GSE26939_GSM663344 | C |
| TCGA_TCGA-44-4112 | B | TCGA_TCGA-50-6597 | A | TCGA_TCGA-64-5775 | C | GSE26939_GSM663345 | C |
| TCGA_TCGA-55-6975 | C | TCGA_TCGA-55-A490 | B | TCGA_TCGA-97-7547 | B | GSE26939_GSM663346 | C |
| TCGA_TCGA-95-7039 | C | TCGA_TCGA-99-8032 | A | TCGA_TCGA-75-7031 | A | GSE26939_GSM663347 | C |
| TCGA_TCGA-05-4432 | A | TCGA_TCGA-55-8299 | B | TCGA_TCGA-44-2655 | A | GSE26939_GSM663348 | B |
| TCGA_TCGA-49-4487 | A | TCGA_TCGA-64-1676 | B | TCGA_TCGA-75-6212 | B | GSE26939_GSM663349 | A |
| TCGA_TCGA-55-7727 | B | TCGA_TCGA-86-8279 | C | TCGA_TCGA-05-4390 | A | GSE26939_GSM663350 | C |
| TCGA_TCGA-73-A9RS | C | TCGA_TCGA-97-A4M5 | A | TCGA_TCGA-86-8674 | A | GSE26939_GSM663351 | B |
| TCGA_TCGA-44-5644 | C | TCGA_TCGA-78-7536 | A | TCGA_TCGA-49-6742 | C | GSE26939_GSM663352 | B |
| TCGA_TCGA-55-7914 | B | TCGA_TCGA-55-8208 | B | TCGA_TCGA-44-A47B | A | GSE26939_GSM663353 | B |
| TCGA_TCGA-62-A46R | B | TCGA_TCGA-95-7562 | A | TCGA_TCGA-MP-A4SW | B | GSE26939_GSM663354 | C |
| TCGA_TCGA-49-AARQ | C | TCGA_TCGA-MP-A4TK | A | TCGA_TCGA-64-5815 | C | GSE26939_GSM663355 | B |
| TCGA_TCGA-75-6207 | B | TCGA_TCGA-49-6761 | C | TCGA_TCGA-MP-A4T8 | C | GSE26939_GSM663356 | B |
| TCGA_TCGA-53-7813 | A | TCGA_TCGA-50-6593 | B | TCGA_TCGA-NJ-A4YI | B | GSE26939_GSM663357 | B |
| TCGA_TCGA-44-3918 | B | TCGA_TCGA-55-7728 | B | TCGA_TCGA-L4-A4E6 | B | GSE26939_GSM663358 | B |
| TCGA_TCGA-64-1681 | B | TCGA_TCGA-86-8055 | C | TCGA_TCGA-55-8302 | C | GSE26939_GSM663359 | A |
| TCGA_TCGA-05-4410 | B | TCGA_TCGA-73-4668 | A | TCGA_TCGA-05-4405 | B | GSE26939_GSM663360 | A |
| TCGA_TCGA-55-8620 | B | TCGA_TCGA-55-8090 | A | TCGA_TCGA-78-7147 | B | GSE26939_GSM663361 | B |
| TCGA_TCGA-64-5774 | A | TCGA_TCGA-38-7271 | B | TCGA_TCGA-MP-A4TD | A | GSE26939_GSM663362 | A |
| TCGA_TCGA-86-A4D0 | C | TCGA_TCGA-55-8514 | A | TCGA_TCGA-55-6971 | A | GSE26939_GSM663363 | B |
| TCGA_TCGA-L9-A444 | B | TCGA_TCGA-50-5932 | A | TCGA_TCGA-50-6591 | A | GSE26939_GSM663364 | A |
| GSE26939_GSM663389 | B | GSE26939_GSM663373 | C | TCGA_TCGA-95-7944 | C | GSE26939_GSM663365 | B |
| GSE26939_GSM663390 | C | GSE26939_GSM663374 | A | TCGA_TCGA-NJ-A4YF | A | GSE26939_GSM663366 | B |
| GSE26939_GSM663391 | A | GSE26939_GSM663375 | B | GSE26939_GSM663383 | B | GSE26939_GSM663367 | C |
| GSE26939_GSM663392 | A | GSE26939_GSM663376 | B | GSE26939_GSM663384 | B | GSE26939_GSM663368 | C |
| GSE26939_GSM663393 | B | GSE26939_GSM663377 | A | GSE26939_GSM663385 | C | GSE26939_GSM663369 | C |
| GSE26939_GSM663394 | B | GSE26939_GSM663378 | B | GSE26939_GSM663386 | B | GSE26939_GSM663370 | B |
| GSE26939_GSM663395 | B | GSE26939_GSM663379 | B | GSE26939_GSM663387 | B | GSE26939_GSM663371 | B |
| GSE26939_GSM663396 | C | GSE26939_GSM663380 | A | GSE26939_GSM663388 | B | GSE26939_GSM663372 | B |
| GSE26939_GSM663397 | B | GSE26939_GSM663381 | A | GSE26939_GSM663399 | B | GSE26939_GSM663382 | B |
| GSE26939_GSM663398 | A |  |  |  |  |  |  |
