# Supplementary material for: Correlation analysis of m6A-modified regulators with immune microenvironment infiltrating cells in lung adenocarcinoma
Source: PLoS One. 2022 Feb 23;17(2):e0264384. doi: 10.1371/journal.pone.0264384 (PMC8865675; doi:10.1371/journal.pone.0264384)
Supplement: S5 Table — (DOCX) [file pone.0264384.s007.docx]

**S5 Table The changes of m6Ascore**

| **ID** | **m6Ascore** | **group** | **ID** | **m6Ascore** | **group** |
| --- | --- | --- | --- | --- | --- |
| TCGA-91-6840 | -0.291060027 | High | TCGA-75-6206 | -2.458863007 | Low |
| TCGA-55-6986 | -2.414928596 | Low | TCGA-62-A472 | -3.1408531 | Low |
| TCGA-05-4395 | 8.898102884 | High | TCGA-99-AA5R | -5.80211511 | Low |
| TCGA-44-7672 | -0.165672922 | High | TCGA-55-8085 | 0.006212757 | High |
| TCGA-44-2662 | -3.732227975 | Low | TCGA-44-8117 | 0.668303093 | High |
| TCGA-97-8175 | -2.122343693 | Low | TCGA-55-8506 | -0.321344738 | High |
| TCGA-55-8087 | -5.267358297 | Low | TCGA-NJ-A7XG | 1.165194615 | High |
| TCGA-78-7160 | -2.277372912 | Low | TCGA-05-4384 | -4.731814184 | Low |
| TCGA-L4-A4E5 | 2.84597065 | High | TCGA-64-1679 | 4.478353377 | High |
| TCGA-97-A4LX | -6.367636959 | Low | TCGA-73-4666 | -2.709281886 | Low |
| TCGA-86-8359 | 4.703320399 | High | TCGA-55-8616 | 0.999818078 | High |
| TCGA-44-6145 | 1.461581588 | High | TCGA-38-6178 | 3.146709907 | High |
| TCGA-78-7155 | 2.119229956 | High | TCGA-93-7348 | 2.111109249 | High |
| TCGA-55-7816 | -4.817908788 | Low | TCGA-55-1594 | 0.41983142 | High |
| TCGA-99-8025 | 3.851927589 | High | TCGA-73-4658 | -3.223850694 | Low |
| TCGA-44-A47G | -2.634342262 | Low | TCGA-55-8505 | 9.042068752 | High |
| TCGA-55-8615 | 6.386933115 | High | TCGA-MP-A4T4 | -1.425997914 | Low |
| TCGA-73-7499 | 0.465449266 | High | TCGA-49-4506 | 9.865858713 | High |
| TCGA-86-7711 | 5.615945522 | High | TCGA-97-8172 | -6.171801763 | Low |
| TCGA-86-8075 | 3.318038054 | High | TCGA-O1-A52J | -6.373717633 | Low |
| TCGA-86-7955 | 8.175694588 | High | TCGA-78-7537 | 1.330884977 | High |
| TCGA-55-8508 | 4.134383285 | High | TCGA-73-4662 | -6.173276516 | Low |
| TCGA-67-3771 | -0.113875483 | High | TCGA-62-8398 | 2.449834875 | High |
| TCGA-55-A4DG | -4.782495111 | Low | TCGA-49-AAQV | 2.323739728 | High |
| TCGA-91-7771 | -3.547497644 | Low | TCGA-55-8621 | -5.434171431 | Low |
| TCGA-91-6849 | -1.753715347 | Low | TCGA-53-7626 | -7.075476331 | Low |
| TCGA-64-5781 | 10.15820408 | High | TCGA-44-7669 | -1.539840163 | Low |
| TCGA-44-6146 | -1.123310509 | Low | TCGA-93-8067 | 4.901316114 | High |
| TCGA-97-7552 | -6.436045404 | Low | TCGA-97-A4M2 | -8.209539416 | Low |
| TCGA-80-5608 | 4.152981365 | High | TCGA-05-4425 | -3.107869928 | Low |
| TCGA-91-6829 | 2.136320807 | High | TCGA-69-7763 | 0.517040167 | High |
| TCGA-49-AARE | 8.484983504 | High | TCGA-86-8056 | -8.42926568 | Low |
| TCGA-50-5946 | 5.033909223 | High | TCGA-50-5931 | 9.597977306 | High |
| TCGA-99-7458 | -6.122191545 | Low | TCGA-55-8512 | -0.908423492 | Low |
| TCGA-05-4424 | -3.400816448 | Low | TCGA-75-7025 | -3.923246528 | Low |
| TCGA-44-2666 | -5.714224805 | Low | TCGA-50-5930 | 5.769347798 | High |
| TCGA-44-6775 | -4.548427256 | Low | TCGA-75-6214 | 10.61637243 | High |
| TCGA-38-4631 | 9.477421682 | High | TCGA-L9-A743 | -0.858197397 | Low |
| TCGA-55-7283 | -3.385669006 | Low | TCGA-50-5939 | 3.270518395 | High |
| TCGA-95-7567 | 1.393370229 | High | TCGA-78-7161 | 4.515475246 | High |
| TCGA-38-4629 | 2.397706386 | High | TCGA-44-3398 | -0.817463373 | Low |
| TCGA-91-8497 | -6.75047726 | Low | TCGA-97-8547 | 1.311846374 | High |
| TCGA-78-7540 | 1.233859977 | High | TCGA-86-8280 | -3.543219378 | Low |
| TCGA-55-A48Y | 5.289505646 | High | TCGA-78-7166 | 8.973877618 | High |
| TCGA-55-7995 | -1.696612066 | Low | TCGA-91-8499 | 1.601823409 | High |
| TCGA-44-3919 | -3.640197819 | Low | TCGA-71-6725 | -0.931273709 | Low |
| TCGA-69-7764 | -1.122993477 | Low | TCGA-69-7980 | -0.543120461 | High |
| TCGA-95-7947 | -0.719324985 | High | TCGA-L9-A8F4 | 1.690038084 | High |
| TCGA-MP-A4TH | -5.498210414 | Low | TCGA-05-4433 | -5.45712278 | Low |
| TCGA-55-7725 | -9.188457031 | Low | TCGA-55-A492 | 1.824859452 | High |
| TCGA-L9-A5IP | 10.54558609 | High | TCGA-73-4675 | 2.916530368 | High |
| TCGA-97-7554 | -1.471204752 | Low | TCGA-50-5936 | 5.361904647 | High |
| TCGA-55-8619 | -6.130119264 | Low | TCGA-44-7662 | 5.896673171 | High |
| TCGA-55-7227 | -4.129995495 | Low | TCGA-78-7154 | 4.044033521 | High |
| TCGA-67-3770 | -3.007751258 | Low | TCGA-05-4244 | -5.01400799 | Low |
| TCGA-78-7145 | -1.003507715 | Low | TCGA-73-7498 | -3.799801752 | Low |
| TCGA-49-AAR3 | 1.396979891 | High | TCGA-50-5933 | -1.197326906 | Low |
| TCGA-44-A479 | -3.55743968 | Low | TCGA-55-8507 | 0.312362636 | High |
| TCGA-4B-A93V | 6.982910104 | High | TCGA-S2-AA1A | -0.789980877 | Low |
| TCGA-78-7633 | -0.693364488 | High | TCGA-95-7948 | 2.701951681 | High |
| TCGA-NJ-A4YP | 5.846045038 | High | TCGA-67-3774 | -1.747275143 | Low |
| TCGA-38-4626 | -6.01918961 | Low | TCGA-38-4628 | -0.764653398 | High |
| TCGA-78-7535 | 1.702694813 | High | TCGA-50-6595 | 7.689696714 | High |
| TCGA-55-6970 | -1.707292565 | Low | TCGA-55-7903 | -3.565561152 | Low |
| TCGA-55-6543 | -2.845799352 | Low | TCGA-44-3396 | -1.399588832 | Low |
| TCGA-05-4402 | 0.005170827 | High | TCGA-80-5611 | -0.994048593 | Low |
| TCGA-55-1596 | 5.64050271 | High | TCGA-53-7624 | 10.67519778 | High |
| TCGA-49-4490 | 4.416895187 | High | TCGA-91-6835 | -7.693714982 | Low |
| TCGA-62-A471 | 10.62879632 | High | TCGA-L9-A50W | -1.697723 | Low |
| TCGA-86-A456 | -3.108929154 | Low | TCGA-86-7713 | 0.09409597 | High |
| TCGA-49-4512 | 0.344793352 | High | TCGA-50-5044 | 6.67418128 | High |
| TCGA-55-A48X | -2.494616859 | Low | TCGA-97-7941 | -1.903578088 | Low |
| TCGA-86-8278 | 1.551762311 | High | TCGA-86-7714 | -8.099635141 | Low |
| TCGA-55-6982 | 1.704070599 | High | TCGA-62-8402 | 0.737882707 | High |
| TCGA-50-5045 | -0.92013269 | Low | TCGA-78-7162 | -3.142762537 | Low |
| TCGA-05-4398 | -0.02666148 | High | TCGA-49-AAR0 | 0.277387562 | High |
| TCGA-55-8513 | -7.022642113 | Low | TCGA-35-4122 | -0.722055532 | High |
| TCGA-MP-A4TC | 5.133056389 | High | TCGA-55-7726 | 7.425949676 | High |
| TCGA-05-4420 | 5.407472723 | High | TCGA-62-A46Y | -5.99604958 | Low |
| TCGA-75-5146 | -3.354029153 | Low | TCGA-55-6980 | -1.036029684 | Low |
| TCGA-78-7167 | 0.715448418 | High | TCGA-05-4430 | 0.621374533 | High |
| TCGA-J2-8194 | -1.03311928 | Low | TCGA-67-3772 | 0.727377697 | High |
| TCGA-97-A4M0 | -7.454554364 | Low | TCGA-MP-A4SY | 2.704307587 | High |
| TCGA-38-4632 | -0.021731917 | High | TCGA-78-7148 | 2.81185747 | High |
| TCGA-44-7661 | -1.578256082 | Low | TCGA-35-3615 | -1.547801923 | Low |
| TCGA-55-8205 | -6.805648397 | Low | TCGA-55-A493 | 1.646752067 | High |
| TCGA-55-A48Z | 2.907647743 | High | TCGA-49-6744 | -1.98067933 | Low |
| TCGA-44-5645 | -10.67048408 | Low | TCGA-49-4494 | 3.668803558 | High |
| TCGA-05-5428 | 1.089824752 | High | TCGA-78-7143 | -4.125904392 | Low |
| TCGA-69-8255 | 0.427304674 | High | TCGA-50-5051 | 4.253061574 | High |
| TCGA-75-5125 | -3.758155895 | Low | TCGA-86-6851 | -5.030925949 | Low |
| TCGA-55-8097 | -4.094024014 | Low | TCGA-49-6743 | 4.657893374 | High |
| TCGA-78-7152 | -0.537343254 | High | TCGA-50-6597 | 0.338818144 | High |
| TCGA-MP-A4TF | 6.170161737 | High | TCGA-55-A490 | 1.304063834 | High |
| TCGA-67-4679 | -8.512997194 | Low | TCGA-99-8032 | 4.740979657 | High |
| TCGA-91-6836 | 3.609832761 | High | TCGA-55-8299 | -0.941994471 | Low |
| TCGA-78-8648 | -0.756209926 | High | TCGA-64-1676 | 0.018562708 | High |
| TCGA-44-A47A | -6.266813332 | Low | TCGA-86-8279 | 3.392828121 | High |
| TCGA-55-A57B | -5.047915561 | Low | TCGA-97-A4M5 | -2.561852006 | Low |
| TCGA-55-8207 | -3.538117109 | Low | TCGA-78-7536 | 0.730680523 | High |
| TCGA-55-7576 | -2.067037782 | Low | TCGA-55-8208 | -2.400023642 | Low |
| TCGA-NJ-A55O | -2.884069011 | Low | TCGA-95-7562 | 2.841581303 | High |
| TCGA-55-A494 | 6.162332199 | High | TCGA-MP-A4TK | 2.181087858 | High |
| TCGA-95-7043 | 4.056771513 | High | TCGA-49-6761 | 3.431671451 | High |
| TCGA-L9-A443 | 1.04012769 | High | TCGA-50-6593 | -0.524507617 | High |
| TCGA-69-7974 | -5.025197982 | Low | TCGA-55-7728 | -5.33994415 | Low |
| TCGA-NJ-A4YG | -2.164224364 | Low | TCGA-86-8055 | 3.263982073 | High |
| TCGA-69-7760 | 6.430481292 | High | TCGA-73-4668 | 6.122584601 | High |
| TCGA-49-4486 | 1.696707336 | High | TCGA-55-8090 | 0.325633792 | High |
| TCGA-93-A4JN | 1.183526182 | High | TCGA-38-7271 | -5.31988341 | Low |
| TCGA-86-8073 | -2.454894664 | Low | TCGA-55-8514 | -2.524612874 | Low |
| TCGA-L9-A7SV | -3.978199189 | Low | TCGA-50-5932 | -0.451398877 | High |
| TCGA-93-A4JP | -5.904263654 | Low | TCGA-95-A4VN | -1.916303895 | Low |
| TCGA-78-7163 | 0.43194649 | High | TCGA-86-7954 | -4.091564606 | Low |
| TCGA-64-1678 | 8.842632657 | High | TCGA-91-A4BC | 2.340308885 | High |
| TCGA-NJ-A55R | 4.961702392 | High | TCGA-MP-A4TJ | -3.006296192 | Low |
| TCGA-97-A4M7 | -4.987411118 | Low | TCGA-62-A470 | -2.311031166 | Low |
| TCGA-38-A44F | -2.859711838 | Low | TCGA-73-4677 | 0.171426674 | High |
| TCGA-62-8399 | 1.718623878 | High | TCGA-55-7911 | -5.590364019 | Low |
| TCGA-49-4514 | 6.426479508 | High | TCGA-50-8460 | -5.793431511 | Low |
| TCGA-44-5643 | 5.119107337 | High | TCGA-55-6981 | -0.147373962 | High |
| TCGA-44-6147 | -3.745866821 | Low | TCGA-44-8120 | 0.557058311 | High |
| TCGA-J2-A4AE | -5.142173711 | Low | TCGA-55-8614 | 6.184600806 | High |
| TCGA-05-4415 | 10.2232625 | High | TCGA-64-1677 | 2.311468512 | High |
| TCGA-91-6830 | -0.375485077 | High | TCGA-97-8552 | -5.767407237 | Low |
| TCGA-49-6745 | -3.024566912 | Low | TCGA-69-8253 | 2.252327033 | High |
| TCGA-86-A4P7 | -5.186075793 | Low | TCGA-86-8668 | -5.172578234 | Low |
| TCGA-44-4112 | -2.430679436 | Low | TCGA-86-8074 | 2.174232174 | High |
| TCGA-55-6975 | 7.92949107 | High | TCGA-69-7765 | 0.613221605 | High |
| TCGA-95-7039 | 3.060709356 | High | TCGA-55-8092 | 3.720734983 | High |
| TCGA-05-4432 | 2.089720872 | High | TCGA-NJ-A4YQ | 0.325660695 | High |
| TCGA-49-4487 | 2.034044563 | High | TCGA-55-6983 | 0.792065897 | High |
| TCGA-55-7727 | -5.949995057 | Low | TCGA-62-8394 | 2.977406269 | High |
| TCGA-73-A9RS | 5.661020699 | High | TCGA-44-2661 | -7.869634907 | Low |
| TCGA-44-5644 | 7.662608551 | High | TCGA-44-7659 | -5.355494857 | Low |
| TCGA-55-7914 | -2.532724212 | Low | TCGA-55-6712 | -0.321708575 | High |
| TCGA-62-A46R | -5.423554205 | Low | TCGA-05-4403 | -0.866768307 | Low |
| TCGA-49-AARQ | 1.675986509 | High | TCGA-97-7553 | -8.779023606 | Low |
| TCGA-53-7813 | 2.138785559 | High | TCGA-78-8655 | -3.181989789 | Low |
| TCGA-44-3918 | -5.956773299 | Low | TCGA-55-7724 | -2.807317299 | Low |
| TCGA-64-1681 | -1.542847676 | Low | TCGA-73-4659 | -0.43656014 | High |
| TCGA-05-4410 | -3.368303077 | Low | TCGA-50-6673 | -0.540923049 | High |
| TCGA-55-8620 | 0.792411259 | High | TCGA-78-7542 | 5.673344065 | High |
| TCGA-64-5774 | 6.231125812 | High | TCGA-38-4625 | 0.892316008 | High |
| TCGA-86-A4D0 | 3.556905733 | High | TCGA-55-7570 | 9.250924688 | High |
| TCGA-L9-A444 | -4.698455334 | Low | TCGA-69-7979 | 5.872876194 | High |
| TCGA-MP-A4T7 | 0.766409664 | High | TCGA-55-A4DF | -1.014561942 | Low |
| TCGA-91-A4BD | -6.710827371 | Low | TCGA-67-3773 | -6.617813115 | Low |
| TCGA-05-4250 | 2.16762884 | High | TCGA-55-7573 | -5.914765557 | Low |
| TCGA-55-7994 | -5.655177072 | Low | TCGA-50-5068 | -3.788028158 | Low |
| TCGA-99-8028 | -0.707717085 | High | TCGA-49-AARN | 0.643432787 | High |
| TCGA-97-8171 | -2.112610159 | Low | TCGA-78-7150 | 12.61403171 | High |
| TCGA-67-6216 | -4.256775337 | Low | TCGA-MP-A4TA | 5.482056524 | High |
| TCGA-91-6848 | 0.006900488 | High | TCGA-55-7907 | -0.667972539 | High |
| TCGA-44-2665 | 1.806241772 | High | TCGA-55-5899 | 4.655792364 | High |
| TCGA-86-7701 | 0.28790334 | High | TCGA-55-7574 | -2.251053716 | Low |
| TCGA-78-7156 | -0.436148038 | High | TCGA-44-6148 | -5.282910986 | Low |
| TCGA-05-5420 | -4.622047318 | Low | TCGA-MN-A4N1 | 6.411497039 | High |
| TCGA-55-6642 | 2.27793695 | High | TCGA-05-5429 | 5.866346573 | High |
| TCGA-78-7149 | 3.669651753 | High | TCGA-91-6847 | 6.406210182 | High |
| TCGA-05-4418 | 6.143676308 | High | TCGA-55-8511 | 1.638541218 | High |
| TCGA-05-4422 | -3.06798788 | Low | TCGA-50-5944 | -3.203046178 | Low |
| TCGA-78-7146 | 4.689525756 | High | TCGA-55-1592 | -3.269869901 | Low |
| TCGA-86-A4P8 | -10.77394912 | Low | TCGA-50-8457 | -6.549710584 | Low |
| TCGA-67-6217 | -4.750033424 | Low | TCGA-78-7159 | 3.593488311 | High |
| TCGA-78-8640 | 2.642912246 | High | TCGA-67-6215 | -6.090946022 | Low |
| TCGA-44-7670 | 5.681865491 | High | TCGA-MP-A4T9 | -1.180152532 | Low |
| TCGA-49-AARO | 0.426338657 | High | TCGA-97-7938 | -3.700586491 | Low |
| TCGA-50-5941 | -4.239540571 | Low | TCGA-05-5425 | -1.437129264 | Low |
| TCGA-49-AARR | -0.885322396 | Low | TCGA-55-7913 | 5.392154394 | High |
| TCGA-J2-8192 | -1.00167388 | Low | TCGA-05-4417 | -0.28471296 | High |
| TCGA-64-5779 | 3.528930046 | High | TCGA-05-4434 | -0.093754334 | High |
| TCGA-62-A46O | 9.465560427 | High | TCGA-55-8096 | 0.424844595 | High |
| TCGA-50-8459 | 1.228354049 | High | TCGA-49-6767 | 4.334873609 | High |
| TCGA-86-A4JF | -0.935678931 | Low | TCGA-MP-A4T6 | -4.356857183 | Low |
| TCGA-05-4249 | -6.666895543 | Low | TCGA-MP-A4TE | 7.152258963 | High |
| TCGA-J2-A4AG | -2.660116229 | Low | GSM663284 | 3.209931292 | High |
| TCGA-44-7671 | 3.497400004 | High | GSM663285 | -3.690855587 | Low |
| TCGA-44-6774 | 2.815757956 | High | GSM663286 | -1.449245342 | Low |
| TCGA-50-5066 | 1.438326282 | High | GSM663287 | -5.167306628 | Low |
| TCGA-50-6594 | 3.414483812 | High | GSM663288 | 8.083924033 | High |
| TCGA-44-8119 | 5.654976301 | High | GSM663289 | 1.551217346 | High |
| TCGA-49-AAR2 | 4.401912476 | High | GSM663290 | 3.155930216 | High |
| TCGA-44-6779 | 1.431117304 | High | GSM663291 | -2.0759323 | Low |
| TCGA-86-8669 | -1.758074617 | Low | GSM663292 | -2.487774232 | Low |
| TCGA-05-5715 | -0.57217238 | High | GSM663293 | -0.720619041 | High |
| TCGA-69-A59K | -1.543158663 | Low | GSM663294 | 1.36595604 | High |
| TCGA-55-A491 | 1.495466267 | High | GSM663295 | -4.784140127 | Low |
| TCGA-71-8520 | 0.904792238 | High | GSM663296 | 0.801591976 | High |
| TCGA-91-8496 | -8.842315404 | Low | GSM663297 | 4.85625462 | High |
| TCGA-69-8254 | -4.412405566 | Low | GSM663298 | -2.296466048 | Low |
| TCGA-95-A4VP | 2.578960799 | High | GSM663299 | -1.308387178 | Low |
| TCGA-95-8039 | -4.380777273 | Low | GSM663300 | -2.23432632 | Low |
| TCGA-86-8076 | -2.509168944 | Low | GSM663301 | 0.245665596 | High |
| TCGA-44-2659 | -4.367078386 | Low | GSM663302 | -2.565787975 | Low |
| TCGA-55-8203 | 0.559583743 | High | GSM663303 | 0.725615299 | High |
| TCGA-MP-A5C7 | -0.400298481 | High | GSM663304 | 8.034461668 | High |
| TCGA-78-7220 | 6.727832548 | High | GSM663305 | 2.055256463 | High |
| TCGA-44-3917 | -5.306101252 | Low | GSM663306 | 2.452290764 | High |
| TCGA-78-7153 | 3.320863729 | High | GSM663307 | -4.910251014 | Low |
| TCGA-55-7910 | 6.060843234 | High | GSM663308 | -0.698601654 | High |
| TCGA-64-5778 | -1.137121415 | Low | GSM663309 | 6.912908425 | High |
| TCGA-55-7815 | -2.434958659 | Low | GSM663310 | 2.754193819 | High |
| TCGA-55-6985 | 1.265389986 | High | GSM663311 | 2.62366617 | High |
| TCGA-69-7761 | -2.060626889 | Low | GSM663312 | -2.24795347 | Low |
| TCGA-MN-A4N4 | 6.964086918 | High | GSM663313 | -0.212422826 | High |
| TCGA-55-6972 | 1.189711272 | High | GSM663314 | 5.391238671 | High |
| TCGA-97-A4M1 | -7.557445704 | Low | GSM663315 | 5.253685033 | High |
| TCGA-83-5908 | 0.013192981 | High | GSM663316 | -2.326921501 | Low |
| TCGA-97-8179 | -0.467542331 | High | GSM663317 | -0.56366035 | High |
| TCGA-64-1680 | -0.343381511 | High | GSM663318 | 9.82517219 | High |
| TCGA-73-4670 | 10.83705355 | High | GSM663319 | 1.8714199 | High |
| TCGA-97-8174 | -4.027373479 | Low | GSM663320 | -0.038839271 | High |
| TCGA-44-6777 | -5.520092674 | Low | GSM663321 | 0.167371906 | High |
| TCGA-62-A46P | 3.199184876 | High | GSM663322 | -4.777586308 | Low |
| TCGA-55-8089 | -2.197589084 | Low | GSM663323 | -3.0672653 | Low |
| TCGA-MN-A4N5 | 0.330398283 | High | GSM663324 | 0.873833567 | High |
| TCGA-49-AAR9 | 9.396843358 | High | GSM663325 | -3.626832971 | Low |
| TCGA-86-8671 | -6.124229381 | Low | GSM663326 | -3.635608982 | Low |
| TCGA-49-4507 | 9.965846037 | High | GSM663327 | 4.064422529 | High |
| TCGA-62-A46V | -1.856297224 | Low | GSM663328 | 1.243636057 | High |
| TCGA-05-4389 | -3.307288105 | Low | GSM663329 | -0.138848874 | High |
| TCGA-50-6592 | 4.833659307 | High | GSM663330 | 3.235166044 | High |
| TCGA-91-6831 | 2.87125587 | High | GSM663331 | 3.259393275 | High |
| TCGA-55-6987 | -2.406496825 | Low | GSM663332 | -1.880109427 | Low |
| TCGA-49-4510 | 3.254012598 | High | GSM663333 | -0.225357738 | High |
| TCGA-86-8672 | 6.46387939 | High | GSM663334 | -0.070549975 | High |
| TCGA-44-2668 | -2.081133537 | Low | GSM663335 | 8.302801411 | High |
| TCGA-93-A4JQ | 1.4544393 | High | GSM663336 | 0.918377419 | High |
| TCGA-97-8177 | -2.447329825 | Low | GSM663337 | -1.958510231 | Low |
| TCGA-05-4396 | 3.16764629 | High | GSM663338 | -1.782950343 | Low |
| TCGA-55-6978 | -2.161913783 | Low | GSM663339 | 0.032942263 | High |
| TCGA-69-8453 | -5.21364433 | Low | GSM663340 | 0.519421931 | High |
| TCGA-50-7109 | 1.29029395 | High | GSM663341 | 1.091667538 | High |
| TCGA-49-4501 | -1.56523954 | Low | GSM663342 | -4.815570336 | Low |
| TCGA-50-5049 | -3.933663396 | Low | GSM663343 | 8.286623827 | High |
| TCGA-78-8660 | -1.433101762 | Low | GSM663344 | 6.340408031 | High |
| TCGA-91-6828 | -6.75097046 | Low | GSM663345 | 3.705858416 | High |
| TCGA-50-6590 | 2.447358706 | High | GSM663346 | 2.237454193 | High |
| TCGA-97-8176 | 2.320418095 | High | GSM663347 | -0.821724418 | Low |
| TCGA-50-5935 | -3.591429881 | Low | GSM663348 | -0.137127622 | High |
| TCGA-55-7281 | -2.340894081 | Low | GSM663349 | -1.056458581 | Low |
| TCGA-49-4488 | 0.067451026 | High | GSM663350 | 0.687486589 | High |
| TCGA-NJ-A55A | -3.896394664 | Low | GSM663351 | -0.67264494 | High |
| TCGA-95-8494 | -0.912545744 | Low | GSM663352 | -4.931723888 | Low |
| TCGA-44-2657 | -4.8152322 | Low | GSM663353 | 0.060369928 | High |
| TCGA-78-7158 | -4.441163425 | Low | GSM663354 | 3.05497853 | High |
| TCGA-93-7347 | -5.370528669 | Low | GSM663355 | -2.55126624 | Low |
| TCGA-38-4630 | 5.317851698 | High | GSM663356 | -2.732515004 | Low |
| TCGA-86-7953 | -1.907429478 | Low | GSM663357 | -3.116019157 | Low |
| TCGA-50-5072 | 7.729406316 | High | GSM663358 | -3.36968381 | Low |
| TCGA-62-A46S | -1.720261148 | Low | GSM663359 | 0.555497816 | High |
| TCGA-53-A4EZ | 1.804925699 | High | GSM663360 | -0.880053526 | Low |
| TCGA-69-7973 | 3.184464499 | High | GSM663362 | -0.767293696 | Low |
| TCGA-55-8204 | 6.643405658 | High | GSM663363 | -3.115451396 | Low |
| TCGA-35-5375 | 5.821573621 | High | GSM663364 | 5.387318451 | High |
| TCGA-78-7539 | -5.33481646 | Low | GSM663365 | -4.664242563 | Low |
| TCGA-95-A4VK | 2.332503978 | High | GSM663366 | -4.80729484 | Low |
| TCGA-50-5055 | -1.551591787 | Low | GSM663367 | 2.895775034 | High |
| TCGA-44-7667 | 5.66725724 | High | GSM663368 | 1.901883723 | High |
| TCGA-MP-A4TI | -2.131262881 | Low | GSM663369 | 3.801246286 | High |
| TCGA-55-6979 | -2.700039122 | Low | GSM663370 | -7.365779179 | Low |
| TCGA-55-7284 | -3.727145981 | Low | GSM663371 | -7.733091736 | Low |
| TCGA-44-2656 | -8.682977638 | Low | GSM663372 | 1.492880963 | High |
| TCGA-44-6778 | -9.101109821 | Low | GSM663373 | 2.385031516 | High |
| TCGA-35-4123 | 0.560655534 | High | GSM663374 | 2.710807508 | High |
| TCGA-44-6776 | 0.928652786 | High | GSM663375 | -2.252092247 | Low |
| TCGA-73-4676 | 0.449167769 | High | GSM663376 | -5.462938661 | Low |
| TCGA-86-8673 | 7.397377606 | High | GSM663377 | 2.201469964 | High |
| TCGA-62-8395 | -1.874257485 | Low | GSM663378 | -2.905579518 | Low |
| TCGA-44-A4SS | 3.051890253 | High | GSM663379 | -5.585639086 | Low |
| TCGA-86-8358 | 2.67072891 | High | GSM663380 | 1.26393053 | High |
| TCGA-55-6968 | 1.262714571 | High | GSM663381 | 7.525907166 | High |
| TCGA-64-5775 | 5.265131914 | High | GSM663382 | -0.737376067 | High |
| TCGA-97-7547 | -2.453864093 | Low | GSM663383 | -0.856906135 | Low |
| TCGA-44-2655 | -1.099946534 | Low | GSM663384 | -2.269026496 | Low |
| TCGA-75-6212 | -7.18179061 | Low | GSM663385 | 6.737686703 | High |
| TCGA-05-4390 | 3.747779346 | High | GSM663386 | -1.928699965 | Low |
| TCGA-86-8674 | 6.103627268 | High | GSM663387 | -2.982955067 | Low |
| TCGA-49-6742 | 5.862918707 | High | GSM663388 | -5.256486344 | Low |
| TCGA-44-A47B | -1.375522499 | Low | GSM663389 | -4.338061847 | Low |
| TCGA-MP-A4SW | -2.096739075 | Low | GSM663390 | 5.159929579 | High |
| TCGA-64-5815 | 2.508571001 | High | GSM663391 | -2.458353638 | Low |
| TCGA-MP-A4T8 | 8.31011717 | High | GSM663392 | 2.381509449 | High |
| TCGA-NJ-A4YI | -5.817406204 | Low | GSM663393 | -5.571563832 | Low |
| TCGA-L4-A4E6 | -9.400609439 | Low | GSM663394 | -6.010135787 | Low |
| TCGA-55-8302 | 1.796938763 | High | GSM663395 | -4.176495674 | Low |
| TCGA-05-4405 | -1.91485313 | Low | GSM663396 | 8.022318617 | High |
| TCGA-78-7147 | 0.021150653 | High | GSM663397 | -4.457762021 | Low |
| TCGA-MP-A4TD | 2.610990112 | High | GSM663398 | 2.666733241 | High |
| TCGA-55-6971 | -4.060913069 | Low | GSM663399 | -0.21663549 | High |
| TCGA-50-6591 | 8.476398472 | High | TCGA-55-6984 | 3.615061919 | High |
| TCGA-95-7944 | 4.016192693 | High | TCGA-05-4427 | -3.81829155 | Low |
| TCGA-NJ-A4YF | 8.948898492 | High | TCGA-86-8281 | -1.055630386 | Low |
| TCGA-55-8091 | 0.306406456 | High | TCGA-44-A4SU | 0.269064907 | High |
| TCGA-49-4505 | -0.601561066 | High | TCGA-75-5147 | -0.475502422 | High |
| TCGA-62-8397 | -4.213358963 | Low | TCGA-97-7546 | -6.71733447 | Low |
| TCGA-50-5942 | -7.238240713 | Low | TCGA-86-8585 | 3.664112674 | High |
| TCGA-55-8206 | -11.44875146 | Low | TCGA-55-8301 | -1.913954111 | Low |
| TCGA-55-8094 | 7.327430396 | High | TCGA-86-6562 | 1.111288423 | High |
| TCGA-05-4382 | 1.578720498 | High | TCGA-J2-A4AD | 6.172261102 | High |
| TCGA-05-5423 | -6.785092139 | Low | TCGA-55-8510 | -2.915872469 | Low |
| TCGA-49-AAR4 | 2.352524602 | High | TCGA-99-8033 | 5.15129875 | High |
| TCGA-97-A4M3 | 1.496412407 | High | TCGA-69-7978 | -2.911664478 | Low |
| TCGA-05-4397 | 4.728988779 | High | TCGA-86-8054 | 4.32049696 | High |
| TCGA-MP-A4SV | -1.639441642 | Low | TCGA-44-7660 | 5.645601384 | High |
| TCGA-38-4627 | 3.537826747 | High | TCGA-78-8662 | 3.66370654 | High |
| TCGA-93-A4JO | -4.337724625 | Low | TCGA-75-7027 | 7.957222244 | High |
| TCGA-05-4426 | -0.958548137 | Low | TCGA-97-A4M6 | -3.370721954 | Low |
| TCGA-97-7937 | 0.983515069 | High |  |  |  |
