# Supplementary material for: Correlation analysis of m6A-modified regulators with immune microenvironment infiltrating cells in lung adenocarcinoma
Source: PLoS One. 2022 Feb 23;17(2):e0264384. doi: 10.1371/journal.pone.0264384 (PMC8865675; doi:10.1371/journal.pone.0264384)
Supplement: S1 Data — (ZIP) [file pone.0264384.s008.zip › raw data/GSVA enrichment analysis.docx]

**The activation states of biological pathways in distinct m6A clusters by GSVA enrichment analysis**

|  | **Activated.B.cellna** | **Activated.CD4.T.cellna** | **Activated.CD8.T.cellna** | **Activated.dendritic.cellna** | **CD56bright.natural.killer.cellna** | **CD56dim.natural.killer.cellna** | **Eosinophilna** | **Gamma.delta.T.cellna** | **Immature..B.cellna** | **Immature.dendritic.cellna** | **MDSCna** | **Macrophagena** | **Mast.cellna** | **Monocytena** | **Natural.killer.T.cellna** | **Natural.killer.cellna** | **Neutrophilna** | **Plasmacytoid.dendritic.cellna** | **Regulatory.T.cellna** | **T.follicular.helper.cellna** | **Type.1.T.helper.cellna** | **Type.17.T.helper.cellna** | **Type.2.T.helper.cellna** |
| --- | --- | --- | --- | --- | --- | --- | --- | --- | --- | --- | --- | --- | --- | --- | --- | --- | --- | --- | --- | --- | --- | --- | --- |
| TCGA_TCGA-91-6840 | 0.270410481 | 0.47803739 | 0.51867677 | 0.605644171 | 0.557708117 | 0.644001892 | 0.33344904 | 0.544147683 | 0.454417007 | 0.644320851 | 0.675983786 | 0.385782673 | 0.418636243 | 0.731165232 | 0.458279716 | 0.626594082 | 0.071951234 | 0.66031259 | 0.594943243 | 0.5419434 | 0.493471619 | 0.401102176 | 0.473748333 |
| TCGA_TCGA-55-6986 | 0.319256603 | 0.415858586 | 0.49774998 | 0.671552367 | 0.623076398 | 0.646518582 | 0.356367998 | 0.609565736 | 0.444402215 | 0.696031975 | 0.718497766 | 0.420756281 | 0.463218866 | 0.797063 | 0.418939565 | 0.636530572 | 0.169514909 | 0.698515163 | 0.588708746 | 0.530579305 | 0.501960372 | 0.366875086 | 0.54410983 |
| TCGA_TCGA-05-4395 | 0.209242856 | 0.641576156 | 0.547247043 | 0.6807268 | 0.625981676 | 0.66276585 | 0.234701642 | 0.66766047 | 0.43633155 | 0.659125446 | 0.760386344 | 0.421777037 | 0.41635325 | 0.749145437 | 0.546957668 | 0.612316365 | 0.388337892 | 0.654382382 | 0.600240932 | 0.528948976 | 0.531895583 | 0.418455452 | 0.53361863 |
| TCGA_TCGA-44-7672 | 0.572401802 | 0.633092203 | 0.610940794 | 0.702049947 | 0.640622126 | 0.692890428 | 0.287192517 | 0.684394663 | 0.603803863 | 0.663697387 | 0.898503311 | 0.466907213 | 0.525662488 | 0.798604529 | 0.56388159 | 0.721103667 | 0.193329494 | 0.704463023 | 0.778309476 | 0.585612666 | 0.586266348 | 0.397727258 | 0.513443428 |
| TCGA_TCGA-44-2662 | 0.211812804 | 0.586388118 | 0.394399209 | 0.71139174 | 0.596301477 | 0.68727015 | 0.393532127 | 0.662211225 | 0.525493865 | 0.768573829 | 0.831363277 | 0.468157897 | 0.540783625 | 0.749710808 | 0.507378736 | 0.676462857 | 0.292220779 | 0.683943482 | 0.811266358 | 0.586865679 | 0.553761773 | 0.452480608 | 0.569052248 |
| TCGA_TCGA-97-8175 | 0.208639158 | 0.468684659 | 0.457972058 | 0.728368563 | 0.650309934 | 0.681881396 | 0.201122036 | 0.631737633 | 0.453141707 | 0.709569499 | 0.778777676 | 0.397260544 | 0.484293073 | 0.770121754 | 0.468412829 | 0.711483878 | 0.22630787 | 0.664042008 | 0.72809561 | 0.546833743 | 0.511335214 | 0.412304754 | 0.515218969 |
| TCGA_TCGA-55-8087 | 0.167710532 | 0.20394746 | 0.403273898 | 0.667059536 | 0.588433338 | 0.6198076 | 0.316542641 | 0.521214738 | 0.384891511 | 0.659548282 | 0.611169093 | 0.393853873 | 0.441285427 | 0.743520216 | 0.370344684 | 0.665260158 | 0.189069789 | 0.663993023 | 0.516511386 | 0.531045659 | 0.466168292 | 0.415765147 | 0.447285266 |
| TCGA_TCGA-78-7160 | 0.371523843 | 0.60223889 | 0.622401306 | 0.684372244 | 0.593330318 | 0.63381606 | 0.39793584 | 0.632641678 | 0.508891576 | 0.709709395 | 0.809217775 | 0.485216647 | 0.547463824 | 0.773923903 | 0.512285095 | 0.667728191 | 0.31586541 | 0.683122289 | 0.658047947 | 0.588006347 | 0.553968271 | 0.372619211 | 0.64221413 |
| TCGA_TCGA-L4-A4E5 | 0.223785529 | 0.461962829 | 0.400184467 | 0.609698963 | 0.549747268 | 0.713635507 | 0.267669582 | 0.594074411 | 0.394293771 | 0.684936015 | 0.597672229 | 0.345839203 | 0.412174571 | 0.760434566 | 0.390215541 | 0.608392321 | 0.207579368 | 0.643108314 | 0.494796801 | 0.509942327 | 0.465245396 | 0.368014215 | 0.524367747 |
| TCGA_TCGA-97-A4LX | 0.5709013 | 0.568822706 | 0.63864069 | 0.687825556 | 0.617735098 | 0.680485274 | 0.333327607 | 0.680216962 | 0.653137848 | 0.669059173 | 0.892032186 | 0.472643925 | 0.500368242 | 0.799357152 | 0.519009664 | 0.715793048 | 0.202684847 | 0.717390354 | 0.786773144 | 0.63000343 | 0.599094837 | 0.423780488 | 0.42996086 |
| TCGA_TCGA-86-8359 | 0.274191549 | 0.524031263 | 0.665593965 | 0.670382915 | 0.668208459 | 0.697647039 | 0.286269532 | 0.5580796 | 0.376419722 | 0.663885149 | 0.776124526 | 0.374655639 | 0.44266527 | 0.735626853 | 0.413108341 | 0.662493523 | 0.297042732 | 0.631240341 | 0.551947947 | 0.522751943 | 0.526235543 | 0.353301901 | 0.584244567 |
| TCGA_TCGA-44-6145 | 0.540993976 | 0.602869572 | 0.658124602 | 0.738950452 | 0.624762245 | 0.635743127 | 0.260424616 | 0.644688181 | 0.601042731 | 0.691605119 | 0.873950785 | 0.483202559 | 0.51062109 | 0.776947778 | 0.522538926 | 0.711384721 | 0.371640568 | 0.710240843 | 0.664876221 | 0.564331058 | 0.580366609 | 0.412791712 | 0.544142197 |
| TCGA_TCGA-78-7155 | 0.150947401 | 0.579313695 | 0.416721033 | 0.55352935 | 0.651984297 | 0.593611748 | 0.207937667 | 0.553218697 | 0.255929636 | 0.637968493 | 0.491572914 | 0.367175689 | 0.449288665 | 0.664384943 | 0.412053718 | 0.550344846 | 0.031538897 | 0.603501583 | 0.544881398 | 0.484336096 | 0.41591393 | 0.306384866 | 0.579707712 |
| TCGA_TCGA-55-7816 | 0.320491296 | 0.379236962 | 0.483434691 | 0.674181724 | 0.595373464 | 0.544271119 | 0.264375594 | 0.713623661 | 0.545372039 | 0.802493191 | 0.856105282 | 0.504641437 | 0.490598404 | 0.793906482 | 0.544403249 | 0.717875576 | 0.150615602 | 0.756086865 | 0.694388182 | 0.672620845 | 0.575118379 | 0.385068118 | 0.475362373 |
| TCGA_TCGA-99-8025 | 0.277724474 | 0.389074068 | 0.440414933 | 0.62468349 | 0.574154131 | 0.704737555 | 0.300349163 | 0.558400261 | 0.402233402 | 0.721269011 | 0.617146389 | 0.345577376 | 0.381507177 | 0.741858744 | 0.424950327 | 0.637583292 | 0.282567828 | 0.647670173 | 0.493582456 | 0.530340463 | 0.4738347 | 0.42255512 | 0.45241159 |
| TCGA_TCGA-44-A47G | 0.536766327 | 0.616852984 | 0.617520427 | 0.747475283 | 0.63051257 | 0.675439432 | 0.362210984 | 0.61411509 | 0.606752843 | 0.719338456 | 0.904748475 | 0.480812546 | 0.571236547 | 0.787221791 | 0.537102237 | 0.689345696 | 0.235218904 | 0.726930378 | 0.741020231 | 0.597893989 | 0.604296082 | 0.416141751 | 0.517944233 |
| TCGA_TCGA-55-8615 | 0.133230913 | 0.351455757 | 0.382148392 | 0.596051006 | 0.546448779 | 0.740678014 | 0.246390505 | 0.595360682 | 0.30687846 | 0.649225662 | 0.611599325 | 0.334511434 | 0.458721711 | 0.765563068 | 0.411208506 | 0.603625866 | 0.301924943 | 0.674756995 | 0.515296426 | 0.503046212 | 0.463219321 | 0.336613302 | 0.524724552 |
| TCGA_TCGA-73-7499 | 0.383564851 | 0.585227353 | 0.707264399 | 0.700447093 | 0.661416097 | 0.707848084 | 0.191484714 | 0.655632361 | 0.489489601 | 0.666732032 | 0.869669576 | 0.406754024 | 0.450579128 | 0.718198676 | 0.465518945 | 0.693675282 | 0.221813085 | 0.654483516 | 0.661548495 | 0.552867662 | 0.552103915 | 0.389530404 | 0.4516898 |
| TCGA_TCGA-86-7711 | 0.263679603 | 0.618360719 | 0.439226588 | 0.705415095 | 0.628887331 | 0.593801926 | 0.321293901 | 0.672044272 | 0.487074933 | 0.773021931 | 0.781882241 | 0.425343422 | 0.512908673 | 0.738003091 | 0.492688644 | 0.697853329 | 0.352552863 | 0.692616286 | 0.689113897 | 0.535873705 | 0.565582123 | 0.408596192 | 0.506307703 |
| TCGA_TCGA-86-8075 | 0.275297719 | 0.504910826 | 0.404410899 | 0.684180628 | 0.597540329 | 0.649456235 | 0.38888786 | 0.648766871 | 0.501552613 | 0.714195385 | 0.76947065 | 0.404291197 | 0.510367239 | 0.741284784 | 0.515055204 | 0.693868192 | 0.146748263 | 0.714951856 | 0.720658858 | 0.565413113 | 0.564869042 | 0.369102934 | 0.56483442 |
| TCGA_TCGA-86-7955 | 0.151704022 | 0.450509435 | 0.457720604 | 0.533269984 | 0.605739438 | 0.628080209 | 0.25271559 | 0.556888935 | 0.371600576 | 0.624299704 | 0.527627212 | 0.262691907 | 0.384007354 | 0.757243142 | 0.377642089 | 0.500722958 | 0.204372718 | 0.593306429 | 0.399047241 | 0.347698103 | 0.407788111 | 0.384233217 | 0.472936422 |
| TCGA_TCGA-55-8508 | 0.508321631 | 0.428778015 | 0.482346078 | 0.661128161 | 0.559354066 | 0.724997935 | 0.248316817 | 0.59229323 | 0.521867789 | 0.650093497 | 0.776455013 | 0.370783313 | 0.434173204 | 0.761493483 | 0.435342804 | 0.658532442 | 0.258722123 | 0.679257505 | 0.582597949 | 0.504931028 | 0.494304346 | 0.311439479 | 0.459810002 |
| TCGA_TCGA-67-3771 | 0.343498746 | 0.548244723 | 0.503804747 | 0.661441271 | 0.654233319 | 0.691819469 | 0.230252806 | 0.609109968 | 0.516620413 | 0.712889827 | 0.804467451 | 0.392704888 | 0.494372927 | 0.718214578 | 0.426545076 | 0.642909567 | 0.12653772 | 0.708705694 | 0.724272651 | 0.567000007 | 0.531419161 | 0.360416148 | 0.390825418 |
| TCGA_TCGA-55-A4DG | 0.458861171 | 0.442419441 | 0.49369671 | 0.621535004 | 0.560716716 | 0.604782462 | 0.387408706 | 0.573118557 | 0.558398025 | 0.663072017 | 0.728785801 | 0.407468118 | 0.563237416 | 0.725024315 | 0.388302563 | 0.633157677 | 0.13504865 | 0.643832917 | 0.562384857 | 0.539715387 | 0.477298847 | 0.376745472 | 0.533312912 |
| TCGA_TCGA-91-7771 | 0.4036355 | 0.53275008 | 0.604197028 | 0.700361433 | 0.582416327 | 0.660188963 | 0.427026382 | 0.631630142 | 0.559706331 | 0.651962556 | 0.813390151 | 0.457792043 | 0.518927007 | 0.774560509 | 0.507600373 | 0.670865968 | 0.158254717 | 0.697393926 | 0.68222304 | 0.554494672 | 0.553170002 | 0.348170194 | 0.55749346 |
| TCGA_TCGA-91-6849 | 0.467442981 | 0.501346526 | 0.518489389 | 0.681551297 | 0.529404761 | 0.658522384 | 0.497009688 | 0.613119772 | 0.568442969 | 0.648804521 | 0.789754648 | 0.47352598 | 0.51265375 | 0.747729136 | 0.446964403 | 0.642229746 | 0.313411746 | 0.6982877 | 0.644715201 | 0.563113552 | 0.521100105 | 0.425300124 | 0.540421302 |
| TCGA_TCGA-64-5781 | 0.333414068 | 0.610491206 | 0.710209295 | 0.678579579 | 0.649365658 | 0.708287195 | 0.300999981 | 0.642839555 | 0.456966505 | 0.662798117 | 0.807571389 | 0.39966148 | 0.548910259 | 0.737220629 | 0.516372683 | 0.702202831 | 0.280602802 | 0.65928605 | 0.534750995 | 0.522625137 | 0.577516675 | 0.384052853 | 0.55956375 |
| TCGA_TCGA-44-6146 | 0.153293137 | 0.379977368 | 0.355914123 | 0.525862745 | 0.511552924 | 0.636025145 | 0.388400974 | 0.478894394 | 0.393641789 | 0.648772543 | 0.455507015 | 0.338163874 | 0.389579536 | 0.744324404 | 0.388318614 | 0.620688346 | 0.283060115 | 0.644305315 | 0.440726155 | 0.488460092 | 0.476514532 | 0.517914026 | 0.588285524 |
| TCGA_TCGA-97-7552 | 0.650674191 | 0.564247247 | 0.694564713 | 0.705766001 | 0.615092213 | 0.648294139 | 0.356095864 | 0.567722507 | 0.712968741 | 0.696279032 | 0.794208113 | 0.451702542 | 0.539472796 | 0.807165833 | 0.486556984 | 0.718506509 | 0.28944387 | 0.709421502 | 0.685589031 | 0.564903204 | 0.577520967 | 0.484299296 | 0.570886884 |
| TCGA_TCGA-80-5608 | 0.1921013 | 0.482689727 | 0.511063712 | 0.700360132 | 0.559038925 | 0.717166328 | 0.276822488 | 0.566049388 | 0.388954868 | 0.651482777 | 0.669177998 | 0.379739 | 0.428945599 | 0.744246154 | 0.4222609 | 0.599783453 | 0.311695553 | 0.645791905 | 0.542823456 | 0.511636853 | 0.446147977 | 0.372229294 | 0.584240499 |
| TCGA_TCGA-91-6829 | 0.219436826 | 0.396265919 | 0.38349048 | 0.609521307 | 0.635154084 | 0.556915912 | 0.216192757 | 0.611183952 | 0.394268193 | 0.681250856 | 0.653244252 | 0.429474788 | 0.552674788 | 0.74147778 | 0.523081911 | 0.66454291 | 0.057261364 | 0.690611226 | 0.614925887 | 0.567401575 | 0.54196461 | 0.347779878 | 0.531878186 |
| TCGA_TCGA-49-AARE | 0.347596364 | 0.528177873 | 0.515822843 | 0.594490674 | 0.609021353 | 0.733050874 | 0.27783151 | 0.589225274 | 0.403109287 | 0.634716519 | 0.738646636 | 0.364832702 | 0.329092544 | 0.739807478 | 0.444056376 | 0.576057408 | 0.21927526 | 0.666240723 | 0.526196138 | 0.438958681 | 0.503582716 | 0.334537117 | 0.38119447 |
| TCGA_TCGA-50-5946 | 0.188238711 | 0.524858015 | 0.430396923 | 0.582351307 | 0.605788499 | 0.632135703 | 0.224632361 | 0.605443989 | 0.379733321 | 0.603823383 | 0.533930759 | 0.282495934 | 0.576894952 | 0.713937833 | 0.450688899 | 0.647004876 | 0.121837753 | 0.651622433 | 0.50930357 | 0.529433299 | 0.475724177 | 0.393596038 | 0.577841066 |
| TCGA_TCGA-99-7458 | 0.32290185 | 0.521541569 | 0.577693185 | 0.691096286 | 0.621466214 | 0.623623066 | 0.352049082 | 0.62276179 | 0.531710226 | 0.702422273 | 0.802881416 | 0.405376468 | 0.533794588 | 0.795878965 | 0.482703696 | 0.705985911 | 0.176528417 | 0.695559615 | 0.700549928 | 0.579965648 | 0.560264766 | 0.421270743 | 0.515789668 |
| TCGA_TCGA-05-4424 | 0.230504515 | 0.517048765 | 0.477896644 | 0.638931367 | 0.61405896 | 0.651859759 | 0.392880716 | 0.619294851 | 0.460741915 | 0.758968745 | 0.784187331 | 0.458790057 | 0.515926471 | 0.778723389 | 0.465998207 | 0.674758211 | 0.151728132 | 0.681655621 | 0.676577118 | 0.57029729 | 0.531941539 | 0.351638237 | 0.519497746 |
| TCGA_TCGA-44-2666 | 0.230852332 | 0.420063341 | 0.35557229 | 0.545624035 | 0.554448347 | 0.625766031 | 0.363414945 | 0.542202704 | 0.51538539 | 0.647744028 | 0.486953687 | 0.362558181 | 0.565764952 | 0.689556747 | 0.386635335 | 0.59512948 | 0.185053984 | 0.645931315 | 0.540660445 | 0.498028377 | 0.472537403 | 0.276958146 | 0.616570913 |
| TCGA_TCGA-44-6775 | 0.308345905 | 0.524976583 | 0.376678605 | 0.707768307 | 0.57012372 | 0.606433694 | 0.39648339 | 0.645874804 | 0.585169573 | 0.693465321 | 0.765268592 | 0.460869437 | 0.541126266 | 0.740083564 | 0.500283369 | 0.715034943 | 0.192870563 | 0.695245883 | 0.778652075 | 0.570165221 | 0.547344306 | 0.428155174 | 0.619011631 |
| TCGA_TCGA-38-4631 | 0.196915328 | 0.588089031 | 0.513553077 | 0.647993563 | 0.566696933 | 0.751222426 | 0.239022638 | 0.617047837 | 0.436155771 | 0.656220502 | 0.679396856 | 0.371391347 | 0.382061335 | 0.747592036 | 0.430276249 | 0.620674381 | 0.28412648 | 0.668917507 | 0.534900435 | 0.441753756 | 0.491011872 | 0.474710926 | 0.467695665 |
| TCGA_TCGA-55-7283 | 0.202537678 | 0.448022007 | 0.514174017 | 0.657149754 | 0.615297783 | 0.612086899 | 0.361304306 | 0.635908429 | 0.413513911 | 0.674192862 | 0.69363951 | 0.37988885 | 0.590163316 | 0.75053308 | 0.413882808 | 0.668562815 | 0.214714809 | 0.673909599 | 0.614117863 | 0.577674561 | 0.471934356 | 0.380643013 | 0.559674896 |
| TCGA_TCGA-95-7567 | 0.327861444 | 0.561703385 | 0.534003887 | 0.647511246 | 0.610865429 | 0.695894886 | 0.229944885 | 0.624598193 | 0.463180499 | 0.665389024 | 0.727297318 | 0.386389709 | 0.42452114 | 0.720210069 | 0.464329783 | 0.649211121 | 0.239834247 | 0.663145558 | 0.601089883 | 0.541770761 | 0.498713129 | 0.402330374 | 0.505778992 |
| TCGA_TCGA-38-4629 | 0.297894099 | 0.582183718 | 0.613437971 | 0.706942812 | 0.635485749 | 0.687281858 | 0.267421051 | 0.732607695 | 0.520752721 | 0.700000034 | 0.895196886 | 0.532247813 | 0.533473664 | 0.761597243 | 0.577877648 | 0.72245728 | 0.241901926 | 0.713678926 | 0.809432378 | 0.642252518 | 0.590862129 | 0.443649169 | 0.494244534 |
| TCGA_TCGA-91-8497 | 0.617517082 | 0.438685571 | 0.521545357 | 0.665081024 | 0.553434956 | 0.681258554 | 0.41179598 | 0.625334545 | 0.619991512 | 0.705459095 | 0.826175788 | 0.451835447 | 0.568780505 | 0.808895632 | 0.457181607 | 0.665302657 | 0.159852266 | 0.731969108 | 0.684949571 | 0.576500583 | 0.537190244 | 0.385736511 | 0.513781268 |
| TCGA_TCGA-78-7540 | 0.212844652 | 0.330582805 | 0.437949504 | 0.588090952 | 0.621001295 | 0.75622289 | 0.258185459 | 0.600168711 | 0.454105421 | 0.658952286 | 0.599665617 | 0.378425926 | 0.38467028 | 0.772062579 | 0.456533856 | 0.634695115 | 0.274631628 | 0.756143327 | 0.524785708 | 0.530801127 | 0.480217995 | 0.487926346 | 0.534599527 |
| TCGA_TCGA-55-A48Y | 0.321367311 | 0.53178243 | 0.464482689 | 0.601793408 | 0.577996367 | 0.679781449 | 0.233451727 | 0.604081276 | 0.48990994 | 0.648208071 | 0.673044888 | 0.394797884 | 0.477974396 | 0.730120684 | 0.481899322 | 0.615360206 | 0.179175572 | 0.703956019 | 0.547834084 | 0.527321205 | 0.516642452 | 0.348371455 | 0.53317174 |
| TCGA_TCGA-55-7995 | 0.391099398 | 0.58401129 | 0.656758856 | 0.679933272 | 0.606381397 | 0.627432118 | 0.272902972 | 0.591681764 | 0.592806359 | 0.736883932 | 0.893086332 | 0.457522708 | 0.409149267 | 0.742108995 | 0.453238728 | 0.692040474 | 0.076461604 | 0.686533525 | 0.698660178 | 0.569043702 | 0.574111603 | 0.394663859 | 0.457146641 |
| TCGA_TCGA-44-3919 | 0.378461505 | 0.515248546 | 0.554682873 | 0.737054509 | 0.602420991 | 0.67410122 | 0.224097797 | 0.625540821 | 0.527676339 | 0.704714662 | 0.842159967 | 0.414471521 | 0.54226306 | 0.754365202 | 0.485905068 | 0.688306732 | 0.165370626 | 0.689132463 | 0.718065017 | 0.594800602 | 0.538600528 | 0.406330545 | 0.526569612 |
| TCGA_TCGA-69-7764 | 0.3104002 | 0.502017174 | 0.534187858 | 0.604271801 | 0.540888328 | 0.650118105 | 0.368881158 | 0.53713351 | 0.515526502 | 0.617712747 | 0.565048039 | 0.358027191 | 0.43817747 | 0.728665899 | 0.379006859 | 0.590859329 | 0.177287893 | 0.636026278 | 0.452853785 | 0.543799497 | 0.484969861 | 0.35628726 | 0.619385054 |
| TCGA_TCGA-95-7947 | 0.32256129 | 0.542169787 | 0.494951257 | 0.625104198 | 0.595497813 | 0.578459243 | 0.319356321 | 0.590154788 | 0.491644085 | 0.657846026 | 0.680063041 | 0.339365436 | 0.359018033 | 0.749829204 | 0.379008331 | 0.668945116 | 0.194787298 | 0.636975116 | 0.541446895 | 0.486356858 | 0.475083483 | 0.394836884 | 0.506974763 |
| TCGA_TCGA-MP-A4TH | 0.671603585 | 0.485310888 | 0.586764416 | 0.687232965 | 0.530995277 | 0.690983934 | 0.39448804 | 0.58186346 | 0.70850781 | 0.632507082 | 0.783808825 | 0.414217654 | 0.500515116 | 0.785661189 | 0.43357759 | 0.655240199 | 0.196078925 | 0.702234982 | 0.613115953 | 0.575535506 | 0.54720947 | 0.428296315 | 0.498853295 |
| TCGA_TCGA-55-7725 | 0.401882921 | 0.539052729 | 0.570505568 | 0.718093978 | 0.656963901 | 0.62540612 | 0.405770929 | 0.611375938 | 0.57156212 | 0.6818433 | 0.764929191 | 0.434465818 | 0.593055313 | 0.728461894 | 0.497389383 | 0.656038881 | 0.191668217 | 0.717225283 | 0.60829094 | 0.571385742 | 0.546389485 | 0.403799027 | 0.559804305 |
| TCGA_TCGA-L9-A5IP | 0.225977761 | 0.58413558 | 0.476401553 | 0.664627185 | 0.594448523 | 0.65898083 | 0.331542345 | 0.605372833 | 0.384876859 | 0.680642237 | 0.693434406 | 0.402862489 | 0.399929668 | 0.73896755 | 0.492629762 | 0.598603171 | 0.404548172 | 0.673815708 | 0.545128413 | 0.50405513 | 0.524758002 | 0.372617272 | 0.463733488 |
| TCGA_TCGA-97-7554 | 0.37415523 | 0.495462898 | 0.457541978 | 0.648599408 | 0.584378465 | 0.571788434 | 0.342705918 | 0.667402141 | 0.508953231 | 0.646036208 | 0.7208324 | 0.374764676 | 0.474452991 | 0.752352916 | 0.47613415 | 0.667855877 | 0.16013754 | 0.676902008 | 0.674277744 | 0.573657546 | 0.524146252 | 0.417095584 | 0.542030386 |
| TCGA_TCGA-55-8619 | 0.517477197 | 0.491810894 | 0.595879505 | 0.705122821 | 0.606738399 | 0.639833783 | 0.383994098 | 0.642783704 | 0.605986831 | 0.720547617 | 0.894233567 | 0.537704658 | 0.630086683 | 0.819610572 | 0.512876442 | 0.724244779 | 0.248383552 | 0.758491819 | 0.719635163 | 0.623283519 | 0.572458666 | 0.454409608 | 0.498479843 |
| TCGA_TCGA-55-7227 | 0.26526013 | 0.539925571 | 0.544464191 | 0.718105576 | 0.613168515 | 0.636129393 | 0.298056044 | 0.623407442 | 0.513479616 | 0.690961284 | 0.809001737 | 0.464986783 | 0.577422451 | 0.797523734 | 0.487351381 | 0.719175074 | 0.197486849 | 0.699781616 | 0.747135317 | 0.581179283 | 0.533928513 | 0.411815407 | 0.525091698 |
| TCGA_TCGA-67-3770 | 0.214588108 | 0.485537523 | 0.55860933 | 0.701887209 | 0.610667447 | 0.643116134 | 0.325165737 | 0.677290301 | 0.428636165 | 0.675122674 | 0.782635636 | 0.451800769 | 0.539766007 | 0.753422015 | 0.458358095 | 0.681855545 | 0.17184207 | 0.700752427 | 0.590249653 | 0.582890157 | 0.528557674 | 0.437698868 | 0.506728357 |
| TCGA_TCGA-78-7145 | 0.144849267 | 0.490769576 | 0.435204134 | 0.653950955 | 0.66163198 | 0.770896785 | 0.328204417 | 0.644745339 | 0.423584988 | 0.711958986 | 0.668786706 | 0.405732223 | 0.575696305 | 0.7673845 | 0.46285348 | 0.659060806 | 0.227339844 | 0.676443167 | 0.633820706 | 0.549367611 | 0.513170167 | 0.432555036 | 0.561118244 |
| TCGA_TCGA-49-AAR3 | 0.452106994 | 0.623258658 | 0.720958443 | 0.692841787 | 0.613262855 | 0.677245324 | 0.271538979 | 0.672008117 | 0.594926976 | 0.703850496 | 0.921395853 | 0.464526116 | 0.397312404 | 0.767729244 | 0.575270335 | 0.720524158 | 0.225009844 | 0.705354725 | 0.75027445 | 0.575471457 | 0.600222229 | 0.415080338 | 0.485209284 |
| TCGA_TCGA-44-A479 | 0.541492135 | 0.611327663 | 0.681790598 | 0.688105549 | 0.612255847 | 0.721347236 | 0.339365125 | 0.607875002 | 0.618057391 | 0.668069694 | 0.861850545 | 0.45666888 | 0.391012159 | 0.766715411 | 0.461116203 | 0.718979572 | 0.141059974 | 0.696292547 | 0.709365729 | 0.592516317 | 0.550537463 | 0.366413496 | 0.480067095 |
| TCGA_TCGA-4B-A93V | 0.269416999 | 0.564034256 | 0.574485925 | 0.63018751 | 0.569432712 | 0.746063315 | 0.439380733 | 0.538343107 | 0.500616546 | 0.611708182 | 0.679468265 | 0.364673273 | 0.364170108 | 0.758304922 | 0.424606674 | 0.612831653 | 0.25787314 | 0.649990333 | 0.512964976 | 0.478538582 | 0.45358237 | 0.386588946 | 0.560940219 |
| TCGA_TCGA-78-7633 | 0.138709205 | 0.316758297 | 0.413729516 | 0.558041271 | 0.532088969 | 0.605453041 | 0.39922049 | 0.586569606 | 0.356727982 | 0.644289161 | 0.512057036 | 0.388076744 | 0.497919862 | 0.708409346 | 0.390838565 | 0.575075008 | 0.190378486 | 0.681065191 | 0.49118745 | 0.531837477 | 0.455413098 | 0.376172084 | 0.510039316 |
| TCGA_TCGA-NJ-A4YP | 0.246618209 | 0.525945634 | 0.502047182 | 0.649914981 | 0.574344544 | 0.737104352 | 0.199010371 | 0.619135161 | 0.387531859 | 0.666955817 | 0.71443738 | 0.398849134 | 0.468445301 | 0.733035941 | 0.496999259 | 0.672390578 | 0.163394812 | 0.668859561 | 0.543644364 | 0.496508132 | 0.521778828 | 0.377899988 | 0.496688113 |
| TCGA_TCGA-38-4626 | 0.262339511 | 0.469353949 | 0.427692393 | 0.683158474 | 0.602761201 | 0.618251142 | 0.465162287 | 0.731307886 | 0.454986471 | 0.774726178 | 0.892351679 | 0.59589819 | 0.659130148 | 0.793658042 | 0.563864606 | 0.712919175 | 0.310163112 | 0.783495312 | 0.726243458 | 0.633578952 | 0.559589668 | 0.385068397 | 0.557097028 |
| TCGA_TCGA-78-7535 | 0.223840064 | 0.454804616 | 0.45849141 | 0.731469082 | 0.595147994 | 0.688266062 | 0.283289239 | 0.594809077 | 0.396906689 | 0.735113533 | 0.810375737 | 0.447481173 | 0.510411726 | 0.81654236 | 0.474157902 | 0.712725499 | 0.290954624 | 0.681241361 | 0.591400492 | 0.580070198 | 0.495860138 | 0.412118461 | 0.531639367 |
| TCGA_TCGA-55-6970 | 0.360421366 | 0.566538564 | 0.531184167 | 0.674255178 | 0.600560744 | 0.645743137 | 0.385118364 | 0.608753424 | 0.55012365 | 0.671317698 | 0.763268444 | 0.498578118 | 0.501578072 | 0.76847569 | 0.465588742 | 0.605734564 | 0.267995266 | 0.677476095 | 0.607241608 | 0.57545894 | 0.516754277 | 0.338007034 | 0.591021746 |
| TCGA_TCGA-55-6543 | 0.226838237 | 0.374416488 | 0.452076191 | 0.68917534 | 0.599610153 | 0.644580754 | 0.366870279 | 0.682667296 | 0.440274732 | 0.716418746 | 0.815040219 | 0.442278504 | 0.553454346 | 0.7559467 | 0.452138267 | 0.666445065 | 0.267541891 | 0.718059131 | 0.696127972 | 0.529472456 | 0.513151077 | 0.384158282 | 0.504252124 |
| TCGA_TCGA-05-4402 | 0.263729898 | 0.427903988 | 0.442084949 | 0.662923699 | 0.626455278 | 0.665907236 | 0.205758465 | 0.665798952 | 0.495770786 | 0.714884078 | 0.750192819 | 0.387545916 | 0.495440263 | 0.753508301 | 0.483599298 | 0.681728222 | 0.174075608 | 0.676764441 | 0.669024353 | 0.556404414 | 0.504826758 | 0.365167789 | 0.532245005 |
| TCGA_TCGA-55-1596 | 0.228482923 | 0.555599442 | 0.507210296 | 0.607882523 | 0.635757079 | 0.65290139 | 0.25503188 | 0.616477529 | 0.374239646 | 0.651394759 | 0.594828777 | 0.357623243 | 0.398206337 | 0.746204278 | 0.407857558 | 0.574921306 | 0.281563522 | 0.606925865 | 0.573824707 | 0.455581612 | 0.479292028 | 0.42483106 | 0.50463848 |
| TCGA_TCGA-49-4490 | 0.194405055 | 0.376384995 | 0.472442779 | 0.677973636 | 0.625408965 | 0.664550458 | 0.268325876 | 0.572612396 | 0.374976631 | 0.706453134 | 0.730409779 | 0.351046646 | 0.477961148 | 0.774120021 | 0.454118963 | 0.634194435 | 0.174721719 | 0.685819492 | 0.479029445 | 0.546180366 | 0.514118503 | 0.388590518 | 0.422666834 |
| TCGA_TCGA-62-A471 | 0.248790435 | 0.515900876 | 0.515485719 | 0.548728532 | 0.591761862 | 0.702000461 | 0.278054976 | 0.542237229 | 0.27474818 | 0.68414919 | 0.616023174 | 0.371990953 | 0.413681911 | 0.745362099 | 0.429822182 | 0.556661525 | 0.36224494 | 0.649722338 | 0.423934932 | 0.421447421 | 0.466179463 | 0.396707606 | 0.577874859 |
| TCGA_TCGA-86-A456 | 0.221985446 | 0.510208635 | 0.501648313 | 0.670222489 | 0.592595667 | 0.697017985 | 0.490485875 | 0.619919436 | 0.492190956 | 0.770505352 | 0.792788716 | 0.449780238 | 0.550050806 | 0.764956944 | 0.494520052 | 0.702040295 | 0.186089983 | 0.685931886 | 0.678237448 | 0.594794992 | 0.528601659 | 0.424835962 | 0.556685831 |
| TCGA_TCGA-49-4512 | 0.265282612 | 0.368263045 | 0.465366372 | 0.667959821 | 0.609732524 | 0.714046692 | 0.266358972 | 0.610230216 | 0.449132754 | 0.694646764 | 0.778503275 | 0.467034314 | 0.522894722 | 0.777637414 | 0.47128565 | 0.659754881 | 0.200121917 | 0.721550505 | 0.623757155 | 0.563064883 | 0.505680764 | 0.438482867 | 0.423735711 |
| TCGA_TCGA-75-5122 | 0.404910825 | 0.72908181 | 0.79322882 | 0.787944115 | 0.663086578 | 0.743238089 | 0.494754994 | 0.772610904 | 0.620094774 | 0.72362981 | 1 | 0.587170945 | 0.643204348 | 0.808657085 | 0.611581501 | 0.766974138 | 0.240502665 | 0.748294823 | 0.881829853 | 0.677929041 | 0.662886329 | 0.426666155 | 0.560476983 |
| TCGA_TCGA-55-A48X | 0.649872766 | 0.508244844 | 0.564201693 | 0.671912664 | 0.565014211 | 0.625406007 | 0.320595751 | 0.606238769 | 0.679203965 | 0.70064507 | 0.798269753 | 0.379399689 | 0.570446768 | 0.770683376 | 0.419409705 | 0.674224048 | 0.22427548 | 0.674379039 | 0.594082469 | 0.550376917 | 0.553137983 | 0.374204908 | 0.496808563 |
| TCGA_TCGA-86-8278 | 0.236159996 | 0.417952217 | 0.495638408 | 0.68038305 | 0.619148782 | 0.678115781 | 0.318445337 | 0.553751335 | 0.427926868 | 0.665715269 | 0.755436323 | 0.40809847 | 0.412446849 | 0.738734676 | 0.447934284 | 0.686517531 | 0.244857526 | 0.683954266 | 0.60737168 | 0.52290084 | 0.514358947 | 0.413575029 | 0.480842472 |
| TCGA_TCGA-55-6982 | 0.326241253 | 0.509555825 | 0.436476673 | 0.680972803 | 0.664740878 | 0.73609867 | 0.215519499 | 0.622978719 | 0.478223948 | 0.702084949 | 0.783913475 | 0.424664396 | 0.447044212 | 0.74928307 | 0.505212722 | 0.672185811 | 0.211013638 | 0.688883794 | 0.730305468 | 0.572770116 | 0.539552512 | 0.416783936 | 0.494337784 |
| TCGA_TCGA-50-5045 | 0.581162222 | 0.615406439 | 0.675121391 | 0.733993955 | 0.647579987 | 0.706252389 | 0.420251598 | 0.71033961 | 0.633922833 | 0.718506189 | 0.947491037 | 0.492039907 | 0.607524278 | 0.814082758 | 0.53605839 | 0.737426611 | 0.239267297 | 0.723702373 | 0.793017508 | 0.660596245 | 0.619066949 | 0.408895688 | 0.502684626 |
| TCGA_TCGA-05-4398 | 0.336268627 | 0.570537302 | 0.501200245 | 0.700508199 | 0.593745072 | 0.671126335 | 0.273466421 | 0.668635792 | 0.523273068 | 0.696928404 | 0.892394345 | 0.438382033 | 0.491961725 | 0.783641865 | 0.546358324 | 0.672791805 | 0.265209124 | 0.700998933 | 0.759225 | 0.604336943 | 0.563993224 | 0.400867674 | 0.440773537 |
| TCGA_TCGA-55-8513 | 0.428039587 | 0.398240008 | 0.425364881 | 0.708094791 | 0.610091804 | 0.638341368 | 0.428844012 | 0.623211525 | 0.57565096 | 0.755643243 | 0.86431917 | 0.517820746 | 0.548764345 | 0.783612128 | 0.483277022 | 0.721288913 | 0.288765812 | 0.740844878 | 0.653839642 | 0.624979115 | 0.540396998 | 0.442888195 | 0.494513057 |
| TCGA_TCGA-MP-A4TC | 0.251304088 | 0.599837157 | 0.497805818 | 0.658103097 | 0.595556534 | 0.661634707 | 0.253026538 | 0.629572559 | 0.452819122 | 0.718731601 | 0.801559786 | 0.423571709 | 0.535419047 | 0.73792775 | 0.5127148 | 0.714056168 | 0.199019438 | 0.700489071 | 0.744730522 | 0.54987804 | 0.558208272 | 0.39277695 | 0.509954572 |
| TCGA_TCGA-05-4420 | 0.1839359 | 0.498215373 | 0.45491401 | 0.646607584 | 0.587427956 | 0.73162023 | 0.217266658 | 0.605986787 | 0.30017038 | 0.716114555 | 0.689805566 | 0.339879746 | 0.405539242 | 0.76325683 | 0.442347153 | 0.625088529 | 0.31984212 | 0.687690259 | 0.555108731 | 0.469808544 | 0.460348087 | 0.316519323 | 0.504179116 |
| TCGA_TCGA-75-5146 | 0.421996244 | 0.473047308 | 0.541702611 | 0.640511443 | 0.581433221 | 0.615715958 | 0.369646052 | 0.575719079 | 0.503715149 | 0.688581619 | 0.690917972 | 0.3951362 | 0.483633788 | 0.743328307 | 0.429136929 | 0.619371757 | 0.247111124 | 0.689761623 | 0.585419173 | 0.523949461 | 0.548770312 | 0.337890968 | 0.566762108 |
| TCGA_TCGA-78-7167 | 0.213753432 | 0.248438505 | 0.406370951 | 0.600648011 | 0.518026167 | 0.663876556 | 0.268688479 | 0.497096135 | 0.347447576 | 0.631237108 | 0.504896327 | 0.386775998 | 0.451043902 | 0.704792721 | 0.372084443 | 0.615935237 | 0.20558793 | 0.651107406 | 0.388665805 | 0.514607418 | 0.438353829 | 0.392187894 | 0.454263658 |
| TCGA_TCGA-J2-8194 | 0.223181106 | 0.454546508 | 0.419900358 | 0.672843019 | 0.593538086 | 0.653551092 | 0.388454329 | 0.589605183 | 0.393036112 | 0.718880693 | 0.717543203 | 0.443073407 | 0.523474699 | 0.731178186 | 0.430795367 | 0.673910651 | 0.235520475 | 0.678547642 | 0.614937266 | 0.545633342 | 0.503393817 | 0.416849976 | 0.524815161 |
| TCGA_TCGA-97-A4M0 | 0.295590057 | 0.486688171 | 0.590472022 | 0.693172628 | 0.586036945 | 0.683039868 | 0.357301571 | 0.593496935 | 0.512954731 | 0.690511126 | 0.8162619 | 0.452331282 | 0.530374975 | 0.757561868 | 0.423240681 | 0.660173416 | 0.180296926 | 0.666879999 | 0.599015834 | 0.562453806 | 0.527610876 | 0.44082426 | 0.432023969 |
| TCGA_TCGA-38-4632 | 0.257411222 | 0.652778943 | 0.583798097 | 0.698464935 | 0.684171352 | 0.704584597 | 0.289965158 | 0.685053843 | 0.503262832 | 0.760089737 | 0.837594182 | 0.470562656 | 0.530024797 | 0.770072632 | 0.530125023 | 0.668489582 | 0.281311906 | 0.704108829 | 0.737725576 | 0.612844436 | 0.577640488 | 0.4078476 | 0.542662147 |
| TCGA_TCGA-44-7661 | 0.325485105 | 0.630061168 | 0.500987101 | 0.70344938 | 0.619426802 | 0.658724632 | 0.304787984 | 0.729155789 | 0.489697919 | 0.708120564 | 0.869718427 | 0.474382103 | 0.558523104 | 0.761506467 | 0.562831787 | 0.741305512 | 0.204924886 | 0.723922191 | 0.777127733 | 0.588725172 | 0.579533418 | 0.37643344 | 0.568870508 |
| TCGA_TCGA-55-8205 | 0.418323342 | 0.665046532 | 0.629671893 | 0.722778382 | 0.588520776 | 0.636708731 | 0.411511871 | 0.711992173 | 0.604861927 | 0.720492617 | 0.948986455 | 0.511761666 | 0.504516674 | 0.766819684 | 0.580327077 | 0.730041392 | 0.265027793 | 0.731893172 | 0.801349178 | 0.618411838 | 0.60278219 | 0.457183929 | 0.548238382 |
| TCGA_TCGA-55-A48Z | 0.26161967 | 0.476954529 | 0.470784622 | 0.633332779 | 0.599366016 | 0.684437157 | 0.214093514 | 0.640616822 | 0.464999723 | 0.689216172 | 0.772682246 | 0.390106882 | 0.458047392 | 0.743191905 | 0.483378055 | 0.638395007 | 0.1548979 | 0.685460687 | 0.671119126 | 0.53201874 | 0.559444818 | 0.388978209 | 0.467989685 |
| TCGA_TCGA-44-5645 | 0.385920443 | 0.481860847 | 0.440631676 | 0.674945277 | 0.533688129 | 0.598916535 | 0.429658092 | 0.562432895 | 0.588464053 | 0.628598639 | 0.706166824 | 0.382315174 | 0.554904237 | 0.707336085 | 0.429656076 | 0.680466787 | 0.265915919 | 0.653238444 | 0.654400665 | 0.542219361 | 0.522427748 | 0.436767347 | 0.565329789 |
| TCGA_TCGA-05-5428 | 0.116230286 | 0.518461939 | 0.518053517 | 0.692814252 | 0.60880483 | 0.741981359 | 0.274316924 | 0.674633068 | 0.39950553 | 0.718758713 | 0.770052633 | 0.43435172 | 0.555826968 | 0.735384611 | 0.490593785 | 0.62215648 | 0.287646008 | 0.642796605 | 0.696670477 | 0.570785873 | 0.515215545 | 0.373518947 | 0.48523591 |
| TCGA_TCGA-69-8255 | 0.366469654 | 0.708622665 | 0.741249367 | 0.635303495 | 0.610483649 | 0.723692394 | 0.288105405 | 0.611244003 | 0.528967445 | 0.586025301 | 0.754288458 | 0.435159395 | 0.355353527 | 0.826602935 | 0.496447379 | 0.63583202 | 0.320596016 | 0.647055238 | 0.559168517 | 0.496572316 | 0.488420336 | 0.345165608 | 0.549889425 |
| TCGA_TCGA-75-5125 | 0.287896689 | 0.627957792 | 0.563545243 | 0.678147644 | 0.600704875 | 0.679015331 | 0.284190182 | 0.676597551 | 0.501990542 | 0.711943641 | 0.854436402 | 0.426099415 | 0.466516616 | 0.749104872 | 0.523363983 | 0.674103004 | 0.203280786 | 0.688352133 | 0.72357942 | 0.61640566 | 0.524174309 | 0.405629818 | 0.485557095 |
| TCGA_TCGA-55-8097 | 0.311431969 | 0.340015514 | 0.472619343 | 0.61175082 | 0.546587873 | 0.631502806 | 0.385875178 | 0.580736476 | 0.427756418 | 0.653823002 | 0.698596096 | 0.421011826 | 0.602620327 | 0.757809474 | 0.388858824 | 0.637983106 | 0.168772646 | 0.661922318 | 0.53319176 | 0.543327379 | 0.487668981 | 0.433532533 | 0.465287504 |
| TCGA_TCGA-78-7152 | 0.416332865 | 0.519668356 | 0.541584223 | 0.644372668 | 0.59595321 | 0.680107381 | 0.235662078 | 0.555922446 | 0.509431943 | 0.66027681 | 0.687579063 | 0.364482291 | 0.450449689 | 0.757025267 | 0.440912114 | 0.646127943 | 0.121112371 | 0.677695661 | 0.580769458 | 0.534434623 | 0.52570758 | 0.346099808 | 0.481969908 |
| TCGA_TCGA-MP-A4TF | 0.232963449 | 0.47422989 | 0.440088834 | 0.643856231 | 0.608570058 | 0.741568315 | 0.289607914 | 0.589262084 | 0.39095228 | 0.681519574 | 0.623497645 | 0.295726623 | 0.451046451 | 0.739057578 | 0.434333837 | 0.609244559 | 0.262478882 | 0.661712105 | 0.517702278 | 0.468291763 | 0.470040793 | 0.399900258 | 0.53363937 |
| TCGA_TCGA-67-4679 | 0.213779996 | 0.40420631 | 0.441186619 | 0.691612712 | 0.581538699 | 0.647780508 | 0.394381275 | 0.585410929 | 0.444248255 | 0.741675288 | 0.760400193 | 0.476992895 | 0.665197462 | 0.763421528 | 0.435903453 | 0.654488347 | 0.191270501 | 0.705731203 | 0.621873613 | 0.601956183 | 0.526070213 | 0.423005642 | 0.500466819 |
| TCGA_TCGA-91-6836 | 0.238728953 | 0.608506501 | 0.441986948 | 0.575573509 | 0.603698876 | 0.703603569 | 0.233466721 | 0.627020806 | 0.383596645 | 0.661024327 | 0.592075076 | 0.348306581 | 0.534096311 | 0.719081432 | 0.478886734 | 0.565395039 | 0.193507396 | 0.608569755 | 0.521397826 | 0.496366762 | 0.4511516 | 0.327894709 | 0.54594724 |
| TCGA_TCGA-78-8648 | 0.507314406 | 0.55495231 | 0.667255049 | 0.692456494 | 0.580818053 | 0.632532477 | 0.464826599 | 0.687632126 | 0.6141023 | 0.722934655 | 0.932825669 | 0.556363515 | 0.686770669 | 0.802743787 | 0.610410403 | 0.700487258 | 0.146284311 | 0.77111995 | 0.746537424 | 0.658149794 | 0.618376368 | 0.388762961 | 0.566752973 |
| TCGA_TCGA-44-A47A | 0.208061476 | 0.504527524 | 0.481459339 | 0.699253031 | 0.636911632 | 0.694527146 | 0.303921083 | 0.608022283 | 0.464041902 | 0.729313462 | 0.776101816 | 0.465303251 | 0.463394758 | 0.764338633 | 0.431826209 | 0.663757122 | 0.19545506 | 0.705745136 | 0.653007333 | 0.579962868 | 0.513200584 | 0.42949307 | 0.458241891 |
| TCGA_TCGA-55-A57B | 0.18794972 | 0.422277155 | 0.428470814 | 0.648365549 | 0.545233992 | 0.633889723 | 0.399546511 | 0.602972772 | 0.421045262 | 0.687109316 | 0.706694104 | 0.385712275 | 0.442235011 | 0.747696666 | 0.415483321 | 0.695722794 | 0.153966672 | 0.659755403 | 0.607299608 | 0.555444734 | 0.491132065 | 0.38989483 | 0.480036997 |
| TCGA_TCGA-55-8207 | 0.193437985 | 0.387242414 | 0.450741374 | 0.693086582 | 0.566377 | 0.66751231 | 0.36457297 | 0.600615312 | 0.44421156 | 0.724259251 | 0.808500687 | 0.498943894 | 0.651401309 | 0.730070473 | 0.507893863 | 0.69526027 | 0.255535826 | 0.734166313 | 0.721282987 | 0.571775558 | 0.532173624 | 0.410392382 | 0.475649038 |
| TCGA_TCGA-55-7576 | 0.28534781 | 0.514616045 | 0.493575205 | 0.611955215 | 0.605580965 | 0.686963461 | 0.264826484 | 0.646002286 | 0.484227562 | 0.700732638 | 0.723682946 | 0.401289261 | 0.511844494 | 0.745885429 | 0.482293334 | 0.642168541 | 0.229299782 | 0.685434109 | 0.657033708 | 0.556438769 | 0.519937142 | 0.378098124 | 0.540584276 |
| TCGA_TCGA-NJ-A55O | 0.275289792 | 0.39159717 | 0.472771921 | 0.617626759 | 0.597179263 | 0.710879787 | 0.390478928 | 0.650148395 | 0.451278138 | 0.691346049 | 0.724245558 | 0.47683872 | 0.447095306 | 0.755369035 | 0.478498463 | 0.623438576 | 0.196553114 | 0.730334807 | 0.609513036 | 0.571306867 | 0.505428819 | 0.391893763 | 0.587741052 |
| TCGA_TCGA-55-A494 | 0.029839631 | 0.348433897 | 0.409362966 | 0.533264638 | 0.612313109 | 0.767400618 | 0.253995554 | 0.520985916 | 0.237952416 | 0.673682478 | 0.4310798 | 0.277917557 | 0.420252952 | 0.750941907 | 0.362077164 | 0.57129094 | 0.300508408 | 0.632414393 | 0.361862373 | 0.474011534 | 0.451428188 | 0.356440731 | 0.489277879 |
| TCGA_TCGA-95-7043 | 0.170684696 | 0.411387103 | 0.450499408 | 0.495868233 | 0.463994309 | 0.577437039 | 0.30625082 | 0.526995385 | 0.303825864 | 0.648800985 | 0.496253438 | 0.371674551 | 0.340357179 | 0.680206181 | 0.414715644 | 0.559411347 | 0.167495723 | 0.63274415 | 0.39082667 | 0.476720455 | 0.431057281 | 0.400600736 | 0.429558322 |
| TCGA_TCGA-L9-A443 | 0.206590445 | 0.445410586 | 0.488337693 | 0.634836837 | 0.554837859 | 0.720358605 | 0.280769617 | 0.52324617 | 0.377103659 | 0.685447244 | 0.717882563 | 0.389142073 | 0.544079684 | 0.710007004 | 0.473019207 | 0.654854075 | 0.264512036 | 0.662384616 | 0.466414334 | 0.521620082 | 0.522374783 | 0.423452026 | 0.47238767 |
| TCGA_TCGA-69-7974 | 0.325352544 | 0.603249601 | 0.500334222 | 0.676542411 | 0.587791707 | 0.647200075 | 0.38024195 | 0.676001088 | 0.5047818 | 0.743485876 | 0.844551036 | 0.524275631 | 0.505985249 | 0.765508885 | 0.540787221 | 0.701348235 | 0.243312066 | 0.683433233 | 0.765069932 | 0.586565792 | 0.538630912 | 0.412450592 | 0.56231501 |
| TCGA_TCGA-NJ-A4YG | 0.242429326 | 0.40870884 | 0.503125378 | 0.697580773 | 0.622091418 | 0.663762505 | 0.309818277 | 0.596100768 | 0.437456936 | 0.724960731 | 0.756599257 | 0.454587867 | 0.490401707 | 0.758321273 | 0.467581324 | 0.674333412 | 0.199650705 | 0.68413676 | 0.593957495 | 0.547332487 | 0.487621933 | 0.416024588 | 0.470276498 |
| TCGA_TCGA-69-7760 | 0.14140426 | 0.457913016 | 0.41035179 | 0.582625853 | 0.675615926 | 0.746554977 | 0.30901979 | 0.623824325 | 0.303916527 | 0.682893095 | 0.530832791 | 0.291849497 | 0.422901452 | 0.753184996 | 0.438018785 | 0.63265816 | 0.131923608 | 0.637284019 | 0.480430536 | 0.467286094 | 0.444711025 | 0.380792936 | 0.572389985 |
| TCGA_TCGA-49-4486 | 0.192407057 | 0.316406706 | 0.478712097 | 0.590653201 | 0.580681276 | 0.600702917 | 0.413234328 | 0.595805483 | 0.310784698 | 0.63663943 | 0.537224647 | 0.362436164 | 0.473632936 | 0.711485228 | 0.330415199 | 0.518039435 | 0.245285069 | 0.599084742 | 0.375528027 | 0.469898117 | 0.440984202 | 0.375942285 | 0.486246349 |
| TCGA_TCGA-93-A4JN | 0.434845558 | 0.485522947 | 0.424746023 | 0.614418139 | 0.558511138 | 0.580174023 | 0.364160752 | 0.588504872 | 0.543955681 | 0.664484578 | 0.683190288 | 0.358699172 | 0.552601558 | 0.750265854 | 0.432076753 | 0.70420185 | 0.150813994 | 0.657083792 | 0.544986965 | 0.544377816 | 0.517414821 | 0.385963403 | 0.581207156 |
| TCGA_TCGA-86-8073 | 0.200783633 | 0.39465658 | 0.403190026 | 0.606630567 | 0.55025882 | 0.583680854 | 0.335910961 | 0.586886734 | 0.420189885 | 0.711166455 | 0.651366573 | 0.430544175 | 0.519319167 | 0.725375906 | 0.461190329 | 0.629217059 | 0.150759587 | 0.689492919 | 0.682523924 | 0.535172334 | 0.518077921 | 0.382834957 | 0.496352897 |
| TCGA_TCGA-L9-A7SV | 0.201795964 | 0.477417125 | 0.437462836 | 0.511144194 | 0.525715123 | 0.747928553 | 0.423373709 | 0.54005022 | 0.380074388 | 0.703087493 | 0.514071604 | 0.267463227 | 0.345632536 | 0.70503788 | 0.367662901 | 0.545028345 | 0.135560777 | 0.621444476 | 0.440586855 | 0.52449938 | 0.434934468 | 0.361683223 | 0.49502845 |
| TCGA_TCGA-93-A4JP | 0.229074458 | 0.38414712 | 0.417583344 | 0.644848324 | 0.614147972 | 0.595118243 | 0.438655318 | 0.660073864 | 0.472005195 | 0.692874925 | 0.77670546 | 0.419986765 | 0.580847351 | 0.760177854 | 0.475432562 | 0.670622237 | 0.180179547 | 0.739427135 | 0.60377176 | 0.555940229 | 0.500424091 | 0.361257643 | 0.522020812 |
| TCGA_TCGA-78-7163 | 0.192023999 | 0.340832012 | 0.517092029 | 0.601260384 | 0.612185994 | 0.680358397 | 0.191838373 | 0.514359597 | 0.344669133 | 0.74762793 | 0.550525449 | 0.27087312 | 0.326467225 | 0.764386456 | 0.370786136 | 0.523407906 | 0.157549588 | 0.68396721 | 0.414285361 | 0.403676718 | 0.437885356 | 0.389681523 | 0.438857326 |
| TCGA_TCGA-64-1678 | 0.217257707 | 0.595856597 | 0.56019656 | 0.550791475 | 0.592403127 | 0.750266001 | 0.167140371 | 0.569675517 | 0.339624786 | 0.599411567 | 0.496409234 | 0.295032145 | 0.435543516 | 0.722269577 | 0.400082323 | 0.557255111 | 0.200928632 | 0.612118348 | 0.422597422 | 0.425686895 | 0.401899764 | 0.294821686 | 0.540117934 |
| TCGA_TCGA-NJ-A55R | 0.394551098 | 0.372853619 | 0.391829677 | 0.605029639 | 0.536272464 | 0.731215328 | 0.280142147 | 0.484503605 | 0.442829521 | 0.612607091 | 0.574564961 | 0.317210734 | 0.448089579 | 0.737286319 | 0.390711385 | 0.611607826 | 0.202091209 | 0.683279998 | 0.432338167 | 0.519721793 | 0.432559247 | 0.399573029 | 0.44114156 |
| TCGA_TCGA-97-A4M7 | 0.391194995 | 0.481237579 | 0.506890762 | 0.684397841 | 0.626798324 | 0.689307411 | 0.389314375 | 0.598575666 | 0.518161244 | 0.743227425 | 0.825311136 | 0.448946236 | 0.568607454 | 0.786029794 | 0.446487835 | 0.726187496 | 0.241465189 | 0.697921183 | 0.69881039 | 0.605086234 | 0.538695807 | 0.408588943 | 0.52641057 |
| TCGA_TCGA-38-A44F | 0.558678846 | 0.479259768 | 0.558589491 | 0.716022053 | 0.606739146 | 0.652944446 | 0.433890662 | 0.611220653 | 0.58708643 | 0.73188126 | 0.845822997 | 0.504298171 | 0.58252393 | 0.781723567 | 0.489434114 | 0.721813894 | 0.239493287 | 0.737740771 | 0.70043597 | 0.572688156 | 0.567915128 | 0.493907658 | 0.504446905 |
| TCGA_TCGA-62-8399 | 0.243864455 | 0.459888408 | 0.404868405 | 0.61676025 | 0.566992539 | 0.623698471 | 0.487504081 | 0.618731458 | 0.398894331 | 0.733244993 | 0.69194847 | 0.334490799 | 0.539063553 | 0.724499959 | 0.427128876 | 0.60099847 | 0.249457243 | 0.631703803 | 0.555577366 | 0.558819951 | 0.496673356 | 0.340992046 | 0.489954157 |
| TCGA_TCGA-49-4514 | 0.380194071 | 0.602875516 | 0.614643042 | 0.64965116 | 0.619570015 | 0.725569843 | 0.253816687 | 0.636806832 | 0.41689456 | 0.661481598 | 0.688242203 | 0.409898386 | 0.381347421 | 0.767978499 | 0.432896357 | 0.597303738 | 0.270889382 | 0.638414082 | 0.458518225 | 0.499641401 | 0.521325148 | 0.373890816 | 0.542367722 |
| TCGA_TCGA-44-5643 | 0.330652135 | 0.660124531 | 0.542893215 | 0.615946909 | 0.666188858 | 0.662036997 | 0.299750385 | 0.54549741 | 0.470236782 | 0.678958102 | 0.624185771 | 0.364901661 | 0.333363852 | 0.707656959 | 0.421648833 | 0.611514084 | 0.094909305 | 0.676166893 | 0.533039434 | 0.512373157 | 0.501896281 | 0.348540508 | 0.477124813 |
| TCGA_TCGA-44-6147 | 0.466630175 | 0.515962061 | 0.433745115 | 0.61286731 | 0.563201121 | 0.581604354 | 0.477935579 | 0.536155565 | 0.646489729 | 0.640927892 | 0.678225949 | 0.378852776 | 0.551750967 | 0.751410674 | 0.417437751 | 0.661065564 | 0.20660502 | 0.667953506 | 0.573178072 | 0.559749667 | 0.515700401 | 0.389011092 | 0.565946609 |
| TCGA_TCGA-J2-A4AE | 0.410679842 | 0.555614256 | 0.469542397 | 0.659773532 | 0.552045771 | 0.649638827 | 0.483654366 | 0.605623832 | 0.509710365 | 0.717844303 | 0.719495054 | 0.366809616 | 0.502991083 | 0.736990404 | 0.419665594 | 0.628618667 | 0.212819165 | 0.691167072 | 0.594010244 | 0.535344431 | 0.531990734 | 0.386203889 | 0.568359841 |
| TCGA_TCGA-05-4415 | 0.256610356 | 0.658753623 | 0.494670788 | 0.60848186 | 0.562221471 | 0.711468726 | 0.287221405 | 0.657754292 | 0.367580127 | 0.608305674 | 0.663826771 | 0.3163904 | 0.435331464 | 0.698707279 | 0.47202912 | 0.574894937 | 0.184710703 | 0.623321425 | 0.59641366 | 0.477009775 | 0.450934254 | 0.334242206 | 0.613584469 |
| TCGA_TCGA-91-6830 | 0.261252016 | 0.471856893 | 0.488186827 | 0.682225111 | 0.630132852 | 0.608621458 | 0.365221535 | 0.629335922 | 0.466952309 | 0.718725419 | 0.712892874 | 0.484301087 | 0.559436245 | 0.731850661 | 0.523562491 | 0.704259677 | 0.269270598 | 0.736894972 | 0.642105569 | 0.564527614 | 0.564639652 | 0.368405273 | 0.489722629 |
| TCGA_TCGA-49-6745 | 0.287590289 | 0.569287318 | 0.538455352 | 0.70029715 | 0.599445889 | 0.607688303 | 0.346159759 | 0.677959447 | 0.474526208 | 0.734244774 | 0.809983947 | 0.444692427 | 0.530010919 | 0.777359986 | 0.533721303 | 0.702447562 | 0.272497192 | 0.697226161 | 0.735683469 | 0.552485871 | 0.570766814 | 0.418617291 | 0.561766117 |
| TCGA_TCGA-86-A4P7 | 0.433508286 | 0.44746784 | 0.555790184 | 0.624667255 | 0.577458832 | 0.639969976 | 0.415412552 | 0.626245053 | 0.559514696 | 0.6740733 | 0.820406685 | 0.463542359 | 0.592093396 | 0.760692604 | 0.473867904 | 0.676100883 | 0.169539401 | 0.729712133 | 0.632143955 | 0.610527297 | 0.538262 | 0.374710179 | 0.529436605 |
| TCGA_TCGA-44-4112 | 0.236391568 | 0.538456784 | 0.395172305 | 0.666004838 | 0.570238721 | 0.627427719 | 0.408310245 | 0.641549201 | 0.565721118 | 0.637052975 | 0.647295371 | 0.398459406 | 0.549756578 | 0.734457043 | 0.479446846 | 0.661066689 | 0.184937577 | 0.658855285 | 0.635288489 | 0.545761932 | 0.499521432 | 0.380538592 | 0.578611463 |
| TCGA_TCGA-55-6975 | 0.327704679 | 0.569595194 | 0.464278505 | 0.586499601 | 0.589268192 | 0.663568833 | 0.32308276 | 0.616960498 | 0.423693247 | 0.658821753 | 0.675273349 | 0.379282931 | 0.411840876 | 0.757662518 | 0.496038037 | 0.618507529 | 0.218283626 | 0.692255796 | 0.548651038 | 0.476181132 | 0.50845927 | 0.357762136 | 0.553592146 |
| TCGA_TCGA-95-7039 | 0.205842123 | 0.468357512 | 0.451270569 | 0.636185444 | 0.576180338 | 0.718237923 | 0.372698747 | 0.598792304 | 0.397785919 | 0.672796344 | 0.597628741 | 0.321556797 | 0.516514956 | 0.765456738 | 0.495832038 | 0.662121931 | 0.243097071 | 0.645049307 | 0.53314997 | 0.583425671 | 0.515699022 | 0.393499236 | 0.535402691 |
| TCGA_TCGA-05-4432 | 0.337014129 | 0.533199939 | 0.512203835 | 0.661036043 | 0.587319542 | 0.753585467 | 0.253272547 | 0.594105022 | 0.513961846 | 0.709357685 | 0.739225559 | 0.385307266 | 0.532642392 | 0.780014253 | 0.450639592 | 0.671729109 | 0.208298273 | 0.663133333 | 0.661990951 | 0.535996622 | 0.476376337 | 0.365677527 | 0.529043771 |
| TCGA_TCGA-49-4487 | 0.607577698 | 0.728524129 | 0.690113824 | 0.682547302 | 0.627151331 | 0.666583665 | 0.31510641 | 0.639087292 | 0.666211847 | 0.702746845 | 0.851948954 | 0.432523874 | 0.528212883 | 0.789343286 | 0.477704843 | 0.668402363 | 0.165522014 | 0.7025303 | 0.62352717 | 0.576051177 | 0.589104366 | 0.369807791 | 0.575242135 |
| TCGA_TCGA-55-7727 | 0.407544896 | 0.615717258 | 0.507376231 | 0.661918919 | 0.623283333 | 0.601156276 | 0.291329753 | 0.599567314 | 0.594997139 | 0.728830198 | 0.668797665 | 0.413978973 | 0.548017514 | 0.714341304 | 0.429743421 | 0.628277183 | 0.188534466 | 0.652835122 | 0.627190023 | 0.538263852 | 0.504273178 | 0.355887184 | 0.618954911 |
| TCGA_TCGA-73-A9RS | 0.176518913 | 0.566729821 | 0.48356943 | 0.575448256 | 0.580826355 | 0.657439837 | 0.306748497 | 0.588747048 | 0.365465171 | 0.595973749 | 0.580681349 | 0.348276605 | 0.452620053 | 0.710142234 | 0.383430152 | 0.550853916 | 0.311415863 | 0.615301331 | 0.441526066 | 0.45383018 | 0.401612529 | 0.371735582 | 0.455165662 |
| TCGA_TCGA-44-5644 | 0.162950194 | 0.473253532 | 0.412082328 | 0.550338685 | 0.570520341 | 0.794513178 | 0.371527491 | 0.562961058 | 0.296648088 | 0.516604736 | 0.539147325 | 0.27295495 | 0.386791788 | 0.75189266 | 0.422495497 | 0.571645898 | 0.393908576 | 0.626819103 | 0.426992968 | 0.501184734 | 0.392605559 | 0.380676412 | 0.465702755 |
| TCGA_TCGA-55-7914 | 0.377197694 | 0.455268067 | 0.52124102 | 0.618681801 | 0.616520453 | 0.632042418 | 0.266833591 | 0.5405104 | 0.52111631 | 0.682812194 | 0.660721446 | 0.34665662 | 0.512126006 | 0.725624625 | 0.442054495 | 0.623867257 | 0.113387897 | 0.666710935 | 0.475507257 | 0.546532608 | 0.527625924 | 0.42209327 | 0.522736139 |
| TCGA_TCGA-62-A46R | 0.260266186 | 0.571707834 | 0.517738237 | 0.679161477 | 0.599522384 | 0.684734891 | 0.305886996 | 0.632590958 | 0.526821666 | 0.73724998 | 0.767440832 | 0.39502737 | 0.5375044 | 0.743373449 | 0.443502629 | 0.639374338 | 0.239445958 | 0.666163587 | 0.604701016 | 0.539218283 | 0.506576443 | 0.400506799 | 0.509194948 |
| TCGA_TCGA-49-AARQ | 0.258606633 | 0.542033642 | 0.536070214 | 0.628656994 | 0.633571699 | 0.687040661 | 0.3069584 | 0.579533681 | 0.436841119 | 0.685333484 | 0.717081686 | 0.37469166 | 0.367499648 | 0.751509198 | 0.397630781 | 0.651409865 | 0.089545863 | 0.614109233 | 0.42639226 | 0.517115844 | 0.458314265 | 0.394583496 | 0.456235428 |
| TCGA_TCGA-75-6207 | 0.243400167 | 0.486655664 | 0.488471956 | 0.635505291 | 0.588816283 | 0.628255977 | 0.359302025 | 0.649773255 | 0.411698058 | 0.68794734 | 0.668324919 | 0.3666677 | 0.393926716 | 0.744831425 | 0.433818556 | 0.58394347 | 0.171419644 | 0.610771066 | 0.601647987 | 0.526831206 | 0.46589938 | 0.3967893 | 0.498602967 |
| TCGA_TCGA-53-7813 | 0.372552988 | 0.564821947 | 0.558081205 | 0.630193519 | 0.622427141 | 0.732409971 | 0.330108243 | 0.525824448 | 0.51502963 | 0.660801115 | 0.586462366 | 0.337821734 | 0.44580563 | 0.714810358 | 0.372812479 | 0.631767618 | 0.199082925 | 0.643346577 | 0.433986871 | 0.468582293 | 0.44051353 | 0.369217098 | 0.624712218 |
| TCGA_TCGA-44-3918 | 0.369299441 | 0.601831558 | 0.586735985 | 0.700719728 | 0.608417254 | 0.691547721 | 0.457120289 | 0.657189484 | 0.64419774 | 0.632477494 | 0.843861665 | 0.457664938 | 0.527359482 | 0.709571163 | 0.512867644 | 0.715227151 | 0.232343734 | 0.658091509 | 0.74100489 | 0.606746343 | 0.570770841 | 0.435257745 | 0.561577014 |
| TCGA_TCGA-64-1681 | 0.329003392 | 0.482067997 | 0.447694839 | 0.685859215 | 0.624244681 | 0.654942759 | 0.340628073 | 0.610455005 | 0.462373528 | 0.711890694 | 0.688249237 | 0.378817082 | 0.524680883 | 0.783580239 | 0.451471899 | 0.684419818 | 0.185944328 | 0.696215655 | 0.582193328 | 0.574250465 | 0.505830694 | 0.466395869 | 0.488578644 |
| TCGA_TCGA-05-4410 | 0.482482445 | 0.514026111 | 0.537446015 | 0.676412328 | 0.567270848 | 0.695687935 | 0.323567639 | 0.581854436 | 0.584706037 | 0.657182343 | 0.833285506 | 0.38011116 | 0.46378185 | 0.772687921 | 0.454081213 | 0.68499153 | 0.259699751 | 0.653927649 | 0.580915587 | 0.578032961 | 0.53580108 | 0.407860833 | 0.500079928 |
| TCGA_TCGA-55-8620 | 0.311132403 | 0.508334507 | 0.511107903 | 0.624487916 | 0.512794378 | 0.667101532 | 0.221401719 | 0.591006811 | 0.467694745 | 0.566241554 | 0.610207973 | 0.307563556 | 0.288759633 | 0.727838191 | 0.370431103 | 0.631100991 | 0.069484651 | 0.656487403 | 0.584248608 | 0.484596499 | 0.397296971 | 0.366873352 | 0.428120262 |
| TCGA_TCGA-64-5774 | 0.215218342 | 0.526298702 | 0.474267721 | 0.594664967 | 0.561856825 | 0.695116055 | 0.23093321 | 0.579061115 | 0.338293434 | 0.639365768 | 0.590234309 | 0.268047974 | 0.421260479 | 0.706856329 | 0.455534229 | 0.638090627 | 0.303988999 | 0.610527277 | 0.498189337 | 0.44486026 | 0.456550871 | 0.336894723 | 0.529555293 |
| TCGA_TCGA-86-A4D0 | 0.173415939 | 0.614646036 | 0.509369974 | 0.591089876 | 0.590357253 | 0.728840293 | 0.274648123 | 0.571874312 | 0.333454818 | 0.601556919 | 0.535775453 | 0.310518802 | 0.361093746 | 0.730620358 | 0.388543203 | 0.630079097 | 0.190624092 | 0.612994244 | 0.493943527 | 0.466859244 | 0.424710596 | 0.370131572 | 0.542460897 |
| TCGA_TCGA-L9-A444 | 0.506368536 | 0.54714187 | 0.743933421 | 0.686843419 | 0.568850514 | 0.753396434 | 0.302568112 | 0.628995081 | 0.596968688 | 0.634504319 | 0.873482022 | 0.448701231 | 0.489681235 | 0.779626747 | 0.491965921 | 0.678652624 | 0.187054837 | 0.693183426 | 0.631429871 | 0.604897467 | 0.584645355 | 0.431286508 | 0.486544377 |
| TCGA_TCGA-MP-A4T7 | 0.261141767 | 0.464294039 | 0.480128138 | 0.645030199 | 0.587610841 | 0.686620704 | 0.39456535 | 0.611667852 | 0.434000831 | 0.711109918 | 0.743796303 | 0.45630213 | 0.464452991 | 0.752207244 | 0.42445581 | 0.594737733 | 0.291871053 | 0.698571933 | 0.591070385 | 0.537704465 | 0.484704875 | 0.381518989 | 0.556570089 |
| TCGA_TCGA-91-A4BD | 0.25803041 | 0.384520763 | 0.524961609 | 0.691977703 | 0.614015012 | 0.680839388 | 0.399008461 | 0.62943258 | 0.474721553 | 0.776342897 | 0.86824763 | 0.529045783 | 0.623576411 | 0.7872887 | 0.427961352 | 0.646625519 | 0.258247733 | 0.744280212 | 0.642692367 | 0.564577144 | 0.498434098 | 0.407074321 | 0.467696918 |
| TCGA_TCGA-05-4250 | 0.241213493 | 0.636085208 | 0.595406931 | 0.706891503 | 0.618416542 | 0.66934469 | 0.336780859 | 0.661662506 | 0.444266514 | 0.680373855 | 0.755740385 | 0.426774214 | 0.457901003 | 0.765928616 | 0.533911033 | 0.681356531 | 0.225884835 | 0.667368697 | 0.705409721 | 0.573740385 | 0.571820824 | 0.431830605 | 0.516281168 |
| TCGA_TCGA-55-7994 | 0.347206881 | 0.624511077 | 0.621619582 | 0.701252056 | 0.576575979 | 0.641904932 | 0.301165178 | 0.639794115 | 0.565978031 | 0.669067618 | 0.831506202 | 0.430982027 | 0.452745332 | 0.778753183 | 0.47376375 | 0.697450388 | 0.272126917 | 0.660433893 | 0.708880693 | 0.602203143 | 0.527257249 | 0.467830878 | 0.509665234 |
| TCGA_TCGA-99-8028 | 0.533088892 | 0.642352279 | 0.738333164 | 0.717212961 | 0.657971028 | 0.723060257 | 0.390655344 | 0.672837412 | 0.637106746 | 0.745554449 | 0.989530135 | 0.517949775 | 0.608631174 | 0.79764906 | 0.558885768 | 0.76112285 | 0.177875101 | 0.746432869 | 0.799931354 | 0.644307085 | 0.645346011 | 0.438283674 | 0.568562695 |
| TCGA_TCGA-97-8171 | 0.154984019 | 0.326876717 | 0.437023371 | 0.539915133 | 0.531870386 | 0.652044787 | 0.283447539 | 0.58244964 | 0.369031802 | 0.658251015 | 0.536022383 | 0.316356637 | 0.288883861 | 0.702342559 | 0.37544907 | 0.579948048 | 0.099393828 | 0.624743891 | 0.38601749 | 0.504263923 | 0.403157508 | 0.389695658 | 0.429878486 |
| TCGA_TCGA-67-6216 | 0.382399553 | 0.486484148 | 0.486009619 | 0.728155773 | 0.627299433 | 0.685308281 | 0.374454861 | 0.590487198 | 0.502811578 | 0.731883952 | 0.811497873 | 0.457222547 | 0.542748332 | 0.789302619 | 0.434341787 | 0.669830016 | 0.260171287 | 0.728990002 | 0.681193033 | 0.531306903 | 0.502601895 | 0.402589583 | 0.534764198 |
| TCGA_TCGA-91-6848 | 0.325659015 | 0.699293011 | 0.563776158 | 0.670532931 | 0.561418072 | 0.652295353 | 0.404148891 | 0.699925849 | 0.501978266 | 0.612292598 | 0.81838083 | 0.546344107 | 0.423549298 | 0.74356655 | 0.598991022 | 0.622857099 | 0.201129693 | 0.723168124 | 0.739185627 | 0.599376494 | 0.599313503 | 0.422421638 | 0.520058973 |
| TCGA_TCGA-44-2665 | 0.170418392 | 0.455915658 | 0.414782965 | 0.715056171 | 0.705225023 | 0.66671065 | 0.197788388 | 0.672615274 | 0.402781073 | 0.693607709 | 0.856865022 | 0.479772474 | 0.529388934 | 0.746762122 | 0.519003807 | 0.664231178 | 0.172308361 | 0.73845603 | 0.728332327 | 0.563162269 | 0.53986744 | 0.422939803 | 0.501665331 |
| TCGA_TCGA-86-7701 | 0.544429816 | 0.706474106 | 0.643298122 | 0.687769539 | 0.633480351 | 0.739525454 | 0.294535924 | 0.615529599 | 0.614789171 | 0.632095633 | 0.765134474 | 0.375088004 | 0.485164009 | 0.781784785 | 0.465094093 | 0.702710578 | 0.277698839 | 0.688661012 | 0.640548335 | 0.552549968 | 0.557346136 | 0.408854969 | 0.553326207 |
| TCGA_TCGA-78-7156 | 0.16890287 | 0.341910035 | 0.453862769 | 0.558288123 | 0.553295664 | 0.587626882 | 0.363556806 | 0.526177348 | 0.366982003 | 0.619998237 | 0.520705071 | 0.328965404 | 0.433023107 | 0.726960817 | 0.388832859 | 0.558157458 | 0.20127372 | 0.670790546 | 0.428085117 | 0.496718663 | 0.437612976 | 0.404819474 | 0.474596164 |
| TCGA_TCGA-05-5420 | 0.571087973 | 0.708493187 | 0.793106477 | 0.744917235 | 0.656193451 | 0.697751367 | 0.356956579 | 0.727830258 | 0.658064534 | 0.696820304 | 0.954583318 | 0.570322637 | 0.618933433 | 0.824557735 | 0.565196068 | 0.695123222 | 0.445479862 | 0.748676011 | 0.785854459 | 0.62963316 | 0.622301875 | 0.422759273 | 0.569798168 |
| TCGA_TCGA-55-6642 | 0.493131324 | 0.515106046 | 0.557293903 | 0.635135497 | 0.575304395 | 0.719565562 | 0.25809814 | 0.63246085 | 0.63636612 | 0.647160957 | 0.752227065 | 0.386469837 | 0.569712865 | 0.773563313 | 0.510626702 | 0.687450262 | 0.210786163 | 0.7071398 | 0.645428214 | 0.502379096 | 0.531522731 | 0.367958718 | 0.544398069 |
| TCGA_TCGA-78-7149 | 0.265592392 | 0.296277637 | 0.448559775 | 0.578627986 | 0.573251899 | 0.662139803 | 0.352262614 | 0.556732677 | 0.3348727 | 0.628198139 | 0.558687319 | 0.356218863 | 0.433501917 | 0.733037851 | 0.425617137 | 0.613050515 | 0.211654218 | 0.641540456 | 0.423994651 | 0.500935597 | 0.464907152 | 0.412314197 | 0.419236462 |
| TCGA_TCGA-05-4418 | 0.399059695 | 0.548342291 | 0.57113204 | 0.686468818 | 0.610818072 | 0.701882553 | 0.23794181 | 0.686923729 | 0.530381451 | 0.679813847 | 0.849993853 | 0.460908721 | 0.500678481 | 0.779473664 | 0.478017564 | 0.598954737 | 0.336824371 | 0.711139225 | 0.667105154 | 0.521206036 | 0.537461921 | 0.414236519 | 0.561867843 |
| TCGA_TCGA-05-4422 | 0.415096402 | 0.574378776 | 0.625230258 | 0.699216639 | 0.588601362 | 0.662431917 | 0.294279845 | 0.51929245 | 0.518033105 | 0.693883806 | 0.7782375 | 0.46652765 | 0.403225154 | 0.762692089 | 0.45458599 | 0.589688576 | 0.186580074 | 0.684514586 | 0.562226125 | 0.568255165 | 0.532860396 | 0.378207688 | 0.447494073 |
| TCGA_TCGA-78-7146 | 0.206081915 | 0.599002752 | 0.423901171 | 0.674199039 | 0.655195681 | 0.617213209 | 0.201657671 | 0.629240912 | 0.400517541 | 0.66799225 | 0.694846691 | 0.373343853 | 0.460243457 | 0.7027708 | 0.537195897 | 0.585951249 | 0.222358007 | 0.662220717 | 0.601580987 | 0.499826133 | 0.476438516 | 0.392063221 | 0.387189375 |
| TCGA_TCGA-86-A4P8 | 0.313055706 | 0.525631205 | 0.607057272 | 0.699544193 | 0.593798434 | 0.595691122 | 0.537736659 | 0.649323559 | 0.557649803 | 0.724509736 | 0.903333518 | 0.528055879 | 0.673479272 | 0.78680674 | 0.53297083 | 0.726540675 | 0.182401237 | 0.757374286 | 0.676755792 | 0.658084961 | 0.580834758 | 0.400411954 | 0.560182957 |
| TCGA_TCGA-67-6217 | 0.410639427 | 0.470261137 | 0.529233075 | 0.678512491 | 0.568122485 | 0.665236051 | 0.428683363 | 0.605876435 | 0.516951054 | 0.706736718 | 0.766211971 | 0.374433752 | 0.473877017 | 0.762213183 | 0.435735016 | 0.682860599 | 0.207154833 | 0.68759779 | 0.611596623 | 0.569101659 | 0.512880377 | 0.425792157 | 0.481796351 |
| TCGA_TCGA-78-8640 | 0.251069634 | 0.509393193 | 0.504190378 | 0.660558371 | 0.610577173 | 0.659125605 | 0.26092064 | 0.621847715 | 0.518479901 | 0.709627481 | 0.659027851 | 0.345098348 | 0.424501037 | 0.793616459 | 0.42479332 | 0.574662284 | 0.158621749 | 0.626825851 | 0.597659528 | 0.467860693 | 0.471216162 | 0.320908678 | 0.441513684 |
| TCGA_TCGA-44-7670 | 0.262617576 | 0.555618529 | 0.387503606 | 0.618592673 | 0.606114651 | 0.715562162 | 0.317471575 | 0.544097271 | 0.364927742 | 0.677332838 | 0.52479546 | 0.3221448 | 0.38429178 | 0.706374546 | 0.439416798 | 0.593058187 | 0.131181507 | 0.681186042 | 0.578511776 | 0.489115206 | 0.488935685 | 0.367849956 | 0.525521531 |
| TCGA_TCGA-49-AARO | 0.405266216 | 0.503142762 | 0.567911228 | 0.720687448 | 0.604958677 | 0.683485619 | 0.322891676 | 0.635657473 | 0.510963804 | 0.729721328 | 0.888305813 | 0.48096022 | 0.466550762 | 0.787954536 | 0.529085685 | 0.730761716 | 0.257771783 | 0.725126286 | 0.763699454 | 0.575795032 | 0.548562366 | 0.439191009 | 0.447247194 |
| TCGA_TCGA-75-7030 | 0.440221245 | 0.473965293 | 0.486082289 | 0.678218907 | 0.592285346 | 0.641128155 | 0.394053969 | 0.554240828 | 0.49474858 | 0.705909737 | 0.661495164 | 0.419845503 | 0.539055738 | 0.797063039 | 0.476115082 | 0.696044334 | 0.259126428 | 0.718174959 | 0.594891032 | 0.554943016 | 0.572731499 | 0.484153276 | 0.537515358 |
| TCGA_TCGA-50-5941 | 0.495026793 | 0.602276542 | 0.650947421 | 0.713360893 | 0.64714182 | 0.683124813 | 0.336371004 | 0.642253928 | 0.59123018 | 0.712485386 | 0.90472652 | 0.455056335 | 0.567695199 | 0.778606905 | 0.50425192 | 0.742114102 | 0.304690378 | 0.71583678 | 0.769306079 | 0.618870815 | 0.572127353 | 0.435966337 | 0.529190837 |
| TCGA_TCGA-49-AARR | 0.462780655 | 0.389041996 | 0.479988653 | 0.676600256 | 0.601950672 | 0.594878679 | 0.522095966 | 0.605639014 | 0.508692094 | 0.721723861 | 0.761278149 | 0.446255629 | 0.545402816 | 0.749047704 | 0.438698463 | 0.71175492 | 0.263334271 | 0.751360293 | 0.62265175 | 0.531153772 | 0.526309983 | 0.372888068 | 0.496690338 |
| TCGA_TCGA-J2-8192 | 0.260674433 | 0.483863671 | 0.465942018 | 0.715473355 | 0.609357447 | 0.664494211 | 0.30395899 | 0.655055528 | 0.499564942 | 0.723881364 | 0.871573944 | 0.469728788 | 0.632704611 | 0.757798309 | 0.509111339 | 0.726618841 | 0.191158382 | 0.718158554 | 0.759632395 | 0.572435232 | 0.583010791 | 0.405751409 | 0.554551625 |
| TCGA_TCGA-64-5779 | 0.48476498 | 0.597893276 | 0.550331241 | 0.613688343 | 0.539982641 | 0.736884338 | 0.307804823 | 0.563184982 | 0.527904524 | 0.650511946 | 0.61955237 | 0.318261464 | 0.410054492 | 0.746401541 | 0.451314462 | 0.646968583 | 0.159359481 | 0.671102807 | 0.480729599 | 0.505255156 | 0.486728812 | 0.309896959 | 0.521408669 |
| TCGA_TCGA-62-A46O | 0.199644227 | 0.525771376 | 0.348347601 | 0.51773639 | 0.585945074 | 0.649866152 | 0.263379977 | 0.57327553 | 0.304475056 | 0.681487196 | 0.484035291 | 0.272927976 | 0.384831957 | 0.680417897 | 0.384393156 | 0.508008616 | 0.288866508 | 0.626589043 | 0.340786674 | 0.365376898 | 0.419529644 | 0.322125918 | 0.568053619 |
| TCGA_TCGA-50-8459 | 0.453512374 | 0.448297656 | 0.453109163 | 0.71589538 | 0.590381626 | 0.690192366 | 0.274633658 | 0.640397202 | 0.549051881 | 0.727411822 | 0.90170595 | 0.560992721 | 0.572611063 | 0.798792116 | 0.526334728 | 0.732250538 | 0.172293471 | 0.774428255 | 0.811965943 | 0.575651458 | 0.605376305 | 0.476322288 | 0.459746555 |
| TCGA_TCGA-86-A4JF | 0.286743098 | 0.564217498 | 0.529341143 | 0.660093271 | 0.656682721 | 0.663424348 | 0.372212113 | 0.625630207 | 0.514591706 | 0.706454661 | 0.76357065 | 0.397916575 | 0.529837119 | 0.76447407 | 0.438976019 | 0.631312123 | 0.318273901 | 0.691005702 | 0.625918269 | 0.544844941 | 0.488518114 | 0.347897707 | 0.57647541 |
| TCGA_TCGA-05-4249 | 0.300738032 | 0.352119614 | 0.461958668 | 0.679109007 | 0.634049342 | 0.64845976 | 0.329738056 | 0.557065216 | 0.491159204 | 0.701844775 | 0.735665518 | 0.43247618 | 0.593140363 | 0.780581178 | 0.42678576 | 0.683524764 | 0.205321696 | 0.687742924 | 0.653426671 | 0.565621836 | 0.493474481 | 0.405178567 | 0.459160697 |
| TCGA_TCGA-J2-A4AG | 0.466688717 | 0.572162436 | 0.558782493 | 0.698069282 | 0.58541296 | 0.682850275 | 0.443609604 | 0.597994779 | 0.572781883 | 0.696975926 | 0.777449925 | 0.376072959 | 0.550716251 | 0.781793614 | 0.488473224 | 0.697911898 | 0.169694856 | 0.688932427 | 0.672276799 | 0.586033567 | 0.548965434 | 0.42946975 | 0.594877644 |
| TCGA_TCGA-44-7671 | 0.225807245 | 0.358696679 | 0.410524443 | 0.610342994 | 0.561963253 | 0.602665396 | 0.426534356 | 0.58259389 | 0.342774819 | 0.663916314 | 0.591021273 | 0.369038279 | 0.517542617 | 0.755685949 | 0.408959272 | 0.601743109 | 0.218771004 | 0.633707536 | 0.5063643 | 0.540441012 | 0.463594147 | 0.390752476 | 0.55430393 |
| TCGA_TCGA-44-6774 | 0.284148498 | 0.444844304 | 0.430026758 | 0.6539957 | 0.638664407 | 0.66268824 | 0.390179934 | 0.65842605 | 0.451751744 | 0.734966928 | 0.786761175 | 0.467849347 | 0.567533029 | 0.755083871 | 0.540748973 | 0.704110952 | 0.149097881 | 0.736528013 | 0.724454359 | 0.582957356 | 0.565743237 | 0.335198746 | 0.482821505 |
| TCGA_TCGA-50-5066 | 0.368140114 | 0.610115626 | 0.59418291 | 0.676176698 | 0.591808691 | 0.632106309 | 0.307151745 | 0.663083106 | 0.52985231 | 0.714166224 | 0.818969122 | 0.415403192 | 0.569701009 | 0.741785142 | 0.511108838 | 0.683850307 | 0.212492876 | 0.705717668 | 0.698301958 | 0.582945311 | 0.545068694 | 0.346447934 | 0.546414195 |
| TCGA_TCGA-50-6594 | 0.180450587 | 0.485059035 | 0.444596536 | 0.638102634 | 0.663614225 | 0.66807212 | 0.194392228 | 0.637779872 | 0.438232553 | 0.731325696 | 0.621308394 | 0.316215034 | 0.341339385 | 0.719302734 | 0.511360775 | 0.648202315 | 0.203358955 | 0.673788332 | 0.521375937 | 0.544629123 | 0.530455421 | 0.382388774 | 0.370766254 |
| TCGA_TCGA-44-8119 | 0.294080157 | 0.543016386 | 0.511622022 | 0.669646173 | 0.652259391 | 0.768936588 | 0.35189245 | 0.644664093 | 0.453358485 | 0.698466908 | 0.784671608 | 0.392585244 | 0.465183605 | 0.747983084 | 0.468243985 | 0.665508929 | 0.243513051 | 0.695293245 | 0.674006653 | 0.517023005 | 0.524893978 | 0.348695795 | 0.530000818 |
| TCGA_TCGA-49-AAR2 | 0.388333322 | 0.507850341 | 0.541211735 | 0.619820976 | 0.551345895 | 0.720531387 | 0.280031277 | 0.525847845 | 0.473380728 | 0.684596792 | 0.727907318 | 0.400862962 | 0.362376078 | 0.798269846 | 0.378766072 | 0.589861732 | 0.205030748 | 0.651761567 | 0.433608938 | 0.506291706 | 0.474328428 | 0.375151872 | 0.439428912 |
| TCGA_TCGA-44-6779 | 0.275389759 | 0.61948694 | 0.674715797 | 0.711445811 | 0.643037647 | 0.692941396 | 0.302635589 | 0.682333853 | 0.519309005 | 0.715888005 | 0.85906057 | 0.492222888 | 0.504219531 | 0.791705005 | 0.554623229 | 0.722582937 | 0.332089269 | 0.69278407 | 0.752752316 | 0.585404036 | 0.576073403 | 0.437224321 | 0.54002295 |
| TCGA_TCGA-86-8669 | 0.289978938 | 0.504362233 | 0.52845158 | 0.611893433 | 0.612597649 | 0.695588481 | 0.337463623 | 0.543968696 | 0.474551322 | 0.729406466 | 0.688798519 | 0.380254262 | 0.428484098 | 0.751506018 | 0.414660558 | 0.606833062 | 0.167996141 | 0.650918516 | 0.584916911 | 0.564929265 | 0.474151059 | 0.442951949 | 0.529178766 |
| TCGA_TCGA-05-5715 | 0.233750262 | 0.373447357 | 0.443313142 | 0.659081153 | 0.641016005 | 0.657298071 | 0.281784726 | 0.6321623 | 0.422056065 | 0.74564865 | 0.752054218 | 0.425013234 | 0.552639212 | 0.780469452 | 0.487990662 | 0.651942615 | 0.167526225 | 0.7038848 | 0.684174595 | 0.576153559 | 0.523905071 | 0.43884034 | 0.481615384 |
| TCGA_TCGA-69-A59K | 0.352829615 | 0.493943904 | 0.468951012 | 0.640383732 | 0.560344034 | 0.710832622 | 0.342773307 | 0.601818275 | 0.556191001 | 0.686667314 | 0.70038267 | 0.382532958 | 0.38880431 | 0.767082339 | 0.46748976 | 0.608904597 | 0.175309958 | 0.676832263 | 0.620621791 | 0.521480409 | 0.492284249 | 0.390098015 | 0.412326743 |
| TCGA_TCGA-55-A491 | 0.376619071 | 0.448019332 | 0.501901033 | 0.605215512 | 0.652030143 | 0.703737586 | 0.293854124 | 0.581220407 | 0.509246333 | 0.742489693 | 0.76899817 | 0.393596596 | 0.506582708 | 0.757139662 | 0.466229655 | 0.63250219 | 0.166755798 | 0.718474822 | 0.657722771 | 0.57168319 | 0.554849666 | 0.40080224 | 0.457511717 |
| TCGA_TCGA-71-8520 | 0.155002915 | 0.43204986 | 0.394719522 | 0.625718249 | 0.601198484 | 0.646994226 | 0.257591293 | 0.5795138 | 0.391618316 | 0.682346345 | 0.576868134 | 0.359894685 | 0.498927794 | 0.73991534 | 0.439838714 | 0.66049328 | 0.180364455 | 0.678384723 | 0.556679649 | 0.566580577 | 0.505291109 | 0.463727263 | 0.574822291 |
| TCGA_TCGA-91-8496 | 0.209682043 | 0.391434305 | 0.541371443 | 0.747030106 | 0.60891155 | 0.657925512 | 0.350937891 | 0.685568836 | 0.488603077 | 0.729068312 | 0.864110789 | 0.504921577 | 0.642258393 | 0.81203627 | 0.468153132 | 0.677894945 | 0.20735071 | 0.741587936 | 0.671179832 | 0.616985698 | 0.550896418 | 0.451671279 | 0.479344774 |
| TCGA_TCGA-69-8254 | 0.371495572 | 0.46475142 | 0.485994541 | 0.670797366 | 0.596230028 | 0.701381671 | 0.421980749 | 0.578362405 | 0.491604757 | 0.717077035 | 0.717259522 | 0.404718259 | 0.491213815 | 0.7745763 | 0.466561144 | 0.635118162 | 0.241843763 | 0.661011626 | 0.610578 | 0.529149966 | 0.533915521 | 0.463695559 | 0.56271605 |
| TCGA_TCGA-95-A4VP | 0.459853691 | 0.563648142 | 0.591214956 | 0.669578591 | 0.585304668 | 0.737604858 | 0.4173241 | 0.600329289 | 0.513205973 | 0.70116015 | 0.757508901 | 0.445311347 | 0.463253162 | 0.768414979 | 0.424349711 | 0.71118362 | 0.33157907 | 0.692712948 | 0.573843013 | 0.54500748 | 0.511141346 | 0.375753543 | 0.538786791 |
| TCGA_TCGA-95-8039 | 0.296406631 | 0.490102974 | 0.563088684 | 0.679031058 | 0.623778613 | 0.674146513 | 0.325764894 | 0.646740573 | 0.470031086 | 0.679314501 | 0.788460093 | 0.426351996 | 0.518789612 | 0.774727357 | 0.441966546 | 0.710264025 | 0.187046005 | 0.682480365 | 0.689707866 | 0.588793772 | 0.544620012 | 0.469499252 | 0.512731958 |
| TCGA_TCGA-86-8076 | 0.549501221 | 0.524232644 | 0.643399392 | 0.682941104 | 0.620122107 | 0.633982355 | 0.377917265 | 0.592380348 | 0.547145589 | 0.677817046 | 0.828097213 | 0.455172553 | 0.523640159 | 0.801237376 | 0.443862472 | 0.701328379 | 0.268172305 | 0.700428689 | 0.660218374 | 0.547631535 | 0.558056253 | 0.383313407 | 0.511514439 |
| TCGA_TCGA-44-2659 | 0.336643233 | 0.46023479 | 0.549215112 | 0.647264814 | 0.613625207 | 0.68254477 | 0.289516684 | 0.588479018 | 0.529364065 | 0.662516386 | 0.733423853 | 0.395999547 | 0.510069425 | 0.765519048 | 0.471836305 | 0.665661022 | 0.155136362 | 0.665184469 | 0.625500493 | 0.579140995 | 0.52723553 | 0.436261211 | 0.511875124 |
| TCGA_TCGA-55-8203 | 0.484854468 | 0.541576177 | 0.575003695 | 0.661259474 | 0.578545434 | 0.730104466 | 0.206239341 | 0.579728572 | 0.565535829 | 0.680922275 | 0.749026908 | 0.383629231 | 0.441126734 | 0.772522945 | 0.431240755 | 0.665797068 | 0.230818926 | 0.684620437 | 0.613808812 | 0.516073147 | 0.493141088 | 0.364116812 | 0.523749132 |
| TCGA_TCGA-MP-A5C7 | 0.148852438 | 0.386795133 | 0.414483947 | 0.539941778 | 0.515985867 | 0.646645524 | 0.343402784 | 0.487662404 | 0.333463989 | 0.609545337 | 0.458655456 | 0.316139639 | 0.401016024 | 0.719026029 | 0.373850524 | 0.584934941 | 0.191318054 | 0.653040424 | 0.415699785 | 0.482377556 | 0.422364515 | 0.37811074 | 0.501542507 |
| TCGA_TCGA-78-7220 | 0.144446548 | 0.540616691 | 0.429411226 | 0.579256832 | 0.562632507 | 0.665473539 | 0.208394535 | 0.554372543 | 0.315656234 | 0.662096197 | 0.572458827 | 0.383179815 | 0.418649827 | 0.710895102 | 0.43056506 | 0.570003717 | 0.312125403 | 0.657589577 | 0.484794325 | 0.494799961 | 0.46537697 | 0.408390975 | 0.571361357 |
| TCGA_TCGA-44-3917 | 0.299731988 | 0.65803681 | 0.466332004 | 0.605386606 | 0.562264525 | 0.616136957 | 0.464586564 | 0.589362119 | 0.535169131 | 0.601792062 | 0.634293744 | 0.36460463 | 0.409170555 | 0.696588614 | 0.432359671 | 0.601470955 | 0.202424378 | 0.565762212 | 0.692878859 | 0.554282979 | 0.496592336 | 0.36646175 | 0.630318955 |
| TCGA_TCGA-78-7153 | 0.286028054 | 0.411845045 | 0.528433798 | 0.573252028 | 0.651132827 | 0.677908125 | 0.273597417 | 0.531621157 | 0.387994504 | 0.63473354 | 0.558759352 | 0.371987729 | 0.376141739 | 0.734951275 | 0.409038577 | 0.579681546 | 0.114493434 | 0.617632818 | 0.435572041 | 0.480086158 | 0.453324733 | 0.410485032 | 0.540631556 |
| TCGA_TCGA-55-7910 | 0.274384656 | 0.585576324 | 0.424216893 | 0.604334523 | 0.570452366 | 0.722918099 | 0.456806455 | 0.550389776 | 0.435424861 | 0.751741325 | 0.671866008 | 0.356266221 | 0.436348901 | 0.730717284 | 0.422390638 | 0.63562959 | 0.2915853 | 0.677980738 | 0.487000953 | 0.469340796 | 0.456424459 | 0.363328294 | 0.579184846 |
| TCGA_TCGA-64-5778 | 0.492892159 | 0.613271278 | 0.750637745 | 0.685910883 | 0.632327059 | 0.667179234 | 0.293590593 | 0.625857021 | 0.547514574 | 0.691874962 | 0.809934522 | 0.415034631 | 0.462279493 | 0.739280028 | 0.430961704 | 0.672434908 | 0.22955093 | 0.67334922 | 0.591962985 | 0.582152452 | 0.542310409 | 0.451998669 | 0.516038916 |
| TCGA_TCGA-55-7815 | 0.297005657 | 0.678722918 | 0.553440667 | 0.67084826 | 0.6875362 | 0.632557406 | 0.260604328 | 0.648279003 | 0.5764056 | 0.733024361 | 0.784007451 | 0.449070941 | 0.520706259 | 0.722018307 | 0.50951675 | 0.670510805 | 0.329169193 | 0.691245529 | 0.728594288 | 0.537234382 | 0.530002219 | 0.395685678 | 0.652554331 |
| TCGA_TCGA-55-6985 | 0.344696137 | 0.53823933 | 0.545812332 | 0.684228501 | 0.611076336 | 0.657117418 | 0.438946252 | 0.595577505 | 0.513351737 | 0.710823778 | 0.813665346 | 0.401766184 | 0.566936908 | 0.756768844 | 0.485023325 | 0.67675413 | 0.226119052 | 0.68414154 | 0.674651125 | 0.550350726 | 0.561022045 | 0.408791179 | 0.584931678 |
| TCGA_TCGA-69-7761 | 0.458727357 | 0.657144252 | 0.583649697 | 0.726367984 | 0.599338191 | 0.679309477 | 0.364232811 | 0.578360284 | 0.636085142 | 0.662600163 | 0.810160472 | 0.460122853 | 0.536304284 | 0.781428061 | 0.533731634 | 0.710147019 | 0.196735203 | 0.71489234 | 0.689128568 | 0.5504851 | 0.581294051 | 0.535476457 | 0.548862218 |
| TCGA_TCGA-MN-A4N4 | 0.257422146 | 0.398317692 | 0.431060265 | 0.666268909 | 0.623097385 | 0.645071984 | 0.224638998 | 0.58940568 | 0.387211729 | 0.656208585 | 0.767492212 | 0.333033956 | 0.509776389 | 0.704482372 | 0.457072754 | 0.656950104 | 0.2133182 | 0.667109879 | 0.56730363 | 0.521513158 | 0.523815707 | 0.356368574 | 0.363668655 |
| TCGA_TCGA-55-6972 | 0.1535588 | 0.311099355 | 0.44937093 | 0.535074128 | 0.547106498 | 0.620717345 | 0.359132926 | 0.51395427 | 0.245887268 | 0.62804693 | 0.38600212 | 0.322607757 | 0.35984783 | 0.72130894 | 0.312846025 | 0.483556913 | 0.212716852 | 0.600985852 | 0.330822269 | 0.442404812 | 0.39211628 | 0.420169648 | 0.384325723 |
| TCGA_TCGA-97-A4M1 | 0.213585871 | 0.247811785 | 0.458351344 | 0.670791317 | 0.5581998 | 0.645133732 | 0.386377357 | 0.626927602 | 0.429609316 | 0.715238305 | 0.743559138 | 0.48194308 | 0.617948555 | 0.782896652 | 0.43167732 | 0.667326236 | 0.16018595 | 0.717422289 | 0.614746924 | 0.588707816 | 0.511900009 | 0.360962949 | 0.461350825 |
| TCGA_TCGA-83-5908 | 0.333773481 | 0.751347027 | 0.719003578 | 0.692361086 | 0.639144738 | 0.687232964 | 0.341446606 | 0.628071811 | 0.571297188 | 0.678044839 | 0.796094241 | 0.3840137 | 0.482946136 | 0.758976614 | 0.535354902 | 0.731091938 | 0.143854949 | 0.682295403 | 0.660801096 | 0.575549183 | 0.575347637 | 0.410728612 | 0.537701306 |
| TCGA_TCGA-97-8179 | 0.173399319 | 0.369956353 | 0.445728327 | 0.665737495 | 0.514304034 | 0.599654918 | 0.350508632 | 0.564678328 | 0.379761224 | 0.662410456 | 0.618748631 | 0.402398909 | 0.443654569 | 0.740247835 | 0.430655211 | 0.626059651 | 0.180122208 | 0.642492777 | 0.546029828 | 0.521484945 | 0.461383363 | 0.37894863 | 0.531092533 |
| TCGA_TCGA-64-1680 | 0.168675604 | 0.395142116 | 0.442726959 | 0.689559077 | 0.618775192 | 0.628873325 | 0.266029881 | 0.549221036 | 0.369542332 | 0.745311614 | 0.616982547 | 0.368224406 | 0.545711588 | 0.705303141 | 0.371335457 | 0.638793895 | 0.204691211 | 0.672488633 | 0.508754629 | 0.48452115 | 0.468810459 | 0.429197824 | 0.494826364 |
| TCGA_TCGA-73-4670 | 0.217636698 | 0.573243026 | 0.432110586 | 0.656793757 | 0.6626634 | 0.694506837 | 0.25737041 | 0.610138207 | 0.380051589 | 0.671666123 | 0.722920749 | 0.404529858 | 0.479668387 | 0.730382207 | 0.439292248 | 0.626599041 | 0.403056325 | 0.641078726 | 0.569318559 | 0.488508975 | 0.498600972 | 0.392020051 | 0.470528357 |
| TCGA_TCGA-97-8174 | 0.382776935 | 0.40719254 | 0.451596967 | 0.653498546 | 0.503252218 | 0.59490523 | 0.365902082 | 0.597283862 | 0.46130915 | 0.642505316 | 0.731301335 | 0.451657109 | 0.533572498 | 0.735524847 | 0.450312948 | 0.65629134 | 0.121763954 | 0.685329531 | 0.610074728 | 0.574658301 | 0.514872968 | 0.462503757 | 0.538467589 |
| TCGA_TCGA-44-6777 | 0.416464835 | 0.525544375 | 0.490373223 | 0.72222136 | 0.608731482 | 0.675084536 | 0.316041265 | 0.739709393 | 0.607397465 | 0.759053237 | 0.947564323 | 0.542225815 | 0.627350326 | 0.795713871 | 0.576932439 | 0.734038302 | 0.223587569 | 0.731921378 | 0.857722627 | 0.652924874 | 0.60512283 | 0.4306826 | 0.514669125 |
| TCGA_TCGA-62-A46P | 0.192490807 | 0.352131374 | 0.503595118 | 0.595607076 | 0.625399426 | 0.738027745 | 0.347592483 | 0.551431876 | 0.354901165 | 0.658038349 | 0.604585046 | 0.366577548 | 0.379293974 | 0.754107573 | 0.381224239 | 0.621622614 | 0.28481524 | 0.656796971 | 0.478304111 | 0.524905929 | 0.433149756 | 0.374744117 | 0.51582999 |
| TCGA_TCGA-55-8089 | 0.455423137 | 0.673651261 | 0.748820428 | 0.715266862 | 0.64679447 | 0.681418815 | 0.29793384 | 0.684619098 | 0.604168558 | 0.711531279 | 0.917866991 | 0.50544911 | 0.511145875 | 0.800661527 | 0.587907229 | 0.739908141 | 0.250501584 | 0.724516432 | 0.791891969 | 0.592885759 | 0.616420282 | 0.419203803 | 0.564353775 |
| TCGA_TCGA-MN-A4N5 | 0.269423745 | 0.482893027 | 0.549976009 | 0.671986495 | 0.641682214 | 0.67229519 | 0.247092498 | 0.590169624 | 0.420015091 | 0.686799265 | 0.744578553 | 0.368908487 | 0.361526209 | 0.714075014 | 0.429156036 | 0.647727228 | 0.21202626 | 0.648136074 | 0.504098451 | 0.54926465 | 0.517330779 | 0.385553257 | 0.461211262 |
| TCGA_TCGA-49-AAR9 | 0.095137056 | 0.544073462 | 0.414674162 | 0.604660045 | 0.615429042 | 0.738748306 | 0.208361333 | 0.539904897 | 0.309966544 | 0.688379281 | 0.522202885 | 0.304028399 | 0.396968199 | 0.720629042 | 0.448176683 | 0.622694882 | 0.067713071 | 0.659664986 | 0.478542707 | 0.467123614 | 0.502505138 | 0.422667905 | 0.471834837 |
| TCGA_TCGA-86-8671 | 0.687074829 | 0.586055613 | 0.740536584 | 0.715095055 | 0.62784997 | 0.694549853 | 0.306596219 | 0.632137475 | 0.734692248 | 0.684487591 | 0.938052895 | 0.505251002 | 0.557040359 | 0.822826453 | 0.542632325 | 0.741488371 | 0.181418798 | 0.743875651 | 0.786503195 | 0.665986333 | 0.639184504 | 0.432681928 | 0.483820841 |
| TCGA_TCGA-49-4507 | 0.34288829 | 0.68073938 | 0.749437504 | 0.706329064 | 0.699162318 | 0.691987274 | 0.238707308 | 0.661642453 | 0.441681403 | 0.640887746 | 0.841751445 | 0.475173571 | 0.40126111 | 0.736432523 | 0.528740353 | 0.652655259 | 0.329849648 | 0.652947362 | 0.589432694 | 0.523643555 | 0.552503058 | 0.449382125 | 0.518626963 |
| TCGA_TCGA-62-A46V | 0.154494868 | 0.376113716 | 0.406824215 | 0.677715382 | 0.633369749 | 0.696420348 | 0.267926392 | 0.554111722 | 0.380562949 | 0.713156601 | 0.660900896 | 0.394369969 | 0.470547075 | 0.748840296 | 0.426944474 | 0.660469773 | 0.210165526 | 0.683354978 | 0.585747912 | 0.544227163 | 0.463744437 | 0.459340953 | 0.415781504 |
| TCGA_TCGA-05-4389 | 0.241461735 | 0.544690418 | 0.65863756 | 0.675437082 | 0.6404696 | 0.722697385 | 0.214785049 | 0.671408713 | 0.479055082 | 0.750866991 | 0.763545537 | 0.450935887 | 0.511775171 | 0.772765825 | 0.485002001 | 0.626677932 | 0.163725912 | 0.678063647 | 0.677518311 | 0.580682638 | 0.536694195 | 0.409723765 | 0.490755439 |
| TCGA_TCGA-50-6592 | 0.271299149 | 0.636906558 | 0.59304556 | 0.693431682 | 0.61737933 | 0.694659535 | 0.265665135 | 0.624981157 | 0.500585081 | 0.683379464 | 0.892660988 | 0.413717526 | 0.496730419 | 0.727141557 | 0.485775328 | 0.674269118 | 0.207234295 | 0.691761376 | 0.709957067 | 0.565358975 | 0.565055747 | 0.504396705 | 0.526223406 |
| TCGA_TCGA-91-6831 | 0.275625767 | 0.584233161 | 0.464998233 | 0.644552412 | 0.63560497 | 0.712469197 | 0.286173886 | 0.616796697 | 0.482750559 | 0.660300068 | 0.757589661 | 0.432248051 | 0.485435492 | 0.733332383 | 0.473321777 | 0.682173168 | 0.240196378 | 0.678372055 | 0.709733616 | 0.514123539 | 0.492496009 | 0.328193686 | 0.556447102 |
| TCGA_TCGA-55-6987 | 0.619425833 | 0.670180209 | 0.760753896 | 0.739678231 | 0.630575701 | 0.621066137 | 0.395997655 | 0.661798806 | 0.673252214 | 0.729060465 | 0.957247338 | 0.508084947 | 0.583602952 | 0.778702137 | 0.520829869 | 0.728851695 | 0.221873315 | 0.739253445 | 0.790713324 | 0.597046455 | 0.622054135 | 0.38541882 | 0.597995308 |
| TCGA_TCGA-49-4510 | 0.252452203 | 0.309157202 | 0.470407579 | 0.648111709 | 0.598774474 | 0.747093259 | 0.384807327 | 0.579214272 | 0.3908998 | 0.633772007 | 0.655155722 | 0.382904212 | 0.507498867 | 0.738790551 | 0.418561275 | 0.622257312 | 0.291219529 | 0.670410961 | 0.493628533 | 0.53341192 | 0.466779527 | 0.399369818 | 0.470571729 |
| TCGA_TCGA-86-8672 | 0.344533521 | 0.550072159 | 0.630878238 | 0.706002667 | 0.609507078 | 0.678604743 | 0.235524809 | 0.638278009 | 0.490847662 | 0.697281687 | 0.870766289 | 0.381534725 | 0.476378316 | 0.740224329 | 0.498150107 | 0.657369422 | 0.284343787 | 0.670417917 | 0.699505331 | 0.557316118 | 0.524203029 | 0.390619002 | 0.477980907 |
| TCGA_TCGA-44-2668 | 0.323185012 | 0.747583401 | 0.510684499 | 0.74815038 | 0.614124264 | 0.649455182 | 0.365927394 | 0.673251584 | 0.553049545 | 0.695342176 | 0.86270241 | 0.491724033 | 0.579843902 | 0.743621174 | 0.576920745 | 0.72301331 | 0.371452288 | 0.697996582 | 0.772260769 | 0.595188515 | 0.601536034 | 0.540449739 | 0.567913009 |
| TCGA_TCGA-93-A4JQ | 0.612534443 | 0.585940181 | 0.612673316 | 0.646878588 | 0.561860105 | 0.699114422 | 0.349326854 | 0.606472963 | 0.675659485 | 0.682040786 | 0.828981321 | 0.385879333 | 0.461226159 | 0.748308365 | 0.483820067 | 0.672358968 | 0.133298368 | 0.674917088 | 0.654678788 | 0.571637869 | 0.599371876 | 0.428990648 | 0.508949008 |
| TCGA_TCGA-97-8177 | 0.370950083 | 0.460279903 | 0.54655691 | 0.691059924 | 0.635514016 | 0.697202269 | 0.41090292 | 0.676375255 | 0.500220997 | 0.759227629 | 0.903460891 | 0.470293291 | 0.659927481 | 0.768079916 | 0.478186912 | 0.710754341 | 0.209259036 | 0.746321239 | 0.745220078 | 0.60268222 | 0.540746941 | 0.435647357 | 0.486016567 |
| TCGA_TCGA-05-4396 | 0.180509587 | 0.388251073 | 0.388071861 | 0.604711421 | 0.562069207 | 0.713566615 | 0.257115894 | 0.506025809 | 0.384084666 | 0.630834051 | 0.53294029 | 0.319313785 | 0.376808341 | 0.738217836 | 0.388502924 | 0.632804839 | 0.290509571 | 0.657675209 | 0.456141937 | 0.498172111 | 0.483997287 | 0.364728693 | 0.573921279 |
| TCGA_TCGA-55-6978 | 0.347235727 | 0.678653648 | 0.701573304 | 0.687705452 | 0.621227674 | 0.65258749 | 0.335006429 | 0.682303407 | 0.546863783 | 0.662516086 | 0.841948057 | 0.470511353 | 0.466869948 | 0.7570833 | 0.578385317 | 0.720728877 | 0.219483934 | 0.690842139 | 0.807600446 | 0.601298232 | 0.606731575 | 0.408565538 | 0.574035218 |
| TCGA_TCGA-69-8453 | 0.36496196 | 0.523343189 | 0.578133308 | 0.702684163 | 0.603982399 | 0.691323455 | 0.471743364 | 0.682641769 | 0.548602349 | 0.760072464 | 0.910444262 | 0.504772472 | 0.622584602 | 0.801562732 | 0.534467427 | 0.722745661 | 0.222585511 | 0.770438817 | 0.730769652 | 0.619060885 | 0.585291808 | 0.464744856 | 0.551872049 |
| TCGA_TCGA-50-7109 | 0.303297367 | 0.524105159 | 0.589828324 | 0.657397274 | 0.550353107 | 0.695157898 | 0.292892287 | 0.555694064 | 0.506285418 | 0.631376093 | 0.685586185 | 0.37428821 | 0.44106076 | 0.757751829 | 0.413169668 | 0.635830271 | 0.229060676 | 0.695024178 | 0.547865807 | 0.510094799 | 0.488530704 | 0.3842708 | 0.563971408 |
| TCGA_TCGA-49-4501 | 0.380126625 | 0.468371922 | 0.556881507 | 0.663325311 | 0.59548518 | 0.681872453 | 0.354500693 | 0.642806862 | 0.485707673 | 0.674855898 | 0.744114326 | 0.419654027 | 0.538474403 | 0.781112207 | 0.458575302 | 0.644455807 | 0.164447605 | 0.720031249 | 0.605563377 | 0.578253648 | 0.556602978 | 0.401067101 | 0.485024245 |
| TCGA_TCGA-50-5049 | 0.586344824 | 0.64705436 | 0.742736362 | 0.693954791 | 0.627840996 | 0.630251763 | 0.424725333 | 0.697531677 | 0.67526658 | 0.716328759 | 0.938547896 | 0.498518626 | 0.616520618 | 0.814198855 | 0.54051164 | 0.727449244 | 0.128216918 | 0.721670198 | 0.830416279 | 0.661837046 | 0.641855949 | 0.396861303 | 0.514288636 |
| TCGA_TCGA-78-8660 | 0.323244677 | 0.623816034 | 0.674591541 | 0.6751428 | 0.620062331 | 0.648317609 | 0.340821917 | 0.64195234 | 0.496126178 | 0.702784255 | 0.830905536 | 0.462819898 | 0.545798386 | 0.761708972 | 0.503560309 | 0.654926442 | 0.182276345 | 0.682658619 | 0.720094779 | 0.595826294 | 0.546576215 | 0.387635843 | 0.528724567 |
| TCGA_TCGA-91-6828 | 0.311720869 | 0.572375317 | 0.543842105 | 0.73736099 | 0.619350053 | 0.733835959 | 0.373316768 | 0.636964081 | 0.527818474 | 0.695772404 | 0.841549512 | 0.461535816 | 0.526008022 | 0.773735174 | 0.494801377 | 0.695969005 | 0.298075111 | 0.700355041 | 0.746070067 | 0.601823692 | 0.510340769 | 0.38573705 | 0.566338487 |
| TCGA_TCGA-50-6590 | 0.451737893 | 0.65034713 | 0.578592797 | 0.691035195 | 0.63249845 | 0.688434142 | 0.256102187 | 0.665918789 | 0.599207751 | 0.630358338 | 0.895585599 | 0.462911122 | 0.415037727 | 0.76730026 | 0.5766829 | 0.684339556 | 0.172287329 | 0.696702197 | 0.804188015 | 0.568949551 | 0.576100604 | 0.371517215 | 0.449061014 |
| TCGA_TCGA-97-8176 | 0.23299787 | 0.520569433 | 0.445935973 | 0.633172826 | 0.589391968 | 0.683823003 | 0.224596856 | 0.633316816 | 0.377647524 | 0.720245611 | 0.73546006 | 0.438883125 | 0.383087025 | 0.748967948 | 0.437956196 | 0.603286559 | 0.285026179 | 0.676273921 | 0.60847331 | 0.508470221 | 0.48441967 | 0.392916887 | 0.535066361 |
| TCGA_TCGA-50-5935 | 0.205912448 | 0.468793971 | 0.50241443 | 0.665795083 | 0.617375369 | 0.664996273 | 0.331010443 | 0.600679825 | 0.442325104 | 0.698330564 | 0.781771441 | 0.396078431 | 0.534317128 | 0.754818056 | 0.440431331 | 0.637627545 | 0.190811003 | 0.675606391 | 0.616671155 | 0.554164604 | 0.523294677 | 0.428601642 | 0.497094741 |
| TCGA_TCGA-55-7281 | 0.252734002 | 0.48305558 | 0.510920396 | 0.727806753 | 0.59814193 | 0.681201483 | 0.382531479 | 0.637777835 | 0.474216171 | 0.75935949 | 0.831307057 | 0.47962143 | 0.593368062 | 0.765326827 | 0.500755728 | 0.737222011 | 0.189936157 | 0.705106035 | 0.721600929 | 0.588376719 | 0.548994865 | 0.464733434 | 0.512612234 |
| TCGA_TCGA-49-4488 | 0.30497749 | 0.562853242 | 0.550927471 | 0.727582645 | 0.681616508 | 0.736703131 | 0.245697612 | 0.628187293 | 0.477148004 | 0.707693327 | 0.728142988 | 0.379167476 | 0.501798397 | 0.765192714 | 0.465842057 | 0.644152259 | 0.198409049 | 0.638505391 | 0.660981692 | 0.591426905 | 0.509644885 | 0.419396394 | 0.490497774 |
| TCGA_TCGA-NJ-A55A | 0.537417385 | 0.483624518 | 0.540250515 | 0.664327129 | 0.596159103 | 0.629998009 | 0.420687857 | 0.554671057 | 0.575869748 | 0.689012022 | 0.749320684 | 0.398851774 | 0.574882262 | 0.757054032 | 0.389349261 | 0.687756312 | 0.250706765 | 0.712156164 | 0.588256693 | 0.538464728 | 0.51170642 | 0.389890716 | 0.543119717 |
| TCGA_TCGA-95-8494 | 0.186323813 | 0.538568983 | 0.471324801 | 0.680136996 | 0.603701393 | 0.687272961 | 0.351762634 | 0.621029817 | 0.443805367 | 0.688895154 | 0.768213728 | 0.371241688 | 0.506207084 | 0.754909695 | 0.480253285 | 0.631576147 | 0.202586986 | 0.660872032 | 0.666580205 | 0.535808254 | 0.492576546 | 0.389641099 | 0.451678273 |
| TCGA_TCGA-44-2657 | 0.742500588 | 0.571569509 | 0.701333622 | 0.711933797 | 0.608148142 | 0.651607266 | 0.394026625 | 0.655406826 | 0.755667538 | 0.67723221 | 0.888846862 | 0.464441764 | 0.63074496 | 0.806911648 | 0.521616906 | 0.684631566 | 0.131717624 | 0.725708088 | 0.683775609 | 0.642495467 | 0.597722711 | 0.373680724 | 0.476930601 |
| TCGA_TCGA-78-7158 | 0.141165997 | 0.34985781 | 0.421188737 | 0.602598749 | 0.568330773 | 0.604223766 | 0.239746825 | 0.54263691 | 0.410075725 | 0.651126557 | 0.619668573 | 0.360091878 | 0.435455188 | 0.738644578 | 0.398652559 | 0.619885345 | 0.13837458 | 0.641547513 | 0.468711279 | 0.52037786 | 0.454083766 | 0.444417331 | 0.464653826 |
| TCGA_TCGA-93-7347 | 0.465868251 | 0.540498502 | 0.656072822 | 0.686989392 | 0.643102115 | 0.685163202 | 0.302421664 | 0.617442856 | 0.55367189 | 0.743525188 | 0.862346869 | 0.475428861 | 0.582819579 | 0.805470748 | 0.503654318 | 0.67240094 | 0.187027885 | 0.715090476 | 0.727951678 | 0.611205377 | 0.603495789 | 0.468733998 | 0.509927555 |
| TCGA_TCGA-38-4630 | 0.157152046 | 0.562543375 | 0.447949668 | 0.58755409 | 0.606046296 | 0.674426396 | 0.285870615 | 0.582054588 | 0.333552788 | 0.604100998 | 0.532407963 | 0.337682033 | 0.367048804 | 0.693374639 | 0.4204263 | 0.604628077 | 0 | 0.656154601 | 0.58279192 | 0.477359496 | 0.434068222 | 0.314096879 | 0.439978049 |
| TCGA_TCGA-86-7953 | 0.291174652 | 0.644479092 | 0.505760494 | 0.656547188 | 0.655708424 | 0.6532742 | 0.38451809 | 0.605543144 | 0.476713041 | 0.764593753 | 0.823159832 | 0.423706894 | 0.457311504 | 0.751681678 | 0.467856126 | 0.680657008 | 0.117776832 | 0.687654572 | 0.665988247 | 0.548223941 | 0.526260518 | 0.407931663 | 0.553566151 |
| TCGA_TCGA-50-5072 | 0.167814504 | 0.596601419 | 0.413519646 | 0.612079357 | 0.602375659 | 0.690740873 | 0.285913043 | 0.617272434 | 0.380983528 | 0.687083468 | 0.63838957 | 0.368987458 | 0.46458505 | 0.736634864 | 0.469595139 | 0.60526048 | 0.296084047 | 0.650771311 | 0.552198599 | 0.486982332 | 0.514367719 | 0.372865546 | 0.603997749 |
| TCGA_TCGA-62-A46S | 0.246253164 | 0.387251222 | 0.495951998 | 0.645542639 | 0.591925842 | 0.718355113 | 0.423614904 | 0.53931557 | 0.429925528 | 0.678714755 | 0.718773523 | 0.378775284 | 0.472322928 | 0.748650962 | 0.431837712 | 0.576720468 | 0.245111141 | 0.669872628 | 0.571290613 | 0.556746798 | 0.467488151 | 0.485310377 | 0.574034725 |
| TCGA_TCGA-53-A4EZ | 0.239892662 | 0.506353972 | 0.457214437 | 0.600034994 | 0.613169309 | 0.778985997 | 0.24283375 | 0.581376967 | 0.37804326 | 0.715678891 | 0.564543622 | 0.310051585 | 0.346752283 | 0.722324446 | 0.412735168 | 0.55302725 | 0.191811949 | 0.641744336 | 0.484993106 | 0.520256602 | 0.401190259 | 0.334044043 | 0.498939893 |
| TCGA_TCGA-69-7973 | 0.253047234 | 0.539498936 | 0.432192012 | 0.600554912 | 0.5763103 | 0.694418303 | 0.309995552 | 0.575809863 | 0.388011625 | 0.689218688 | 0.6076827 | 0.400225584 | 0.489576829 | 0.712750977 | 0.372917693 | 0.599867696 | 0.222025935 | 0.649326251 | 0.489987474 | 0.495570287 | 0.490826572 | 0.317280088 | 0.5915929 |
| TCGA_TCGA-55-8204 | 0.348251161 | 0.494440315 | 0.473777488 | 0.642360225 | 0.702229456 | 0.637371705 | 0.327653574 | 0.548888915 | 0.487829249 | 0.698040204 | 0.702989359 | 0.377906582 | 0.46276572 | 0.757978185 | 0.506180825 | 0.636208419 | 0.216061246 | 0.722006647 | 0.610341495 | 0.500115858 | 0.498632362 | 0.409228233 | 0.529742222 |
| TCGA_TCGA-35-5375 | 0.271379973 | 0.600394665 | 0.633752365 | 0.638239996 | 0.686973698 | 0.609999905 | 0.240615511 | 0.639488831 | 0.475324785 | 0.708645162 | 0.760876193 | 0.387318922 | 0.414971555 | 0.678224395 | 0.432984205 | 0.645117471 | 0.135749143 | 0.639875407 | 0.670447775 | 0.572144626 | 0.507322196 | 0.361185425 | 0.427031436 |
| TCGA_TCGA-78-7539 | 0.288900929 | 0.485778786 | 0.625333879 | 0.688803148 | 0.633989837 | 0.696470274 | 0.373218481 | 0.603333663 | 0.488959556 | 0.698972977 | 0.786368395 | 0.451903466 | 0.459154334 | 0.77432573 | 0.439397897 | 0.670398558 | 0.198302046 | 0.708989586 | 0.643792932 | 0.547247573 | 0.513690612 | 0.431852762 | 0.467673302 |
| TCGA_TCGA-95-A4VK | 0.458063204 | 0.49110112 | 0.54041919 | 0.630895045 | 0.550816536 | 0.679240697 | 0.313347146 | 0.528760443 | 0.499154442 | 0.611850477 | 0.702520093 | 0.390183583 | 0.383984254 | 0.744436027 | 0.371027861 | 0.600397889 | 0.316691937 | 0.652668059 | 0.497353785 | 0.495029823 | 0.519017536 | 0.374786478 | 0.493691314 |
| TCGA_TCGA-50-5055 | 0.802992442 | 0.632136188 | 0.73463145 | 0.722395877 | 0.665929293 | 0.729625449 | 0.469558383 | 0.63657835 | 0.8195217 | 0.698640408 | 0.89365515 | 0.468299424 | 0.567551855 | 0.849687838 | 0.525918983 | 0.729614636 | 0.142093799 | 0.727412675 | 0.741515713 | 0.632477962 | 0.649258768 | 0.433993799 | 0.50246636 |
| TCGA_TCGA-44-7667 | 0.151164921 | 0.562233655 | 0.459482913 | 0.561434866 | 0.576766715 | 0.61799745 | 0.22971305 | 0.633878604 | 0.289957983 | 0.615174505 | 0.640825416 | 0.339265939 | 0.377463293 | 0.67643084 | 0.517202651 | 0.598245098 | 0.114152587 | 0.611310587 | 0.504794391 | 0.448115305 | 0.43918776 | 0.28215837 | 0.479211154 |
| TCGA_TCGA-MP-A4TI | 0.415145437 | 0.673320928 | 0.707570414 | 0.722669446 | 0.645740172 | 0.690412173 | 0.358892075 | 0.71753067 | 0.568100673 | 0.697240692 | 0.97566676 | 0.52996402 | 0.545187568 | 0.768719005 | 0.580419629 | 0.744149833 | 0.256604195 | 0.732449855 | 0.843267863 | 0.626344031 | 0.619725989 | 0.421989079 | 0.531452489 |
| TCGA_TCGA-55-6979 | 0.435133284 | 0.605869059 | 0.608589291 | 0.714787285 | 0.619388243 | 0.683668892 | 0.329909836 | 0.693479776 | 0.56942059 | 0.728860122 | 0.952016867 | 0.488406307 | 0.543295555 | 0.765205784 | 0.522720207 | 0.734994267 | 0.155473399 | 0.704363839 | 0.792961443 | 0.621816908 | 0.606423818 | 0.434700449 | 0.566101092 |
| TCGA_TCGA-55-7284 | 0.217845 | 0.398767189 | 0.469077011 | 0.63555158 | 0.593921657 | 0.675422735 | 0.298517492 | 0.612254418 | 0.451201324 | 0.69644064 | 0.739900847 | 0.458683189 | 0.537389724 | 0.781228296 | 0.476640264 | 0.690573367 | 0.255695414 | 0.717686489 | 0.593833641 | 0.571533341 | 0.52450254 | 0.492367903 | 0.563134175 |
| TCGA_TCGA-44-2656 | 0.365398494 | 0.631380832 | 0.579108933 | 0.708275285 | 0.598600075 | 0.657523164 | 0.421645716 | 0.654313712 | 0.667829888 | 0.700093168 | 0.779105167 | 0.445026337 | 0.559304467 | 0.760677111 | 0.502829244 | 0.703151124 | 0.202605914 | 0.674644479 | 0.743707917 | 0.586402656 | 0.528856259 | 0.425433151 | 0.604748449 |
| TCGA_TCGA-44-6778 | 0.498544388 | 0.672746848 | 0.679601783 | 0.675101307 | 0.57032066 | 0.590062989 | 0.487898678 | 0.683714285 | 0.684443411 | 0.711158311 | 0.914881903 | 0.506575364 | 0.672240609 | 0.794513642 | 0.521888394 | 0.734786698 | 0.190198735 | 0.730356772 | 0.797791289 | 0.665130964 | 0.560941359 | 0.350501707 | 0.60400996 |
| TCGA_TCGA-35-4123 | 0.325840555 | 0.657278674 | 0.759132981 | 0.716779232 | 0.640495964 | 0.676857705 | 0.314159 | 0.686154345 | 0.523665315 | 0.680634758 | 0.922043836 | 0.463347567 | 0.583027701 | 0.76133343 | 0.619371906 | 0.70238385 | 0.181216912 | 0.695237889 | 0.78894052 | 0.613188896 | 0.62134291 | 0.423665015 | 0.500915775 |
| TCGA_TCGA-44-6776 | 0.241994979 | 0.351538703 | 0.44923597 | 0.576366673 | 0.550795893 | 0.627180082 | 0.387292962 | 0.609053605 | 0.371708978 | 0.652768857 | 0.492972265 | 0.325404481 | 0.51091651 | 0.735767863 | 0.344881374 | 0.57217907 | 0.17109594 | 0.644746133 | 0.431501038 | 0.535205723 | 0.441148202 | 0.351425778 | 0.55693679 |
| TCGA_TCGA-73-4676 | 0.15932958 | 0.571999752 | 0.450019646 | 0.68108259 | 0.589357865 | 0.641546319 | 0.316606106 | 0.658641437 | 0.452796794 | 0.754390958 | 0.67482612 | 0.406789074 | 0.548046763 | 0.775156605 | 0.493641341 | 0.624653172 | 0.240065148 | 0.65619898 | 0.618932159 | 0.561375016 | 0.514169193 | 0.297854068 | 0.548123587 |
| TCGA_TCGA-86-8673 | 0.250722127 | 0.498548911 | 0.547691118 | 0.652222241 | 0.621386851 | 0.682986089 | 0.241079554 | 0.616478868 | 0.36379749 | 0.636755641 | 0.747285458 | 0.391924987 | 0.365559446 | 0.749918912 | 0.477177414 | 0.63058084 | 0.24504061 | 0.672708285 | 0.52807645 | 0.492035348 | 0.478788287 | 0.418876331 | 0.467564434 |
| TCGA_TCGA-62-8395 | 0.230955316 | 0.31978397 | 0.437253505 | 0.587821118 | 0.546037264 | 0.64247768 | 0.377011989 | 0.522241033 | 0.417703583 | 0.659370669 | 0.629516743 | 0.402855521 | 0.443348816 | 0.736164732 | 0.415387855 | 0.594066496 | 0.178785196 | 0.677967146 | 0.538739385 | 0.55519853 | 0.481772796 | 0.437541675 | 0.442853178 |
| TCGA_TCGA-44-A4SS | 0.328352809 | 0.58302469 | 0.606490835 | 0.653433892 | 0.593776248 | 0.668656502 | 0.376601291 | 0.60707482 | 0.490273287 | 0.702195359 | 0.80628382 | 0.480434845 | 0.457333995 | 0.770855188 | 0.541575337 | 0.682386231 | 0.127587529 | 0.708766226 | 0.740698475 | 0.5441477 | 0.561915851 | 0.439777184 | 0.50706953 |
| TCGA_TCGA-86-8358 | 0.184303717 | 0.620443335 | 0.410846689 | 0.547238182 | 0.477286316 | 0.643936742 | 0.248331949 | 0.612587874 | 0.389331106 | 0.596246365 | 0.529059066 | 0.291527656 | 0.272938873 | 0.666612745 | 0.433712476 | 0.624169352 | 0.043022187 | 0.586934084 | 0.617629594 | 0.500945085 | 0.409694165 | 0.331403372 | 0.543649589 |
| TCGA_TCGA-55-6968 | 0.34083342 | 0.646020194 | 0.522766423 | 0.634852966 | 0.55701196 | 0.601543911 | 0.211915639 | 0.648171513 | 0.548298934 | 0.679280582 | 0.758677206 | 0.404457029 | 0.359049309 | 0.716409668 | 0.488954437 | 0.663519554 | 0.068246752 | 0.669378891 | 0.715218222 | 0.574363733 | 0.511081226 | 0.335981202 | 0.488742697 |
| TCGA_TCGA-64-5775 | 0.1354076 | 0.527809579 | 0.400351929 | 0.65323831 | 0.631641411 | 0.590940809 | 0.246626816 | 0.713061079 | 0.406174614 | 0.605479664 | 0.67863792 | 0.50154928 | 0.358160965 | 0.737565377 | 0.541578416 | 0.644635045 | 0.135108857 | 0.703107556 | 0.60062767 | 0.503752723 | 0.472886133 | 0.345951317 | 0.487640929 |
| TCGA_TCGA-97-7547 | 0.369057209 | 0.344605222 | 0.424980308 | 0.620285521 | 0.574802903 | 0.6392542 | 0.294393894 | 0.51748246 | 0.482230841 | 0.718219527 | 0.661446305 | 0.349241199 | 0.539559054 | 0.793895711 | 0.444623322 | 0.633256206 | 0.166401756 | 0.720336022 | 0.610434725 | 0.548608389 | 0.527069835 | 0.364537683 | 0.458272064 |
| TCGA_TCGA-75-7031 | 0.251511459 | 0.530306304 | 0.604557707 | 0.67301208 | 0.612727358 | 0.749607918 | 0.335650909 | 0.579132692 | 0.420772082 | 0.716497096 | 0.713877763 | 0.389205505 | 0.387954557 | 0.753134052 | 0.415712844 | 0.617107188 | 0.168873723 | 0.646842397 | 0.632235288 | 0.534766007 | 0.484766079 | 0.36817977 | 0.510846299 |
| TCGA_TCGA-44-2655 | 0.407192989 | 0.469194568 | 0.500707442 | 0.695019094 | 0.603366738 | 0.625986215 | 0.336614076 | 0.625169883 | 0.475884065 | 0.707283375 | 0.697460703 | 0.41418264 | 0.489564526 | 0.754617041 | 0.432723632 | 0.617112659 | 0.22684281 | 0.685100218 | 0.582666341 | 0.525955024 | 0.505498054 | 0.38639676 | 0.49771476 |
| TCGA_TCGA-75-6212 | 0.507326425 | 0.466233036 | 0.505229678 | 0.735158492 | 0.635405418 | 0.653843853 | 0.402535279 | 0.636942483 | 0.581729779 | 0.716563264 | 0.824327474 | 0.508793013 | 0.533855253 | 0.782256144 | 0.469716812 | 0.673427338 | 0.204890484 | 0.746743753 | 0.654942892 | 0.608357802 | 0.538866622 | 0.432771209 | 0.47213747 |
| TCGA_TCGA-05-4390 | 0.191534408 | 0.501002394 | 0.438176806 | 0.639050655 | 0.571642156 | 0.757473188 | 0.179188823 | 0.652703282 | 0.425618805 | 0.676081723 | 0.697516879 | 0.351827379 | 0.510782242 | 0.752668823 | 0.467591895 | 0.607814409 | 0.343067481 | 0.646998081 | 0.587517567 | 0.54958142 | 0.457666741 | 0.329036663 | 0.57640735 |
| TCGA_TCGA-86-8674 | 0.185858601 | 0.358834813 | 0.453564219 | 0.581612126 | 0.569369987 | 0.687343065 | 0.353475302 | 0.503309009 | 0.294062891 | 0.638891047 | 0.553269698 | 0.337685526 | 0.41015905 | 0.733857248 | 0.361739241 | 0.603449118 | 0.259508793 | 0.609741432 | 0.366270678 | 0.478463246 | 0.446707106 | 0.392489636 | 0.462174388 |
| TCGA_TCGA-49-6742 | 0.128901234 | 0.45541003 | 0.407920124 | 0.609785996 | 0.590076613 | 0.692767529 | 0.330822875 | 0.602833617 | 0.306103339 | 0.645546188 | 0.545529696 | 0.435079363 | 0.407729737 | 0.764852156 | 0.381635578 | 0.611122895 | 0.294941598 | 0.648906056 | 0.439286109 | 0.475788841 | 0.461187406 | 0.363624927 | 0.568137101 |
| TCGA_TCGA-44-A47B | 0.162865983 | 0.527001056 | 0.513190306 | 0.659102214 | 0.635370763 | 0.696114212 | 0.287178689 | 0.558346624 | 0.417927775 | 0.694287336 | 0.706169869 | 0.370161596 | 0.467479611 | 0.717698446 | 0.452998328 | 0.640517178 | 0.131452351 | 0.652952427 | 0.609849125 | 0.532534679 | 0.468795358 | 0.360585845 | 0.486814137 |
| TCGA_TCGA-MP-A4SW | 0.401451461 | 0.470967655 | 0.495016938 | 0.669075201 | 0.606194767 | 0.685046518 | 0.367824157 | 0.623098766 | 0.516797914 | 0.708301238 | 0.787245486 | 0.442928324 | 0.469787027 | 0.761150261 | 0.476342591 | 0.657935744 | 0.215567507 | 0.68908061 | 0.655680891 | 0.5689225 | 0.525915332 | 0.466472841 | 0.454348969 |
| TCGA_TCGA-64-5815 | 0.264261339 | 0.50151477 | 0.464655405 | 0.687816898 | 0.638539309 | 0.708235895 | 0.248671867 | 0.691730085 | 0.484846017 | 0.690061224 | 0.851216117 | 0.479049821 | 0.590754938 | 0.752836763 | 0.542689479 | 0.698512304 | 0.166683885 | 0.712875771 | 0.772246709 | 0.581100814 | 0.570061278 | 0.426237688 | 0.525032711 |
| TCGA_TCGA-MP-A4T8 | 0.131621489 | 0.465566898 | 0.440287167 | 0.589080597 | 0.631955308 | 0.738044779 | 0.220576881 | 0.566413893 | 0.308948744 | 0.67254236 | 0.621446493 | 0.322336633 | 0.39000219 | 0.756301799 | 0.437470019 | 0.599739174 | 0.232684785 | 0.648059893 | 0.468406938 | 0.465683154 | 0.457442791 | 0.327347662 | 0.491890454 |
| TCGA_TCGA-NJ-A4YI | 0.150604743 | 0.327852645 | 0.395191354 | 0.720742403 | 0.539842979 | 0.689224182 | 0.228240147 | 0.595699851 | 0.421437863 | 0.675242835 | 0.75595403 | 0.426398101 | 0.519881307 | 0.756030526 | 0.447559245 | 0.631971951 | 0.162872076 | 0.714270761 | 0.571941003 | 0.556789307 | 0.49868359 | 0.404618421 | 0.408248467 |
| TCGA_TCGA-L4-A4E6 | 0.35768034 | 0.424641451 | 0.49769456 | 0.690505714 | 0.636743695 | 0.622027243 | 0.486520573 | 0.702723157 | 0.544309522 | 0.7434819 | 0.941608511 | 0.583036273 | 0.689362641 | 0.816870834 | 0.565186373 | 0.714375764 | 0.164651713 | 0.764385283 | 0.739288049 | 0.672113107 | 0.585398423 | 0.399386025 | 0.50733313 |
| TCGA_TCGA-55-8302 | 0.303933638 | 0.451617673 | 0.484655728 | 0.636435678 | 0.597016813 | 0.719054643 | 0.233659908 | 0.620011158 | 0.462836868 | 0.699755601 | 0.718349296 | 0.387207685 | 0.437679277 | 0.758883937 | 0.423284259 | 0.644516097 | 0.304090698 | 0.691924295 | 0.624971285 | 0.535599159 | 0.518655535 | 0.383483208 | 0.472404433 |
| TCGA_TCGA-05-4405 | 0.295532275 | 0.395477668 | 0.410465101 | 0.646775487 | 0.583496904 | 0.678610252 | 0.272368503 | 0.576869755 | 0.468277705 | 0.718142681 | 0.736747955 | 0.408496961 | 0.588604413 | 0.721655454 | 0.483008432 | 0.675136572 | 0.192000607 | 0.689737584 | 0.602114603 | 0.581113007 | 0.508935274 | 0.403071492 | 0.532502281 |
| TCGA_TCGA-78-7147 | 0.20511657 | 0.449638442 | 0.478210809 | 0.662190546 | 0.643111194 | 0.658157754 | 0.248265226 | 0.610008033 | 0.368731651 | 0.691643432 | 0.651548593 | 0.371886745 | 0.44578179 | 0.736462303 | 0.420061299 | 0.62290549 | 0.208770325 | 0.671212913 | 0.523628493 | 0.497067644 | 0.482957451 | 0.386823383 | 0.540687272 |
| TCGA_TCGA-MP-A4TD | 0.449860071 | 0.487857199 | 0.525213018 | 0.615259852 | 0.575001116 | 0.72215001 | 0.414184507 | 0.563895226 | 0.525742185 | 0.647692849 | 0.721061563 | 0.416190994 | 0.45469239 | 0.737890133 | 0.452864932 | 0.604978392 | 0.153258165 | 0.674024856 | 0.590567008 | 0.532642645 | 0.51136586 | 0.359404064 | 0.528104026 |
| TCGA_TCGA-55-6971 | 0.582005884 | 0.634086546 | 0.626772435 | 0.714841073 | 0.60589408 | 0.728828714 | 0.436552254 | 0.631546016 | 0.629906041 | 0.687141104 | 0.898968926 | 0.510933796 | 0.583655851 | 0.787569822 | 0.510688751 | 0.683276185 | 0.235865991 | 0.72791444 | 0.759919726 | 0.593859381 | 0.56992879 | 0.37227723 | 0.582380128 |
| TCGA_TCGA-50-6591 | 0.070545274 | 0.458371102 | 0.357488401 | 0.481874825 | 0.562344465 | 0.583951773 | 0.309078576 | 0.593420932 | 0.150296775 | 0.531921753 | 0.387429179 | 0.278928799 | 0.266017996 | 0.640004965 | 0.368821705 | 0.599064857 | 0.065405594 | 0.626883874 | 0.385675032 | 0.458858399 | 0.431842371 | 0.223030164 | 0.648989733 |
| TCGA_TCGA-95-7944 | 0.337091866 | 0.611132216 | 0.748806116 | 0.70681468 | 0.642518511 | 0.709258498 | 0.261921206 | 0.657475478 | 0.478960636 | 0.636376133 | 0.833898313 | 0.508187488 | 0.407170157 | 0.762810443 | 0.515333265 | 0.695718223 | 0.233352453 | 0.68563074 | 0.619352414 | 0.573467931 | 0.574408609 | 0.382672363 | 0.575728004 |
| TCGA_TCGA-NJ-A4YF | 0.201755132 | 0.393115288 | 0.456499806 | 0.606501371 | 0.602542802 | 0.780373324 | 0.342858108 | 0.55232954 | 0.337769269 | 0.689499081 | 0.691721806 | 0.32437401 | 0.302796034 | 0.744141509 | 0.385985012 | 0.54833211 | 0.301202503 | 0.660496456 | 0.436626347 | 0.477774932 | 0.436471377 | 0.363479848 | 0.374953823 |
| TCGA_TCGA-55-8091 | 0.267706224 | 0.450703884 | 0.481742683 | 0.677489065 | 0.611126537 | 0.717635552 | 0.377058815 | 0.641964439 | 0.467274475 | 0.706313429 | 0.828188399 | 0.464958875 | 0.546329869 | 0.751747041 | 0.493074431 | 0.680399265 | 0.241421217 | 0.716784917 | 0.752922648 | 0.559962399 | 0.561839291 | 0.486000109 | 0.519370651 |
| TCGA_TCGA-49-4505 | 0.35892865 | 0.47079285 | 0.563059357 | 0.726232666 | 0.678968356 | 0.698340991 | 0.451174847 | 0.659203673 | 0.478225102 | 0.720187579 | 0.852533404 | 0.45007435 | 0.617969068 | 0.785361563 | 0.489231756 | 0.697422497 | 0.21387813 | 0.689823263 | 0.717716862 | 0.59006136 | 0.551435091 | 0.448834 | 0.469818192 |
| TCGA_TCGA-62-8397 | 0.230666414 | 0.316125049 | 0.445562851 | 0.662052828 | 0.618261909 | 0.66512577 | 0.319175186 | 0.59601729 | 0.462327901 | 0.738060296 | 0.749159119 | 0.441132821 | 0.529980974 | 0.766498989 | 0.435075023 | 0.666221269 | 0.243828156 | 0.699838627 | 0.575773085 | 0.569770475 | 0.489278505 | 0.502285688 | 0.428304819 |
| TCGA_TCGA-50-5942 | 0.295143788 | 0.362448081 | 0.422143446 | 0.61698767 | 0.549537952 | 0.595753797 | 0.41451217 | 0.496773042 | 0.467207912 | 0.675752871 | 0.602193022 | 0.393803244 | 0.527515792 | 0.767780028 | 0.410806445 | 0.651204792 | 0.165036429 | 0.672452297 | 0.512468569 | 0.550263385 | 0.493426781 | 0.450674671 | 0.533959024 |
| TCGA_TCGA-55-8206 | 0.326230404 | 0.377586831 | 0.464942189 | 0.721003901 | 0.553537788 | 0.624813934 | 0.477139504 | 0.619563537 | 0.529394535 | 0.712568871 | 0.85833241 | 0.533440906 | 0.660847121 | 0.838088816 | 0.480581172 | 0.692070291 | 0.310476681 | 0.722926276 | 0.67090321 | 0.620312458 | 0.555039215 | 0.447154286 | 0.460914696 |
| TCGA_TCGA-55-8094 | 0.093602404 | 0.446254873 | 0.444757711 | 0.545116287 | 0.675492097 | 0.746657863 | 0.227854618 | 0.532262187 | 0.232035169 | 0.614574488 | 0.376046187 | 0.268042055 | 0.401882452 | 0.774901118 | 0.402861118 | 0.522940998 | 0.324829342 | 0.644002892 | 0.414904482 | 0.463830263 | 0.373814702 | 0.383242852 | 0.555206512 |
| TCGA_TCGA-05-4382 | 0.355671971 | 0.512190976 | 0.460579146 | 0.681717254 | 0.600306719 | 0.687975646 | 0.236105793 | 0.716814151 | 0.592869956 | 0.698830868 | 0.877329543 | 0.505430769 | 0.487539225 | 0.77732461 | 0.559592191 | 0.724187561 | 0.187352244 | 0.707173336 | 0.818842779 | 0.591385004 | 0.599244041 | 0.402585591 | 0.448515383 |
| TCGA_TCGA-05-5423 | 0.310526838 | 0.518543813 | 0.618370082 | 0.727448294 | 0.649316678 | 0.648337375 | 0.440462372 | 0.693964471 | 0.531504279 | 0.779504096 | 0.868433172 | 0.499826088 | 0.626151431 | 0.799801711 | 0.478261692 | 0.711616353 | 0.221477406 | 0.702890032 | 0.749871731 | 0.629791219 | 0.554223344 | 0.45001289 | 0.533496317 |
| TCGA_TCGA-49-AAR4 | 0.424806737 | 0.608651181 | 0.746275022 | 0.665039026 | 0.589287287 | 0.691845925 | 0.350395356 | 0.620560222 | 0.526065565 | 0.693673992 | 0.822609179 | 0.36209836 | 0.474134849 | 0.75944268 | 0.466657867 | 0.702782764 | 0.208799692 | 0.702713345 | 0.619225464 | 0.545504598 | 0.554243361 | 0.335193685 | 0.446576372 |
| TCGA_TCGA-97-A4M3 | 0.18699533 | 0.397821032 | 0.411150806 | 0.609785981 | 0.516031349 | 0.719216179 | 0.469445776 | 0.524909706 | 0.376394105 | 0.639352976 | 0.603533742 | 0.374144538 | 0.48622856 | 0.718938565 | 0.419433641 | 0.606525548 | 0.239331391 | 0.682433488 | 0.491014514 | 0.513345199 | 0.491373252 | 0.369506406 | 0.481685686 |
| TCGA_TCGA-05-4397 | 0.120730372 | 0.57910215 | 0.4186342 | 0.652609683 | 0.617792728 | 0.673166583 | 0.211749163 | 0.639278368 | 0.437306664 | 0.689255824 | 0.689319222 | 0.402476199 | 0.283479474 | 0.763139496 | 0.469723327 | 0.558378429 | 0.114096662 | 0.704787318 | 0.568849333 | 0.510073049 | 0.46060869 | 0.345858211 | 0.474493436 |
| TCGA_TCGA-55-6984 | 0.340448803 | 0.501814787 | 0.474630042 | 0.604813801 | 0.613914014 | 0.736677904 | 0.33940778 | 0.556108787 | 0.45055102 | 0.664927601 | 0.62976552 | 0.369996989 | 0.44136493 | 0.77806866 | 0.447347643 | 0.645920259 | 0.224122137 | 0.667710073 | 0.479087058 | 0.478427979 | 0.49999025 | 0.494983991 | 0.535348458 |
| TCGA_TCGA-05-4427 | 0.248341972 | 0.549860525 | 0.464014983 | 0.654730558 | 0.587153316 | 0.660005154 | 0.328996852 | 0.64808353 | 0.472971322 | 0.678662136 | 0.724693336 | 0.380162697 | 0.460892952 | 0.731809713 | 0.50693676 | 0.672283357 | 0.116731971 | 0.688031736 | 0.692381946 | 0.533801624 | 0.49806433 | 0.389574735 | 0.533485202 |
| TCGA_TCGA-86-8281 | 0.305398714 | 0.333490492 | 0.486772048 | 0.615193275 | 0.523634807 | 0.645126441 | 0.291068537 | 0.555597664 | 0.434353148 | 0.625138151 | 0.608677632 | 0.373642946 | 0.46444798 | 0.726218802 | 0.391400856 | 0.592120044 | 0.215319198 | 0.656829232 | 0.513298043 | 0.52870814 | 0.479860821 | 0.416430521 | 0.492518693 |
| TCGA_TCGA-44-A4SU | 0.457932524 | 0.391200128 | 0.49703411 | 0.653326395 | 0.578251501 | 0.675492312 | 0.325576265 | 0.552462103 | 0.504783162 | 0.680125473 | 0.683502886 | 0.336663061 | 0.50555626 | 0.731914383 | 0.416345914 | 0.659317711 | 0.129705264 | 0.686748982 | 0.548221779 | 0.523804738 | 0.499793947 | 0.374864621 | 0.43293611 |
| TCGA_TCGA-75-5147 | 0.329452983 | 0.565037667 | 0.521919761 | 0.703357371 | 0.652991361 | 0.700067891 | 0.242920655 | 0.644126773 | 0.489073109 | 0.727047176 | 0.81920484 | 0.406803008 | 0.561196902 | 0.744198956 | 0.443351563 | 0.671809295 | 0.178471771 | 0.707745418 | 0.700529393 | 0.525846293 | 0.560321842 | 0.373766492 | 0.504296438 |
| TCGA_TCGA-97-7546 | 0.398680657 | 0.488999702 | 0.481563421 | 0.699300763 | 0.591295349 | 0.603594347 | 0.45574633 | 0.616906787 | 0.523386596 | 0.645855653 | 0.754072091 | 0.439626571 | 0.596987565 | 0.752993438 | 0.469301465 | 0.676953652 | 0.20826062 | 0.703821205 | 0.641335601 | 0.584087798 | 0.570484995 | 0.391854555 | 0.569724697 |
| TCGA_TCGA-86-8585 | 0.403068622 | 0.622115114 | 0.634465566 | 0.679352903 | 0.590769198 | 0.707096118 | 0.297108171 | 0.636772427 | 0.551151484 | 0.681902406 | 0.92178467 | 0.459792593 | 0.494166037 | 0.791177243 | 0.490939027 | 0.603388563 | 0.241643781 | 0.677718458 | 0.640824435 | 0.518928281 | 0.532903565 | 0.328555282 | 0.445534519 |
| TCGA_TCGA-55-8301 | 0.534963402 | 0.618870408 | 0.70196022 | 0.68624856 | 0.608379719 | 0.652502418 | 0.305442874 | 0.663086721 | 0.653408572 | 0.718305245 | 0.910902563 | 0.431307373 | 0.518384044 | 0.774859492 | 0.505758728 | 0.692153683 | 0.151947878 | 0.705479376 | 0.719476914 | 0.590003644 | 0.567117073 | 0.370405119 | 0.464432562 |
| TCGA_TCGA-86-6562 | 0.217040305 | 0.447187847 | 0.417755833 | 0.633769952 | 0.588375486 | 0.638753847 | 0.372687562 | 0.645191804 | 0.446416249 | 0.677134724 | 0.688512103 | 0.422712483 | 0.564490616 | 0.718367444 | 0.465549176 | 0.641673547 | 0.246349443 | 0.681400135 | 0.636497839 | 0.541464123 | 0.522572394 | 0.353415897 | 0.590481682 |
| TCGA_TCGA-J2-A4AD | 0.108544873 | 0.418963002 | 0.378524931 | 0.55987309 | 0.615156901 | 0.589464734 | 0.272637982 | 0.551861455 | 0.335232904 | 0.690878796 | 0.563858269 | 0.272574142 | 0.390284257 | 0.753704206 | 0.399886857 | 0.589963788 | 0.184024391 | 0.650134946 | 0.455266827 | 0.437659548 | 0.504138474 | 0.371392258 | 0.439664775 |
| TCGA_TCGA-55-8510 | 0.337936122 | 0.477938757 | 0.548694268 | 0.692612042 | 0.602518274 | 0.69065944 | 0.303406669 | 0.655557634 | 0.515618443 | 0.73526689 | 0.906363714 | 0.503373351 | 0.5375334 | 0.789166722 | 0.512986035 | 0.720397765 | 0.227577747 | 0.716525544 | 0.702577065 | 0.63740224 | 0.568273373 | 0.423933492 | 0.47153209 |
| TCGA_TCGA-99-8033 | 0.307478492 | 0.623599035 | 0.592025659 | 0.686729206 | 0.613103642 | 0.690886151 | 0.297077876 | 0.585835411 | 0.446685861 | 0.692872567 | 0.766461198 | 0.380078151 | 0.511798653 | 0.725376171 | 0.489792367 | 0.658147632 | 0.351062326 | 0.646903396 | 0.593977383 | 0.51633905 | 0.530480095 | 0.377883229 | 0.569005305 |
| TCGA_TCGA-69-7978 | 0.423262636 | 0.642763807 | 0.622492313 | 0.68748095 | 0.606667625 | 0.709033957 | 0.259516412 | 0.674829856 | 0.606765358 | 0.679227461 | 0.946593144 | 0.469558235 | 0.530921066 | 0.750260475 | 0.576543942 | 0.724950483 | 0.209791785 | 0.715041548 | 0.849165134 | 0.610878802 | 0.609535851 | 0.430345813 | 0.500437447 |
| TCGA_TCGA-86-8054 | 0.134975525 | 0.556277878 | 0.406070112 | 0.536765748 | 0.594856754 | 0.636896362 | 0.323351833 | 0.611069537 | 0.340170816 | 0.660630597 | 0.598640355 | 0.340505742 | 0.466391158 | 0.677092579 | 0.401102238 | 0.59713626 | 0.216292996 | 0.632616764 | 0.536164919 | 0.48539918 | 0.426721607 | 0.263289694 | 0.58227796 |
| TCGA_TCGA-44-7660 | 0.254344811 | 0.493917121 | 0.442092409 | 0.598962878 | 0.621063359 | 0.661016556 | 0.325386598 | 0.567823628 | 0.480902621 | 0.674229004 | 0.68050277 | 0.413444955 | 0.25716744 | 0.695280909 | 0.391258696 | 0.586447026 | 0.055548811 | 0.625143781 | 0.521112623 | 0.509068392 | 0.472055581 | 0.309892009 | 0.501253169 |
| TCGA_TCGA-78-8662 | 0.154714878 | 0.441267184 | 0.430619289 | 0.595418333 | 0.556557737 | 0.683220147 | 0.416514766 | 0.581970534 | 0.284866758 | 0.633444749 | 0.457967049 | 0.332684218 | 0.405051045 | 0.733631398 | 0.382043902 | 0.600590916 | 0.176755049 | 0.59661854 | 0.422826759 | 0.470201566 | 0.450113416 | 0.327794975 | 0.544894758 |
| TCGA_TCGA-75-7027 | 0.221878368 | 0.547894221 | 0.43993322 | 0.597212543 | 0.596496207 | 0.671338738 | 0.232932476 | 0.572372498 | 0.408198776 | 0.675288468 | 0.581455012 | 0.402996462 | 0.430127248 | 0.738436647 | 0.474975275 | 0.588447464 | 0.187276197 | 0.672568292 | 0.476478845 | 0.461822197 | 0.461455367 | 0.394906094 | 0.566373594 |
| TCGA_TCGA-97-A4M6 | 0.450883853 | 0.491590679 | 0.578157622 | 0.740995485 | 0.645235874 | 0.716813413 | 0.300411312 | 0.592336976 | 0.537637562 | 0.67827842 | 0.836852352 | 0.458798476 | 0.52041089 | 0.803863033 | 0.455703437 | 0.699634868 | 0.248287564 | 0.716133934 | 0.655355501 | 0.567522557 | 0.532921371 | 0.415521464 | 0.475606538 |
| TCGA_TCGA-MP-A4SV | 0.322206763 | 0.614387461 | 0.591345587 | 0.651376247 | 0.58182996 | 0.72811841 | 0.32370151 | 0.588119885 | 0.535842944 | 0.671989209 | 0.785070103 | 0.370994821 | 0.44345026 | 0.747937483 | 0.465168643 | 0.633276199 | 0.156852381 | 0.662165549 | 0.635010335 | 0.551945133 | 0.506574506 | 0.381743253 | 0.498867983 |
| TCGA_TCGA-38-4627 | 0.241691801 | 0.443385704 | 0.484761613 | 0.666671746 | 0.654885646 | 0.645570353 | 0.271295548 | 0.73723301 | 0.440273975 | 0.737696076 | 0.853102827 | 0.500498895 | 0.57680302 | 0.772945346 | 0.534149445 | 0.695000903 | 0.155071874 | 0.75010017 | 0.753538172 | 0.59528601 | 0.583273205 | 0.397290083 | 0.508505775 |
| TCGA_TCGA-93-A4JO | 0.449353954 | 0.515556423 | 0.611683994 | 0.68817774 | 0.572329902 | 0.624943287 | 0.36824719 | 0.5788741 | 0.541941702 | 0.716394382 | 0.879016484 | 0.434268702 | 0.592825108 | 0.779381877 | 0.453821099 | 0.697025742 | 0.169268739 | 0.692316914 | 0.702794592 | 0.570895741 | 0.548604339 | 0.357628762 | 0.50430629 |
| TCGA_TCGA-05-4426 | 0.191645811 | 0.420509502 | 0.417753409 | 0.640863123 | 0.618420104 | 0.625133395 | 0.228851364 | 0.614809215 | 0.387164193 | 0.764431297 | 0.730399738 | 0.412258889 | 0.472463765 | 0.744341864 | 0.45307403 | 0.685157842 | 0.258077959 | 0.714687313 | 0.697340906 | 0.534364085 | 0.494035236 | 0.385124656 | 0.472683805 |
| TCGA_TCGA-97-7937 | 0.161868138 | 0.365987123 | 0.393931032 | 0.615434577 | 0.624092535 | 0.728730259 | 0.299296951 | 0.565954732 | 0.404105029 | 0.706643199 | 0.581153632 | 0.344256948 | 0.521779818 | 0.723673814 | 0.412271991 | 0.613454317 | 0.182362409 | 0.64085705 | 0.553897552 | 0.531237178 | 0.469093554 | 0.37062733 | 0.529636215 |
| TCGA_TCGA-75-6206 | 0.227120234 | 0.329410132 | 0.414760403 | 0.669921788 | 0.557820918 | 0.588716273 | 0.462690936 | 0.601355634 | 0.450171189 | 0.66252556 | 0.697502789 | 0.452540839 | 0.535056271 | 0.732980935 | 0.461694679 | 0.62236813 | 0.2353223 | 0.689831462 | 0.600278551 | 0.591417483 | 0.496205356 | 0.438098322 | 0.510618031 |
| TCGA_TCGA-62-A472 | 0.212321402 | 0.552191804 | 0.514997511 | 0.644627889 | 0.67144242 | 0.721333584 | 0.266767773 | 0.607278564 | 0.455901025 | 0.72222184 | 0.749223429 | 0.401475006 | 0.519721244 | 0.74972219 | 0.430695921 | 0.604484052 | 0.217465998 | 0.663721982 | 0.641242889 | 0.53177414 | 0.492839479 | 0.418624596 | 0.523651217 |
| TCGA_TCGA-99-AA5R | 0.604321258 | 0.526360016 | 0.67749267 | 0.659796664 | 0.62841576 | 0.627802443 | 0.483686237 | 0.631875612 | 0.67328357 | 0.703127549 | 0.864906571 | 0.481167616 | 0.670668626 | 0.788877227 | 0.549110214 | 0.712777502 | 0.155808302 | 0.739864433 | 0.673959 | 0.621037356 | 0.595980753 | 0.399651122 | 0.529763518 |
| TCGA_TCGA-55-8085 | 0.419612111 | 0.629303473 | 0.610385339 | 0.710793523 | 0.589377413 | 0.662789008 | 0.249523399 | 0.617577205 | 0.525480142 | 0.667913907 | 0.824137486 | 0.444564134 | 0.458486704 | 0.746056137 | 0.45898673 | 0.665971758 | 0.144253659 | 0.66046675 | 0.638237079 | 0.548656651 | 0.521497772 | 0.379129077 | 0.500544265 |
| TCGA_TCGA-44-8117 | 0.232867513 | 0.431692069 | 0.4676239 | 0.621591497 | 0.56843511 | 0.628098159 | 0.387165996 | 0.613747107 | 0.393250868 | 0.670047311 | 0.631658667 | 0.379036924 | 0.464770001 | 0.727008693 | 0.393917108 | 0.615644605 | 0.161850725 | 0.637828947 | 0.609199321 | 0.526108598 | 0.480738358 | 0.430177944 | 0.480141495 |
| TCGA_TCGA-55-8506 | 0.217739909 | 0.480239275 | 0.459461473 | 0.652310367 | 0.59549577 | 0.711513086 | 0.277980154 | 0.581229181 | 0.440797025 | 0.678165548 | 0.741608574 | 0.380834581 | 0.565990634 | 0.735042137 | 0.474212405 | 0.6278653 | 0.27015971 | 0.693931404 | 0.61810944 | 0.5594119 | 0.49033522 | 0.394584225 | 0.540934143 |
| TCGA_TCGA-NJ-A7XG | 0.176919799 | 0.337158962 | 0.386558759 | 0.623075443 | 0.633309948 | 0.660349027 | 0.283898519 | 0.49026573 | 0.327158077 | 0.728492259 | 0.572880821 | 0.340478562 | 0.492437962 | 0.722039449 | 0.316436174 | 0.595716691 | 0.2060068 | 0.631510092 | 0.388812287 | 0.464379113 | 0.454919909 | 0.379263893 | 0.505424648 |
| TCGA_TCGA-05-4384 | 0.225337296 | 0.363487349 | 0.39452871 | 0.665126438 | 0.546853278 | 0.607269141 | 0.386853817 | 0.617832541 | 0.441756751 | 0.693065717 | 0.726049538 | 0.466906203 | 0.530587412 | 0.749345905 | 0.448361642 | 0.61114149 | 0.261528582 | 0.696105277 | 0.628666395 | 0.565617993 | 0.475271824 | 0.391955258 | 0.494701341 |
| TCGA_TCGA-64-1679 | 0.222047621 | 0.428949348 | 0.368157248 | 0.713108811 | 0.618671421 | 0.654673905 | 0.212635801 | 0.602386335 | 0.438105018 | 0.696814749 | 0.776070807 | 0.43977015 | 0.511192358 | 0.766884582 | 0.500076015 | 0.684310221 | 0.21527573 | 0.683455504 | 0.728109876 | 0.564554246 | 0.537556762 | 0.40746144 | 0.491383485 |
| TCGA_TCGA-73-4666 | 0.27071702 | 0.686780589 | 0.66462112 | 0.710077044 | 0.601917214 | 0.681192261 | 0.304969837 | 0.699997887 | 0.532678022 | 0.709734013 | 0.888024859 | 0.470388238 | 0.421763181 | 0.758452273 | 0.532079807 | 0.668731217 | 0.272160637 | 0.657212861 | 0.754175341 | 0.610042426 | 0.558139262 | 0.394522597 | 0.534261943 |
| TCGA_TCGA-55-8616 | 0.390076898 | 0.424976519 | 0.474315221 | 0.592228788 | 0.568038661 | 0.694203813 | 0.349036392 | 0.540235235 | 0.43692851 | 0.704920798 | 0.594398949 | 0.319209199 | 0.500038 | 0.747383685 | 0.353914896 | 0.631152292 | 0.260123827 | 0.653472947 | 0.468142055 | 0.538268939 | 0.476112136 | 0.319684505 | 0.469253947 |
| TCGA_TCGA-38-6178 | 0.277202099 | 0.45278978 | 0.406096185 | 0.630462424 | 0.603742241 | 0.656585831 | 0.324225692 | 0.591009545 | 0.440276316 | 0.660430979 | 0.671025713 | 0.343998466 | 0.515509745 | 0.741091164 | 0.42016254 | 0.675387088 | 0.193808034 | 0.671864252 | 0.552243532 | 0.533221564 | 0.493428321 | 0.376134121 | 0.507445427 |
| TCGA_TCGA-93-7348 | 0.370044229 | 0.454118643 | 0.444441451 | 0.63400245 | 0.613556192 | 0.644017326 | 0.464562259 | 0.52583077 | 0.500241385 | 0.688243808 | 0.697467768 | 0.380803922 | 0.498223966 | 0.740112941 | 0.442377199 | 0.653837025 | 0.192943551 | 0.681886405 | 0.563677539 | 0.535702931 | 0.540382982 | 0.398208801 | 0.532401284 |
| TCGA_TCGA-55-1594 | 0.284294994 | 0.489779735 | 0.534724403 | 0.608870897 | 0.635741816 | 0.619419981 | 0.251300163 | 0.578650847 | 0.433406124 | 0.69402073 | 0.634841577 | 0.384966974 | 0.419087342 | 0.741041163 | 0.394649105 | 0.633143104 | 0.094963711 | 0.651765655 | 0.648532608 | 0.560249803 | 0.473670476 | 0.3701195 | 0.426357799 |
| TCGA_TCGA-73-4658 | 0.290823683 | 0.544346917 | 0.553194923 | 0.721870273 | 0.637474811 | 0.674216513 | 0.444856369 | 0.657839178 | 0.512136907 | 0.723015626 | 0.881970967 | 0.475029381 | 0.566303111 | 0.804879344 | 0.527674231 | 0.737871634 | 0.231755892 | 0.707751254 | 0.811719353 | 0.631023692 | 0.585469824 | 0.443420883 | 0.500442344 |
| TCGA_TCGA-80-5607 | 0.296728484 | 0.596868943 | 0.546693049 | 0.685528012 | 0.603138245 | 0.655711555 | 0.439346315 | 0.601977125 | 0.439538684 | 0.692632319 | 0.744677063 | 0.40260711 | 0.53227849 | 0.759024367 | 0.458465597 | 0.676643045 | 0.21501053 | 0.657256676 | 0.624283212 | 0.545143798 | 0.49181727 | 0.406507243 | 0.581419456 |
| TCGA_TCGA-55-8505 | 0.215194996 | 0.409630538 | 0.383423546 | 0.581434079 | 0.582465937 | 0.685668396 | 0.2011854 | 0.5738374 | 0.365741527 | 0.656416764 | 0.593307976 | 0.352177515 | 0.422094799 | 0.774522998 | 0.505035625 | 0.598033437 | 0.237395401 | 0.665423982 | 0.528589821 | 0.465028464 | 0.48551387 | 0.389189804 | 0.49640794 |
| TCGA_TCGA-MP-A4T4 | 0.440399347 | 0.582888436 | 0.675747499 | 0.642960115 | 0.601412166 | 0.69541086 | 0.26741274 | 0.620742138 | 0.586152692 | 0.684075698 | 0.862840557 | 0.437475055 | 0.4942612 | 0.745182858 | 0.54760221 | 0.686931263 | 0.210452152 | 0.708440814 | 0.719346063 | 0.602822574 | 0.581520053 | 0.380949873 | 0.463157007 |
| TCGA_TCGA-49-4506 | 0.354663907 | 0.710735867 | 0.69773862 | 0.692609276 | 0.609591615 | 0.672064909 | 0.278344809 | 0.634192364 | 0.519130994 | 0.679718122 | 0.784756744 | 0.45555635 | 0.394481231 | 0.775842094 | 0.457129938 | 0.631012061 | 0.257482965 | 0.638824464 | 0.584270293 | 0.532496207 | 0.541976492 | 0.332597168 | 0.527482835 |
| TCGA_TCGA-97-8172 | 0.594636647 | 0.467311458 | 0.573354722 | 0.678282179 | 0.546697725 | 0.676147336 | 0.282164985 | 0.590762499 | 0.652280958 | 0.66136607 | 0.814324683 | 0.467326548 | 0.574763381 | 0.776719446 | 0.469317997 | 0.672750348 | 0.177091636 | 0.684251176 | 0.645552725 | 0.592382079 | 0.548929642 | 0.394114659 | 0.484695611 |
| TCGA_TCGA-O1-A52J | 0.164825758 | 0.422534274 | 0.485459494 | 0.692915853 | 0.605783577 | 0.673483758 | 0.362713646 | 0.630847619 | 0.446824969 | 0.767219483 | 0.864976684 | 0.522909206 | 0.534779521 | 0.78434158 | 0.463528254 | 0.667081677 | 0.214882783 | 0.719543565 | 0.66057576 | 0.598004361 | 0.51721048 | 0.468970673 | 0.420353929 |
| TCGA_TCGA-78-7537 | 0.316456198 | 0.323849563 | 0.449127171 | 0.632115392 | 0.591697207 | 0.642202144 | 0.297361322 | 0.578010241 | 0.405310981 | 0.643168557 | 0.572402393 | 0.402016168 | 0.431013471 | 0.749702659 | 0.407144632 | 0.6235583 | 0.142576721 | 0.704374486 | 0.485699192 | 0.532232145 | 0.48804906 | 0.406385398 | 0.512626914 |
| TCGA_TCGA-73-4662 | 0.408865465 | 0.520073204 | 0.536499273 | 0.640788067 | 0.603298007 | 0.69465307 | 0.343610427 | 0.641251929 | 0.55016635 | 0.665557727 | 0.760920856 | 0.409650887 | 0.540694772 | 0.783367056 | 0.442723629 | 0.625674538 | 0.196650286 | 0.693212249 | 0.677613198 | 0.576648713 | 0.522605532 | 0.396396348 | 0.492138909 |
| TCGA_TCGA-62-8398 | 0.243158917 | 0.544398458 | 0.488442138 | 0.662310848 | 0.598726133 | 0.69038558 | 0.268528407 | 0.607687762 | 0.420867094 | 0.706094809 | 0.695821157 | 0.439028111 | 0.46850472 | 0.765896901 | 0.468771076 | 0.587983081 | 0.288197023 | 0.677789075 | 0.62765543 | 0.521711704 | 0.527241057 | 0.426858309 | 0.590081141 |
| TCGA_TCGA-49-AAQV | 0.424572423 | 0.525797384 | 0.570967494 | 0.676833823 | 0.710643501 | 0.682576357 | 0.311365424 | 0.580810907 | 0.445314834 | 0.725486762 | 0.817915944 | 0.324932295 | 0.43714165 | 0.758508436 | 0.422631438 | 0.622449664 | 0.259661344 | 0.665164791 | 0.51970277 | 0.516539157 | 0.493867624 | 0.386255315 | 0.458807309 |
| TCGA_TCGA-55-8621 | 0.391040768 | 0.44545652 | 0.539320906 | 0.691240871 | 0.599316699 | 0.632065627 | 0.416194184 | 0.693622722 | 0.582600764 | 0.7712753 | 0.901840131 | 0.498074096 | 0.64381815 | 0.786882432 | 0.528572481 | 0.7041084 | 0.194459278 | 0.752569009 | 0.748277139 | 0.619138383 | 0.552025403 | 0.395650532 | 0.491744987 |
| TCGA_TCGA-53-7626 | 0.449466231 | 0.576717336 | 0.607832019 | 0.701157782 | 0.596023441 | 0.608517388 | 0.491330689 | 0.633846601 | 0.571125822 | 0.702145969 | 0.858947235 | 0.460966063 | 0.716228521 | 0.744649445 | 0.487569501 | 0.719192266 | 0.217014254 | 0.682775851 | 0.695650231 | 0.613740358 | 0.56910288 | 0.387555526 | 0.559381807 |
| TCGA_TCGA-44-7669 | 0.269345378 | 0.608531991 | 0.471651064 | 0.609016869 | 0.547838785 | 0.625540333 | 0.429764732 | 0.642924387 | 0.497500374 | 0.705241824 | 0.721526745 | 0.426267095 | 0.472782288 | 0.724866889 | 0.421374586 | 0.695088093 | 0.178738265 | 0.667127541 | 0.671000852 | 0.5600844 | 0.449860783 | 0.307419682 | 0.593864892 |
| TCGA_TCGA-93-8067 | 0.213447097 | 0.505904479 | 0.484245546 | 0.644327182 | 0.630887118 | 0.651680141 | 0.346524661 | 0.580872084 | 0.359672247 | 0.627500152 | 0.614640039 | 0.363774261 | 0.426830042 | 0.714085935 | 0.42572071 | 0.619576128 | 0.231646174 | 0.627990861 | 0.539639132 | 0.497267931 | 0.461958431 | 0.370803548 | 0.55562578 |
| TCGA_TCGA-97-A4M2 | 0.410402029 | 0.439058937 | 0.583779525 | 0.706884967 | 0.578329903 | 0.646231191 | 0.424725159 | 0.634710773 | 0.588485975 | 0.743971615 | 0.895189805 | 0.539490026 | 0.650167756 | 0.802936574 | 0.483906314 | 0.679814529 | 0.227655382 | 0.737736243 | 0.662813309 | 0.611011562 | 0.553360518 | 0.416493115 | 0.480041289 |
| TCGA_TCGA-05-4425 | 0.2131339 | 0.567347211 | 0.522076624 | 0.676553328 | 0.679039924 | 0.701667795 | 0.230010792 | 0.626028508 | 0.46148755 | 0.684673169 | 0.846188458 | 0.491173666 | 0.570082232 | 0.756097809 | 0.457651914 | 0.728143852 | 0.162771801 | 0.697390238 | 0.743072175 | 0.636438424 | 0.559915674 | 0.465820449 | 0.538933441 |
| TCGA_TCGA-69-7763 | 0.310676206 | 0.368361296 | 0.447388741 | 0.627249837 | 0.549973092 | 0.645668232 | 0.398269438 | 0.593098189 | 0.485999829 | 0.661940303 | 0.658956123 | 0.392507716 | 0.524957719 | 0.735788464 | 0.446521966 | 0.655117583 | 0.213727629 | 0.680979741 | 0.569903938 | 0.551317665 | 0.492775389 | 0.361913699 | 0.500542767 |
| TCGA_TCGA-86-8056 | 0.266006111 | 0.416946976 | 0.398940735 | 0.646633625 | 0.552706124 | 0.642254676 | 0.454279727 | 0.577960081 | 0.493320809 | 0.749996144 | 0.701648463 | 0.399448357 | 0.5129555 | 0.734060739 | 0.442008405 | 0.646080813 | 0.241082282 | 0.722468001 | 0.657286857 | 0.540212783 | 0.505461962 | 0.393975556 | 0.532917751 |
| TCGA_TCGA-50-5931 | 0.184089392 | 0.489603926 | 0.412951985 | 0.498125409 | 0.604748613 | 0.603426994 | 0.413745601 | 0.560253776 | 0.284777012 | 0.646217339 | 0.487045175 | 0.321225728 | 0.312861043 | 0.647656233 | 0.402968638 | 0.562382668 | 0.014040928 | 0.661639122 | 0.370275097 | 0.485778661 | 0.446679303 | 0.416849846 | 0.507701846 |
| TCGA_TCGA-55-8512 | 0.229999628 | 0.273752906 | 0.402889192 | 0.609934653 | 0.565262956 | 0.616997329 | 0.506318525 | 0.557083069 | 0.375670749 | 0.698218269 | 0.619954714 | 0.374384787 | 0.489150242 | 0.731054127 | 0.384333724 | 0.636997841 | 0.354047076 | 0.731231637 | 0.537510086 | 0.494941828 | 0.472015396 | 0.367380799 | 0.464000453 |
| TCGA_TCGA-75-7025 | 0.404362491 | 0.379068991 | 0.470553094 | 0.690788016 | 0.606743645 | 0.640558421 | 0.442820499 | 0.562635379 | 0.502160125 | 0.701129079 | 0.764399166 | 0.417012019 | 0.551218923 | 0.79051371 | 0.440181399 | 0.692362885 | 0.186819593 | 0.704416486 | 0.62818159 | 0.551389932 | 0.5213383 | 0.422699653 | 0.463442753 |
| TCGA_TCGA-50-5930 | 0.446346632 | 0.544130399 | 0.536444764 | 0.641761869 | 0.579019244 | 0.672575211 | 0.331880237 | 0.628061138 | 0.547176194 | 0.691343169 | 0.813237525 | 0.44868714 | 0.477572118 | 0.743160604 | 0.495727401 | 0.631677932 | 0.306975684 | 0.693657194 | 0.621345063 | 0.535314474 | 0.551735977 | 0.383758544 | 0.493788068 |
| TCGA_TCGA-75-6214 | 0.130807167 | 0.445355234 | 0.392153781 | 0.59634342 | 0.672598327 | 0.700227579 | 0.179672583 | 0.634388511 | 0.361092549 | 0.71836464 | 0.611849644 | 0.335887762 | 0.330931105 | 0.713731081 | 0.477061838 | 0.621891734 | 0.42606149 | 0.664954145 | 0.507617275 | 0.498899658 | 0.486176881 | 0.361312528 | 0.504416152 |
| TCGA_TCGA-L9-A743 | 0.467321227 | 0.645762826 | 0.598909447 | 0.706640937 | 0.622495502 | 0.699550775 | 0.43997502 | 0.639690017 | 0.567171768 | 0.715159928 | 0.886359606 | 0.459394663 | 0.547645907 | 0.766203309 | 0.526739946 | 0.719693426 | 0.254753747 | 0.706927711 | 0.742157005 | 0.600170145 | 0.581812556 | 0.385577739 | 0.538551368 |
| TCGA_TCGA-50-5939 | 0.417751943 | 0.570769221 | 0.449513213 | 0.683310243 | 0.603907015 | 0.641620021 | 0.394248214 | 0.680961811 | 0.541062741 | 0.695454143 | 0.821259655 | 0.460775711 | 0.472624085 | 0.751707112 | 0.544382608 | 0.679888481 | 0.344453152 | 0.685471746 | 0.787423299 | 0.53591117 | 0.550825313 | 0.399227297 | 0.556140861 |
| TCGA_TCGA-78-7161 | 0.132685875 | 0.369265316 | 0.425637966 | 0.569983728 | 0.550427907 | 0.753346224 | 0.369921536 | 0.523901736 | 0.298217264 | 0.58978429 | 0.554991159 | 0.319995065 | 0.450792657 | 0.736184877 | 0.366268339 | 0.591016534 | 0.249423602 | 0.615042254 | 0.411247122 | 0.465168644 | 0.400693072 | 0.370881033 | 0.565764598 |
| TCGA_TCGA-44-3398 | 0.307397556 | 0.512866129 | 0.562817165 | 0.741399046 | 0.68015609 | 0.689798863 | 0.358895085 | 0.726851942 | 0.495055462 | 0.765335035 | 0.871486719 | 0.487155088 | 0.58650166 | 0.765597503 | 0.540462615 | 0.715440979 | 0.302644962 | 0.741857592 | 0.816806634 | 0.594797183 | 0.611337675 | 0.407738627 | 0.568505571 |
| TCGA_TCGA-97-8547 | 0.129704175 | 0.373285718 | 0.435924618 | 0.675721785 | 0.598618527 | 0.703727592 | 0.185932584 | 0.616750525 | 0.37381925 | 0.65184388 | 0.761581941 | 0.430867712 | 0.492445305 | 0.771861362 | 0.476143247 | 0.656963964 | 0.19061603 | 0.698266841 | 0.696675835 | 0.576965049 | 0.5394795 | 0.445303164 | 0.454689049 |
| TCGA_TCGA-86-8280 | 0.405798896 | 0.468501603 | 0.563888323 | 0.697537518 | 0.580143543 | 0.6300974 | 0.379377404 | 0.631503547 | 0.527781763 | 0.703532269 | 0.804723263 | 0.437611704 | 0.545553126 | 0.769405409 | 0.478817721 | 0.665557513 | 0.164997582 | 0.704544879 | 0.687396652 | 0.598869262 | 0.548205985 | 0.424831601 | 0.480450089 |
| TCGA_TCGA-78-7166 | 0.315824692 | 0.504994877 | 0.541550133 | 0.622340246 | 0.608367264 | 0.714234751 | 0.195288607 | 0.586371427 | 0.358482473 | 0.641240852 | 0.636164322 | 0.372180486 | 0.432391352 | 0.767461709 | 0.411555701 | 0.566151203 | 0.288094147 | 0.642953401 | 0.426906953 | 0.454957904 | 0.464951792 | 0.390938088 | 0.498451129 |
| TCGA_TCGA-91-8499 | 0.239670693 | 0.568762133 | 0.583026553 | 0.601271749 | 0.607099123 | 0.664976668 | 0.304663731 | 0.657228501 | 0.41452864 | 0.635445649 | 0.664334402 | 0.394139167 | 0.362334615 | 0.700649425 | 0.409031147 | 0.662139744 | 0.029786094 | 0.64937165 | 0.5780724 | 0.544998378 | 0.482070433 | 0.372755257 | 0.570116597 |
| TCGA_TCGA-71-6725 | 0.139854347 | 0.382988494 | 0.424248402 | 0.608249022 | 0.608652881 | 0.656219091 | 0.300314453 | 0.555051911 | 0.382684869 | 0.623015364 | 0.646970005 | 0.338060628 | 0.487181405 | 0.755933104 | 0.403546441 | 0.656388732 | 0.230740352 | 0.638752771 | 0.509662352 | 0.547629957 | 0.455502314 | 0.394404635 | 0.491499378 |
| TCGA_TCGA-69-7980 | 0.249448538 | 0.476433466 | 0.489963121 | 0.672857039 | 0.57764269 | 0.667977306 | 0.391140072 | 0.59361383 | 0.45320611 | 0.664703928 | 0.736533171 | 0.441131213 | 0.488067317 | 0.74655447 | 0.44413011 | 0.651378934 | 0.130477336 | 0.675074868 | 0.610475689 | 0.548914559 | 0.521904251 | 0.438925587 | 0.469107375 |
| TCGA_TCGA-L9-A8F4 | 0.427046612 | 0.572379969 | 0.651308003 | 0.651494935 | 0.569470217 | 0.677191056 | 0.290631504 | 0.621361228 | 0.570033483 | 0.636423893 | 0.842497718 | 0.440560941 | 0.423889602 | 0.771624133 | 0.472299203 | 0.622206882 | 0.202847246 | 0.67640006 | 0.667081032 | 0.538437149 | 0.512375913 | 0.412367905 | 0.371949987 |
| TCGA_TCGA-05-4433 | 0.274640012 | 0.46966806 | 0.535126368 | 0.680943315 | 0.598921447 | 0.714367697 | 0.386391002 | 0.635131565 | 0.51073379 | 0.683346437 | 0.812592152 | 0.443592428 | 0.51078245 | 0.792564965 | 0.508260119 | 0.65654302 | 0.285402255 | 0.731772005 | 0.63372681 | 0.56577785 | 0.532696844 | 0.466536669 | 0.563618681 |
| TCGA_TCGA-55-A492 | 0.268184854 | 0.291130912 | 0.470172028 | 0.608765979 | 0.546172758 | 0.639621672 | 0.29466047 | 0.597509003 | 0.339905877 | 0.654740085 | 0.596869333 | 0.414819436 | 0.464870117 | 0.728180095 | 0.384945064 | 0.583004068 | 0.272490716 | 0.674473308 | 0.482924602 | 0.493671483 | 0.448317038 | 0.407404267 | 0.444274066 |
| TCGA_TCGA-73-4675 | 0.229146609 | 0.364848068 | 0.408472572 | 0.658794662 | 0.552254642 | 0.719081165 | 0.263738696 | 0.606136115 | 0.41717537 | 0.676219192 | 0.658760307 | 0.444268379 | 0.550653724 | 0.769059942 | 0.434347022 | 0.599329381 | 0.125534868 | 0.676180278 | 0.583786159 | 0.53661357 | 0.49307031 | 0.407544374 | 0.45149122 |
| TCGA_TCGA-50-5936 | 0.283865041 | 0.490186262 | 0.446902073 | 0.6413577 | 0.639296919 | 0.672568104 | 0.195155363 | 0.68314411 | 0.417079749 | 0.707697169 | 0.748975787 | 0.413763836 | 0.501086833 | 0.772603506 | 0.463724707 | 0.62253335 | 0.284033386 | 0.678209783 | 0.668553355 | 0.513922509 | 0.508977527 | 0.386007599 | 0.552695388 |
| TCGA_TCGA-44-7662 | 0.286392226 | 0.545211159 | 0.528248177 | 0.661832278 | 0.604921946 | 0.69727586 | 0.24823751 | 0.678420229 | 0.497757427 | 0.680017 | 0.787217384 | 0.437299824 | 0.531820441 | 0.750682413 | 0.569481105 | 0.691587515 | 0.218905965 | 0.666196422 | 0.711849488 | 0.547125491 | 0.536821299 | 0.353970299 | 0.494010266 |
| TCGA_TCGA-78-7154 | 0.20766623 | 0.615277925 | 0.473911506 | 0.588345524 | 0.602043469 | 0.653875804 | 0.32378795 | 0.563532433 | 0.41049448 | 0.7010744 | 0.538178669 | 0.315620888 | 0.364552957 | 0.742478113 | 0.496723113 | 0.562645846 | 0.109374201 | 0.640864517 | 0.529405794 | 0.491542856 | 0.475009122 | 0.360967262 | 0.537309689 |
| TCGA_TCGA-05-4244 | 0.157924449 | 0.510100077 | 0.469043075 | 0.694521029 | 0.627580529 | 0.693107164 | 0.295754833 | 0.648023133 | 0.433479 | 0.721998479 | 0.774775844 | 0.391494064 | 0.549100033 | 0.772532983 | 0.453996063 | 0.645249366 | 0.257511136 | 0.671888031 | 0.70077689 | 0.540116594 | 0.507139834 | 0.390639219 | 0.485972304 |
| TCGA_TCGA-73-7498 | 0.34964184 | 0.3218553 | 0.45577784 | 0.623693284 | 0.531936762 | 0.625866649 | 0.320031855 | 0.5724823 | 0.443760949 | 0.644578344 | 0.714060532 | 0.440011399 | 0.527055363 | 0.734560107 | 0.410059548 | 0.619074736 | 0.197787937 | 0.692054365 | 0.566050599 | 0.563597597 | 0.478996552 | 0.421155488 | 0.489664373 |
| TCGA_TCGA-50-5933 | 0.246879135 | 0.566500782 | 0.480814195 | 0.692245767 | 0.64644971 | 0.686107214 | 0.282553948 | 0.703968989 | 0.509730067 | 0.670764015 | 0.840121054 | 0.48360959 | 0.551667151 | 0.757610595 | 0.610254568 | 0.706458943 | 0.240527243 | 0.71930375 | 0.808441296 | 0.61035706 | 0.586066039 | 0.410636027 | 0.474995907 |
| TCGA_TCGA-55-8507 | 0.15388862 | 0.444135201 | 0.434376994 | 0.622993237 | 0.534944632 | 0.679533551 | 0.282213544 | 0.613994906 | 0.416575002 | 0.696716354 | 0.673675922 | 0.414395303 | 0.520237644 | 0.747656455 | 0.419331256 | 0.652849692 | 0.20839347 | 0.691570933 | 0.571506211 | 0.520826303 | 0.465171611 | 0.346770981 | 0.522084984 |
| TCGA_TCGA-S2-AA1A | 0.656922786 | 0.502202617 | 0.626137033 | 0.686222395 | 0.573707838 | 0.684562816 | 0.482133049 | 0.565335039 | 0.696698077 | 0.594254897 | 0.817351841 | 0.398754006 | 0.547501707 | 0.786315161 | 0.463959931 | 0.649500423 | 0.101013158 | 0.70817971 | 0.622618419 | 0.563806109 | 0.60598048 | 0.441235584 | 0.531206535 |
| TCGA_TCGA-95-7948 | 0.218571555 | 0.349192072 | 0.447268946 | 0.648651259 | 0.563968949 | 0.640229389 | 0.381328339 | 0.558643223 | 0.422431929 | 0.641227396 | 0.613614907 | 0.308920578 | 0.473330633 | 0.75572958 | 0.372123841 | 0.628903143 | 0.199831298 | 0.621301295 | 0.437166471 | 0.500198306 | 0.481634086 | 0.396934509 | 0.377600348 |
| TCGA_TCGA-67-3774 | 0.313181455 | 0.463262709 | 0.511958031 | 0.709486578 | 0.59842977 | 0.660493048 | 0.425337678 | 0.619727679 | 0.464911007 | 0.6808393 | 0.752083496 | 0.43878063 | 0.552236751 | 0.756267501 | 0.447022686 | 0.63472936 | 0.246888289 | 0.703597134 | 0.616658067 | 0.559931742 | 0.531237691 | 0.380867236 | 0.506410994 |
| TCGA_TCGA-38-4628 | 0.092735522 | 0.34368798 | 0.421545898 | 0.661832251 | 0.646867746 | 0.720841784 | 0.175523724 | 0.688329079 | 0.414976216 | 0.765297539 | 0.704380397 | 0.4353103 | 0.511990855 | 0.787510456 | 0.509021548 | 0.645603553 | 0.209534646 | 0.697051114 | 0.628832683 | 0.567204877 | 0.570481169 | 0.438568662 | 0.475863767 |
| TCGA_TCGA-50-6595 | 0.231900295 | 0.589806798 | 0.536102063 | 0.640220748 | 0.644435713 | 0.677323931 | 0.266638521 | 0.699695357 | 0.493208808 | 0.667927574 | 0.771160418 | 0.420376103 | 0.454471711 | 0.747734038 | 0.561887953 | 0.714835348 | 0.271703523 | 0.670799951 | 0.752971499 | 0.5581797 | 0.562714765 | 0.43082983 | 0.552981099 |
| TCGA_TCGA-55-7903 | 0.224149373 | 0.532724829 | 0.564421723 | 0.670007736 | 0.651926695 | 0.70181111 | 0.260619359 | 0.609749126 | 0.445140859 | 0.720724115 | 0.717800011 | 0.356243406 | 0.577208098 | 0.746530699 | 0.428007709 | 0.634425184 | 0.217256599 | 0.647668278 | 0.597262877 | 0.532786241 | 0.485630666 | 0.379086648 | 0.548474975 |
| TCGA_TCGA-44-3396 | 0.386233215 | 0.633383675 | 0.570272349 | 0.720811755 | 0.599464404 | 0.644116657 | 0.359814031 | 0.686058149 | 0.547814135 | 0.692999432 | 0.860585109 | 0.462109561 | 0.523201793 | 0.763841176 | 0.520467586 | 0.721829543 | 0.260582697 | 0.710331233 | 0.78723573 | 0.595020789 | 0.568159373 | 0.44123707 | 0.496259564 |
| TCGA_TCGA-80-5611 | 0.201160551 | 0.5414879 | 0.487260093 | 0.698685096 | 0.669215848 | 0.673734659 | 0.213173932 | 0.649930611 | 0.523768938 | 0.735623056 | 0.877593837 | 0.47771037 | 0.485359248 | 0.737867227 | 0.481433499 | 0.670122718 | 0.196824419 | 0.699300112 | 0.732426079 | 0.538111795 | 0.547908312 | 0.379219785 | 0.45439548 |
| TCGA_TCGA-53-7624 | 0.161970768 | 0.607337384 | 0.432794951 | 0.620056704 | 0.608639209 | 0.640847547 | 0.224997655 | 0.623901038 | 0.387879282 | 0.684280373 | 0.587865961 | 0.397346782 | 0.325060413 | 0.688994921 | 0.470980065 | 0.626035243 | 0.195914757 | 0.636605192 | 0.516793505 | 0.486873825 | 0.468029245 | 0.362798586 | 0.553615595 |
| TCGA_TCGA-91-6835 | 0.592697661 | 0.61959484 | 0.675583771 | 0.684708505 | 0.579691615 | 0.639873919 | 0.396123072 | 0.622993945 | 0.727316153 | 0.719144804 | 0.888383077 | 0.470916593 | 0.585738521 | 0.783380379 | 0.516073792 | 0.728661456 | 0.247578282 | 0.692163815 | 0.72872332 | 0.627550886 | 0.617360735 | 0.398593187 | 0.550893226 |
| TCGA_TCGA-L9-A50W | 0.261737079 | 0.349507382 | 0.453280869 | 0.689097299 | 0.59622247 | 0.656081904 | 0.289959409 | 0.546545231 | 0.396881568 | 0.674069828 | 0.678609597 | 0.398773865 | 0.568253382 | 0.750034154 | 0.386504882 | 0.696306008 | 0.247157071 | 0.675073053 | 0.46433843 | 0.588584405 | 0.506358574 | 0.496889744 | 0.483355187 |
| TCGA_TCGA-86-7713 | 0.197827742 | 0.459976267 | 0.418055362 | 0.525440127 | 0.552236141 | 0.720464638 | 0.359293159 | 0.554982942 | 0.378375203 | 0.655962967 | 0.567737453 | 0.318197167 | 0.440019013 | 0.725198011 | 0.368157077 | 0.576302756 | 0.23413036 | 0.609569442 | 0.492901068 | 0.488647976 | 0.467201009 | 0.356765222 | 0.671868306 |
| TCGA_TCGA-50-5044 | 0.275252503 | 0.540322252 | 0.568298727 | 0.655349051 | 0.620994032 | 0.632020242 | 0.275663944 | 0.703073352 | 0.4018936 | 0.64589924 | 0.717003553 | 0.440446091 | 0.451410144 | 0.75316127 | 0.551828703 | 0.684277061 | 0.187145909 | 0.710051403 | 0.580571416 | 0.568778886 | 0.517551951 | 0.404954599 | 0.505813592 |
| TCGA_TCGA-97-7941 | 0.27507174 | 0.351819372 | 0.474572341 | 0.595893929 | 0.576149904 | 0.711824506 | 0.295593901 | 0.56366279 | 0.415343603 | 0.697397386 | 0.697748012 | 0.416682855 | 0.456789129 | 0.789075069 | 0.43857465 | 0.654178089 | 0.204162001 | 0.721706152 | 0.614810287 | 0.531388206 | 0.514572195 | 0.487701886 | 0.490337885 |
| TCGA_TCGA-86-7714 | 0.192738355 | 0.407365319 | 0.405784154 | 0.717566002 | 0.604808329 | 0.62757408 | 0.258414742 | 0.538356178 | 0.441032831 | 0.679700107 | 0.683085084 | 0.404027272 | 0.506637203 | 0.753147684 | 0.411820012 | 0.692073755 | 0.271808396 | 0.691078057 | 0.60801342 | 0.538065139 | 0.492734907 | 0.415702722 | 0.548737046 |
| TCGA_TCGA-62-8402 | 0.240503629 | 0.583672126 | 0.639971382 | 0.64929909 | 0.607072513 | 0.708088519 | 0.281148124 | 0.602475153 | 0.483694398 | 0.639008991 | 0.730565477 | 0.433998652 | 0.4111354 | 0.755051631 | 0.415352532 | 0.690735497 | 0.198723571 | 0.674290125 | 0.627928471 | 0.556903568 | 0.520380077 | 0.389283366 | 0.519259294 |
| TCGA_TCGA-78-7162 | 0.375606822 | 0.451931926 | 0.579473106 | 0.635281601 | 0.571250137 | 0.603167981 | 0.434443804 | 0.664112643 | 0.475611684 | 0.690920376 | 0.694637992 | 0.424156676 | 0.617784687 | 0.761694715 | 0.457256881 | 0.643701645 | 0.13487161 | 0.700568475 | 0.601159714 | 0.573907847 | 0.517752587 | 0.396481418 | 0.536053688 |
| TCGA_TCGA-49-AAR0 | 0.381279648 | 0.463700695 | 0.561672509 | 0.632344468 | 0.605656627 | 0.700780036 | 0.40989115 | 0.628561461 | 0.479835752 | 0.703313652 | 0.832404994 | 0.443784467 | 0.462905253 | 0.761994876 | 0.469127825 | 0.652995634 | 0.15838123 | 0.705710516 | 0.622841684 | 0.582104909 | 0.504664483 | 0.372202389 | 0.471550419 |
| TCGA_TCGA-35-4122 | 0.384004641 | 0.661623374 | 0.709198968 | 0.750507576 | 0.675149727 | 0.678923627 | 0.352993849 | 0.750336075 | 0.58794039 | 0.737785909 | 0.954614767 | 0.545358121 | 0.614283179 | 0.76939764 | 0.592921693 | 0.701800641 | 0.245632054 | 0.707064451 | 0.812774235 | 0.650361988 | 0.634942361 | 0.441161813 | 0.498278279 |
| TCGA_TCGA-55-7726 | 0.242638422 | 0.534792447 | 0.402229131 | 0.67811295 | 0.658950292 | 0.692201135 | 0.295982241 | 0.629152884 | 0.468064402 | 0.670357473 | 0.739271608 | 0.421509867 | 0.460183474 | 0.670285273 | 0.54525542 | 0.665951472 | 0.192523571 | 0.703779836 | 0.669427183 | 0.534439423 | 0.548968558 | 0.396815658 | 0.535698746 |
| TCGA_TCGA-62-A46Y | 0.280877049 | 0.472354506 | 0.527795696 | 0.734732673 | 0.611184851 | 0.64668085 | 0.315965364 | 0.638359468 | 0.479703865 | 0.795822157 | 0.838150284 | 0.471587925 | 0.491552289 | 0.783588364 | 0.44619099 | 0.696071444 | 0.214271798 | 0.730970538 | 0.698767369 | 0.576439717 | 0.517816694 | 0.390488852 | 0.514063007 |
| TCGA_TCGA-55-6980 | 0.236319687 | 0.435731322 | 0.550347346 | 0.70743628 | 0.630368133 | 0.69159197 | 0.413314976 | 0.591003651 | 0.459110776 | 0.711295104 | 0.834507741 | 0.464390877 | 0.57664448 | 0.82511124 | 0.484876761 | 0.726405907 | 0.25134875 | 0.739602644 | 0.692546582 | 0.591434712 | 0.575676521 | 0.425574852 | 0.478705711 |
| TCGA_TCGA-05-4430 | 0.261131986 | 0.444040232 | 0.504811353 | 0.679562343 | 0.63478579 | 0.64710858 | 0.306970581 | 0.681563876 | 0.443841204 | 0.732415268 | 0.813696705 | 0.475454262 | 0.621409648 | 0.780959979 | 0.486061397 | 0.706391671 | 0.172857781 | 0.700233641 | 0.732282769 | 0.634719496 | 0.53091637 | 0.39548525 | 0.487424992 |
| TCGA_TCGA-67-3772 | 0.391940107 | 0.445071521 | 0.534910004 | 0.685853242 | 0.614025709 | 0.649904051 | 0.278883588 | 0.685233484 | 0.501337209 | 0.693106157 | 0.779982487 | 0.424257779 | 0.616542903 | 0.778517131 | 0.483263098 | 0.654173781 | 0.181913991 | 0.705411252 | 0.681110707 | 0.557131879 | 0.545554295 | 0.375386829 | 0.519604758 |
| TCGA_TCGA-MP-A4SY | 0.127443796 | 0.432111534 | 0.41016127 | 0.654869161 | 0.614300041 | 0.721007263 | 0.204371791 | 0.614248934 | 0.395613051 | 0.683191694 | 0.713791007 | 0.403522484 | 0.452339426 | 0.752471654 | 0.472930528 | 0.655556979 | 0.247834221 | 0.659774872 | 0.689850205 | 0.526528923 | 0.516570445 | 0.35909858 | 0.449743751 |
| TCGA_TCGA-78-7148 | 0.222519126 | 0.513937502 | 0.553078894 | 0.623696954 | 0.639182991 | 0.676896798 | 0.217130312 | 0.607916601 | 0.400704627 | 0.666211486 | 0.670816397 | 0.364748148 | 0.424490013 | 0.779535432 | 0.447496637 | 0.599319575 | 0.345319353 | 0.653199061 | 0.518220223 | 0.503482662 | 0.472010407 | 0.402191308 | 0.594498044 |
| TCGA_TCGA-35-3615 | 0.280917608 | 0.341509371 | 0.467103293 | 0.642653837 | 0.571299133 | 0.6336087 | 0.288376574 | 0.591205607 | 0.457347378 | 0.634723623 | 0.651194392 | 0.421409677 | 0.496305986 | 0.730121484 | 0.395598462 | 0.593897903 | 0.191171574 | 0.674187005 | 0.578890135 | 0.549456073 | 0.486667807 | 0.368827975 | 0.510311835 |
| TCGA_TCGA-55-A493 | 0.371041642 | 0.687308715 | 0.604338766 | 0.679965856 | 0.582539809 | 0.697860717 | 0.253245471 | 0.672150235 | 0.53732347 | 0.662187443 | 0.863517518 | 0.447425036 | 0.417906958 | 0.75104477 | 0.550859609 | 0.654067395 | 0.164902163 | 0.699136734 | 0.780513369 | 0.60387911 | 0.570718259 | 0.432320418 | 0.452152956 |
| TCGA_TCGA-49-6744 | 0.412164648 | 0.566295011 | 0.601094255 | 0.732014177 | 0.63587235 | 0.670233155 | 0.377374264 | 0.679960167 | 0.541925455 | 0.728694848 | 0.901034205 | 0.479418745 | 0.614134231 | 0.786432534 | 0.525631056 | 0.726612727 | 0.252803117 | 0.710922839 | 0.749842386 | 0.594767794 | 0.574556966 | 0.425384388 | 0.525550319 |
| TCGA_TCGA-49-4494 | 0.206521312 | 0.551576054 | 0.576458472 | 0.700545872 | 0.63273453 | 0.662879184 | 0.19618925 | 0.673255013 | 0.387701393 | 0.719109598 | 0.743353963 | 0.396194193 | 0.436929437 | 0.763031341 | 0.449758524 | 0.631725294 | 0.160478815 | 0.625905914 | 0.649224835 | 0.522128222 | 0.525703346 | 0.351816353 | 0.53357174 |
| TCGA_TCGA-78-7143 | 0.253946163 | 0.530378747 | 0.399549092 | 0.692619374 | 0.585013198 | 0.690111987 | 0.268410888 | 0.624090555 | 0.474265122 | 0.685685553 | 0.673410802 | 0.391554677 | 0.470604416 | 0.766647214 | 0.429649831 | 0.663814557 | 0.206053506 | 0.664071932 | 0.611514692 | 0.533829759 | 0.492577252 | 0.398509693 | 0.56545101 |
| TCGA_TCGA-50-5051 | 0.283448514 | 0.500275437 | 0.458038219 | 0.645737835 | 0.628069595 | 0.771402451 | 0.35971298 | 0.538732727 | 0.370435938 | 0.671971842 | 0.594567245 | 0.301916281 | 0.469256626 | 0.762837026 | 0.400635287 | 0.607949156 | 0.233231085 | 0.651015576 | 0.448284262 | 0.463297732 | 0.447160498 | 0.380928274 | 0.540801461 |
| TCGA_TCGA-86-6851 | 0.526268047 | 0.585993892 | 0.688323421 | 0.721545948 | 0.540782092 | 0.715087751 | 0.280555528 | 0.59669369 | 0.632224777 | 0.679059995 | 0.899311812 | 0.460285714 | 0.545760406 | 0.742357917 | 0.511423857 | 0.703906955 | 0.103883381 | 0.713583826 | 0.651239233 | 0.586082934 | 0.594240181 | 0.459447282 | 0.471830701 |
| TCGA_TCGA-75-6205 | 0.440682133 | 0.633357857 | 0.658619391 | 0.65247683 | 0.628168662 | 0.686453451 | 0.393419 | 0.669274987 | 0.599951821 | 0.717568853 | 0.918124019 | 0.528904403 | 0.596193098 | 0.766064468 | 0.570785251 | 0.727422451 | 0.146110024 | 0.72298927 | 0.776270386 | 0.641968354 | 0.62420237 | 0.43718414 | 0.561206682 |
| TCGA_TCGA-49-6743 | 0.275629477 | 0.635027788 | 0.427936473 | 0.640105002 | 0.632779224 | 0.685206299 | 0.259012139 | 0.62115453 | 0.468120228 | 0.6695559 | 0.763968017 | 0.366034885 | 0.457503119 | 0.75391303 | 0.47628715 | 0.670490326 | 0.356176978 | 0.64373874 | 0.679456301 | 0.528784807 | 0.531112158 | 0.382831241 | 0.492526128 |
| TCGA_TCGA-50-6597 | 0.450934387 | 0.460100067 | 0.634425628 | 0.656883241 | 0.668999726 | 0.738952537 | 0.252557761 | 0.591485222 | 0.472022702 | 0.733163487 | 0.734272206 | 0.398942076 | 0.415585549 | 0.79148259 | 0.419887363 | 0.617932203 | 0.298916222 | 0.700680442 | 0.479042489 | 0.561760569 | 0.534300803 | 0.443226587 | 0.513293343 |
| TCGA_TCGA-55-A490 | 0.283592376 | 0.416063596 | 0.505759154 | 0.625833595 | 0.599427945 | 0.711897012 | 0.237098004 | 0.621397426 | 0.430220334 | 0.625326256 | 0.782527175 | 0.410133753 | 0.544832652 | 0.779193989 | 0.48719134 | 0.599255716 | 0.235112042 | 0.64488784 | 0.652363793 | 0.545311298 | 0.46888569 | 0.400251581 | 0.459101642 |
| TCGA_TCGA-99-8032 | 0.374829075 | 0.489613687 | 0.463045131 | 0.639464788 | 0.624534102 | 0.741262333 | 0.296647089 | 0.613751995 | 0.465115732 | 0.7024153 | 0.718614341 | 0.358583749 | 0.532182354 | 0.742649563 | 0.44801094 | 0.664296388 | 0.240620281 | 0.692016232 | 0.577932166 | 0.510702134 | 0.49597539 | 0.375509899 | 0.547143108 |
| TCGA_TCGA-55-8299 | 0.438640829 | 0.600625091 | 0.692651834 | 0.694456353 | 0.664355847 | 0.629061609 | 0.342182674 | 0.670505628 | 0.510290738 | 0.724746734 | 0.919540553 | 0.51185998 | 0.611136784 | 0.780837134 | 0.51970062 | 0.704107887 | 0.228370421 | 0.725759142 | 0.722983626 | 0.582261766 | 0.593237794 | 0.412510008 | 0.593277357 |
| TCGA_TCGA-64-1676 | 0.285457576 | 0.520312674 | 0.609772718 | 0.678364332 | 0.68168034 | 0.695836566 | 0.288014529 | 0.687051716 | 0.46765782 | 0.725891064 | 0.877398613 | 0.500243877 | 0.607862768 | 0.775673091 | 0.528328113 | 0.685533335 | 0.203537743 | 0.689624367 | 0.728181589 | 0.619378806 | 0.553008204 | 0.379209468 | 0.50958903 |
| TCGA_TCGA-86-8279 | 0.128595717 | 0.366201717 | 0.383945802 | 0.644104278 | 0.608846419 | 0.700949931 | 0.264532268 | 0.609289688 | 0.35376242 | 0.720386266 | 0.641685612 | 0.374738472 | 0.559579892 | 0.727943362 | 0.435990955 | 0.656852141 | 0.177892039 | 0.648360127 | 0.574032484 | 0.508502882 | 0.44291535 | 0.371218154 | 0.503564028 |
| TCGA_TCGA-97-A4M5 | 0.312116345 | 0.375226922 | 0.49031923 | 0.648871059 | 0.622382266 | 0.683589383 | 0.370835749 | 0.636662294 | 0.499662922 | 0.681950188 | 0.725831324 | 0.484972659 | 0.607856919 | 0.763476674 | 0.453253158 | 0.658791488 | 0.187827269 | 0.707571284 | 0.619666302 | 0.546675396 | 0.509786712 | 0.353345731 | 0.519624436 |
| TCGA_TCGA-78-7536 | 0.19935617 | 0.605491327 | 0.498486675 | 0.655299021 | 0.647377733 | 0.68526887 | 0.251258233 | 0.583154905 | 0.426327287 | 0.807379619 | 0.762940112 | 0.409459744 | 0.398367363 | 0.711555717 | 0.421779293 | 0.63140572 | 0.188922791 | 0.654126763 | 0.653322807 | 0.507294217 | 0.45654331 | 0.324814951 | 0.467147774 |
| TCGA_TCGA-55-8208 | 0.604664789 | 0.639619192 | 0.654146231 | 0.700963301 | 0.65682629 | 0.705892454 | 0.299283452 | 0.686676344 | 0.666695966 | 0.717932224 | 0.966621287 | 0.536228361 | 0.539866739 | 0.788240681 | 0.543066304 | 0.739571255 | 0.183820051 | 0.740628925 | 0.795733805 | 0.643523057 | 0.617134695 | 0.442064791 | 0.495398041 |
| TCGA_TCGA-95-7562 | 0.200137489 | 0.543177928 | 0.458647206 | 0.665986827 | 0.620651609 | 0.581715321 | 0.274555052 | 0.602832576 | 0.453184819 | 0.689716475 | 0.771095982 | 0.356703187 | 0.461392766 | 0.732726203 | 0.419968705 | 0.644868714 | 0.153855877 | 0.659450644 | 0.621885618 | 0.565665299 | 0.496955994 | 0.309677532 | 0.555903161 |
| TCGA_TCGA-MP-A4TK | 0.420234348 | 0.52907492 | 0.486359853 | 0.683740374 | 0.63609336 | 0.692564478 | 0.254649709 | 0.684366604 | 0.526826356 | 0.666631662 | 0.830593375 | 0.500497258 | 0.544989545 | 0.746818432 | 0.527049623 | 0.653507571 | 0.185151396 | 0.70382324 | 0.71100543 | 0.559685314 | 0.563918145 | 0.391795387 | 0.515651671 |
| TCGA_TCGA-49-6761 | 0.356422043 | 0.56377024 | 0.581529747 | 0.630020547 | 0.642682517 | 0.719907701 | 0.325112373 | 0.636725483 | 0.491788156 | 0.641289381 | 0.759241656 | 0.36327257 | 0.538825937 | 0.742452894 | 0.503793158 | 0.623625783 | 0.226817017 | 0.64207844 | 0.639385363 | 0.540756869 | 0.541352402 | 0.335567427 | 0.465773085 |
| TCGA_TCGA-50-6593 | 0.218133425 | 0.447238959 | 0.404156914 | 0.668878769 | 0.598580712 | 0.715189496 | 0.292594436 | 0.627108085 | 0.448758292 | 0.752542458 | 0.769326554 | 0.450818062 | 0.548913245 | 0.764943726 | 0.519263066 | 0.718087571 | 0.255013681 | 0.700481353 | 0.669576403 | 0.588009356 | 0.531740584 | 0.423744101 | 0.467528826 |
| TCGA_TCGA-55-7728 | 0.463574147 | 0.482236712 | 0.544845578 | 0.736120895 | 0.610265989 | 0.757130279 | 0.423068426 | 0.68968987 | 0.626838559 | 0.729570837 | 0.9790897 | 0.568972494 | 0.538029841 | 0.825096898 | 0.509829102 | 0.69216492 | 0.32346385 | 0.74643804 | 0.712408796 | 0.604231251 | 0.551388326 | 0.498743686 | 0.492444608 |
| TCGA_TCGA-86-8055 | 0.266136689 | 0.499417841 | 0.424314592 | 0.64987141 | 0.614018896 | 0.670547768 | 0.233489925 | 0.653667092 | 0.499661759 | 0.698908838 | 0.758170266 | 0.411923354 | 0.522033741 | 0.741518762 | 0.528433078 | 0.675389484 | 0.13115277 | 0.685461706 | 0.733614194 | 0.546138911 | 0.565492986 | 0.403597927 | 0.530758381 |
| TCGA_TCGA-73-4668 | 0.220958215 | 0.44818743 | 0.419225227 | 0.659238977 | 0.614604026 | 0.678641415 | 0.302473354 | 0.582986618 | 0.400638728 | 0.760857668 | 0.656904104 | 0.302556661 | 0.602189616 | 0.748013363 | 0.482467711 | 0.652304399 | 0.253406681 | 0.678742261 | 0.576515457 | 0.525446977 | 0.497032843 | 0.350450072 | 0.511061491 |
| TCGA_TCGA-55-8090 | 0.232343418 | 0.461729704 | 0.425940803 | 0.64212492 | 0.631976153 | 0.710075662 | 0.321837616 | 0.624732128 | 0.432160866 | 0.698400685 | 0.699872026 | 0.391132818 | 0.52606869 | 0.778682707 | 0.468772473 | 0.660172408 | 0.187302504 | 0.697192863 | 0.683573263 | 0.524574327 | 0.500522574 | 0.412107011 | 0.541722044 |
| TCGA_TCGA-38-7271 | 0.780974483 | 0.645799261 | 0.743563689 | 0.695458034 | 0.59283457 | 0.664211707 | 0.490384459 | 0.639074556 | 0.818606981 | 0.692254442 | 0.913507294 | 0.517330988 | 0.689330896 | 0.826687253 | 0.533919633 | 0.719822033 | 0.184377464 | 0.705501262 | 0.755433643 | 0.63679531 | 0.636269265 | 0.370790023 | 0.5954346 |
| TCGA_TCGA-55-8514 | 0.205888893 | 0.346933053 | 0.458764086 | 0.630977489 | 0.57078857 | 0.761909057 | 0.406465067 | 0.545553546 | 0.441472166 | 0.710501233 | 0.715755557 | 0.388440248 | 0.466230385 | 0.78305648 | 0.417239818 | 0.640935439 | 0.183878339 | 0.688186815 | 0.57343375 | 0.546552617 | 0.472903971 | 0.367235433 | 0.490191135 |
| TCGA_TCGA-50-5932 | 0.180374071 | 0.33061821 | 0.411957333 | 0.586964277 | 0.567708734 | 0.651438138 | 0.258526051 | 0.593023411 | 0.449341833 | 0.655334951 | 0.579777702 | 0.379860616 | 0.423777369 | 0.745103976 | 0.382260338 | 0.599371367 | 0.213593228 | 0.659146675 | 0.513856822 | 0.518819976 | 0.447286989 | 0.376116906 | 0.464306234 |
| TCGA_TCGA-95-A4VN | 0.331249952 | 0.655488842 | 0.618435125 | 0.653202864 | 0.584211019 | 0.716650422 | 0.349538659 | 0.648854978 | 0.510071452 | 0.727793946 | 0.876957046 | 0.434258412 | 0.501446673 | 0.775412647 | 0.497413248 | 0.674370779 | 0.281467869 | 0.69024248 | 0.696150314 | 0.593383243 | 0.546285384 | 0.402805206 | 0.527783984 |
| TCGA_TCGA-86-7954 | 0.316253487 | 0.531336005 | 0.542528163 | 0.685975624 | 0.673430875 | 0.712301004 | 0.314613995 | 0.61350989 | 0.496823914 | 0.698035795 | 0.872543642 | 0.480912899 | 0.461408465 | 0.760658449 | 0.46507083 | 0.703323764 | 0.163181858 | 0.671281454 | 0.704601225 | 0.599957256 | 0.530132339 | 0.399916994 | 0.484216788 |
| TCGA_TCGA-91-A4BC | 0.485203123 | 0.616166593 | 0.778030935 | 0.671069909 | 0.575609023 | 0.647367228 | 0.294315574 | 0.617507672 | 0.538036855 | 0.676872806 | 0.881858354 | 0.44404829 | 0.354815836 | 0.761375196 | 0.520103141 | 0.69269368 | 0.182300814 | 0.687339336 | 0.596297443 | 0.567632903 | 0.569040137 | 0.331601963 | 0.494262066 |
| TCGA_TCGA-MP-A4TJ | 0.617051721 | 0.676109619 | 0.72336299 | 0.708450317 | 0.596284005 | 0.721466572 | 0.484065838 | 0.613565422 | 0.689678159 | 0.721514958 | 0.896058596 | 0.469971653 | 0.566681316 | 0.792423695 | 0.493442968 | 0.717140347 | 0.295655794 | 0.709007114 | 0.736530125 | 0.602962042 | 0.598148235 | 0.400129533 | 0.531867013 |
| TCGA_TCGA-75-5126 | 0.273918883 | 0.611131835 | 0.575002064 | 0.734763197 | 0.665656092 | 0.657032029 | 0.353124315 | 0.757719422 | 0.5133307 | 0.767093585 | 0.924756777 | 0.586255748 | 0.626796551 | 0.797819114 | 0.563576614 | 0.735373545 | 0.340807998 | 0.72898951 | 0.830671323 | 0.639081974 | 0.603833191 | 0.406378209 | 0.555641073 |
| TCGA_TCGA-75-6211 | 0.308938313 | 0.532379251 | 0.506899744 | 0.600818079 | 0.585409222 | 0.728174375 | 0.240387482 | 0.54407413 | 0.436759573 | 0.700200377 | 0.59451798 | 0.343339827 | 0.427217181 | 0.74730843 | 0.4120179 | 0.615976837 | 0.233541276 | 0.646795561 | 0.546266716 | 0.5184386 | 0.43567885 | 0.334949104 | 0.504157618 |
| TCGA_TCGA-62-A470 | 0.138804678 | 0.454765288 | 0.441130476 | 0.631104033 | 0.582962415 | 0.723989781 | 0.398896279 | 0.587521684 | 0.409652341 | 0.657634031 | 0.618068155 | 0.413759019 | 0.473732511 | 0.701238874 | 0.410021196 | 0.609942073 | 0.172245705 | 0.670262569 | 0.563268681 | 0.564459434 | 0.436260188 | 0.391866296 | 0.544578956 |
| TCGA_TCGA-73-4677 | 0.310250371 | 0.427941005 | 0.473062486 | 0.63104784 | 0.551543915 | 0.616955661 | 0.415138584 | 0.642618919 | 0.452341891 | 0.657221716 | 0.693456171 | 0.420248646 | 0.525326657 | 0.745400385 | 0.426091671 | 0.605736758 | 0.265697333 | 0.650634173 | 0.575770473 | 0.534216793 | 0.494890263 | 0.387980615 | 0.517548552 |
| TCGA_TCGA-55-7911 | 0.309139366 | 0.695043551 | 0.670210329 | 0.671654382 | 0.619959179 | 0.665110048 | 0.336019895 | 0.628300757 | 0.528537342 | 0.702063942 | 0.780231248 | 0.390124723 | 0.386575116 | 0.726249071 | 0.443494704 | 0.657790136 | 0.22858497 | 0.659945918 | 0.619062587 | 0.545204962 | 0.50981334 | 0.396132352 | 0.610304225 |
| TCGA_TCGA-50-8460 | 0.42777619 | 0.4385705 | 0.541754311 | 0.696328926 | 0.683101172 | 0.655277434 | 0.294073431 | 0.62033562 | 0.503069364 | 0.742104959 | 0.799851548 | 0.503397375 | 0.613909608 | 0.790714657 | 0.398853916 | 0.658945682 | 0.199714262 | 0.741876505 | 0.620704208 | 0.589478977 | 0.518331565 | 0.446544498 | 0.473561669 |
| TCGA_TCGA-55-6981 | 0.308460972 | 0.422728011 | 0.429131253 | 0.651368524 | 0.639932938 | 0.635664013 | 0.282869756 | 0.614644655 | 0.460075549 | 0.70420345 | 0.648284346 | 0.350596317 | 0.52297381 | 0.768435551 | 0.435022699 | 0.641456455 | 0.178399562 | 0.672556656 | 0.585899403 | 0.545113493 | 0.505695521 | 0.402423721 | 0.52216153 |
| TCGA_TCGA-44-8120 | 0.254086683 | 0.385453453 | 0.427572866 | 0.631053955 | 0.573324797 | 0.605389826 | 0.343427757 | 0.582805674 | 0.464846772 | 0.662492993 | 0.647329063 | 0.402668161 | 0.566037568 | 0.760442894 | 0.411935799 | 0.689023363 | 0.169453381 | 0.67463797 | 0.60405353 | 0.549527266 | 0.495765075 | 0.399889005 | 0.528826651 |
| TCGA_TCGA-55-8614 | 0.250051893 | 0.43683222 | 0.402604743 | 0.615641975 | 0.626223372 | 0.619006542 | 0.43615505 | 0.551571958 | 0.406432267 | 0.687936216 | 0.609826104 | 0.356087902 | 0.468105648 | 0.71358747 | 0.426472155 | 0.660768319 | 0.135352771 | 0.655312087 | 0.543481997 | 0.518124618 | 0.460460822 | 0.360269407 | 0.505451612 |
| TCGA_TCGA-64-1677 | 0.389882722 | 0.672198558 | 0.635535504 | 0.654172984 | 0.65600918 | 0.663763537 | 0.235667314 | 0.642764262 | 0.489949586 | 0.672126239 | 0.735223621 | 0.348772289 | 0.450061673 | 0.708795397 | 0.420497761 | 0.649206976 | 0.184832491 | 0.646401257 | 0.555159011 | 0.488655644 | 0.483571597 | 0.342675558 | 0.534277672 |
| TCGA_TCGA-97-8552 | 0.399748774 | 0.344213009 | 0.473034772 | 0.693040211 | 0.54761306 | 0.641031531 | 0.400455772 | 0.631345939 | 0.480714209 | 0.722338179 | 0.819138906 | 0.473535179 | 0.548322379 | 0.788154872 | 0.436228003 | 0.669841309 | 0.291123567 | 0.737355157 | 0.624284535 | 0.581083061 | 0.509239528 | 0.464487589 | 0.442972262 |
| TCGA_TCGA-69-8253 | 0.363223018 | 0.433952441 | 0.491976624 | 0.642208051 | 0.562869776 | 0.658073217 | 0.332520545 | 0.609239314 | 0.438636919 | 0.676834664 | 0.647314048 | 0.381921294 | 0.564985565 | 0.760090623 | 0.405980319 | 0.580687328 | 0.266653672 | 0.664864426 | 0.535711323 | 0.527661348 | 0.488917714 | 0.351842529 | 0.47961079 |
| TCGA_TCGA-86-8668 | 0.403814454 | 0.394904642 | 0.450447419 | 0.667276124 | 0.518376522 | 0.633162047 | 0.316907935 | 0.580974923 | 0.498085931 | 0.63490157 | 0.725995885 | 0.451809765 | 0.49482065 | 0.76466532 | 0.47800688 | 0.688891374 | 0.174906657 | 0.694231127 | 0.622496212 | 0.588322096 | 0.527491114 | 0.445998232 | 0.483368092 |
| TCGA_TCGA-86-8074 | 0.212997678 | 0.457840996 | 0.421883839 | 0.678777232 | 0.629310082 | 0.697922016 | 0.267392539 | 0.630233661 | 0.451417217 | 0.671330042 | 0.750231282 | 0.401393766 | 0.507504629 | 0.754552893 | 0.478896912 | 0.682391935 | 0.163465805 | 0.69365511 | 0.689107561 | 0.563053732 | 0.532832489 | 0.372010767 | 0.498248339 |
| TCGA_TCGA-69-7765 | 0.349783844 | 0.504655699 | 0.491008431 | 0.694787152 | 0.615385358 | 0.640173508 | 0.309756277 | 0.617513884 | 0.529143211 | 0.73703122 | 0.766035053 | 0.418758537 | 0.566534564 | 0.723887158 | 0.512927702 | 0.686866771 | 0.202201101 | 0.720273755 | 0.657770892 | 0.566184405 | 0.548236771 | 0.355664917 | 0.559727787 |
| TCGA_TCGA-55-8092 | 0.538389451 | 0.562131048 | 0.631917244 | 0.707698581 | 0.573216425 | 0.665398208 | 0.289143209 | 0.581972382 | 0.588498806 | 0.652912874 | 0.792922938 | 0.409612875 | 0.419903742 | 0.757877278 | 0.474576906 | 0.653645508 | 0.203813501 | 0.674778475 | 0.571725823 | 0.496432079 | 0.545623194 | 0.430271273 | 0.50826382 |
| TCGA_TCGA-NJ-A4YQ | 0.631382819 | 0.59293082 | 0.735367792 | 0.651853971 | 0.556353901 | 0.661676451 | 0.309305168 | 0.624837165 | 0.687223434 | 0.666717902 | 0.916129799 | 0.413813996 | 0.431481226 | 0.79037663 | 0.490789744 | 0.638197843 | 0.385247289 | 0.690549318 | 0.712033244 | 0.557804729 | 0.596031356 | 0.38118307 | 0.432535053 |
| TCGA_TCGA-55-6983 | 0.559258947 | 0.519127352 | 0.620464543 | 0.656240685 | 0.635055047 | 0.696124919 | 0.320984008 | 0.591131098 | 0.581431138 | 0.67935882 | 0.749539361 | 0.402517919 | 0.511789345 | 0.791139223 | 0.453222882 | 0.684240738 | 0.160215277 | 0.684430627 | 0.623550533 | 0.561014981 | 0.544204893 | 0.319730086 | 0.540305863 |
| TCGA_TCGA-62-8394 | 0.18779547 | 0.531438246 | 0.443190891 | 0.59380398 | 0.600026281 | 0.694534286 | 0.307735318 | 0.623875632 | 0.41926407 | 0.677036325 | 0.666251221 | 0.350271201 | 0.422510661 | 0.714911543 | 0.466423961 | 0.659389791 | 0.149756441 | 0.645200452 | 0.550730046 | 0.521731286 | 0.523674287 | 0.318825072 | 0.541185295 |
| TCGA_TCGA-44-2661 | 0.528693942 | 0.550820043 | 0.69187777 | 0.750914111 | 0.652691569 | 0.65161815 | 0.445188392 | 0.691597713 | 0.623981437 | 0.743454686 | 0.942726601 | 0.537643918 | 0.670099868 | 0.801178967 | 0.534303632 | 0.735651332 | 0.219192349 | 0.764287525 | 0.800449774 | 0.647500347 | 0.607448319 | 0.440462719 | 0.517663548 |
| TCGA_TCGA-44-7659 | 0.335728376 | 0.384971166 | 0.434453974 | 0.654621034 | 0.591027035 | 0.654221665 | 0.437196296 | 0.577156751 | 0.496728684 | 0.693545915 | 0.709225628 | 0.413310535 | 0.482566759 | 0.730812765 | 0.389060498 | 0.629475727 | 0.213726856 | 0.663868776 | 0.514705989 | 0.536076689 | 0.479328632 | 0.439598819 | 0.460730506 |
| TCGA_TCGA-55-6712 | 0.424954558 | 0.623218178 | 0.619795114 | 0.693332206 | 0.620774478 | 0.649561153 | 0.3031419 | 0.675369373 | 0.588431719 | 0.681415175 | 0.825581605 | 0.401271772 | 0.587422945 | 0.797067291 | 0.535200399 | 0.698412976 | 0.24600033 | 0.695390812 | 0.70118634 | 0.568958738 | 0.570083269 | 0.412169477 | 0.524340549 |
| TCGA_TCGA-05-4403 | 0.234502155 | 0.400388937 | 0.514991911 | 0.660387679 | 0.629054372 | 0.744654234 | 0.337599547 | 0.680294878 | 0.47387909 | 0.707444523 | 0.790917728 | 0.452947717 | 0.540185878 | 0.795046519 | 0.501360183 | 0.671968662 | 0.288537351 | 0.725203148 | 0.717593525 | 0.591349489 | 0.541188119 | 0.488655009 | 0.519769683 |
| TCGA_TCGA-97-7553 | 0.402288675 | 0.56212624 | 0.571026882 | 0.740460223 | 0.621493876 | 0.593968605 | 0.492651704 | 0.699400806 | 0.587036639 | 0.74452667 | 0.917888901 | 0.570347919 | 0.715306659 | 0.826128443 | 0.532132685 | 0.760119869 | 0.28852775 | 0.7407982 | 0.747581402 | 0.650621816 | 0.582236438 | 0.384234532 | 0.557720278 |
| TCGA_TCGA-78-8655 | 0.267698618 | 0.415067912 | 0.534628722 | 0.705202296 | 0.614446156 | 0.693747679 | 0.26514223 | 0.62211511 | 0.465597153 | 0.702509702 | 0.81128141 | 0.447822384 | 0.577774706 | 0.76449865 | 0.46305016 | 0.695525015 | 0.203144603 | 0.697263407 | 0.655223648 | 0.553197356 | 0.515058214 | 0.412733946 | 0.503010043 |
| TCGA_TCGA-55-7724 | 0.387921314 | 0.61169077 | 0.51059422 | 0.696466033 | 0.676819019 | 0.570281183 | 0.398208037 | 0.66986581 | 0.590649741 | 0.73732448 | 0.800117217 | 0.464952884 | 0.598737802 | 0.703579296 | 0.535094106 | 0.698304035 | 0.193788098 | 0.752956131 | 0.706196482 | 0.566655183 | 0.544615569 | 0.410031491 | 0.599873382 |
| TCGA_TCGA-73-4659 | 0.208599193 | 0.474116678 | 0.434026227 | 0.654637285 | 0.576194162 | 0.730741024 | 0.372536534 | 0.67476975 | 0.407084221 | 0.719187019 | 0.754392903 | 0.478233354 | 0.523611258 | 0.763024495 | 0.463387326 | 0.632996758 | 0.33902982 | 0.692694477 | 0.689064104 | 0.557740973 | 0.498679399 | 0.351019679 | 0.582482868 |
| TCGA_TCGA-50-6673 | 0.27627939 | 0.419253818 | 0.409768812 | 0.636075159 | 0.638304033 | 0.676734967 | 0.332010054 | 0.637447908 | 0.426821672 | 0.653530255 | 0.717699651 | 0.393807091 | 0.458914167 | 0.774244045 | 0.520108746 | 0.652451142 | 0.167114842 | 0.667859729 | 0.584849001 | 0.549055814 | 0.531296274 | 0.405085476 | 0.488409054 |
| TCGA_TCGA-78-7542 | 0.230505867 | 0.533921554 | 0.520072177 | 0.61421974 | 0.649007353 | 0.658415607 | 0.209767026 | 0.633904664 | 0.46087788 | 0.662159505 | 0.632146278 | 0.30010249 | 0.390942971 | 0.741536307 | 0.490587321 | 0.673700289 | 0.24953429 | 0.658490387 | 0.52860344 | 0.473997257 | 0.505952532 | 0.380224307 | 0.557295696 |
| TCGA_TCGA-38-4625 | 0.310707471 | 0.658589248 | 0.551785605 | 0.664829993 | 0.591274994 | 0.658000781 | 0.38684986 | 0.665671914 | 0.544480352 | 0.637404567 | 0.762631506 | 0.479266302 | 0.480903741 | 0.791342092 | 0.497409512 | 0.668962435 | 0.287812296 | 0.689998968 | 0.692458745 | 0.545157444 | 0.534524185 | 0.394828185 | 0.505898604 |
| TCGA_TCGA-55-7570 | 0.225986777 | 0.521435238 | 0.486330646 | 0.55877291 | 0.569084453 | 0.621184532 | 0.320116904 | 0.589397614 | 0.39848646 | 0.607400116 | 0.504221082 | 0.267560255 | 0.393948551 | 0.703580331 | 0.413864119 | 0.521972207 | 0.021898552 | 0.623019421 | 0.442649826 | 0.427994677 | 0.40383375 | 0.312901557 | 0.550355335 |
| TCGA_TCGA-69-7979 | 0.146338722 | 0.402152019 | 0.409109712 | 0.632030027 | 0.564073345 | 0.646085282 | 0.242768185 | 0.567130701 | 0.3315699 | 0.638430141 | 0.607534706 | 0.304827924 | 0.494575777 | 0.70436713 | 0.427397103 | 0.589764895 | 0.180255188 | 0.641423771 | 0.52038807 | 0.508381029 | 0.453510203 | 0.367722873 | 0.495061471 |
| TCGA_TCGA-55-A4DF | 0.323178149 | 0.629936493 | 0.561956961 | 0.681591364 | 0.65710728 | 0.644813417 | 0.23134358 | 0.61694599 | 0.571924764 | 0.731943122 | 0.800017695 | 0.399291396 | 0.435514091 | 0.684800986 | 0.437932369 | 0.671656585 | 0.07180575 | 0.671440894 | 0.670551462 | 0.510270587 | 0.520093061 | 0.32157498 | 0.501950306 |
| TCGA_TCGA-67-3773 | 0.239770604 | 0.471296103 | 0.579601588 | 0.719173982 | 0.687786822 | 0.63993562 | 0.388825405 | 0.623365583 | 0.473471551 | 0.725470306 | 0.819740997 | 0.500806458 | 0.620139487 | 0.742465547 | 0.463179231 | 0.702521627 | 0.197114741 | 0.696989995 | 0.688282303 | 0.600469087 | 0.539659519 | 0.444163564 | 0.479062036 |
| TCGA_TCGA-55-7573 | 0.50328989 | 0.421649899 | 0.482430286 | 0.680659502 | 0.593575078 | 0.612353846 | 0.41827029 | 0.568333922 | 0.554646612 | 0.702465943 | 0.742707736 | 0.404681818 | 0.513454206 | 0.759676211 | 0.449366595 | 0.695313515 | 0.163529912 | 0.690094392 | 0.619227816 | 0.559691758 | 0.5138127 | 0.418234178 | 0.519901942 |
| TCGA_TCGA-50-5068 | 0.500677056 | 0.604539477 | 0.743928936 | 0.714440388 | 0.645853675 | 0.675371622 | 0.398899891 | 0.640768211 | 0.59076489 | 0.696306487 | 0.841483189 | 0.425987643 | 0.631494 | 0.767815952 | 0.495029829 | 0.715407106 | 0.192448378 | 0.672309573 | 0.74035418 | 0.606719344 | 0.58693402 | 0.420447184 | 0.571797012 |
| TCGA_TCGA-49-AARN | 0.404275834 | 0.382173721 | 0.448703096 | 0.653149331 | 0.629825575 | 0.69630465 | 0.272444785 | 0.543591077 | 0.463969538 | 0.666513673 | 0.69329163 | 0.367162948 | 0.482131058 | 0.749344875 | 0.411803489 | 0.674515808 | 0.177161064 | 0.654536847 | 0.496495325 | 0.508854137 | 0.502647131 | 0.415529847 | 0.518740534 |
| TCGA_TCGA-78-7150 | 0.2056301 | 0.503462559 | 0.406444462 | 0.56946913 | 0.567866906 | 0.68874891 | 0.217388742 | 0.604467996 | 0.299728768 | 0.661278908 | 0.563061488 | 0.346110532 | 0.428678473 | 0.708329183 | 0.465127532 | 0.571611399 | 0.261728583 | 0.621929775 | 0.491195547 | 0.484099513 | 0.48979027 | 0.364672812 | 0.576514485 |
| TCGA_TCGA-MP-A4TA | 0.234322185 | 0.548860133 | 0.507130733 | 0.679553821 | 0.597320649 | 0.648906716 | 0.239432142 | 0.630237808 | 0.429505088 | 0.667320775 | 0.771473683 | 0.398279965 | 0.451463861 | 0.734443503 | 0.493750255 | 0.61729471 | 0.27888817 | 0.661929629 | 0.560104944 | 0.462164323 | 0.518697768 | 0.366008276 | 0.493706226 |
| TCGA_TCGA-55-7907 | 0.286483278 | 0.487646674 | 0.554712676 | 0.655396179 | 0.580683179 | 0.752919151 | 0.243738445 | 0.611082527 | 0.471204007 | 0.681027151 | 0.711957695 | 0.410597295 | 0.499641498 | 0.745847376 | 0.499872389 | 0.649366859 | 0.236588395 | 0.679742389 | 0.619682443 | 0.564132837 | 0.574299535 | 0.387820852 | 0.532370854 |
| TCGA_TCGA-55-5899 | 0.182407556 | 0.550674816 | 0.548908969 | 0.61032479 | 0.602519211 | 0.731847268 | 0.238175499 | 0.657479772 | 0.395037982 | 0.608898552 | 0.672727554 | 0.325602493 | 0.386878418 | 0.772540501 | 0.457920366 | 0.603823823 | 0.053353302 | 0.691602773 | 0.639200452 | 0.521888553 | 0.473965651 | 0.396301451 | 0.481301988 |
| TCGA_TCGA-55-7574 | 0.649236612 | 0.628826443 | 0.638237949 | 0.686701741 | 0.575238884 | 0.660083303 | 0.416705764 | 0.623206545 | 0.718147027 | 0.680193929 | 0.826820156 | 0.451296157 | 0.530804878 | 0.764611677 | 0.490089132 | 0.68043037 | 0.203553343 | 0.722158074 | 0.641679684 | 0.586232938 | 0.594816574 | 0.374487315 | 0.518204272 |
| TCGA_TCGA-44-6148 | 0.342898336 | 0.328864048 | 0.412032298 | 0.628063393 | 0.586868558 | 0.571388306 | 0.498683723 | 0.53920025 | 0.459053366 | 0.684760196 | 0.69909261 | 0.397749681 | 0.594903634 | 0.742940921 | 0.422756891 | 0.672139915 | 0.208638204 | 0.744972582 | 0.57150983 | 0.531289948 | 0.476958725 | 0.454842059 | 0.561218083 |
| TCGA_TCGA-MN-A4N1 | 0.132814261 | 0.390674351 | 0.367763185 | 0.574976112 | 0.57456972 | 0.615453424 | 0.25068039 | 0.596227335 | 0.305226133 | 0.644822241 | 0.560171828 | 0.334544525 | 0.447977953 | 0.743084407 | 0.427895544 | 0.588208724 | 0.210795235 | 0.609888685 | 0.491936171 | 0.507960493 | 0.496504733 | 0.3609906 | 0.489058204 |
| TCGA_TCGA-05-5429 | 0.193317249 | 0.488737044 | 0.500026027 | 0.61575276 | 0.61448778 | 0.707254148 | 0.332061453 | 0.580695608 | 0.381935135 | 0.663662102 | 0.572981628 | 0.358448143 | 0.502498378 | 0.737127822 | 0.476825146 | 0.630622296 | 0.196667048 | 0.654979376 | 0.482374974 | 0.480225446 | 0.479910572 | 0.483607256 | 0.494236198 |
| TCGA_TCGA-91-6847 | 0.142810278 | 0.405384284 | 0.459542494 | 0.484110081 | 0.652664572 | 0.573828972 | 0.241005009 | 0.600192255 | 0.238355876 | 0.667883033 | 0.496103271 | 0.282599933 | 0.291688168 | 0.739426569 | 0.340678658 | 0.615881431 | 0.114869282 | 0.59798574 | 0.333075886 | 0.374686765 | 0.456847086 | 0.340034238 | 0.531331186 |
| TCGA_TCGA-55-8511 | 0.472246496 | 0.612166561 | 0.563783043 | 0.682373606 | 0.578399585 | 0.707485563 | 0.325340354 | 0.611177238 | 0.592969892 | 0.660007977 | 0.836361187 | 0.394282459 | 0.481516299 | 0.781410923 | 0.495658248 | 0.668725061 | 0.239326334 | 0.675866856 | 0.624166672 | 0.58310967 | 0.580332708 | 0.419810467 | 0.511916451 |
| TCGA_TCGA-50-5944 | 0.206645206 | 0.327094261 | 0.415212139 | 0.646915439 | 0.560945386 | 0.639687621 | 0.318708812 | 0.617851757 | 0.436751831 | 0.682786719 | 0.640401379 | 0.429998251 | 0.56151081 | 0.754693683 | 0.446299463 | 0.679760694 | 0.158995973 | 0.700440913 | 0.576569198 | 0.576234575 | 0.501067832 | 0.444913208 | 0.553777309 |
| TCGA_TCGA-55-1592 | 0.184434604 | 0.411244498 | 0.42436078 | 0.626949742 | 0.593886874 | 0.577263036 | 0.33261765 | 0.634909222 | 0.463852082 | 0.754853906 | 0.737241446 | 0.42094312 | 0.5382132 | 0.784632415 | 0.472539097 | 0.648016468 | 0.157942213 | 0.684104616 | 0.691213068 | 0.5769144 | 0.499869511 | 0.339974518 | 0.470420749 |
| TCGA_TCGA-50-8457 | 0.548089806 | 0.468279261 | 0.556568253 | 0.672399191 | 0.581008306 | 0.607048142 | 0.425219129 | 0.590342504 | 0.613850632 | 0.651599796 | 0.784643333 | 0.451481052 | 0.574624749 | 0.786424225 | 0.488894565 | 0.68012936 | 0.206401136 | 0.696804195 | 0.649648374 | 0.571984409 | 0.550320073 | 0.398888719 | 0.487258142 |
| TCGA_TCGA-78-7159 | 0.307250284 | 0.501476947 | 0.492618336 | 0.604963612 | 0.596105529 | 0.720073676 | 0.215825401 | 0.597349003 | 0.443869562 | 0.67734519 | 0.684706128 | 0.367946847 | 0.407966444 | 0.73745717 | 0.497772191 | 0.633982729 | 0.264435579 | 0.653549348 | 0.537536337 | 0.479049297 | 0.460658788 | 0.352869534 | 0.587845569 |
| TCGA_TCGA-67-6215 | 0.179090011 | 0.417541891 | 0.396169654 | 0.699113888 | 0.635895149 | 0.655057779 | 0.235378995 | 0.5974468 | 0.409204072 | 0.715538238 | 0.72529345 | 0.43877564 | 0.451926112 | 0.739686468 | 0.423194559 | 0.65011022 | 0.173820609 | 0.709969249 | 0.564345444 | 0.543526528 | 0.495098713 | 0.362758631 | 0.504143857 |
| TCGA_TCGA-MP-A4T9 | 0.2482779 | 0.449078698 | 0.473572746 | 0.672244071 | 0.587757687 | 0.665173728 | 0.409900846 | 0.658821223 | 0.438192772 | 0.715797331 | 0.746713586 | 0.41748616 | 0.587692639 | 0.771731892 | 0.477998901 | 0.681978656 | 0.221176794 | 0.72396519 | 0.644781184 | 0.59751411 | 0.549983929 | 0.389070266 | 0.482412678 |
| TCGA_TCGA-97-7938 | 0.251490222 | 0.435494547 | 0.418885316 | 0.651712143 | 0.607903035 | 0.632553444 | 0.450866075 | 0.560047313 | 0.436804852 | 0.667158258 | 0.639799975 | 0.389530371 | 0.59767866 | 0.702375183 | 0.415367703 | 0.672610866 | 0.229663533 | 0.657039973 | 0.555074198 | 0.554185818 | 0.5096602 | 0.416632604 | 0.573017469 |
| TCGA_TCGA-05-5425 | 0.335021009 | 0.647713905 | 0.63726272 | 0.702379251 | 0.618252726 | 0.670086824 | 0.395436469 | 0.672225107 | 0.53055005 | 0.702488321 | 0.868347892 | 0.509451101 | 0.580517175 | 0.769538317 | 0.492102559 | 0.720596711 | 0.29002177 | 0.683136009 | 0.712209412 | 0.617036449 | 0.573082444 | 0.383011086 | 0.562342894 |
| TCGA_TCGA-55-7913 | 0.188537827 | 0.514807585 | 0.454895552 | 0.569873181 | 0.625265124 | 0.707891107 | 0.202924112 | 0.572689788 | 0.335323529 | 0.67258328 | 0.583538483 | 0.327780391 | 0.387690805 | 0.731346326 | 0.370117792 | 0.543624524 | 0.347655108 | 0.667682039 | 0.497186999 | 0.472464473 | 0.45409508 | 0.352733509 | 0.578062715 |
| TCGA_TCGA-05-4417 | 0.397400039 | 0.586202327 | 0.577399377 | 0.648980792 | 0.590315765 | 0.709186154 | 0.394067694 | 0.670461436 | 0.536682025 | 0.722869841 | 0.83012577 | 0.446211598 | 0.548940072 | 0.773380204 | 0.513516727 | 0.676762315 | 0.267802118 | 0.709703674 | 0.710231018 | 0.573578362 | 0.546867504 | 0.382521335 | 0.55574667 |
| TCGA_TCGA-75-6203 | 0.296915979 | 0.399375297 | 0.557391999 | 0.705016423 | 0.614567308 | 0.604521932 | 0.446293201 | 0.697874597 | 0.526701108 | 0.727532063 | 0.902950604 | 0.502201061 | 0.650486651 | 0.810761302 | 0.576705083 | 0.737763086 | 0.191104699 | 0.772154765 | 0.703324157 | 0.633400832 | 0.57906817 | 0.404233679 | 0.534438433 |
| TCGA_TCGA-05-4434 | 0.386160403 | 0.6000615 | 0.566085951 | 0.698013403 | 0.63236912 | 0.680813993 | 0.259536965 | 0.668710732 | 0.515493609 | 0.704535372 | 0.900956104 | 0.513342433 | 0.531260673 | 0.775745688 | 0.492000819 | 0.653725872 | 0.236297214 | 0.697821618 | 0.816709453 | 0.620095752 | 0.587082139 | 0.399283593 | 0.4859961 |
| TCGA_TCGA-55-8096 | 0.35975139 | 0.392008328 | 0.428389649 | 0.663611365 | 0.647205132 | 0.660065764 | 0.261106767 | 0.676897206 | 0.519861813 | 0.710218501 | 0.852969385 | 0.450319697 | 0.553878753 | 0.776463362 | 0.521956313 | 0.671452186 | 0.091273475 | 0.717110776 | 0.771442712 | 0.577461786 | 0.534422685 | 0.361044549 | 0.476699094 |
| TCGA_TCGA-49-6767 | 0.187905559 | 0.563391203 | 0.604230923 | 0.645393033 | 0.593528127 | 0.66454749 | 0.202322365 | 0.654370713 | 0.461916171 | 0.684293579 | 0.808120762 | 0.376177084 | 0.349213835 | 0.759078105 | 0.493290662 | 0.630919534 | 0.188536903 | 0.684604406 | 0.609294629 | 0.522084296 | 0.512310533 | 0.480917763 | 0.439565582 |
| TCGA_TCGA-MP-A4T6 | 0.285861211 | 0.380990364 | 0.505179698 | 0.659910974 | 0.583005148 | 0.699149259 | 0.343821367 | 0.536810633 | 0.46507282 | 0.608635217 | 0.681074118 | 0.424373768 | 0.458523088 | 0.762324212 | 0.370338956 | 0.604239105 | 0.270167669 | 0.661714713 | 0.536461608 | 0.540689512 | 0.486816189 | 0.404104513 | 0.428688032 |
| TCGA_TCGA-MP-A4TE | 0.22593873 | 0.405177699 | 0.445125474 | 0.544697224 | 0.564164992 | 0.814076743 | 0.194401554 | 0.511932197 | 0.273768083 | 0.63602248 | 0.54750428 | 0.291367732 | 0.458946712 | 0.737264802 | 0.350376073 | 0.613714943 | 0.370583291 | 0.619918375 | 0.404129574 | 0.481037351 | 0.37949227 | 0.363998889 | 0.511154618 |
| GSE26939_GSM663284 | 0.295467632 | 0.626482592 | 0.487742128 | 0.655198556 | 0.570041015 | 0.753834329 | 0.251735413 | 0.598785657 | 0.481802541 | 0.624335173 | 0.716982336 | 0.421653285 | 0.436773087 | 0.723637578 | 0.407017246 | 0.67870628 | 0.210241903 | 0.607852729 | 0.554187618 | 0.510385424 | 0.476246648 | 0.325811601 | 0.546220093 |
| GSE26939_GSM663285 | 0.290579956 | 0.5366648 | 0.567405712 | 0.708743438 | 0.617033578 | 0.642732629 | 0.3101404 | 0.707503406 | 0.496426484 | 0.724296055 | 0.860027232 | 0.498721818 | 0.587253333 | 0.790852405 | 0.550505019 | 0.693872721 | 0.231316696 | 0.730432402 | 0.749131598 | 0.625957245 | 0.593168287 | 0.439481 | 0.497848531 |
| GSE26939_GSM663286 | 0.452087776 | 0.62311665 | 0.541487683 | 0.694196703 | 0.583924441 | 0.71758254 | 0.389102595 | 0.611157236 | 0.559515002 | 0.705595372 | 0.815621668 | 0.469546428 | 0.468021035 | 0.73580194 | 0.441580272 | 0.613486547 | 0.308191442 | 0.65666621 | 0.677435582 | 0.55647051 | 0.52435244 | 0.353347202 | 0.553191941 |
| GSE26939_GSM663287 | 0.206081717 | 0.403674336 | 0.496062774 | 0.650187256 | 0.567925623 | 0.612188813 | 0.366855625 | 0.584560133 | 0.437368851 | 0.715389792 | 0.806937722 | 0.40200779 | 0.527949844 | 0.768439106 | 0.466976506 | 0.708707434 | 0.243439344 | 0.69358092 | 0.634869579 | 0.608784356 | 0.522614278 | 0.422879302 | 0.47710502 |
| GSE26939_GSM663288 | 0.181482136 | 0.453054254 | 0.413883545 | 0.641797261 | 0.602444508 | 0.570863258 | 0.23463594 | 0.639504818 | 0.327789785 | 0.641533852 | 0.693250719 | 0.349839671 | 0.394419755 | 0.75634039 | 0.47358075 | 0.620354401 | 0.20960645 | 0.646837645 | 0.558580391 | 0.518771716 | 0.505027465 | 0.338035732 | 0.541068618 |
| GSE26939_GSM663289 | 0.155617637 | 0.47858133 | 0.438439716 | 0.659807361 | 0.601385372 | 0.688880938 | 0.234774289 | 0.67408354 | 0.394698758 | 0.668438045 | 0.787990433 | 0.370370872 | 0.485966829 | 0.763220968 | 0.505165346 | 0.668871389 | 0.195188869 | 0.682844305 | 0.590078007 | 0.529905119 | 0.524917417 | 0.342925563 | 0.42471054 |
| GSE26939_GSM663290 | 0.191104317 | 0.323545974 | 0.512988413 | 0.587345084 | 0.615359045 | 0.741689753 | 0.304163798 | 0.608170072 | 0.373003932 | 0.652123411 | 0.572848923 | 0.386223582 | 0.383245118 | 0.75390035 | 0.433835884 | 0.615576461 | 0.177574125 | 0.703635832 | 0.521314943 | 0.530397957 | 0.492754145 | 0.460534435 | 0.499383445 |
| GSE26939_GSM663291 | 0.228465453 | 0.457674672 | 0.551381849 | 0.671952846 | 0.598698267 | 0.665209424 | 0.429338851 | 0.630037585 | 0.445022991 | 0.690558988 | 0.761063927 | 0.413669665 | 0.594999507 | 0.769808309 | 0.479926535 | 0.677259393 | 0.15899602 | 0.686516094 | 0.607157338 | 0.592553443 | 0.558668118 | 0.415742901 | 0.493067641 |
| GSE26939_GSM663292 | 0.221310594 | 0.503313491 | 0.472450162 | 0.696156581 | 0.61933259 | 0.640897558 | 0.476711651 | 0.631646472 | 0.46201158 | 0.720384116 | 0.756534983 | 0.466084706 | 0.618496086 | 0.749883424 | 0.536167314 | 0.70733756 | 0.273271827 | 0.731045149 | 0.666334026 | 0.576119971 | 0.576868072 | 0.41323387 | 0.558730846 |
| GSE26939_GSM663293 | 0.123410881 | 0.280063913 | 0.377597631 | 0.657763593 | 0.616115256 | 0.730027091 | 0.187075407 | 0.629595149 | 0.421674524 | 0.750902725 | 0.716163641 | 0.416510849 | 0.472004668 | 0.789841768 | 0.540719288 | 0.650249895 | 0.140017243 | 0.712059954 | 0.629900454 | 0.544401565 | 0.562490538 | 0.408960699 | 0.450859811 |
| GSE26939_GSM663294 | 0.535642119 | 0.555128058 | 0.587635069 | 0.67650957 | 0.58725914 | 0.697322791 | 0.40313173 | 0.557898379 | 0.601678229 | 0.690294238 | 0.766864857 | 0.413207457 | 0.532793472 | 0.763517836 | 0.449762344 | 0.654817157 | 0.28805783 | 0.688691091 | 0.6035119 | 0.511920586 | 0.535501397 | 0.348931744 | 0.547496065 |
| GSE26939_GSM663295 | 0.304165447 | 0.4259911 | 0.515771536 | 0.714873 | 0.590857607 | 0.65494586 | 0.389185513 | 0.590160092 | 0.486674626 | 0.716324708 | 0.754797211 | 0.43459697 | 0.610785297 | 0.778293848 | 0.430026277 | 0.666736336 | 0.217435633 | 0.690479552 | 0.671910914 | 0.574476663 | 0.515923808 | 0.430244014 | 0.496478699 |
| GSE26939_GSM663296 | 0.201157463 | 0.353417248 | 0.462388753 | 0.635454649 | 0.618119166 | 0.614249841 | 0.330648091 | 0.598352044 | 0.363941096 | 0.662458029 | 0.557871224 | 0.39614229 | 0.459758743 | 0.725713316 | 0.387985642 | 0.657312916 | 0.163384966 | 0.646446475 | 0.586276431 | 0.54595109 | 0.482638958 | 0.41670218 | 0.497245102 |
| GSE26939_GSM663297 | 0.287057014 | 0.475726082 | 0.517254395 | 0.66189824 | 0.616749956 | 0.691056824 | 0.192379524 | 0.611348907 | 0.469109776 | 0.696649893 | 0.782564906 | 0.47446176 | 0.504469119 | 0.739943064 | 0.533251983 | 0.676870314 | 0.171477741 | 0.693482832 | 0.690784561 | 0.566620858 | 0.540615857 | 0.443603612 | 0.428646775 |
| GSE26939_GSM663298 | 0.269613492 | 0.40048468 | 0.507314145 | 0.670613911 | 0.623445778 | 0.657466235 | 0.268099737 | 0.582004074 | 0.451266595 | 0.716211108 | 0.738367594 | 0.462663084 | 0.520744229 | 0.754340848 | 0.473557305 | 0.678566637 | 0.173190043 | 0.673666883 | 0.632077909 | 0.57367383 | 0.519532036 | 0.418575853 | 0.46875335 |
| GSE26939_GSM663299 | 0.203363106 | 0.342910572 | 0.411824331 | 0.620115536 | 0.587434954 | 0.60193326 | 0.438213067 | 0.593543933 | 0.392317225 | 0.721522906 | 0.681495822 | 0.385081258 | 0.589063196 | 0.763355649 | 0.436556656 | 0.664837921 | 0.173684278 | 0.703394376 | 0.564862802 | 0.559691051 | 0.501221773 | 0.385967388 | 0.485720249 |
| GSE26939_GSM663300 | 0.163909343 | 0.464540559 | 0.502834708 | 0.683903225 | 0.599592172 | 0.652900122 | 0.3474451 | 0.64543857 | 0.405615255 | 0.699140042 | 0.792051841 | 0.477660885 | 0.540397772 | 0.757777769 | 0.471685081 | 0.700553665 | 0.245725501 | 0.711646309 | 0.654632085 | 0.598391444 | 0.527399427 | 0.373122861 | 0.603341081 |
| GSE26939_GSM663301 | 0.449032928 | 0.655555286 | 0.660998057 | 0.696106763 | 0.572778152 | 0.66146449 | 0.326199068 | 0.662555294 | 0.568134501 | 0.7018111 | 0.884961774 | 0.524611948 | 0.434697144 | 0.749226004 | 0.496836451 | 0.67759692 | 0.18120506 | 0.706269701 | 0.685963884 | 0.548084061 | 0.562648448 | 0.396269433 | 0.524590264 |
| GSE26939_GSM663302 | 0.171497692 | 0.440795795 | 0.487166401 | 0.673758345 | 0.579990431 | 0.685065015 | 0.407809215 | 0.624393295 | 0.438171257 | 0.682571805 | 0.665401006 | 0.390019417 | 0.496870704 | 0.710437738 | 0.407007255 | 0.671751101 | 0.224807923 | 0.64429115 | 0.610882237 | 0.549600974 | 0.497852479 | 0.402285184 | 0.504291002 |
| GSE26939_GSM663303 | 0.198236853 | 0.57803289 | 0.541715413 | 0.680698416 | 0.595884978 | 0.715421315 | 0.443993678 | 0.619614551 | 0.433726332 | 0.671911337 | 0.72693717 | 0.372351806 | 0.508273836 | 0.752946979 | 0.479125366 | 0.673116671 | 0.295320298 | 0.666038652 | 0.68071344 | 0.55643347 | 0.546147358 | 0.411947517 | 0.560008946 |
| GSE26939_GSM663304 | 0.115066938 | 0.462181525 | 0.437509016 | 0.592275991 | 0.633955266 | 0.697831631 | 0.219434998 | 0.623260229 | 0.325468084 | 0.663993606 | 0.706909481 | 0.403923563 | 0.478849124 | 0.756389968 | 0.433548743 | 0.587131378 | 0.368886357 | 0.651857645 | 0.522681119 | 0.467674223 | 0.438635575 | 0.405362242 | 0.573062772 |
| GSE26939_GSM663305 | 0.142847865 | 0.512122278 | 0.456296703 | 0.598125578 | 0.554555533 | 0.723673316 | 0.28954477 | 0.602896971 | 0.324350606 | 0.626383567 | 0.544583349 | 0.305744793 | 0.402639254 | 0.727915394 | 0.381001416 | 0.596648664 | 0.182898422 | 0.643623009 | 0.514174547 | 0.500013028 | 0.412608169 | 0.366442614 | 0.546075005 |
| GSE26939_GSM663306 | 0.152567278 | 0.493523673 | 0.470592781 | 0.644771519 | 0.586876553 | 0.714160244 | 0.196860117 | 0.603193897 | 0.383768046 | 0.623864767 | 0.70208699 | 0.333617868 | 0.539836399 | 0.686299547 | 0.400629411 | 0.599328258 | 0.191209392 | 0.601030987 | 0.457558583 | 0.508889815 | 0.450051924 | 0.354046974 | 0.507825231 |
| GSE26939_GSM663307 | 0.198698518 | 0.448085008 | 0.528438956 | 0.72060183 | 0.594016313 | 0.649177112 | 0.403697289 | 0.653346446 | 0.500231973 | 0.759272704 | 0.833785501 | 0.458431203 | 0.613391546 | 0.767558973 | 0.528193099 | 0.72348174 | 0.21832542 | 0.726631918 | 0.703910482 | 0.599046737 | 0.568820234 | 0.410401376 | 0.53686171 |
| GSE26939_GSM663308 | 0.397316829 | 0.671467882 | 0.686785541 | 0.712324431 | 0.597205718 | 0.63042332 | 0.36990289 | 0.67277515 | 0.58866277 | 0.661540194 | 0.88557698 | 0.52782305 | 0.504300706 | 0.766453514 | 0.581250971 | 0.765495431 | 0.236647993 | 0.722861815 | 0.786239251 | 0.584096236 | 0.595185398 | 0.438947448 | 0.595780235 |
| GSE26939_GSM663309 | 0.274859046 | 0.557995023 | 0.530819889 | 0.645475664 | 0.539688343 | 0.739840369 | 0.26045493 | 0.582360365 | 0.483527586 | 0.617461953 | 0.645658342 | 0.360189268 | 0.360290396 | 0.75778253 | 0.438611039 | 0.631042167 | 0.254905666 | 0.658044 | 0.473770563 | 0.431288953 | 0.474658784 | 0.473160352 | 0.500040877 |
| GSE26939_GSM663310 | 0.404225081 | 0.588897663 | 0.592212752 | 0.653154333 | 0.645764728 | 0.650433298 | 0.339615212 | 0.625623124 | 0.559213846 | 0.695450976 | 0.712564848 | 0.401959438 | 0.448680263 | 0.72210827 | 0.411129392 | 0.667659426 | 0.16528943 | 0.708912363 | 0.535296669 | 0.524183255 | 0.504877845 | 0.379174573 | 0.489325103 |
| GSE26939_GSM663311 | 0.500547536 | 0.679939017 | 0.595309136 | 0.667192556 | 0.578540011 | 0.646923159 | 0.435457242 | 0.614129916 | 0.535731685 | 0.655380619 | 0.692526203 | 0.416988573 | 0.574170466 | 0.705937006 | 0.463927734 | 0.630064514 | 0.200465084 | 0.677577777 | 0.587134451 | 0.526164621 | 0.52335071 | 0.388943752 | 0.544460755 |
| GSE26939_GSM663312 | 0.511020604 | 0.552569131 | 0.645953201 | 0.687885346 | 0.548058128 | 0.694959734 | 0.248712565 | 0.642474353 | 0.591577818 | 0.677767449 | 0.771643855 | 0.462125452 | 0.490705581 | 0.789465582 | 0.511577603 | 0.735468259 | 0.203624261 | 0.719794201 | 0.644675291 | 0.587957533 | 0.564262533 | 0.440541253 | 0.451428519 |
| GSE26939_GSM663313 | 0.20889911 | 0.440788573 | 0.505701276 | 0.672865569 | 0.592485763 | 0.676306827 | 0.293357197 | 0.56386023 | 0.436473403 | 0.75969299 | 0.748796795 | 0.446260395 | 0.485824694 | 0.752667617 | 0.401631316 | 0.655791244 | 0.188284954 | 0.675676139 | 0.584139673 | 0.494764631 | 0.462045727 | 0.38677588 | 0.484670287 |
| GSE26939_GSM663314 | 0.169063886 | 0.565035567 | 0.456003878 | 0.591136782 | 0.587725663 | 0.695233228 | 0.37008928 | 0.596982687 | 0.362466276 | 0.679537812 | 0.586955076 | 0.288186605 | 0.457758747 | 0.694390199 | 0.37625437 | 0.648854588 | 0.193112328 | 0.614744466 | 0.534880818 | 0.469332965 | 0.47519891 | 0.340017523 | 0.564555174 |
| GSE26939_GSM663315 | 0.201524922 | 0.509046313 | 0.452640183 | 0.640269334 | 0.571074568 | 0.667646681 | 0.266396346 | 0.618997804 | 0.433510285 | 0.66252992 | 0.633266394 | 0.384782204 | 0.556458174 | 0.73069074 | 0.463536037 | 0.637197859 | 0.215835397 | 0.666399584 | 0.573099426 | 0.523884199 | 0.511009256 | 0.394132308 | 0.542149992 |
| GSE26939_GSM663316 | 0.245017994 | 0.467289094 | 0.508539763 | 0.654809831 | 0.591717228 | 0.635950082 | 0.471593078 | 0.613668795 | 0.436590881 | 0.729839973 | 0.6961933 | 0.395387194 | 0.562364901 | 0.745015466 | 0.4456218 | 0.657322624 | 0.217811987 | 0.712311933 | 0.637015605 | 0.584693409 | 0.537367433 | 0.425855098 | 0.590285181 |
| GSE26939_GSM663317 | 0.506893161 | 0.600920938 | 0.608117794 | 0.686307795 | 0.560918476 | 0.633697301 | 0.373055958 | 0.645031976 | 0.604382611 | 0.672536254 | 0.784912647 | 0.413742489 | 0.538844198 | 0.725126613 | 0.487566539 | 0.631173081 | 0.104601099 | 0.71433974 | 0.704894023 | 0.538432051 | 0.547904749 | 0.397035677 | 0.482917062 |
| GSE26939_GSM663318 | 0.205570859 | 0.466589493 | 0.402184924 | 0.490830177 | 0.626369477 | 0.584198074 | 0.255020402 | 0.576108616 | 0.28297912 | 0.641004184 | 0.445533487 | 0.315517453 | 0.312565035 | 0.628352791 | 0.378474072 | 0.58198311 | 0.04312881 | 0.607504756 | 0.4144797 | 0.393682591 | 0.421203081 | 0.422427306 | 0.460228637 |
| GSE26939_GSM663319 | 0.301776733 | 0.618319771 | 0.557900984 | 0.660229375 | 0.667080346 | 0.702258193 | 0.241906231 | 0.619411355 | 0.489339633 | 0.695255506 | 0.718106616 | 0.416576938 | 0.406299998 | 0.699068822 | 0.439931629 | 0.676381758 | 0.170671883 | 0.667615631 | 0.628691727 | 0.554053478 | 0.525779213 | 0.413914092 | 0.497835837 |
| GSE26939_GSM663320 | 0.294012788 | 0.532924626 | 0.571440231 | 0.670923666 | 0.585678881 | 0.735822892 | 0.224333607 | 0.602710917 | 0.46086586 | 0.604130659 | 0.619577591 | 0.377809028 | 0.402221179 | 0.757942099 | 0.403112311 | 0.660428898 | 0.350805986 | 0.655555817 | 0.607898478 | 0.489807752 | 0.440908153 | 0.415515948 | 0.503293674 |
| GSE26939_GSM663321 | 0.667929614 | 0.679189727 | 0.703113088 | 0.726125268 | 0.625220401 | 0.637138778 | 0.316079652 | 0.661871705 | 0.812107485 | 0.65149573 | 0.837281728 | 0.500157974 | 0.473391716 | 0.770515106 | 0.5440469 | 0.724782222 | 0.303454545 | 0.711811089 | 0.806902796 | 0.581807251 | 0.629729508 | 0.356275605 | 0.492000555 |
| GSE26939_GSM663322 | 0.346960269 | 0.570052762 | 0.607104655 | 0.693531234 | 0.572031066 | 0.694921837 | 0.363296849 | 0.642151674 | 0.510925604 | 0.690913651 | 0.786098981 | 0.449015409 | 0.557871188 | 0.77141137 | 0.449939218 | 0.660326285 | 0.21403579 | 0.691669237 | 0.66793067 | 0.652836276 | 0.531932532 | 0.395400627 | 0.515577885 |
| GSE26939_GSM663323 | 0.367826566 | 0.525442898 | 0.665242698 | 0.688103906 | 0.629490288 | 0.692761027 | 0.388164404 | 0.645372819 | 0.518918879 | 0.698182683 | 0.833984383 | 0.423894498 | 0.539538184 | 0.786514627 | 0.47367595 | 0.687315393 | 0.214606257 | 0.711880575 | 0.685156657 | 0.597480568 | 0.561352007 | 0.456984061 | 0.470255051 |
| GSE26939_GSM663324 | 0.323944921 | 0.483224898 | 0.585474593 | 0.672817972 | 0.57455326 | 0.688619856 | 0.31345567 | 0.618692724 | 0.473362767 | 0.649342938 | 0.737216057 | 0.417983299 | 0.490223124 | 0.741376902 | 0.449623249 | 0.603058679 | 0.154956467 | 0.657779258 | 0.572588873 | 0.529426427 | 0.481599587 | 0.341564004 | 0.485369745 |
| GSE26939_GSM663325 | 0.689438304 | 0.588594741 | 0.666068718 | 0.678535887 | 0.587778537 | 0.655816096 | 0.439092951 | 0.598376797 | 0.676937402 | 0.675918275 | 0.84863864 | 0.457258413 | 0.603631891 | 0.799991709 | 0.472990397 | 0.676493032 | 0.187603556 | 0.729318695 | 0.653895567 | 0.583443507 | 0.564613371 | 0.366906556 | 0.532600041 |
| GSE26939_GSM663326 | 0.377827154 | 0.493215066 | 0.492521784 | 0.687773395 | 0.588022205 | 0.646751306 | 0.484499965 | 0.616566046 | 0.534704928 | 0.768837026 | 0.809075782 | 0.467446299 | 0.632460788 | 0.768711314 | 0.490420851 | 0.697656209 | 0.239699341 | 0.72185812 | 0.651774565 | 0.596860166 | 0.558925957 | 0.381058014 | 0.521531171 |
| GSE26939_GSM663327 | 0.197439729 | 0.494118365 | 0.484007315 | 0.623620544 | 0.549097901 | 0.689881573 | 0.21215802 | 0.554245075 | 0.412915387 | 0.662927627 | 0.657217035 | 0.356316753 | 0.28062002 | 0.728653949 | 0.403956421 | 0.570160683 | 0.264480626 | 0.620405497 | 0.53121256 | 0.475279806 | 0.481925696 | 0.404160406 | 0.430099466 |
| GSE26939_GSM663328 | 0.460649339 | 0.598666276 | 0.558096862 | 0.674030978 | 0.638030918 | 0.702433366 | 0.330243607 | 0.614534958 | 0.612936281 | 0.700220889 | 0.793591282 | 0.445245781 | 0.5106318 | 0.719412871 | 0.473961389 | 0.658451555 | 0.193226593 | 0.69655698 | 0.624688675 | 0.549846288 | 0.572845748 | 0.470514079 | 0.46446915 |
| GSE26939_GSM663329 | 0.198099228 | 0.246766255 | 0.382729663 | 0.512301542 | 0.554360131 | 0.605962409 | 0.268188486 | 0.509227641 | 0.32776847 | 0.642223952 | 0.454312278 | 0.35073609 | 0.435728481 | 0.739981033 | 0.355553495 | 0.548467667 | 0.134398995 | 0.647223991 | 0.402288612 | 0.521768484 | 0.395284274 | 0.414656161 | 0.528041727 |
| GSE26939_GSM663330 | 0.244082807 | 0.459099258 | 0.4352972 | 0.633258166 | 0.560055505 | 0.654914319 | 0.272837678 | 0.568798004 | 0.421145004 | 0.658502161 | 0.610066806 | 0.356451881 | 0.382289387 | 0.75359008 | 0.437603823 | 0.605271622 | 0.274949656 | 0.647042673 | 0.505330415 | 0.5133368 | 0.474942179 | 0.438694703 | 0.444430436 |
| GSE26939_GSM663331 | 0.391990414 | 0.469875068 | 0.450842877 | 0.642248814 | 0.655554734 | 0.627318326 | 0.32647312 | 0.570954432 | 0.502233004 | 0.711229324 | 0.728545888 | 0.412052401 | 0.405176848 | 0.729418457 | 0.455930507 | 0.677415575 | 0.098162912 | 0.731205522 | 0.592949805 | 0.524186189 | 0.499201978 | 0.45402897 | 0.484888551 |
| GSE26939_GSM663332 | 0.249792385 | 0.45648315 | 0.428654066 | 0.682473942 | 0.637153064 | 0.662382176 | 0.370452109 | 0.585694714 | 0.448939974 | 0.710926897 | 0.672148501 | 0.39808152 | 0.459314595 | 0.751360265 | 0.401787117 | 0.649130385 | 0.270628672 | 0.645117465 | 0.596118184 | 0.541388725 | 0.500526585 | 0.414505507 | 0.516214762 |
| GSE26939_GSM663333 | 0.513347791 | 0.574125643 | 0.67810439 | 0.709678578 | 0.621366852 | 0.736134025 | 0.325007151 | 0.660519478 | 0.635096853 | 0.722650177 | 0.962854323 | 0.497973161 | 0.563794384 | 0.762831647 | 0.566357663 | 0.717699983 | 0.181076614 | 0.722109774 | 0.751870728 | 0.654678449 | 0.616036155 | 0.421207919 | 0.48281883 |
| GSE26939_GSM663334 | 0.099441326 | 0.480162266 | 0.471012819 | 0.575917477 | 0.575353949 | 0.696746555 | 0.454798063 | 0.613610872 | 0.341071795 | 0.686868811 | 0.669602208 | 0.38205397 | 0.544717661 | 0.744679725 | 0.463301758 | 0.632416222 | 0.19801055 | 0.65954262 | 0.491830729 | 0.535631741 | 0.470860205 | 0.357113594 | 0.567766472 |
| GSE26939_GSM663335 | 0.21542579 | 0.490213398 | 0.426663221 | 0.508621705 | 0.621774228 | 0.591936501 | 0.156798293 | 0.596297747 | 0.257393268 | 0.641618301 | 0.460917354 | 0.259876901 | 0.327255808 | 0.704782669 | 0.341037787 | 0.494367812 | 0.0816656 | 0.531049766 | 0.371795958 | 0.39889145 | 0.339113278 | 0.290327871 | 0.524271799 |
| GSE26939_GSM663336 | 0.40634086 | 0.554103116 | 0.581175585 | 0.735471358 | 0.573218709 | 0.714579599 | 0.266942468 | 0.634240416 | 0.585532381 | 0.714320931 | 0.906094596 | 0.486576876 | 0.540661405 | 0.750437744 | 0.502700236 | 0.709285882 | 0.205081393 | 0.691148307 | 0.774171594 | 0.56334786 | 0.59786527 | 0.417779442 | 0.505084103 |
| GSE26939_GSM663337 | 0.271218034 | 0.565520348 | 0.502283414 | 0.705296952 | 0.591796992 | 0.617126865 | 0.3237311 | 0.653647097 | 0.486022013 | 0.72585538 | 0.764067122 | 0.392766925 | 0.538224584 | 0.749922984 | 0.448677948 | 0.706905608 | 0.302846889 | 0.647250499 | 0.750319742 | 0.575214034 | 0.536126722 | 0.410636117 | 0.576182757 |
| GSE26939_GSM663338 | 0.432096334 | 0.56453607 | 0.636457708 | 0.709945098 | 0.594983476 | 0.747672625 | 0.302115888 | 0.600879122 | 0.580165502 | 0.687602666 | 0.804923833 | 0.418079171 | 0.484139196 | 0.749274066 | 0.447977314 | 0.655153174 | 0.295692183 | 0.692535293 | 0.683921647 | 0.5539022 | 0.522048456 | 0.359151069 | 0.57047433 |
| GSE26939_GSM663339 | 0.234773403 | 0.599960122 | 0.532394984 | 0.662958945 | 0.640755323 | 0.697641023 | 0.360014427 | 0.631242334 | 0.4991063 | 0.607930847 | 0.75726315 | 0.441495097 | 0.407928147 | 0.705961621 | 0.461951591 | 0.646846127 | 0.054482614 | 0.655590658 | 0.706797026 | 0.513861655 | 0.459462215 | 0.381255267 | 0.457232564 |
| GSE26939_GSM663340 | 0.17142347 | 0.471692824 | 0.413011215 | 0.670826845 | 0.557050265 | 0.726894086 | 0.35506089 | 0.620725904 | 0.394704377 | 0.70089421 | 0.808175713 | 0.449607244 | 0.492306594 | 0.772554519 | 0.511057133 | 0.651184626 | 0.274306797 | 0.691138779 | 0.642572872 | 0.572650296 | 0.506428238 | 0.377413162 | 0.455703364 |
| GSE26939_GSM663341 | 0.169054533 | 0.435755963 | 0.340992175 | 0.541552464 | 0.519887583 | 0.677801134 | 0.313457559 | 0.527436768 | 0.290533111 | 0.671342177 | 0.678140033 | 0.273802904 | 0.433434258 | 0.802745464 | 0.412446074 | 0.550386238 | 0.111480734 | 0.623072559 | 0.322429341 | 0.503328346 | 0.420726148 | 0.379636393 | 0.53229287 |
| GSE26939_GSM663342 | 0.402360764 | 0.495190648 | 0.557836179 | 0.677551048 | 0.611526712 | 0.637614771 | 0.363122189 | 0.661956856 | 0.539220555 | 0.717041549 | 0.846770861 | 0.471925336 | 0.559047505 | 0.78713693 | 0.478759058 | 0.685916193 | 0.180307091 | 0.735947809 | 0.724459849 | 0.61895727 | 0.54011478 | 0.387262045 | 0.529618455 |
| GSE26939_GSM663343 | 0.178980671 | 0.309901513 | 0.364260976 | 0.483525056 | 0.691745768 | 0.727650261 | 0.173407776 | 0.530579418 | 0.190548748 | 0.600287482 | 0.557686438 | 0.290622002 | 0.343139051 | 0.770919983 | 0.378075547 | 0.539320882 | 0.072596944 | 0.624600948 | 0.326243641 | 0.423775795 | 0.409570099 | 0.393067725 | 0.466353196 |
| GSE26939_GSM663344 | 0.167403234 | 0.483696366 | 0.388770937 | 0.52350316 | 0.588117882 | 0.686306207 | 0.304935641 | 0.545763276 | 0.296530236 | 0.623209503 | 0.541764722 | 0.329353775 | 0.424889731 | 0.78047876 | 0.469320105 | 0.579700307 | 0.08773161 | 0.609269173 | 0.42396372 | 0.486906123 | 0.467863885 | 0.416461204 | 0.478146829 |
| GSE26939_GSM663345 | 0.155895381 | 0.488604893 | 0.541238157 | 0.635197078 | 0.630031764 | 0.671848767 | 0.216675629 | 0.60003097 | 0.409417997 | 0.619709853 | 0.630235064 | 0.399892055 | 0.364262616 | 0.73491761 | 0.414930882 | 0.590138456 | 0.299395156 | 0.629802679 | 0.499742734 | 0.487795213 | 0.442920232 | 0.363123265 | 0.541329296 |
| GSE26939_GSM663346 | 0.369567321 | 0.538500414 | 0.509238827 | 0.70615648 | 0.582293087 | 0.64394158 | 0.292072902 | 0.628913223 | 0.462657626 | 0.738876227 | 0.87669902 | 0.446720079 | 0.527545687 | 0.814250422 | 0.540063557 | 0.675297237 | 0.22406593 | 0.703702663 | 0.663833667 | 0.584776601 | 0.572703707 | 0.385135732 | 0.527942675 |
| GSE26939_GSM663347 | 0.536572401 | 0.543584788 | 0.559243762 | 0.703703735 | 0.651119214 | 0.668544119 | 0.312510551 | 0.610844599 | 0.612636465 | 0.715368981 | 0.833177965 | 0.428427719 | 0.554824265 | 0.778562846 | 0.509211002 | 0.678713633 | 0.212967368 | 0.746069038 | 0.693199775 | 0.563984487 | 0.565758067 | 0.387105989 | 0.523898264 |
| GSE26939_GSM663348 | 0.494857724 | 0.589104785 | 0.501923071 | 0.669849944 | 0.5989254 | 0.65013298 | 0.316877781 | 0.626332532 | 0.549687967 | 0.656501097 | 0.760648824 | 0.457751417 | 0.512974133 | 0.780685604 | 0.495694345 | 0.684305823 | 0.2345924 | 0.689342065 | 0.702701963 | 0.548671723 | 0.519862365 | 0.393511884 | 0.511218894 |
| GSE26939_GSM663349 | 0.151671862 | 0.469369968 | 0.39301217 | 0.654247522 | 0.609035333 | 0.636073774 | 0.287978288 | 0.600665667 | 0.426929802 | 0.698561155 | 0.647240641 | 0.387247961 | 0.541130301 | 0.751857275 | 0.445395043 | 0.638367782 | 0.174562304 | 0.655467342 | 0.623064198 | 0.552968923 | 0.498777926 | 0.395846497 | 0.610889119 |
| GSE26939_GSM663350 | 0.216272856 | 0.553865866 | 0.583155698 | 0.679316941 | 0.569775047 | 0.693453323 | 0.228408014 | 0.656916897 | 0.448770925 | 0.684827444 | 0.802818167 | 0.504791905 | 0.423771824 | 0.743287022 | 0.534513547 | 0.668921352 | 0.224397094 | 0.691835318 | 0.67062605 | 0.610332958 | 0.490036061 | 0.475553336 | 0.472475429 |
| GSE26939_GSM663351 | 0.353330687 | 0.534239332 | 0.449074472 | 0.735473546 | 0.619155652 | 0.687685009 | 0.341256871 | 0.645000669 | 0.496477602 | 0.736631143 | 0.851998931 | 0.451780744 | 0.583662629 | 0.790050607 | 0.515624558 | 0.676294816 | 0.244386187 | 0.739590427 | 0.699473257 | 0.577461006 | 0.558759757 | 0.375112732 | 0.527097976 |
| GSE26939_GSM663352 | 0.45190324 | 0.606772283 | 0.569192391 | 0.6857627 | 0.588958993 | 0.551242785 | 0.54666257 | 0.623904925 | 0.542121954 | 0.737159017 | 0.756619325 | 0.400348918 | 0.618172031 | 0.792168321 | 0.529801482 | 0.700351937 | 0.228198097 | 0.723583539 | 0.676694789 | 0.581067095 | 0.583316061 | 0.407679525 | 0.578978969 |
| GSE26939_GSM663353 | 0.31388572 | 0.501376695 | 0.462714151 | 0.667729692 | 0.622717656 | 0.632373917 | 0.341067427 | 0.64373528 | 0.523772043 | 0.772578173 | 0.785042585 | 0.476765237 | 0.572283435 | 0.786235272 | 0.525167602 | 0.711837587 | 0.175376819 | 0.739327751 | 0.746212133 | 0.567995326 | 0.554767898 | 0.422090269 | 0.482237996 |
| GSE26939_GSM663354 | 0.238532092 | 0.600346004 | 0.531862846 | 0.612412025 | 0.562330013 | 0.761373413 | 0.283346321 | 0.612636775 | 0.397896244 | 0.669451963 | 0.645811284 | 0.378802458 | 0.365650791 | 0.711504724 | 0.42198995 | 0.614085974 | 0.116626146 | 0.637115767 | 0.557293703 | 0.463896999 | 0.404190011 | 0.406676426 | 0.540108206 |
| GSE26939_GSM663355 | 0.221517119 | 0.442981544 | 0.472684679 | 0.705288242 | 0.657487826 | 0.738323288 | 0.400800464 | 0.617500377 | 0.476838666 | 0.706575445 | 0.803799585 | 0.43985463 | 0.491909648 | 0.766091531 | 0.479399393 | 0.656367397 | 0.297814208 | 0.688479336 | 0.673809224 | 0.56476542 | 0.533698835 | 0.40283233 | 0.555594019 |
| GSE26939_GSM663356 | 0.258471848 | 0.51789447 | 0.420531407 | 0.615585703 | 0.546334116 | 0.637919376 | 0.420767194 | 0.578837044 | 0.41520867 | 0.693316761 | 0.767893187 | 0.349918381 | 0.505198365 | 0.820236207 | 0.494244586 | 0.682694719 | 0.179210986 | 0.731277166 | 0.56409299 | 0.576610644 | 0.529534456 | 0.387716365 | 0.543229191 |
| GSE26939_GSM663357 | 0.525859014 | 0.609542269 | 0.602861995 | 0.7225733 | 0.643872024 | 0.619062089 | 0.337570661 | 0.624834061 | 0.597911608 | 0.667776879 | 0.798727837 | 0.37321012 | 0.470175397 | 0.774397588 | 0.47949211 | 0.688378563 | 0.126333459 | 0.675732822 | 0.668182811 | 0.587713807 | 0.562344064 | 0.406851848 | 0.499997966 |
| GSE26939_GSM663358 | 0.275907155 | 0.44661634 | 0.417251699 | 0.706580437 | 0.615044633 | 0.624592191 | 0.490525577 | 0.676904678 | 0.503939789 | 0.754860801 | 0.852164769 | 0.493218799 | 0.573909648 | 0.776329029 | 0.574671096 | 0.715202126 | 0.284023981 | 0.779692057 | 0.699840553 | 0.615871817 | 0.576188423 | 0.369302311 | 0.531110833 |
| GSE26939_GSM663359 | 0.473280567 | 0.414224286 | 0.49971301 | 0.652435616 | 0.592917362 | 0.651988745 | 0.364804242 | 0.589128439 | 0.516995235 | 0.673179058 | 0.724412287 | 0.402499317 | 0.552543639 | 0.78077609 | 0.436369017 | 0.648494631 | 0.215754834 | 0.704707777 | 0.616444327 | 0.552588178 | 0.535735352 | 0.38072359 | 0.543994654 |
| GSE26939_GSM663360 | 0.187109336 | 0.373521203 | 0.471045511 | 0.65521922 | 0.64907838 | 0.701717955 | 0.337220232 | 0.628564746 | 0.448519341 | 0.736164167 | 0.784225697 | 0.478307211 | 0.474979749 | 0.766951986 | 0.479354214 | 0.667657599 | 0.151875639 | 0.713866852 | 0.690688593 | 0.594474096 | 0.518114539 | 0.419722908 | 0.481986327 |
| GSE26939_GSM663361 | 0.430582697 | 0.589999343 | 0.653461521 | 0.715354888 | 0.621318026 | 0.689827309 | 0.407352478 | 0.651123064 | 0.600305365 | 0.694754181 | 0.86151053 | 0.445848097 | 0.564221099 | 0.81036732 | 0.510971722 | 0.689051963 | 0.193868366 | 0.699236469 | 0.793900593 | 0.617391074 | 0.585016098 | 0.421187046 | 0.549527235 |
| GSE26939_GSM663362 | 0.253749711 | 0.578439331 | 0.546635282 | 0.660796959 | 0.620157248 | 0.722518913 | 0.341202744 | 0.619124337 | 0.493143525 | 0.655307074 | 0.7459531 | 0.425409416 | 0.423580568 | 0.736113073 | 0.445114132 | 0.684196176 | 0.223141327 | 0.625371336 | 0.695389464 | 0.536417992 | 0.527300742 | 0.374921232 | 0.515546516 |
| GSE26939_GSM663363 | 0.350548283 | 0.613848227 | 0.661860562 | 0.680477658 | 0.631847032 | 0.716779927 | 0.295065679 | 0.660405394 | 0.519612633 | 0.70848566 | 0.84607146 | 0.420737693 | 0.523114054 | 0.763469284 | 0.482192823 | 0.653405806 | 0.234097248 | 0.670701183 | 0.76426878 | 0.605761699 | 0.57747479 | 0.404684124 | 0.495956578 |
| GSE26939_GSM663364 | 0.274961382 | 0.442041757 | 0.432561135 | 0.593847143 | 0.58847623 | 0.688488897 | 0.240966788 | 0.584474452 | 0.418770551 | 0.656748792 | 0.546420818 | 0.348361347 | 0.376466202 | 0.692915123 | 0.442662276 | 0.652607418 | 0.17438955 | 0.618904864 | 0.524732622 | 0.482340542 | 0.471242796 | 0.347763737 | 0.436954419 |
| GSE26939_GSM663365 | 0.481061057 | 0.693989345 | 0.690237999 | 0.706335318 | 0.63340008 | 0.718681585 | 0.331843682 | 0.623005022 | 0.651366389 | 0.693819963 | 0.870320027 | 0.47999209 | 0.537892658 | 0.820592277 | 0.545737079 | 0.718243073 | 0.21929933 | 0.736084439 | 0.756699648 | 0.614381952 | 0.584141676 | 0.383474329 | 0.535864748 |
| GSE26939_GSM663366 | 0.187916046 | 0.368014049 | 0.490344077 | 0.658678541 | 0.607070263 | 0.586972398 | 0.386444456 | 0.62088249 | 0.458446857 | 0.716252804 | 0.720482316 | 0.434535406 | 0.64317633 | 0.763313563 | 0.483165277 | 0.685602126 | 0.162995804 | 0.711159566 | 0.64458768 | 0.587015125 | 0.544635694 | 0.389625673 | 0.556692799 |
| GSE26939_GSM663367 | 0.154076226 | 0.430785937 | 0.43830119 | 0.599297034 | 0.618733625 | 0.719323287 | 0.199616651 | 0.595441209 | 0.348062751 | 0.6051381 | 0.525753256 | 0.364707966 | 0.280904355 | 0.788772371 | 0.473040268 | 0.577842233 | 0.289369112 | 0.682228331 | 0.437171783 | 0.442920755 | 0.419285137 | 0.424871221 | 0.422300493 |
| GSE26939_GSM663368 | 0.283753982 | 0.591024151 | 0.519092757 | 0.621111532 | 0.605432659 | 0.69490946 | 0.37906497 | 0.590859659 | 0.537607078 | 0.655586639 | 0.698831963 | 0.451831457 | 0.388575822 | 0.738347278 | 0.428551449 | 0.626008428 | 0.153000683 | 0.641242182 | 0.5937142 | 0.530918801 | 0.486082201 | 0.332679001 | 0.565729892 |
| GSE26939_GSM663369 | 0.229313333 | 0.578192722 | 0.470708143 | 0.626318924 | 0.620540388 | 0.689418696 | 0.258217457 | 0.64509406 | 0.403375388 | 0.681714966 | 0.692094799 | 0.36213699 | 0.450450486 | 0.722245916 | 0.478518072 | 0.622990344 | 0.251883569 | 0.632223273 | 0.699180666 | 0.515578001 | 0.508143508 | 0.39623511 | 0.542268554 |
| GSE26939_GSM663370 | 0.307714592 | 0.460248805 | 0.492535241 | 0.656330086 | 0.601055519 | 0.585924849 | 0.418664146 | 0.59165158 | 0.514419125 | 0.734260908 | 0.670717887 | 0.417537613 | 0.577745041 | 0.748561258 | 0.415738522 | 0.677381054 | 0.189191951 | 0.680557675 | 0.674099393 | 0.566806617 | 0.530694573 | 0.39129289 | 0.517466596 |
| GSE26939_GSM663371 | 0.17519185 | 0.386524753 | 0.486108873 | 0.683340885 | 0.657128324 | 0.592954095 | 0.406573052 | 0.671767665 | 0.475833549 | 0.751753981 | 0.782113098 | 0.463168408 | 0.60831333 | 0.75935448 | 0.478596307 | 0.698960208 | 0.191454218 | 0.745514597 | 0.67531038 | 0.618926337 | 0.552465836 | 0.408723201 | 0.536803882 |
| GSE26939_GSM663372 | 0.241802547 | 0.584319703 | 0.536499711 | 0.718377849 | 0.631734763 | 0.689718158 | 0.290232741 | 0.668328962 | 0.482282485 | 0.738059627 | 0.890379806 | 0.442375366 | 0.5484207 | 0.771224841 | 0.509585706 | 0.708399642 | 0.152889222 | 0.730374012 | 0.783498495 | 0.568842235 | 0.564853977 | 0.508674331 | 0.521693342 |
| GSE26939_GSM663373 | 0.372559625 | 0.573202263 | 0.47955266 | 0.65102948 | 0.610441403 | 0.783403461 | 0.264521768 | 0.62039858 | 0.425043893 | 0.641201915 | 0.850183713 | 0.361007863 | 0.482865579 | 0.829596546 | 0.497898439 | 0.651056662 | 0.172612726 | 0.665747365 | 0.549460597 | 0.542475681 | 0.539218975 | 0.382165061 | 0.510435231 |
| GSE26939_GSM663374 | 0.265746053 | 0.436423327 | 0.495449755 | 0.624571066 | 0.55840653 | 0.674301664 | 0.244683717 | 0.591676042 | 0.450064858 | 0.599755612 | 0.72735508 | 0.408291307 | 0.460013958 | 0.73698715 | 0.444381337 | 0.641897735 | 0.229528035 | 0.645060164 | 0.632924534 | 0.512041315 | 0.478949839 | 0.321132353 | 0.51002453 |
| GSE26939_GSM663375 | 0.329106935 | 0.546989409 | 0.594304598 | 0.685303583 | 0.625621639 | 0.680619636 | 0.32645156 | 0.635524972 | 0.514615723 | 0.729485623 | 0.809478925 | 0.406807706 | 0.557622954 | 0.791729606 | 0.470365896 | 0.647227178 | 0.287179673 | 0.654673026 | 0.725486503 | 0.554630202 | 0.545951631 | 0.415293035 | 0.482410152 |
| GSE26939_GSM663376 | 0.775456883 | 0.656360212 | 0.689127867 | 0.68118062 | 0.591277222 | 0.653960834 | 0.403467649 | 0.589148862 | 0.789009768 | 0.694757513 | 0.83784826 | 0.448078869 | 0.550978876 | 0.836335958 | 0.485122093 | 0.697549694 | 0.126175251 | 0.725340592 | 0.713436053 | 0.631059069 | 0.5964041 | 0.367718407 | 0.447571418 |
| GSE26939_GSM663377 | 0.426733821 | 0.551041623 | 0.520975399 | 0.692936395 | 0.595819449 | 0.73385 | 0.318798516 | 0.622367592 | 0.523664854 | 0.677381644 | 0.881224303 | 0.437358741 | 0.493544767 | 0.748301745 | 0.461976322 | 0.651252666 | 0.264835878 | 0.666466939 | 0.680101096 | 0.536387786 | 0.520808154 | 0.33211365 | 0.48829397 |
| GSE26939_GSM663378 | 0.48718865 | 0.516222117 | 0.523438015 | 0.680411056 | 0.589900997 | 0.64650329 | 0.423345817 | 0.627024648 | 0.551458901 | 0.711819081 | 0.862616042 | 0.442009004 | 0.556682242 | 0.801994648 | 0.499759172 | 0.692158301 | 0.150528949 | 0.728821131 | 0.651148123 | 0.599044898 | 0.55612948 | 0.349070356 | 0.529231634 |
| GSE26939_GSM663379 | 0.21055117 | 0.3099859 | 0.430618707 | 0.635879789 | 0.600054955 | 0.626638073 | 0.306076218 | 0.574492261 | 0.418110853 | 0.725844716 | 0.659427358 | 0.420779079 | 0.587129225 | 0.748015755 | 0.415656984 | 0.661257839 | 0.193978748 | 0.716180546 | 0.611915289 | 0.551776238 | 0.4988659 | 0.431821161 | 0.505183678 |
| GSE26939_GSM663380 | 0.319101808 | 0.464803371 | 0.498958277 | 0.639424626 | 0.59984267 | 0.671726517 | 0.247650473 | 0.60463579 | 0.458898145 | 0.660083976 | 0.75410146 | 0.405809636 | 0.576764493 | 0.727270117 | 0.421549781 | 0.670631435 | 0.190102066 | 0.652763142 | 0.617966361 | 0.518756621 | 0.504709935 | 0.308422342 | 0.508188841 |
| GSE26939_GSM663381 | 0.160011432 | 0.405984111 | 0.468882296 | 0.590986824 | 0.568225386 | 0.719026408 | 0.165899663 | 0.573695673 | 0.357115631 | 0.650837042 | 0.614754422 | 0.344382067 | 0.371287392 | 0.764250184 | 0.422790226 | 0.640444748 | 0.21997574 | 0.624440811 | 0.490631396 | 0.50389281 | 0.45805674 | 0.257952221 | 0.488908206 |
| GSE26939_GSM663382 | 0.244359088 | 0.515985528 | 0.455150082 | 0.626369456 | 0.591435946 | 0.671050369 | 0.408177262 | 0.602519932 | 0.383949944 | 0.693291933 | 0.720030381 | 0.368904768 | 0.461032749 | 0.73242659 | 0.396390952 | 0.606273382 | 0.245497813 | 0.651601899 | 0.485045226 | 0.560492972 | 0.45379503 | 0.39033784 | 0.5324259 |
| GSE26939_GSM663383 | 0.252226873 | 0.438052954 | 0.479487068 | 0.720764856 | 0.67417503 | 0.702723363 | 0.264312813 | 0.623201355 | 0.459626766 | 0.71175687 | 0.834708219 | 0.495725174 | 0.540559356 | 0.76851476 | 0.487430355 | 0.690305682 | 0.165649321 | 0.717227877 | 0.686100083 | 0.602375297 | 0.577234561 | 0.411248873 | 0.455372 |
| GSE26939_GSM663384 | 0.43678416 | 0.622379961 | 0.713579529 | 0.713501492 | 0.64348425 | 0.664807396 | 0.30168993 | 0.653015208 | 0.578780023 | 0.711635348 | 0.935423641 | 0.481802267 | 0.620218729 | 0.784583825 | 0.537542684 | 0.7124807 | 0.173579181 | 0.745641203 | 0.748097348 | 0.605536375 | 0.613508263 | 0.35977124 | 0.512620504 |
| GSE26939_GSM663385 | 0.37535301 | 0.460855057 | 0.555567516 | 0.607333501 | 0.612921621 | 0.67263644 | 0.2025864 | 0.606129903 | 0.395919285 | 0.691484756 | 0.722614381 | 0.418209822 | 0.44976425 | 0.732819517 | 0.412156866 | 0.598369011 | 0.245101153 | 0.703673186 | 0.538052565 | 0.523808463 | 0.498603622 | 0.317347084 | 0.504771668 |
| GSE26939_GSM663386 | 0.541082053 | 0.601030884 | 0.737521733 | 0.721832038 | 0.66354129 | 0.687547726 | 0.336742914 | 0.66470842 | 0.615648313 | 0.658005462 | 0.947095522 | 0.474201279 | 0.486935104 | 0.804752549 | 0.546052782 | 0.704387549 | 0.219811725 | 0.743210152 | 0.724670228 | 0.604592394 | 0.616686611 | 0.353744881 | 0.452131489 |
| GSE26939_GSM663387 | 0.424932566 | 0.505648351 | 0.511636447 | 0.687468287 | 0.613224588 | 0.665196848 | 0.387722015 | 0.615455582 | 0.489357399 | 0.738689092 | 0.875436009 | 0.424880598 | 0.532102969 | 0.80479032 | 0.485789058 | 0.713962163 | 0.311322875 | 0.7330532 | 0.648408897 | 0.591918558 | 0.553477788 | 0.413914739 | 0.533979225 |
| GSE26939_GSM663388 | 0.257617179 | 0.359948244 | 0.429131874 | 0.621071778 | 0.552928181 | 0.60229191 | 0.298092915 | 0.591521035 | 0.406887514 | 0.659644371 | 0.704650484 | 0.428439501 | 0.5168254 | 0.735641216 | 0.389496665 | 0.608840591 | 0.235404748 | 0.697651134 | 0.492219475 | 0.554416501 | 0.481754544 | 0.467630833 | 0.420343317 |
| GSE26939_GSM663389 | 0.227041086 | 0.452380189 | 0.494769203 | 0.691623385 | 0.65432825 | 0.727016983 | 0.304372304 | 0.655275021 | 0.486289234 | 0.749876986 | 0.862363489 | 0.511138347 | 0.542871965 | 0.79686238 | 0.513276609 | 0.670986662 | 0.191189895 | 0.701427457 | 0.730648239 | 0.569852297 | 0.552887528 | 0.423167368 | 0.468344111 |
| GSE26939_GSM663390 | 0.332766662 | 0.546981786 | 0.468982378 | 0.642056904 | 0.662142031 | 0.631792863 | 0.173799778 | 0.549266043 | 0.431619604 | 0.57293461 | 0.74695255 | 0.416738236 | 0.467425078 | 0.678461623 | 0.441043703 | 0.635172265 | 0.075766436 | 0.645241091 | 0.593187212 | 0.536880804 | 0.480772939 | 0.369340323 | 0.503049811 |
| GSE26939_GSM663391 | 0.25487821 | 0.482940938 | 0.488891896 | 0.680485775 | 0.600577809 | 0.653525359 | 0.451842899 | 0.611706495 | 0.467894326 | 0.704480137 | 0.762833962 | 0.451941768 | 0.572260872 | 0.763391497 | 0.479126744 | 0.685589452 | 0.180621829 | 0.7006155 | 0.644551345 | 0.594504234 | 0.505852349 | 0.348854773 | 0.532740875 |
| GSE26939_GSM663392 | 0.185886496 | 0.477968909 | 0.431145495 | 0.598451793 | 0.576727975 | 0.737189771 | 0.218515479 | 0.605233677 | 0.332866701 | 0.664788305 | 0.613376367 | 0.359306667 | 0.398934966 | 0.706601316 | 0.425751513 | 0.558325363 | 0.315059809 | 0.648074341 | 0.551443932 | 0.501175557 | 0.441514376 | 0.452799159 | 0.615891123 |
| GSE26939_GSM663393 | 0.483881213 | 0.666420047 | 0.798279329 | 0.73563566 | 0.612296722 | 0.712713633 | 0.324303123 | 0.652353575 | 0.664957237 | 0.64315381 | 0.922324161 | 0.524471631 | 0.550426499 | 0.797414814 | 0.565010128 | 0.706152904 | 0.167822083 | 0.734944607 | 0.806497574 | 0.669432396 | 0.625791465 | 0.435075056 | 0.501318307 |
| GSE26939_GSM663394 | 0.404211807 | 0.48162261 | 0.54466683 | 0.67675794 | 0.610383044 | 0.610061192 | 0.449688458 | 0.651950679 | 0.52811081 | 0.7239118 | 0.795663285 | 0.405354896 | 0.612037159 | 0.763051027 | 0.468080938 | 0.682906739 | 0.161951058 | 0.726550487 | 0.674489121 | 0.581628091 | 0.532385375 | 0.387880197 | 0.604480734 |
| GSE26939_GSM663395 | 0.66090227 | 0.601650227 | 0.658473853 | 0.689424011 | 0.559298101 | 0.648518882 | 0.400001521 | 0.578234275 | 0.716922634 | 0.675444801 | 0.807457226 | 0.415518024 | 0.577254134 | 0.783378564 | 0.4840503 | 0.690078493 | 0.147444996 | 0.699260398 | 0.672260644 | 0.602759307 | 0.571664984 | 0.426466166 | 0.489691087 |
| GSE26939_GSM663396 | 0.24675473 | 0.45235854 | 0.436419905 | 0.607140117 | 0.677711606 | 0.599729164 | 0.264413097 | 0.587816951 | 0.383200133 | 0.771247758 | 0.5878 | 0.314268117 | 0.389953656 | 0.705399979 | 0.408639242 | 0.622988756 | 0.200061177 | 0.672469446 | 0.527925631 | 0.434411093 | 0.443562898 | 0.433763064 | 0.476775884 |
| GSE26939_GSM663397 | 0.324135468 | 0.452649084 | 0.537115289 | 0.712409764 | 0.634572385 | 0.71637214 | 0.280858305 | 0.612350182 | 0.506074365 | 0.706587881 | 0.792598541 | 0.420646744 | 0.500520039 | 0.758770053 | 0.448222381 | 0.647813548 | 0.225686972 | 0.677826826 | 0.68684256 | 0.567385317 | 0.504600516 | 0.415987228 | 0.541132971 |
| GSE26939_GSM663398 | 0.217180594 | 0.420306739 | 0.411565166 | 0.623199524 | 0.631666988 | 0.649997065 | 0.271302018 | 0.525514632 | 0.334793714 | 0.780241374 | 0.732649672 | 0.425838008 | 0.379353134 | 0.750953111 | 0.416693469 | 0.570794937 | 0.167159566 | 0.635205124 | 0.508715884 | 0.500438063 | 0.419301083 | 0.380941253 | 0.533486905 |
| GSE26939_GSM663399 | 0.357340498 | 0.541308092 | 0.519642826 | 0.641389505 | 0.636291743 | 0.624011575 | 0.411495631 | 0.588067137 | 0.480772891 | 0.681186607 | 0.752798168 | 0.367546791 | 0.492215927 | 0.720620506 | 0.415162752 | 0.66344004 | 0.174134331 | 0.691821342 | 0.610143568 | 0.504890259 | 0.517547012 | 0.36046822 | 0.59719613 |
