# Supplementary material for: Correlation analysis of m6A-modified regulators with immune microenvironment infiltrating cells in lung adenocarcinoma
Source: PLoS One. 2022 Feb 23;17(2):e0264384. doi: 10.1371/journal.pone.0264384 (PMC8865675; doi:10.1371/journal.pone.0264384)
Supplement: S1 Data — (ZIP) [file pone.0264384.s008.zip › raw data/Immunotherapy.docx]

**The gene sets used in TCGA for Immunotherapy**

|  | **ips_ctla4_neg_pd1_neg** | **ips_ctla4_neg_pd1_pos** | **ips_ctla4_pos_pd1_neg** | **ips_ctla4_pos_pd1_pos** |
| --- | --- | --- | --- | --- |
| TCGA-44-2662 | 5 | 5 | 5 | 5 |
| TCGA-05-5428 | 6 | 5 | 6 | 5 |
| TCGA-78-7155 | 6 | 5 | 5 | 4 |
| TCGA-78-7159 | 6 | 5 | 7 | 5 |
| TCGA-44-7667 | 6 | 5 | 6 | 5 |
| TCGA-05-4390 | 6 | 5 | 6 | 5 |
| TCGA-55-A490 | 6 | 5 | 6 | 5 |
| TCGA-55-8087 | 6 | 5 | 6 | 4 |
| TCGA-55-7910 | 6 | 5 | 6 | 4 |
| TCGA-86-7713 | 6 | 5 | 6 | 4 |
| TCGA-78-7535 | 7 | 5 | 6 | 5 |
| TCGA-86-7955 | 7 | 5 | 6 | 5 |
| TCGA-86-8054 | 7 | 5 | 6 | 5 |
| TCGA-44-5644 | 7 | 5 | 6 | 5 |
| TCGA-86-8279 | 7 | 5 | 7 | 5 |
| TCGA-55-8615 | 7 | 5 | 6 | 4 |
| TCGA-78-8662 | 7 | 5 | 6 | 5 |
| TCGA-95-7043 | 7 | 5 | 6 | 5 |
| TCGA-64-1678 | 7 | 5 | 7 | 5 |
| TCGA-62-A46V | 7 | 5 | 7 | 5 |
| TCGA-97-7937 | 7 | 5 | 6 | 5 |
| TCGA-NJ-A4YF | 7 | 5 | 6 | 5 |
| TCGA-73-4675 | 7 | 5 | 6 | 5 |
| TCGA-62-A46O | 7 | 5 | 7 | 5 |
| TCGA-62-A470 | 7 | 5 | 6 | 5 |
| TCGA-55-7570 | 7 | 5 | 7 | 6 |
| TCGA-44-8119 | 6 | 6 | 6 | 6 |
| TCGA-86-7711 | 6 | 6 | 6 | 5 |
| TCGA-78-7536 | 6 | 6 | 6 | 5 |
| TCGA-91-6831 | 6 | 6 | 6 | 6 |
| TCGA-44-8117 | 6 | 6 | 6 | 5 |
| TCGA-86-7953 | 6 | 6 | 6 | 5 |
| TCGA-62-8399 | 6 | 6 | 6 | 5 |
| TCGA-05-4415 | 6 | 6 | 6 | 5 |
| TCGA-49-6743 | 6 | 6 | 6 | 6 |
| TCGA-55-6543 | 6 | 6 | 6 | 5 |
| TCGA-44-7660 | 6 | 6 | 6 | 5 |
| TCGA-78-7146 | 6 | 6 | 6 | 6 |
| TCGA-44-6774 | 7 | 6 | 6 | 6 |
| TCGA-55-8096 | 7 | 6 | 6 | 5 |
| TCGA-J2-8192 | 7 | 6 | 7 | 6 |
| TCGA-44-6775 | 7 | 6 | 7 | 6 |
| TCGA-05-4420 | 7 | 6 | 7 | 5 |
| TCGA-55-8506 | 7 | 6 | 7 | 6 |
| TCGA-86-8056 | 7 | 6 | 7 | 6 |
| TCGA-05-4249 | 7 | 6 | 7 | 6 |
| TCGA-67-3771 | 7 | 6 | 7 | 6 |
| TCGA-73-4670 | 7 | 6 | 7 | 6 |
| TCGA-38-4626 | 7 | 6 | 6 | 6 |
| TCGA-55-A48Y | 7 | 6 | 7 | 6 |
| TCGA-97-8176 | 7 | 6 | 7 | 5 |
| TCGA-55-7724 | 7 | 6 | 7 | 6 |
| TCGA-J2-8194 | 7 | 6 | 6 | 5 |
