# Supplementary material for: Correlation analysis of m6A-modified regulators with immune microenvironment infiltrating cells in lung adenocarcinoma
Source: PLoS One. 2022 Feb 23;17(2):e0264384. doi: 10.1371/journal.pone.0264384 (PMC8865675; doi:10.1371/journal.pone.0264384)
Supplement: S1 Data — (ZIP) [file pone.0264384.s008.zip › raw data/Rcode.docx]

1. **Differential expressionanalysis(R codes)**

if (!requireNamespace("BiocManager", quietly = TRUE))

install.packages("BiocManager")

BiocManager::install("limma")

install.packages("VennDiagram")

library(limma)

library(VennDiagram)

expFile="merge.txt"

cluFile="m6aCluster.txt"

adj.P.Val.Filter=0.001

setwd("f")

rt=read.table(expFile, header=T, sep="\t", check.names=F)

rt=as.matrix(rt)

rownames(rt)=rt[,1]

exp=rt[,2:ncol(rt)]

dimnames=list(rownames(exp),colnames(exp))

data=matrix(as.numeric(as.matrix(exp)),nrow=nrow(exp),dimnames=dimnames)

data=avereps(data)

data=data[rowMeans(data)>0,]

cluster=read.table(cluFile, header=T, sep="\t", check.names=F, row.names=1)

sameSample=intersect(colnames(data), row.names(cluster))

data=data[,sameSample]

cluster=cluster[sameSample,]

logFCfilter=0

geneList=list()

Type=as.vector(cluster)

design=model.matrix(~0+factor(Type))

colnames(design)=levels(factor(Type))

comp=combn(levels(factor(Type)), 2)

allDiffGenes=c()

for(i in 1:ncol(comp)){

fit=lmFit(data, design)

contrast=paste0(comp[2,i], "-", comp[1,i])

#print(contrast)

cont.matrix=makeContrasts(contrast, levels=design)

fit2=contrasts.fit(fit, cont.matrix)

fit2=eBayes(fit2)

allDiff=topTable(fit2,adjust='fdr',number=200000)

allDiffOut=rbind(id=colnames(allDiff),allDiff)

write.table(allDiffOut, file=paste0(contrast, ".all.txt"), sep="\t", quote=F, col.names=F)

diffSig=allDiff[with(allDiff, (abs(logFC)>logFCfilter & adj.P.Val < adj.P.Val.Filter )), ]

diffSigOut=rbind(id=colnames(diffSig),diffSig)

write.table(diffSigOut, file=paste0(contrast, ".diff.txt"), sep="\t", quote=F, col.names=F)

geneList[[contrast]]=row.names(diffSig)

}

venn.plot=venn.diagram(geneList,filename=NULL,fill=rainbow(length(geneList)) )

pdf(file="venn.pdf", width=5, height=5)

grid.draw(venn.plot)

dev.off()

interGenes=Reduce(intersect,geneList)

write.table(file="interGene.txt",interGenes,sep="\t",quote=F,col.names=F,row.names=F)

interGeneExp=data[interGenes,]

interGeneExp=rbind(id=colnames(interGeneExp), interGeneExp)

write.table(interGeneExp, file="interGeneExp.txt", sep="\t", quote=F, col.names=F)

1. **GSVA(R codes)**

if (!requireNamespace("BiocManager", quietly = TRUE))

install.packages("BiocManager")

BiocManager::install("limma")

BiocManager::install("GSEABase")

BiocManager::install("GSVA")

install.packages("pheatmap")

library(limma)

library(GSEABase)

library(GSVA)

library(pheatmap)

expFile="merge.txt"

clusterFile="m6aCluster.txt"

gmtFile=""

setwd("")

rt=read.table(expFile, header=T, sep="\t", check.names=F)

rt=as.matrix(rt)

rownames(rt)=rt[,1]

exp=rt[,2:ncol(rt)]

dimnames=list(rownames(exp), colnames(exp))

data=matrix(as.numeric(as.matrix(exp)), nrow=nrow(exp), dimnames=dimnames)

data=avereps(data)

geneSets=getGmt(gmtFile, geneIdType=SymbolIdentifier())

gsvaResult=gsva(data,

geneSets,

min.sz=10,

max.sz=500,

verbose=TRUE,

parallel.sz=1)

gsvaOut=rbind(id=colnames(gsvaResult), gsvaResult)

write.table(gsvaOut, file="gsvaOut.txt", sep="\t", quote=F, col.names=F)

cluster=read.table(clusterFile, header=T, sep="\t", check.names=F, row.names=1)

gsvaResult=t(gsvaResult)

sameSample=intersect(row.names(gsvaResult), row.names(cluster))

gsvaResult=gsvaResult[sameSample,,drop=F]

cluster=cluster[sameSample,,drop=F]

gsvaCluster=cbind(gsvaResult, cluster)

Project=gsub("(.*?)\\_.*", "\\1", rownames(gsvaCluster))

gsvaCluster=cbind(gsvaCluster, Project)

adj.P.Val.Filter=0.05

allType=as.vector(gsvaCluster$m6Acluster)

comp=combn(levels(factor(allType)), 2)

for(i in 1:ncol(comp)){

treat=gsvaCluster[gsvaCluster$m6Acluster==comp[2,i],]

con=gsvaCluster[gsvaCluster$m6Acluster==comp[1,i],]

data=rbind(con, treat)

Type=as.vector(data$m6Acluster)

ann=data[,c(ncol(data), (ncol(data)-1))]

data=t(data[,-c((ncol(data)-1), ncol(data))])

design=model.matrix(~0+factor(Type))

colnames(design)=levels(factor(Type))

fit=lmFit(data, design)

contrast=paste0(comp[2,i], "-", comp[1,i])

cont.matrix=makeContrasts(contrast, levels=design)

fit2=contrasts.fit(fit, cont.matrix)

fit2=eBayes(fit2)

allDiff=topTable(fit2,adjust='fdr',number=200000)

allDiffOut=rbind(id=colnames(allDiff),allDiff)

write.table(allDiffOut, file=paste0(contrast, ".all.txt"), sep="\t", quote=F, col.names=F)

diffSig=allDiff[with(allDiff, (abs(logFC)>0.1 & adj.P.Val < adj.P.Val.Filter )), ]

diffSigOut=rbind(id=colnames(diffSig),diffSig)

write.table(diffSigOut, file=paste0(contrast, ".diff.txt"), sep="\t", quote=F, col.names=F)

bioCol=c("#0066FF","#FF9900","#FF0000","#6E568C","#7CC767","#223D6C","#D20A13","#FFD121","#088247","#11AA4D")

ann_colors=list()

m6aCluCol=bioCol[1:length(levels(factor(allType)))]

names(m6aCluCol)=levels(factor(allType))

ann_colors[["m6Acluster"]]=m6aCluCol[c(comp[1,i], comp[2,i])]

termNum=20

diffTermName=as.vector(rownames(diffSig))

diffLength=length(diffTermName)

if(diffLength<termNum){termNum=diffLength}

hmGene=diffTermName[1:termNum]

hmExp=data[hmGene,]

pdf(file=paste0(contrast,".heatmap.pdf"),height=6,width=10)

pheatmap(hmExp,

annotation=ann,

annotation_colors = ann_colors,

color = colorRampPalette(c(rep("blue",2), "white", rep("red",2)))(50),

cluster_cols =F,

show_colnames = F,

gaps_col=as.vector(cumsum(table(Type))),

scale="row",

fontsize = 10,

fontsize_row=7,

fontsize_col=10)

dev.off()

}

1. **ssGSEA(R code)**

if (!requireNamespace("BiocManager", quietly = TRUE))

# install.packages("BiocManager")

#BiocManager::install("limma")

#BiocManager::install("GSEABase")

#BiocManager::install("GSVA")

#install.packages("ggpubr")

library(reshape2)

library(ggpubr)

library(limma)

library(GSEABase)

library(GSVA)

expFile="merge.txt"

gmtFile="immune.gmt"

clusterFile="m6aCluster.txt"

setwd("")

rt=read.table(expFile, header=T, sep="\t", check.names=F)

rt=as.matrix(rt)

rownames(rt)=rt[,1]

exp=rt[,2:ncol(rt)]

dimnames=list(rownames(exp),colnames(exp))

data=matrix(as.numeric(as.matrix(exp)),nrow=nrow(exp),dimnames=dimnames)

data=avereps(data)

geneSets=getGmt(gmtFile, geneIdType=SymbolIdentifier())

ssgseaScore=gsva(data, geneSets, method='ssgsea', kcdf='Gaussian', abs.ranking=TRUE)

normalize=function(x){

return((x-min(x))/(max(x)-min(x)))}

ssgseaScore=normalize(ssgseaScore)

ssgseaOut=rbind(id=colnames(ssgseaScore), ssgseaScore)

write.table(ssgseaOut,file="ssGSEA.result.txt",sep="\t",quote=F,col.names=F)

cluster=read.table(clusterFile, header=T, sep="\t", check.names=F, row.names=1)

ssgseaScore=t(ssgseaScore)

sameSample=intersect(row.names(ssgseaScore), row.names(cluster))

ssgseaScore=ssgseaScore[sameSample,,drop=F]

cluster=cluster[sameSample,,drop=F]

scoreCluster=cbind(ssgseaScore, cluster)

data=melt(scoreCluster, id.vars=c("m6Acluster"))

colnames(data)=c("m6Acluster", "Immune", "Fraction")

bioCol=c("#0066FF","#FF9900","#FF0000","#6E568C","#7CC767","#223D6C","#D20A13","#FFD121","#088247","#11AA4D")

bioCol=bioCol[1:length(levels(factor(data[,"m6Acluster"])))]

p=ggboxplot(data, x="Immune", y="Fraction", color="m6Acluster",

ylab="Immune infiltration",

xlab="",

legend.title="m6Acluster",

palette=bioCol)

p=p+rotate_x_text(50)

pdf(file="boxplot.pdf", width=8, height=6.5)

p+stat_compare_means(aes(group=m6Acluster),symnum.args=list(cutpoints = c(0, 0.001, 0.01, 0.05, 1), symbols = c("***", "**", "*", "ns")),label = "p.signif")

dev.off()
