# Supplementary material for: Correlation analysis of m6A-modified regulators with immune microenvironment infiltrating cells in lung adenocarcinoma
Source: PLoS One. 2022 Feb 23;17(2):e0264384. doi: 10.1371/journal.pone.0264384 (PMC8865675; doi:10.1371/journal.pone.0264384)
Supplement: S1 Data — (ZIP) [file pone.0264384.s008.zip › raw data/TMB.docx]

**The gene sets used in this work for marking each TMB**

| **ID** | **TMB** | **ID** | **TMB** | **ID** | **TMB** |
| --- | --- | --- | --- | --- | --- |
| TCGA-75-7027 | 5.026315789 | TCGA-55-8087 | 0.5 | TCGA-55-8208 | 2.236842105 |
| TCGA-86-8055 | 0.5 | TCGA-78-7159 | 6.289473684 | TCGA-97-8176 | 2.684210526 |
| TCGA-49-4501 | 0.710526316 | TCGA-50-8457 | 1.052631579 | TCGA-73-7498 | 2.289473684 |
| TCGA-78-8640 | 13.52631579 | TCGA-75-6203 | 0.184210526 | TCGA-55-6982 | 3.026315789 |
| TCGA-86-8280 | 0.763157895 | TCGA-64-1679 | 9.657894737 | TCGA-38-4631 | 14.52631579 |
| TCGA-49-4510 | 0.894736842 | TCGA-53-7813 | 3.026315789 | TCGA-55-7725 | 2.736842105 |
| TCGA-64-5775 | 9.894736842 | TCGA-78-7146 | 7.473684211 | TCGA-05-4390 | 11.18421053 |
| TCGA-05-4398 | 11.26315789 | TCGA-J2-8194 | 3.105263158 | TCGA-17-Z004 | 0.289473684 |
| TCGA-17-Z015 | 10.84210526 | TCGA-86-7955 | 6.526315789 | TCGA-44-7669 | 9.131578947 |
| TCGA-55-8206 | 0.526315789 | TCGA-17-Z000 | 2.394736842 | TCGA-99-8032 | 7.447368421 |
| TCGA-67-3773 | 2.236842105 | TCGA-17-Z011 | 2.315789474 | TCGA-50-5944 | 1.236842105 |
| TCGA-55-6969 | 7.868421053 | TCGA-55-8092 | 10.36842105 | TCGA-49-4488 | 5.947368421 |
| TCGA-62-A470 | 2.394736842 | TCGA-55-A48Z | 0.789473684 | TCGA-95-8494 | 1.815789474 |
| TCGA-73-4658 | 3.578947368 | TCGA-69-A59K | 9.473684211 | TCGA-55-8097 | 2.026315789 |
| TCGA-05-4403 | 2.921052632 | TCGA-49-4505 | 3.710526316 | TCGA-44-A47G | 0.578947368 |
| TCGA-71-8520 | 1.710526316 | TCGA-38-4625 | 11.26315789 | TCGA-17-Z048 | 0.631578947 |
| TCGA-97-A4M1 | 0.631578947 | TCGA-67-6217 | 3.368421053 | TCGA-55-8508 | 3.5 |
| TCGA-38-A44F | 0.078947368 | TCGA-55-8615 | 2 | TCGA-69-8253 | 2.789473684 |
| TCGA-17-Z060 | 5.605263158 | TCGA-69-7973 | 5.657894737 | TCGA-55-7284 | 0.815789474 |
| TCGA-49-6745 | 1.894736842 | TCGA-50-5045 | 4.736842105 | TCGA-86-7713 | 3.447368421 |
| TCGA-55-7574 | 2.368421053 | TCGA-44-7662 | 10.81578947 | TCGA-44-6778 | 7.289473684 |
| TCGA-49-AAR2 | 8.736842105 | TCGA-49-AAQV | 0.763157895 | TCGA-55-A491 | 6.815789474 |
| TCGA-35-4122 | 4.684210526 | TCGA-78-7540 | 0.631578947 | TCGA-MP-A4TC | 5.026315789 |
| TCGA-83-5908 | 4.157894737 | TCGA-05-4426 | 1 | TCGA-44-3917 | 4.078947368 |
| TCGA-55-8505 | 0.605263158 | TCGA-49-AARO | 11 | TCGA-99-7458 | 9.105263158 |
| TCGA-17-Z045 | 13.44736842 | TCGA-75-6212 | 0.421052632 | TCGA-97-8552 | 0.5 |
| TCGA-55-6972 | 5.210526316 | TCGA-05-5420 | 1.263157895 | TCGA-55-7227 | 3.368421053 |
| TCGA-78-7161 | 3.473684211 | TCGA-86-8358 | 22.55263158 | TCGA-50-6595 | 1 |
| TCGA-44-5644 | 20.78947368 | TCGA-44-2655 | 2.473684211 | TCGA-44-3919 | 0.657894737 |
| TCGA-44-6775 | 0.947368421 | TCGA-73-4662 | 4.236842105 | TCGA-64-5778 | 10.05263158 |
| TCGA-17-Z023 | 7.842105263 | TCGA-05-4420 | 5.552631579 | TCGA-97-7553 | 0.578947368 |
| TCGA-86-8585 | 11 | TCGA-L9-A443 | 5 | TCGA-05-4424 | 12 |
| TCGA-86-A4P7 | 0.763157895 | TCGA-55-7724 | 2.105263158 | TCGA-17-Z053 | 6.552631579 |
| TCGA-17-Z037 | 2.368421053 | TCGA-64-1677 | 3.605263158 | TCGA-05-4395 | 5.342105263 |
| TCGA-95-A4VK | 5.026315789 | TCGA-50-8459 | 1.473684211 | TCGA-55-7903 | 2.842105263 |
| TCGA-38-7271 | 0.552631579 | TCGA-50-5066 | 4.447368421 | TCGA-05-4250 | 7.105263158 |
| TCGA-86-8278 | 1.157894737 | TCGA-17-Z041 | 2.078947368 | TCGA-17-Z018 | 5.052631579 |
| TCGA-80-5608 | 3.5 | TCGA-35-3615 | 2.5 | TCGA-55-8514 | 5.710526316 |
| TCGA-J2-A4AG | 1.157894737 | TCGA-73-4677 | 5.184210526 | TCGA-78-7152 | 3.342105263 |
| TCGA-97-A4M6 | 0.473684211 | TCGA-55-8089 | 21.71052632 | TCGA-MP-A4SW | 0.842105263 |
| TCGA-35-4123 | 5.368421053 | TCGA-55-8510 | 5.315789474 | TCGA-44-2662 | 3.026315789 |
| TCGA-91-6840 | 2.394736842 | TCGA-86-8056 | 5.105263158 | TCGA-55-7726 | 2.052631579 |
| TCGA-44-A4SU | 2.473684211 | TCGA-55-A494 | 5.026315789 | TCGA-97-8175 | 0.526315789 |
| TCGA-49-AAR3 | 1.421052632 | TCGA-95-7043 | 23.57894737 | TCGA-44-7667 | 8.5 |
| TCGA-50-8460 | 0.578947368 | TCGA-44-A4SS | 6.894736842 | TCGA-05-4249 | 6.868421053 |
| TCGA-62-A46P | 5.526315789 | TCGA-55-8205 | 8.447368421 | TCGA-38-6178 | 0.657894737 |
| TCGA-55-8302 | 11.34210526 | TCGA-17-Z016 | 6.526315789 | TCGA-44-7672 | 2.026315789 |
| TCGA-86-8674 | 6.131578947 | TCGA-NJ-A4YF | 12.10526316 | TCGA-97-7552 | 0.315789474 |
| TCGA-86-8281 | 4.526315789 | TCGA-44-A479 | 4.736842105 | TCGA-17-Z052 | 1.052631579 |
| TCGA-MP-A4T7 | 2.657894737 | TCGA-17-Z057 | 7.342105263 | TCGA-17-Z014 | 7.368421053 |
| TCGA-78-7145 | 4.052631579 | TCGA-50-5941 | 5.447368421 | TCGA-MP-A4TI | 2.578947368 |
| TCGA-17-Z005 | 2.473684211 | TCGA-55-7907 | 24.10526316 | TCGA-05-4430 | 4.052631579 |
| TCGA-53-7624 | 17.42105263 | TCGA-17-Z001 | 4.263157895 | TCGA-91-6829 | 12.39473684 |
| TCGA-50-6594 | 11.63157895 | TCGA-17-Z010 | 5.026315789 | TCGA-64-5774 | 2.947368421 |
| TCGA-91-A4BC | 6.868421053 | TCGA-05-4434 | 0.631578947 | TCGA-78-7153 | 1.736842105 |
| TCGA-17-Z022 | 24.81578947 | TCGA-55-7728 | 1.868421053 | TCGA-86-8054 | 4.710526316 |
| TCGA-62-A471 | 1.894736842 | TCGA-55-A4DG | 13.39473684 | TCGA-05-5428 | 7.473684211 |
| TCGA-NJ-A4YP | 9.342105263 | TCGA-55-7281 | 9.421052632 | TCGA-55-A490 | 25.55263158 |
| TCGA-49-4506 | 2.026315789 | TCGA-97-7546 | 5.5 | TCGA-62-A46U | 0.710526316 |
| TCGA-17-Z061 | 3.684210526 | TCGA-78-8660 | 4.184210526 | TCGA-50-5068 | 1.631578947 |
| TCGA-55-1592 | 10.57894737 | TCGA-55-8506 | 34.84210526 | TCGA-49-AARN | 9.236842105 |
| TCGA-49-6761 | 3.131578947 | TCGA-17-Z059 | 3.289473684 | TCGA-44-8119 | 12.89473684 |
| TCGA-55-8616 | 9.263157895 | TCGA-49-4494 | 3.552631579 | TCGA-69-7979 | 29.52631579 |
| TCGA-38-4626 | 1.578947368 | TCGA-49-4486 | 2.263157895 | TCGA-64-5781 | 21.42105263 |
| TCGA-78-7539 | 8.868421053 | TCGA-55-5899 | 9.052631579 | TCGA-L9-A7SV | 31.5 |
| TCGA-97-A4M0 | 7.710526316 | TCGA-44-6776 | 4.210526316 | TCGA-53-A4EZ | 10.39473684 |
| TCGA-55-6987 | 2.947368421 | TCGA-62-A46Y | 0.815789474 | TCGA-78-7633 | 2.447368421 |
| TCGA-78-7537 | 3.105263158 | TCGA-55-8511 | 10.15789474 | TCGA-55-7913 | 6.473684211 |
| TCGA-78-7166 | 4.552631579 | TCGA-95-A4VP | 2.052631579 | TCGA-99-8033 | 3.394736842 |
| TCGA-05-4425 | 0.842105263 | TCGA-93-A4JQ | 0.710526316 | TCGA-17-Z044 | 2.210526316 |
| TCGA-91-8496 | 0.394736842 | TCGA-99-AA5R | 0.052631579 | TCGA-55-7815 | 2.421052632 |
| TCGA-67-3772 | 0.921052632 | TCGA-55-A492 | 4.947368421 | TCGA-78-7148 | 4.315789474 |
| TCGA-NJ-A55A | 0.684210526 | TCGA-17-Z049 | 12.55263158 | TCGA-44-8117 | 16.15789474 |
| TCGA-50-5939 | 1.447368421 | TCGA-17-Z056 | 5.815789474 | TCGA-NJ-A4YQ | 19.55263158 |
| TCGA-44-2656 | 14.18421053 | TCGA-91-6831 | 5.631578947 | TCGA-49-6744 | 2.868421053 |
| TCGA-05-5425 | 9.894736842 | TCGA-97-7941 | 1.578947368 | TCGA-MP-A5C7 | 1.342105263 |
| TCGA-78-7160 | 0.526315789 | TCGA-44-4112 | 8.342105263 | TCGA-L4-A4E5 | 6.894736842 |
| TCGA-05-4402 | 1.157894737 | TCGA-17-Z017 | 4.684210526 | TCGA-49-4490 | 0.473684211 |
| TCGA-97-8174 | 2.921052632 | TCGA-L9-A444 | 9.842105263 | TCGA-75-5122 | 1.526315789 |
| TCGA-86-8073 | 26.84210526 | TCGA-05-4432 | 9.552631579 | TCGA-75-6207 | 1.210526316 |
| TCGA-MP-A4T9 | 0.184210526 | TCGA-55-7911 | 4.131578947 | TCGA-44-6774 | 5.710526316 |
| TCGA-44-8120 | 12.81578947 | TCGA-17-Z050 | 3.105263158 | TCGA-44-5645 | 0.789473684 |
| TCGA-93-8067 | 9.710526316 | TCGA-73-4668 | 12.23684211 | TCGA-44-6147 | 0.868421053 |
| TCGA-97-7554 | 8.5 | TCGA-75-7025 | 0.447368421 | TCGA-91-8499 | 7.026315789 |
| TCGA-78-7158 | 4.789473684 | TCGA-64-5815 | 3.236842105 | TCGA-L9-A5IP | 6.026315789 |
| TCGA-55-8091 | 0.473684211 | TCGA-44-6779 | 1.973684211 | TCGA-49-4507 | 3.894736842 |
| TCGA-17-Z054 | 0.105263158 | TCGA-44-2661 | 0.552631579 | TCGA-50-5936 | 2.263157895 |
| TCGA-44-3396 | 2.605263158 | TCGA-95-7562 | 9.157894737 | TCGA-55-7573 | 0.973684211 |
| TCGA-49-AARQ | 14.39473684 | TCGA-17-Z019 | 0.026315789 | TCGA-97-7937 | 9.763157895 |
| TCGA-MP-A4TK | 5.789473684 | TCGA-44-6144 | 4.315789474 | TCGA-67-6215 | 1.921052632 |
| TCGA-55-7995 | 13.92105263 | TCGA-44-7671 | 4.894736842 | TCGA-J2-A4AE | 0.631578947 |
| TCGA-86-6851 | 17.76315789 | TCGA-44-6777 | 5.657894737 | TCGA-38-4627 | 0.368421053 |
| TCGA-55-8620 | 10.05263158 | TCGA-93-A4JN | 1.289473684 | TCGA-91-6847 | 1.078947368 |
| TCGA-86-8075 | 0.710526316 | TCGA-55-A48X | 5.973684211 | TCGA-86-8672 | 5.315789474 |
| TCGA-44-2668 | 5.842105263 | TCGA-44-A47A | 3.5 | TCGA-05-4389 | 3.842105263 |
| TCGA-55-6543 | 0.421052632 | TCGA-44-3918 | 15.94736842 | TCGA-49-AARE | 29 |
| TCGA-62-8395 | 1.184210526 | TCGA-64-5779 | 7.5 | TCGA-MP-A4T6 | 1.210526316 |
| TCGA-05-5423 | 3.289473684 | TCGA-L9-A50W | 0.605263158 | TCGA-50-6592 | 4.947368421 |
| TCGA-75-6206 | 1.894736842 | TCGA-78-8655 | 2.184210526 | TCGA-44-5643 | 2.605263158 |
| TCGA-86-8359 | 3.157894737 | TCGA-97-7547 | 3.078947368 | TCGA-55-8085 | 5.368421053 |
| TCGA-17-Z003 | 4.105263158 | TCGA-75-5126 | 9.526315789 | TCGA-91-6835 | 0.736842105 |
| TCGA-MN-A4N4 | 24.26315789 | TCGA-50-7109 | 4.657894737 | TCGA-55-1594 | 4.105263158 |
| TCGA-44-6146 | 0.631578947 | TCGA-49-4487 | 4.868421053 | TCGA-97-A4M7 | 5.368421053 |
| TCGA-NJ-A55O | 1.736842105 | TCGA-69-7974 | 9.526315789 | TCGA-MP-A4TA | 6.052631579 |
| TCGA-55-7283 | 3.526315789 | TCGA-17-Z047 | 0.236842105 | TCGA-69-7760 | 1.236842105 |
| TCGA-17-Z042 | 9.342105263 | TCGA-55-8507 | 19.68421053 | TCGA-55-6978 | 0.368421053 |
| TCGA-69-8254 | 1.526315789 | TCGA-86-7701 | 3.868421053 | TCGA-05-4418 | 5.552631579 |
| TCGA-49-6742 | 4.763157895 | TCGA-50-5935 | 0.631578947 | TCGA-44-2659 | 8.605263158 |
| TCGA-86-7714 | 0.105263158 | TCGA-69-7765 | 10.73684211 | TCGA-50-5930 | 21.60526316 |
| TCGA-78-7220 | 16.94736842 | TCGA-50-6673 | 1.052631579 | TCGA-78-7155 | 33.05263158 |
| TCGA-44-7661 | 4.447368421 | TCGA-91-6828 | 5.315789474 | TCGA-S2-AA1A | 2.184210526 |
| TCGA-95-7567 | 16.39473684 | TCGA-44-6148 | 0.105263158 | TCGA-73-4675 | 1.289473684 |
| TCGA-78-8662 | 23.76315789 | TCGA-67-6216 | 0.394736842 | TCGA-MP-A4TH | 2.157894737 |
| TCGA-64-1680 | 0.763157895 | TCGA-91-6830 | 3 | TCGA-55-6980 | 0.184210526 |
| TCGA-55-8299 | 0.210526316 | TCGA-91-A4BD | 0.473684211 | TCGA-44-2657 | 6.815789474 |
| TCGA-55-6975 | 1.631578947 | TCGA-J2-8192 | 1.236842105 | TCGA-67-3774 | 2.078947368 |
| TCGA-05-4415 | 4.210526316 | TCGA-55-8094 | 9.263157895 | TCGA-NJ-A4YG | 2.052631579 |
| TCGA-75-6211 | 7.263157895 | TCGA-55-7910 | 6.526315789 | TCGA-50-5049 | 11.73684211 |
| TCGA-55-7914 | 3.736842105 | TCGA-17-Z051 | 5.157894737 | TCGA-55-8619 | 0.078947368 |
| TCGA-55-8090 | 1.763157895 | TCGA-69-7978 | 8.315789474 | TCGA-95-7944 | 4.657894737 |
| TCGA-17-Z002 | 0.078947368 | TCGA-62-A46O | 18.34210526 | TCGA-93-7347 | 1.973684211 |
| TCGA-50-5942 | 0.710526316 | TCGA-78-7147 | 6.684210526 | TCGA-38-4629 | 5.684210526 |
| TCGA-17-Z043 | 3.842105263 | TCGA-91-6836 | 10.02631579 | TCGA-91-6849 | 2.394736842 |
| TCGA-49-6743 | 13.15789474 | TCGA-91-7771 | 2.763157895 | TCGA-78-7167 | 3.578947368 |
| TCGA-55-8621 | 0.157894737 | TCGA-17-Z007 | 1.421052632 | TCGA-78-7536 | 12.60526316 |
| TCGA-MP-A4TF | 7.947368421 | TCGA-86-A456 | 5.947368421 | TCGA-91-8497 | 0.763157895 |
| TCGA-55-1595 | 6.447368421 | TCGA-05-4244 | 4.578947368 | TCGA-17-Z031 | 48.31578947 |
| TCGA-93-A4JO | 0.394736842 | TCGA-NJ-A4YI | 9.210526316 | TCGA-86-8669 | 2.473684211 |
| TCGA-86-6562 | 0.710526316 | TCGA-99-8028 | 2.789473684 | TCGA-55-A4DF | 13.60526316 |
| TCGA-55-8096 | 2.131578947 | TCGA-05-5429 | 0.631578947 | TCGA-44-2665 | 0.5 |
| TCGA-17-Z025 | 4.657894737 | TCGA-78-7156 | 5.526315789 | TCGA-97-8172 | 5.078947368 |
| TCGA-MP-A4SV | 4.421052632 | TCGA-64-1676 | 12.26315789 | TCGA-55-6986 | 0.368421053 |
| TCGA-17-Z013 | 1.184210526 | TCGA-17-Z009 | 0.184210526 | TCGA-62-8398 | 1.815789474 |
| TCGA-05-4422 | 0.763157895 | TCGA-78-7149 | 2.473684211 | TCGA-L9-A743 | 1.157894737 |
| TCGA-17-Z058 | 4.894736842 | TCGA-97-8547 | 1.105263158 | TCGA-86-A4JF | 23.47368421 |
| TCGA-78-7154 | 4.394736842 | TCGA-73-4676 | 1.921052632 | TCGA-17-Z055 | 7.736842105 |
| TCGA-55-7727 | 4.315789474 | TCGA-55-A493 | 3.368421053 | TCGA-62-8402 | 0.815789474 |
| TCGA-55-8512 | 0.789473684 | TCGA-69-7980 | 15.81578947 | TCGA-MP-A4TJ | 1.184210526 |
| TCGA-44-7660 | 6.236842105 | TCGA-50-5072 | 3.210526316 | TCGA-62-8394 | 1.342105263 |
| TCGA-L9-A8F4 | 14.44736842 | TCGA-75-6214 | 13.34210526 | TCGA-55-8207 | 6.473684211 |
| TCGA-78-7542 | 7.184210526 | TCGA-44-3398 | 1.789473684 | TCGA-69-7761 | 0.473684211 |
| TCGA-73-4666 | 7.789473684 | TCGA-44-7670 | 22.89473684 | TCGA-86-8074 | 1.421052632 |
| TCGA-05-4405 | 6.5 | TCGA-50-5055 | 0.078947368 | TCGA-50-5931 | 6.236842105 |
| TCGA-64-1681 | 0.947368421 | TCGA-78-7535 | 3.210526316 | TCGA-49-6767 | 8.763157895 |
| TCGA-55-6968 | 8.763157895 | TCGA-05-4427 | 19.94736842 | TCGA-55-7994 | 31.89473684 |
| TCGA-17-Z020 | 1.447368421 | TCGA-05-4433 | 0.763157895 | TCGA-95-7948 | 2.868421053 |
| TCGA-55-6642 | 2.605263158 | TCGA-MN-A4N1 | 8.552631579 | TCGA-71-6725 | 0.842105263 |
| TCGA-50-6597 | 0.815789474 | TCGA-55-6985 | 8.236842105 | TCGA-49-AAR4 | 6.368421053 |
| TCGA-80-5607 | 2.5 | TCGA-73-4670 | 7.868421053 | TCGA-55-6981 | 1.078947368 |
| TCGA-44-7659 | 3.157894737 | TCGA-86-7711 | 4 | TCGA-38-4632 | 9.473684211 |
| TCGA-MP-A4TD | 1.184210526 | TCGA-44-2666 | 0.473684211 | TCGA-69-8255 | 5.894736842 |
| TCGA-69-7764 | 1.552631579 | TCGA-78-7150 | 8.315789474 | TCGA-86-8673 | 9.184210526 |
| TCGA-97-A4M2 | 0.078947368 | TCGA-49-AAR9 | 13.57894737 | TCGA-50-5051 | 1.973684211 |
| TCGA-NJ-A55R | 4.842105263 | TCGA-73-A9RS | 19.18421053 | TCGA-35-5375 | 8.657894737 |
| TCGA-95-7039 | 24.42105263 | TCGA-55-8204 | 3.868421053 | TCGA-MN-A4N5 | 8.894736842 |
| TCGA-55-1596 | 4.078947368 | TCGA-50-6591 | 0.973684211 | TCGA-17-Z036 | 1.631578947 |
| TCGA-97-8179 | 3.552631579 | TCGA-62-8397 | 0.605263158 | TCGA-50-6593 | 5.605263158 |
| TCGA-75-5147 | 0.736842105 | TCGA-93-7348 | 1.605263158 | TCGA-55-A57B | 0.736842105 |
| TCGA-17-Z026 | 14 | TCGA-86-8671 | 0.026315789 | TCGA-17-Z030 | 8.736842105 |
| TCGA-MP-A4T4 | 5.894736842 | TCGA-49-4514 | 6 | TCGA-62-A46V | 0.973684211 |
| TCGA-95-A4VN | 6.710526316 | TCGA-55-6979 | 2 | TCGA-O1-A52J | 3.263157895 |
| TCGA-73-4659 | 4.236842105 | TCGA-55-6983 | 1.684210526 | TCGA-73-7499 | 1.263157895 |
| TCGA-05-4382 | 26.52631579 | TCGA-17-Z032 | 2.052631579 | TCGA-75-5125 | 2.868421053 |
| TCGA-67-4679 | 5.815789474 | TCGA-55-A48Y | 5.578947368 | TCGA-62-A46R | 3.421052632 |
| TCGA-55-8203 | 8.815789474 | TCGA-05-4417 | 5.921052632 | TCGA-17-Z028 | 8.842105263 |
| TCGA-55-6971 | 1.605263158 | TCGA-55-7576 | 6.552631579 | TCGA-J2-A4AD | 5.184210526 |
| TCGA-78-7162 | 1.052631579 | TCGA-50-5933 | 8.921052632 | TCGA-17-Z033 | 2.052631579 |
| TCGA-97-8177 | 0.368421053 | TCGA-4B-A93V | 2.921052632 | TCGA-80-5611 | 3.894736842 |
| TCGA-86-8279 | 10.55263158 | TCGA-97-8171 | 1.078947368 | TCGA-86-7954 | 2.789473684 |
| TCGA-55-6984 | 0.342105263 | TCGA-69-7763 | 1.184210526 | TCGA-50-6590 | 15.31578947 |
| TCGA-05-4397 | 17.86842105 | TCGA-62-8399 | 10.34210526 | TCGA-17-Z027 | 1.973684211 |
| TCGA-67-3770 | 4.342105263 | TCGA-38-4630 | 2.473684211 | TCGA-55-6712 | 1.605263158 |
| TCGA-62-A472 | 1.105263158 | TCGA-55-7570 | 8.131578947 | TCGA-50-5932 | 1.684210526 |
| TCGA-17-Z021 | 1.868421053 | TCGA-86-8668 | 0.605263158 | TCGA-93-A4JP | 0.368421053 |
| TCGA-78-7163 | 0.315789474 | TCGA-44-A47B | 0.973684211 | TCGA-50-5044 | 1.368421053 |
| TCGA-NJ-A7XG | 0.552631579 | TCGA-91-6848 | 11.47368421 | TCGA-55-8614 | 4.157894737 |
| TCGA-17-Z062 | 5.921052632 | TCGA-38-4628 | 2.263157895 | TCGA-05-5715 | 2.368421053 |
| TCGA-49-4512 | 0.526315789 | TCGA-97-7938 | 7.815789474 | TCGA-62-A46S | 2.157894737 |
| TCGA-55-8301 | 4.236842105 | TCGA-75-5146 | 3.763157895 | TCGA-53-7626 | 7.684210526 |
| TCGA-95-8039 | 1.789473684 | TCGA-44-A47F | 1.342105263 | TCGA-MP-A4T2 | 1.131578947 |
| TCGA-86-8076 | 0.921052632 | TCGA-44-6145 | 6.026315789 | TCGA-95-7947 | 11.26315789 |
| TCGA-75-6205 | 0.421052632 | TCGA-97-A4M3 | 4.157894737 | TCGA-05-4396 | 5.763157895 |
| TCGA-05-4410 | 19.23684211 | TCGA-49-AAR0 | 1.815789474 | TCGA-05-4384 | 2.289473684 |
| TCGA-55-6970 | 3.578947368 | TCGA-75-7031 | 4.210526316 | TCGA-MP-A4SY | 2.263157895 |
| TCGA-86-7953 | 0.921052632 | TCGA-MP-A4TE | 2.736842105 | TCGA-50-5946 | 12.84210526 |
| TCGA-97-A4M5 | 2.184210526 | TCGA-MP-A4T8 | 2.368421053 | TCGA-99-8025 | 9.447368421 |
| TCGA-97-A4LX | 1.842105263 | TCGA-67-3771 | 20.63157895 |  |  |
