# Supplementary material for: Correlation analysis of m6A-modified regulators with immune microenvironment infiltrating cells in lung adenocarcinoma
Source: PLoS One. 2022 Feb 23;17(2):e0264384. doi: 10.1371/journal.pone.0264384 (PMC8865675; doi:10.1371/journal.pone.0264384)
Supplement: S1 Data — (ZIP) [file pone.0264384.s008.zip › raw data/Description.doc]

The following is a brief description of the contents of this folder:

1. TCGA-LUAD (annotated document)

2.GEO dataset (platform files and matrix files)

3.TCIA dataset (TCIA-ClinicalData)

4. ssGSEA subsidiary files (immune.gmt)

5. GSVA files (c5.go.v7.4.symbols)

6.GSVA enrichment analysis

7.The gene sets used in TCGA for Immunotherapy

8. The gene sets used in this work for marking each TMB

9. Copy number of 23 regulators in TCGA-LUAD

10.R-Code（Differential expressionanalysis、GSVA、ssGSEA）
