# Supplementary material for: Correlation analysis of m6A-modified regulators with immune microenvironment infiltrating cells in lung adenocarcinoma
Source: PLoS One. 2022 Feb 23;17(2):e0264384. doi: 10.1371/journal.pone.0264384 (PMC8865675; doi:10.1371/journal.pone.0264384)
Supplement: Attachment — Submitted filename: Response to Reviewers.docx [file pone.0264384.s009.docx]

**Dear editors and reviewers**

Thank you very much for your practical advice on the article. We have made as many changes as possible to your suggestions and will respond to each of them：

1. **There is a necessity that the authors provide a detailed step by step visualized guidelines or video recording determines:
   1-1-How the data is searched (search key words) in TCGA and GEO
   1-2-How the data is downloaded
   The mentioned guideline can be uploaded as Supplementary file.**

ANSWER:The description of the data collection is supplemented in the Methods and Materials section. Further, visual graphics were made for representation and uploaded as an Supplementary file（Figure-S1）.

2. **Detailed references of R packages used for GSVA analysis and ssGSEA analysis as well as differential expression using R limma package need to be provided.
   R codes needs to be attached.
   I do suggest to provide step by step visualized guidelines or video recording**

ANSWER:Detailed references for GSVA analysis and ssGSEA analysis and R packages for differential expression using the limma package have been described in the Methods and Materials section and are accompanied by the R code. **3.All downloaded raw data need to be shared.**
ANSWER:All materials have been attached and uploaded as follows：

1. TCGA-LUAD (annotated document)

2.GEO dataset (platform files and matrix files)

3.TCIA dataset (TCIA-ClinicalData)

4. ssGSEA subsidiary files (immune.gmt)

5. GSVA files (c5.go.v7.4.symbols)

4. **A detailed step by step visualized guidelines or video recording of performing Survival analysis and Survival curves needs to be provided.**
   ANSWER:Details of the survival analysis and survival curve are described in detail in the methods and materials section.
5. **Data of two immunotherapy cohorts needs to be provided, including raw data as well as cohort statistics.**
   ANSWER：Raw data has been uploaded via attachments as well as statistical data as Supplementary file. **6.Quality of figures need to be improved.**
   ANSWER:Figure has been appropriately modified.
   **7.A flowchart describing all steps of analysis needs to be provided.**
   ANSWER:We have added the flow chart and uploaded it as an Supplementary file（Figure-S2）.
8. **Please upload a new copy of Figures 1, 3, 4, 5, 6, 7 and 8  as the detail are not clear.**

ANSWER:Revise according to the requirements of the magazine, and use the PACE website to check and re-upload, if there is still a shortage of images please contact, we will promptly revise to ensure the high quality of the manuscript.

In response to your very constructive suggestions, we completed the request as much as possible. However, there are still shortcomings.If you have any questions, please feel free to contact us and we will make changes in time to ensure the smooth progress of the review.

Sincere thanks！

Correspondence author: T.P. Huang
