# Supplementary material for: Correlation analysis of m6A-modified regulators with immune microenvironment infiltrating cells in lung adenocarcinoma
Source: PLoS One. 2022 Feb 23;17(2):e0264384. doi: 10.1371/journal.pone.0264384 (PMC8865675; doi:10.1371/journal.pone.0264384)
Supplement: Attachment — Submitted filename: Response to Reviewers.docx [file pone.0264384.s010.docx]

Dear Editor

Many thanks for the suggestions on the manuscript that can improve the quality of the manuscript. In response to the returned suggestions, the following corrections were made, some of which are marked in red in the manuscript, with the following descriptions：

1. The manuscript has been evaluated by the reveiwers and requires some minor revisions including typos and table formatting. Kindly pay attention to these comments and resubmit the manuscript with all requested changes.

Re: Correction of the manuscript for punctuation and grammar. The table format was corrected according to the journal's requirements and some adjustments were made to make the manuscript more logical.

2. Can you please upload an additional copy of your revised manuscript that does not contain any tracked changes or highlighting as your main article file. This will be used in the production process if your manuscript is accepted.

Re: I apologize that the manuscript was not uploaded correctly due to my carelessness. I have now uploaded the correct version. Also, I have doubts about the file type of the revised manuscript (with markers) that you pointed out. However, I will correct the file type according to the website you provided.

If there are still any questions, feel free to contact us and your comments are welcome!

Sincerethanks

TianPeng Huang
